# Integrated transcriptomic profiling of programmed cell death patterns unveils macrophage-hepatocyte crosstalk via THBS1-CD47 axis in hepatic ischemia-reperfusion injury

**DOI:** 10.3389/fimmu.2026.1769849

**Published:** 2026-05-19

**Authors:** Manling Xie, Changquan Zhang, Lirong Zhu, Yongfeng Pei, Chunyan Liang, Lixin Fu, Haibin Li, Liugen Lan, Ning Wen, Jihua Wu, Xuyong Sun

**Affiliations:** 1 School of Basic Medicine, Guangxi Medical University, Nanning, China; 2 Institute of Transplant Medicine, The Second Affiliated Hospital of Guangxi Medical University, Nanning, China; 3 Guangxi Clinical Research Center for Organ Transplantation, Nanning, China; 4 Guangxi Key Laboratory of Organ Donation and Transplantation, Nanning, China

**Keywords:** hepatic ischemia-reperfusion injury, macrophage-hepatocyte crosstalk, PI3K-AKT- NF-κB signaling, programmed cell death, THBS1-CD47 axis

## Abstract

**Background:**

Hepatic ischemia-reperfusion injury (HIRI) is clinically linked to post-transplant complications, yet the pathogenic role of programmed cell death (PCD) patterns in this process remains poorly delineated. This study aimed to investigate the diversity of programmed cell death (PCD) patterns underlying HIRI, with a focus on mechanistically dissecting macrophage-hepatocyte crosstalk mediated by the THBS1-CD47 axis.

**Methods:**

GSE151648, GSE14951, GSE12720 and GSE171539 were retrieved from the Gene Expression Omnibus (GEO) database. Based on bulk transcriptomic data, we identified differentially expressed PCD-related genes (DE-PCDRGs) in HIRI samples and performed functional annotation of these genes. Furthermore, machine learning algorithms were used to select hub DE-PCDRGs closely related to HIRI, and a robust risk assessment prediction model for HIRI was constructed. Additionally, using single-cell transcriptomic data, we further elucidated 19 diverse patterns of PCD in HIRI samples at the single-cell level and validated the hub DE-PCDRGs. Crucially, we mechanistically linked the THBS1-CD47 axis to apoptosis-exacerbated liver injury via *in vivo* and *in vitro* experiments.

**Results:**

Bulk transcriptomic analysis identified 25 DE-PCDRGs consistently upregulated in HIRI samples. Machine learning algorithms further screened 5 hub DE-PCDRGs (THBS1, MAP1LC3B, PPP1R15A, CXCL8, ZC3H12A), which formed a risk prediction model that effectively classified patients into high-risk and low-risk groups. These hub genes showed elevated expression in high-risk groups, accompanied by pronounced enrichment of 5 PCD patterns (anoikis, immunogenic cell death, NETosis, Netotic cell death, pyroptosis). Single-cell analysis further uncovered 12 distinct PCD patterns within the HIRI sample microenvironment, with spatial validation confirming the 5 hub DE-PCDRGs. The HIRI animal model confirmed the occurrence of apoptosis in liver tissue and upregulation of THBS1 in macrophages. Subsequent *in vitro* co-culture experiments demonstrated that macrophage-derived THBS1 directly engaged hepatocyte CD47, thereby suppressing the PI3K-AKT-NF-κB signaling pathway and promoting apoptosis.

**Conclusions:**

Our study delineated heterogeneous PCD patterns as pathological process of HIRI, demonstrating that the THBS1-CD47 axis drives macrophage-hepatocyte crosstalk to exacerbate apoptosis by inhibiting PI3K-AKT-NF-κB signaling. These results extend the current understanding of HIRI pathogenesis and nominate THBS1-CD47 as a promising candidate target.

## Introduction

Hepatic ischemia-reperfusion injury (HIRI), a sterile inflammatory response driven by innate immunity, critically contributes to postoperative liver failure. It comprises two subtypes: cold ischemia-reperfusion injury, driven by hepatic sinusoidal endothelial cell damage and microcirculatory disturbances during organ preservation and transplantation; and warm ischemia-reperfusion injury results from hepatocyte injury in partial hepatectomy, hemorrhagic shock, or traumatic events (1, 2). HIRI evolves through ischemic-phase metabolic disturbances (glycogen/ATP depletion and hypoxia-induced hepatocellular necrosis/apoptosis) and reperfusion-phase inflammatory cascades triggered by damage-associated molecular patterns (DAMPs) (3). Despite advancements in organ preservation strategies, HIRI-mediated allograft dysfunction continues to significantly impact early post-transplant outcomes (4, 5), highlighting the critical need for mechanism-based therapeutic interventions.

Programmed cell death (PCD), a critical process in cellular homeostasis, comprises diverse molecular pathways, including Apoptosis, Pyroptosis, Ferroptosis, Autophagy, Necroptosis, Cuproptosis, Parthanatos, Entotic cell death, Netotic cell death, Lysosome-dependent cell death, Alkaliptosis, Oxeiptosis, NETosis, Immunogenic cell death, Anoikis, Paraptosis, Methuosis, Entosis, and Disulfidptosis (6, 7). Extensive research has elucidated diverse patterns of PCD and their underlying regulatory mechanisms in HIRI. For instance, hepatocyte-specific GRINA overexpression mitigates HIRI-induced apoptosis by enhancing HRD1-mediated ATF6 ubiquitination (8). Silencing lncRNA KCNQ1OT1 promotes cell proliferation and reduces pyroptosis during HIRI by targeting the miR-142a-3p/HMGB1 axis (9). FTO deficiency in aged donor livers exacerbates ferroptosis during HIRI by upregulating ACSL4 and TFRC expression in an m6A-dependent manner (10). Depletion of the hepatocyte BMAL1/HSD17B13 axis inhibits lipid degradation by impairing hepatocyte autophagy, resulting in lipid overload and aggravated HIRI (11). However, existing studies have focused on individual PCD patterns, lacking a systematic characterization of the heterogeneity or integrative interplay among the 19 PCD patterns in HIRI.

Thrombospondin 1 (THBS1) interacts with multiple effector proteins, including various integrins (α6β1 or α4β1 integrins) and cell surface receptors (CD36 and CD47) (12). Current studies demonstrate that THBS1 in macrophage-derived exosomes directly binds to OTUD5 to promote GPX4 ubiquitination, inducing endothelial ferroptosis and exacerbating cerebral ischemia-reperfusion injury (13). Furthermore, the THBS1-CD47 axis mediates STAT3/Bcl2 signalling to trigger apoptosis in meningeal lymphatic endothelial cells, aggravating cerebral edema and neurological deficits during subarachnoid hemorrhage (14). CD47-reprogrammed exosomes mitigate HIRI by mimicking the “don’t eat me” mechanism to evade immune surveillance and counteract mitochondrial dysfunction (15). Although ferroptosis, apoptosis, and immune dysregulation have been implicated in HIRI, whether the THBS1-CD47 axis drives PCD or modulates macrophage-hepatocyte crosstalk within the hepatic immune microenvironment during HIRI remains unclear.

To address these gaps, this study performed an integrated transcriptomic dissection of PCD heterogeneity and diversity in HIRI. We integrated bulk and single-cell transcriptomics to analyse 19 PCD patterns during HIRI progression. Machine learning algorithms identified 5 hub DE-PCDRGs (THBS1, MAP1LC3B, PPP1R15A, CXCL8, ZC3H12A), which formed the basis for constructing a risk prediction model. Furthermore, *in vivo* and *in vitro* experiments demonstrated that macrophage-derived THBS1 binds to CD47 on the surface of hepatocytes, suppressing PI3K-AKT-NF-κB signalling to promote hepatocyte apoptosis and exacerbating HIRI. By systematically delineating the PCD landscape in HIRI and identifying the THBS1-CD47 axis as a previously unrecognized regulator of PCD-driven tissue injury, this study provides a preliminary framework for developing candidate targeted strategies to mitigate HIRI.

## Materials and methods

### Data source and preprocessing

We extracted gene expression profile data for HIRI and non-HIRI from the Gene Expression Omnibus (GEO) database, which includes four distinct datasets: GSE151648 RNA-seq data (23 pre-transplant samples and 23 post-reperfusion samples), GSE14951 (5 pre-transplant samples and 5 post-reperfusion samples) and GSE12720 (21 pre-transplant samples and 21 post-reperfusion samples) microarray data, and GSE171539 (1 pre-transplant sample and 1 post-reperfusion sample) single-cell sequencing data. The RNA-seq raw data were processed using scaling normalization, while the microarray raw data underwent background correction and quantile normalization. For all datasets, probe names were converted to gene symbols using the corresponding sequencing platform annotation files. Furthermore, quality control, identification of highly variable genes, batch integration, dimensionality reduction, clustering, and annotation of single-cell data were performed using the “Seurat” and “Harmony” R packages, with detailed data filtering criteria and processing workflows as described in previous studies (16). The current research included 19 categories of PCD sourced from prior literature (**Supplementary Table 1**), identifying a total of 1964 PCD-related genes (17).

### Exploration of differentially expressed PCDRGs

We utilized the “limma” R package to compare DEGs between pre-transplant (Pre) samples and post-reperfusion (Post) samples across the datasets GSE151648, GSE14951, and GSE12720. The criteria were set to |log2(FC)| > 1 and adjusted p-value < 0.05, leading to the DE-PCDRGs through intersection with PCDRGs. Furthermore, we conducted Gene Ontology (GO) enrichment analysis and Kyoto Encyclopedia of Genes and Genomes (KEGG) pathway analysis on the DE-PCDRGs using the “clusterProfiler” R package.

### Establishment of HIRI prediction model and selection of hub DE-PCDRGs

After designating GSE151648 as the training set and GSE14951, GSE12720 as the testing sets, we constructed a HIRI risk assessment model using 12 machine learning algorithms (RF, Enet, Lasso, Ridge, SVM, plsRglm, Stepglm, glmBoost, GBM, LDA, XGBoost, and NaiveBayes) within a leave-one-out cross-validation (LOOCV) framework, employing 101 algorithm combinations. Among the models with similar areas under the ROC curve (AUC), the glmBoost + Lasso algorithm incorporated the fewest DE-PCDRGs. Based on the HIRI prediction model constructed with glmBoost + Lasso algorithm, we fitted a logistic regression with the “rms” R package, and performed calibration analysis via bootstrap (B = 1000). Subsequently, we performed HIRI risk assessment on samples from the GSE151648 dataset based on the coefficient values and expression levels of DE-PCDRGs identified by the glmBoost + Lasso algorithm, categorizing the samples into high-risk and low-risk groups according to the median risk value. Additionally, gene set enrichment analysis (GSEA) was conducted on samples from both the high-risk and low-risk groups using the “clusterProfiler” R package, with pathway activity assessed through normalized enrichment scores (NES).

### Investigation of immune cell infiltration and PCD

The CIBERSORT method, a robust computational algorithm, was employed to assess the immune cell infiltration in samples from high-risk and low-risk groups. Concurrently, we conducted a Spearman correlation analysis to evaluate the relationship between the expression levels of hub DE-PCDRGs and the characteristics of immune cells. Furthermore, Spearman correlation analysis was utilized to further validate the associations between various types of PCD and hub DE-PCDRGs.

### Gene set variation analysis for PCD

Based on the 19 categories of PCD-related gene sets included in our study, we employed the GSVA algorithm to compute a comprehensive score for each gene set, thereby assessing the level of variation in various PCD pathways across different samples. We compared the GSVA enrichment scores of the PCD pathways between high-risk and low-risk groups, with a significance threshold set at P<0.05 for determining differences between the two groups.

### Single-cell sequencing data analysis

Utilizing the “FindAllMarkers” function (test. use = “wilcox”) of the “Seurat” R package, we explored the marker genes of various cell clusters. Furthermore, we employed the “FindMarkers” function (test. use = “wilcox”) to identify DEGs between Pre samples and Post samples across the distinct cell clusters. We conducted KEGG pathway analysis on the DEGs of each cell cluster through the “clusterProfiler” R package. The “UCell” and “ggplot2” (stat_compare_means (method = “t.test”) R packages were applied to compare the activity of 19 categories of PCD across the two groups. Moreover, we employed the “effsize” R package to calculate the effect size of the statistically significant differences in PCD UCell scores between Pre samples and Post samples. Finally, we extracted single-cell data from Pre samples and Post samples to create CellChat objects, allowing us to compare the communication networks, enriched signalling pathways, and ligand-receptor interactions among the cell clusters between the two groups.

### Animals and HIRI model

C57BL6/J mice aged 6–8 weeks, weighing between 20-23g, were obtained from Spf Biotech Co. Ltd (Beijing, China). After one-week acclimatization period, the mice were randomly divided into two groups (n=6 per group): the Sham group and the HIRI group. The HIRI model was established by intraperitoneally anesthetizing the mice with tribromoethanol, following previous literature to create a 70% liver ischemia model (18). Briefly, a non-invasive vascular clamp was used to occlude blood flow to the left and middle lobes of the liver via the portal vein and hepatic artery for 60 minutes (19, 20). After a 6-hour reperfusion period, serum and liver tissue were collected for subsequent analysis. Mice in the Sham group underwent laparotomy and closure without occlusion of the portal vein and hepatic artery.

### Liver function assessment and haematoxylin-eosin staining

To assess the variations in serum ALT and AST levels among the groups, we conducted biochemical analyses following the manufacturer’s protocol (Servicebio, Wuhan, China). Liver samples were fixed in 4% paraformaldehyde, embedded in paraffin, and sectioned into 5 µm slices, which were subsequently stained with HE staining.

### TUNEL staining

Apoptosis in tissue sections was assessed via the terminal deoxynucleotidyl transferase dUTP nick end labeling (TUNEL) assay, according to the manufacturer’s instructions (Roche Diagnostics, USA). Briefly, the TUNEL reaction mixture, comprising enzyme and label solution, was applied to tissue sections and incubated at 37 °C in the dark for 90 minutes. Following two washes with phosphate-buffered saline (PBS), sections were counterstained with DAPI at room temperature for 1 minute and subsequently visualized using a fluorescence microscope (Nikon Eclipse Ti2, Japan).

### Immunohistochemistry and immunofluorescence staining

Following overnight incubation of liver tissue sections with primary antibody (anti-THBS1, 67241-1-Ig, Proteintech, China) at 4 °C, sections were incubated with biotinylated anti-rabbit IgG secondary antibody for 2 hours. Subsequently, sections were developed using diaminobenzidine (DAB) chromogen and counterstained with hematoxylin to visualize cell nuclei. Using mouse THBS1 monoclonal antibody and rabbit F4/80 polyclonal antibody, we analyzed the IF staining of THBS1 in macrophages within liver sections. Additionally, we employed rabbit Bax polyclonal antibody, mouse Bcl2 monoclonal antibody, and rabbit Cleaved-caspase 3 polyclonal antibody to detect the expression of apoptosis-related markers in liver sections through IF staining. The secondary antibodies used were CoraLite488-conjugated Goat Anti-Rabbit IgG (H+L) and CoraLite594-conjugated Goat Anti-Mouse IgG (H+L). All antibodies were sourced from Proteintech (Wuhan, China). Furthermore, we captured IF staining images using a fluorescence microscope (Nikon Eclipse Ti2, Japan).

### Enzyme-linked immunosorbent assay

Following the manufacturer’s protocol (Thermo Fisher Scientific, USA), cytokine secretion levels in serum or cell supernatants were determined using enzyme-linked immunosorbent assay.

### Oxygen-glucose deprivation/reperfusion model

AML12 and RAW264.7 (Procell, Wuhan, China) were cultured according to the manufacturer’s instructions. To simulate the microenvironment of HIRI (21), we placed transwell inserts (Corning, USA) containing AML12 cells into a 6-well plate initially seeded with RAW264.7 cells. The culture medium was then replaced with glucose-free, serum-free DMEM, and the cells were incubated under hypoxic conditions for 6 hours (1% O2, 5% CO2, and 94% N2), after which the co-cultured cells were transferred to a normoxic incubator for additional 3 hours.

### Cell treatment

RAW264.7 cells were pre-transfected *in vitro* with CALNP™ RNAi (D-Nano Therapeutics, Beijing, China) targeting siRNA-THBS1 (siRNA sense sequence as follow: 5’-GCTGGAAAGATTTCACTGCAUTT-3’) or siRNA-Negative Control (siNC). AML12 cells were pre-transfected *in vitro* with CALNP™ RNAi targeting siRNA-CD47 (siRNA sense sequence as follow: 5’-GCAGAACTACTTGGATTAGTUTT-3’) or siRNA-Negative Control (siNC). Following transfection, both RAW264.7 and AML12 cells underwent OGD/R stimulation. Furthermore, AML12 cells were seeded in 6-well plates 24 hours prior to the establishment of the OGD/R model, with each well receiving 1 μg/mL of recombinant mouse THBS1 protein (MCE, Shanghai, China).

### Quantitative real-time PCR

Total RNA was reverse transcribed into cDNA using HiScript III RT SuperMix (Vazyme, Nanjing, China) for qPCR reagent containing gDNA wiper. The expression of the target gene was detected by real-time quantitative PCR using ChamQ Universal SYBR qPCR Master Mix (Vazyme, Nanjing, China). Primers used for PCR were as follows: THBS1-F, 5’-GAACGGGAAGCCCTGTGAAG-3’; THBS1-R, 5’-GTTACAGAGTCGGCTGCGTC-3’; CD47-F, 5’-TGGTGGGAAACTACACTTGCG-3’; CD47-R, 5’-GAAAACCACGAAACCGTGCG-3’; ACTB-F, 5’-GGCTGTATTCCCCTCCATCG-3’; ACTB-R, 5’-CCAGTTGGTAACAATGCCATGT-3’.

### Western blotting

Protein extraction from tissues and cells was performed utilizing RIPA lysis buffer, supplemented with protease and phosphatase inhibitors (Beyotime, Shanghai, China). The extracted proteins underwent SDS-PAGE electrophoresis, followed by transfer onto polyvinylidene difluoride (PVDF) membranes. Primary antibodies targeting THBS1, Bax, Bcl2, Cleaved-caspase 3, CD47, p-AKT, AKT, p-NF-kB p65, NF-kB p65, and β-actin (Proteintech, Wuhan, China) were employed, with overnight incubation at 4 °C. Following incubation with the appropriate HRP-conjugated secondary antibodies at room temperature for 2 hours, images were acquired using a Biorad chemiluminescence imaging system.

### Hoechst 33342/propidium iodide dual staining assay

Apoptosis was assessed utilizing an apoptosis/necrosis detection kit (Solarbio, Beijing, China). Following OGD/R treatment, cells were washed three times with phosphate-buffered saline (PBS). Subsequently, cells were incubated in a medium containing propidium iodide (PI) and Hoechst 33342 at 4 °C for 30 minutes. After two additional washes, cells were imaged using fluorescence microscopy.

### Mitochondrial membrane potential assay (ΔψM)

Mitochondrial membrane potential was assessed utilizing the JC-1 Mitochondrial Membrane Potential Assay Kit (Beyotime, Shanghai, China). Following the OGD/R procedure, cells were incubated with JC-1 staining solution at 37 °C for 20 min. Following washing steps, images were captured and observed under a fluorescence microscope.

### Statistical analysis

Data were presented as mean ± standard deviation. Statistical analyses were performed utilizing one-way ANOVA and Student’s t-tests, with statistical significance denoted as *P < 0.05, **P < 0.01, and ***P < 0.001.

## Results

### Identification and functional enrichment analysis of DE-PCDRGs

**Figure 1:**
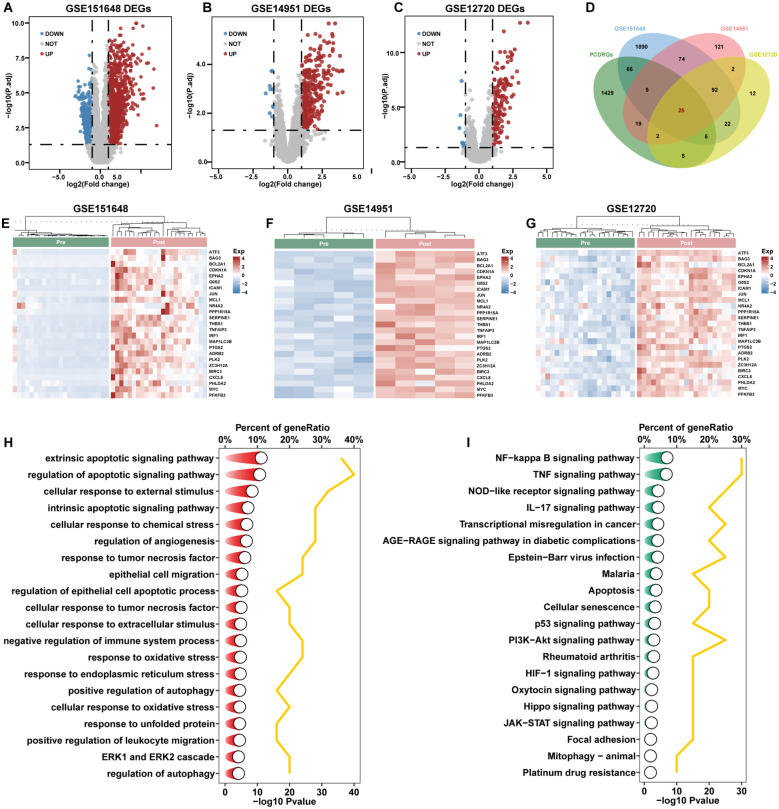
Identification and functional enrichment analysis of DE-PCDRGs. **(A)** Volcano plot of DEGs from GSE151648. **(B)** Volcano plot of DEGs from GSE14951. **(C)** Volcano plot of DEGs from GSE12720. **(D)** Intersection of DEGs and PCDRGs. **(E)** Heatmap of 25 DE-PCDRGs expression in GSE151648. **(F)** Heatmap of 25 DE-PCDRGs expression in GSE14951. **(G)** Heatmap of 25 DE-PCDRGs expression in GSE12720. **(H)** GO enrichment analysis of 25 DE-PCDRGs in biological processes. **(I)** KEGG enrichment analysis of 25 DE-PCDRGs.

Differential expression analysis of the GSE151648, GSE14951, and GSE12720 datasets identified 2183 DEGs (1266 upregulated and 917 downregulated genes; **Figure 1A**, **Supplementary Table 2**), 344 DEGs (334 upregulated and 10 downregulated genes; **Figure 1B**, **Supplementary Table 3**), and 165 DEGs (154 upregulated and 11 downregulated genes; **Figure 1C**, **Supplementary Table 4**), respectively. Following the intersection of these DEGs with 1560 PCDRGs, 25 DE-PCDRGs were identified (**Figure 1D**, **Supplementary Table 5**). Concurrently, the 25 DE-PCDRGs exhibited consistent upregulation in post-reperfusion samples across the three datasets (**Figures 1E–G**). To further elucidate the specific PCD types mediated by the DE-PCDRGs during HIRI, along with their broader biological functions and related signaling pathways, functional enrichment analysis revealed that DE-PCDRGs were associated with apoptosis (extrinsic apoptotic signaling pathway, regulation of apoptotic signaling pathway, intrinsic apoptotic signaling pathway), autophagy (positive regulation of autophagy, regulation of autophagy), oxidative stress (response to oxidative stress, response to endoplasmic reticulum stress, response to unfolded protein), and other biological processes (**Figure 1H**). Furthermore, KEGG analysis indicated that DE-PCDRGs were involved in the regulation of apoptotic, mitophagy, NF-kB, TNF, NOD-like, IL17, and PI3K-AKT signaling pathways (**Figure 1I**).

### Development of the HIRI prediction model and selection of hub DE-PCDRGs

**Figure 2:**
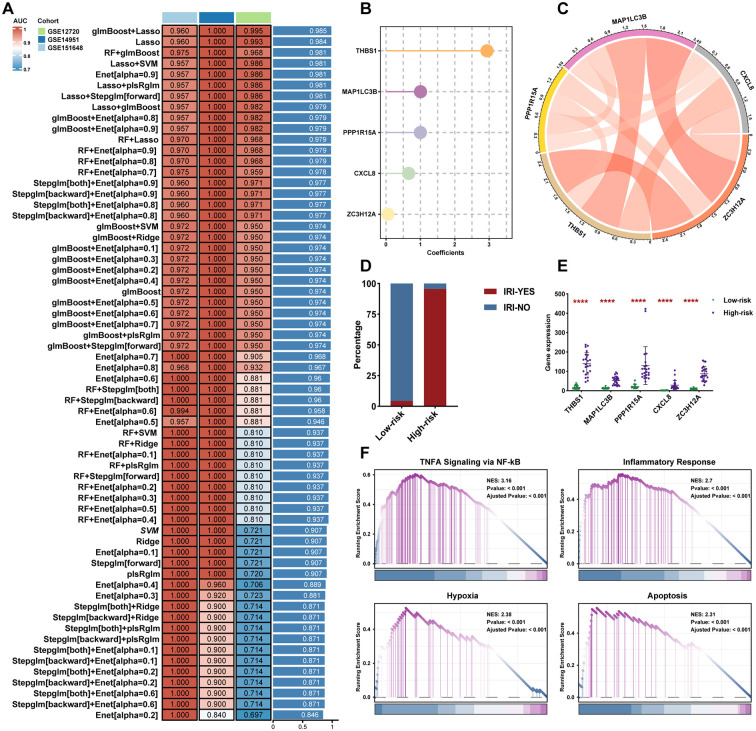
Development of the HIRI prediction model. **(A)** AUC values for each model across 101 algorithms in three datasets. **(B)** Regression coefficients for the 5 hub DE-PCDRGs. **(C)** 5 hub DE-PCDRGs expression correlation chord diagram. **(D)** Histogram of HIRI sample proportion in high-risk and low-risk groups. **(E)** Violin plot of the 5 hub DE-PCDRGs expression between high-risk and low-risk groups. **(F)** Gene set enrichment analysis between high-risk and low-risk groups.

As previously described, we identified 25 DE-PCDRGs in HIRI samples, which exhibited differential expression across liver tissues and influenced HIRI risk prediction. To construct a robust predictive model and identify candidate hub DE-PCDRGs, we employed a LOOCV framework, evaluating 101 algorithm combinations across the GSE151648, GSE14951, and GSE12720 datasets. Average AUC values were calculated for each algorithm (**Supplementary Table 6**), and the glmBoost+Lasso algorithm was selected for its optimal performance and minimal gene inclusion (**Figure 2A**). Within the glmBoost+Lasso algorithm, the model incorporated THBS1, MAP1LC3B, PPP1R15A, CXCL8, and ZC3H12A as hub DE-PCDRGs, with THBS1 exhibiting the highest and ZC3H12A the lowest regression coefficients (**Figure 2B**). To address potential overfitting or instability due to small sample size, we employed bootstrap methods (B = 1000) to evaluate model calibration. Results indicated that (**Supplementary Figure 1**, **S2**), in both training and testing sets, the calibration curves closely align with the ideal line, suggesting the model exhibits robust stability. Furthermore, we analyzed the expression correlation among hub DE-PCDRGs, revealing strong correlation among the three genes, excluding CXCL8 and PPP1R15A (**Figure 2C**). Subsequently, we calculated the HIRI risk for each sample across the three datasets and categorized all samples into high-risk and low-risk groups based on the median risk score. The high-risk group demonstrated a significantly higher proportion of HIRI cases compared to the low-risk group (**Figure 2D**), with hub DE-PCDRGs markedly upregulated in high-risk samples (**Figure 2E**). To assess potential biological differences between the high-risk and low-risk groups, we performed GSEA analysis, which indicated a significant enrichment of hypoxia, apoptosis, TNFA signaling via NF-KB, and inflammatory response in the high-risk group (**Figure 2F**).

### Analysis of immune infiltration and cell death in high-risk and low-risk groups

**Figure 3:**
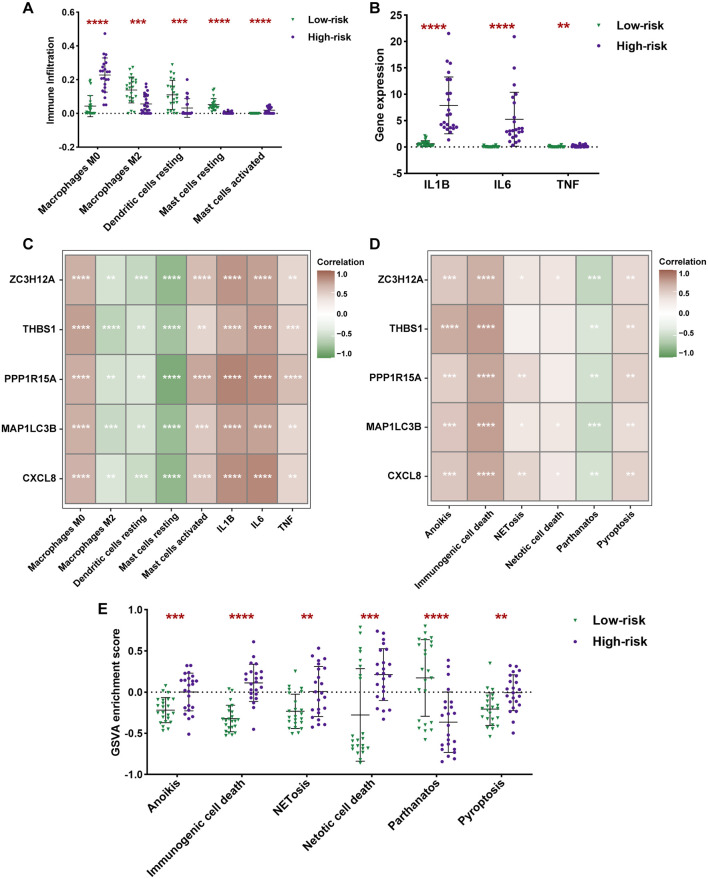
Analysis of immune infiltration and cell death in high-risk and low-risk groups. **(A)** Comparison of immune infiltration between high-risk and low-risk groups. **(B)** Violin plot of pro-inflammatory cytokine expression between high-risk and low-risk groups. **(C)** Heatmap of the correlation between 5 hub DE-PCDRGs and immune cells and cytokines. **(D)** Heatmap of the correlation between 5 hub DE-PCDRGs and PCD. **(E)** Violin plot of PCD enrichment score between high-risk and low-risk groups.

GSEA revealed significant activation of inflammatory response and apoptosis signaling pathways in the high-risk group. To further characterize immune microenvironment and cell death differences between risk groups, we conducted immune infiltration and cell death analyses. CIBERSORT algorithm analysis demonstrated significant disparities in five immune cell types: Macrophages M0, Macrophages M2, Dendritic cells resting, Mast cells resting, and Mast cells activated (**Figure 3A**). Given the anti-inflammatory and reparative role of Macrophages M2, whose infiltration was reduced in the high-risk group, we further compared cytokine expression levels between groups. As shown in **Figure 3B**, high-risk group samples exhibited elevated expression of IL1B, IL6, and TNF. These findings collectively suggest that immune responses are involved in the occurrence and progression of HIRI. Furthermore, we found that hub DE-PCDRGs were highly correlated with immune responses. These hub DE-PCDRGs were positively correlated with Macrophages M0, Mast cells activated, IL1B, IL6, and TNF, while negatively correlated with Macrophages M2, Dendritic cells resting, and Mast cells resting (**Figure 3C**). Subsequently, we compared the activation status of PCD pathways between the high-risk and low-risk groups. The results indicated significant activation of Anoikis, Immunogenic cell death, NETosis, Netotic cell death, and Pyroptosis in the high-risk group, which were positively correlated with hub DE-PCDRGs, whereas Parthanatos was suppressed and negatively correlated with hub DE-PCDRGs (**Figures 3D, E**).

### Differential expression and functional enrichment analysis at single-cell level

**Figure 4:**
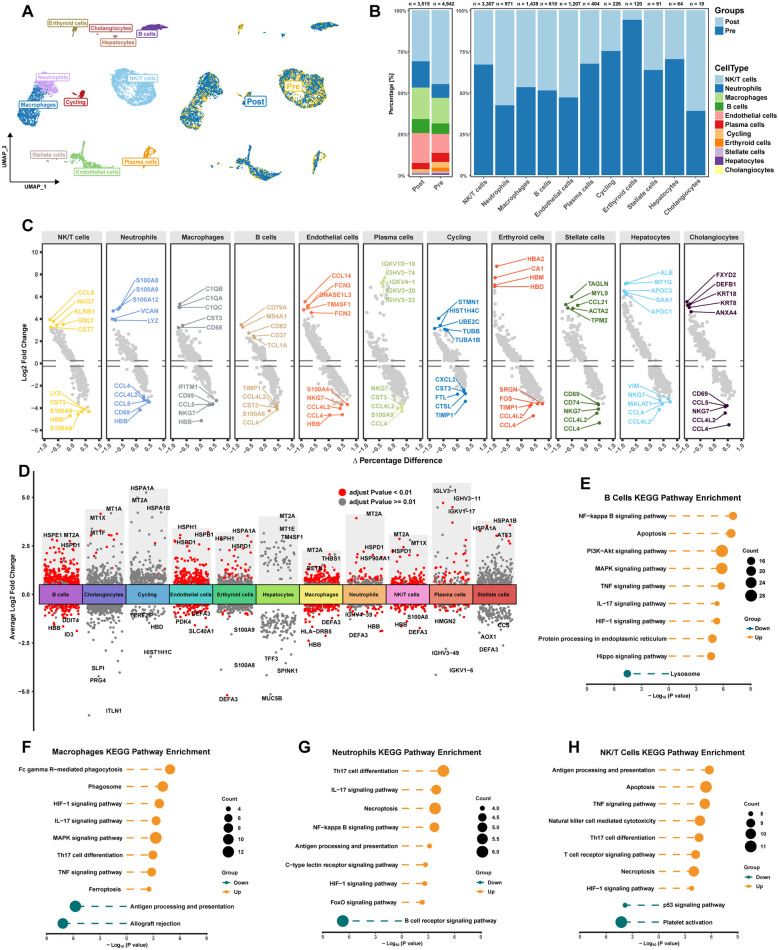
Differential expression and functional enrichment analysis at single-cell level. **(A)** UMAP plot of single-cell sequencing analysis. Distinct colors denoted different groups and cells. **(B)** Histogram illustrated the proportions of various cell types in Pre and Post samples. **(C)** Top 10 marker genes in each cell type. **(D)** DEGs of various cell types between Pre sample and Post sample. **(E)** KEGG enrichment analysis of DEGs in B cells. **(F)** KEGG enrichment analysis of DEGs in Macrophages. **(G)** KEGG enrichment analysis of DEGs in Neutrophils. **(H)** KEGG enrichment analysis of DEGs in NK/T cells.

To further characterize immune cells involved in HIRI, we analyzed single-cell data from pre-transplant and post-reperfusion samples obtained from public databases. Following annotation, 12 distinct cell types were identified, including hepatocytes and non-parenchymal cells (**Figure 4A**). The distribution of these cell types in pre-transplant and post-reperfusion samples is presented in **Figure 4B**. **Figure 4C** illustrates the expression of the top 10 marker genes within each cell cluster. Subsequently, we identified differentially expressed genes (DEGs) within the cell clusters between pre-transplant and post-reperfusion samples, revealing upregulated THBS1 expression in macrophages (**Figure 4D**). Furthermore, KEGG enrichment analysis indicated the activation of distinct PCD patterns in various immune cells following reperfusion. Specifically, apoptosis was observed in B cells (**Figure 4E**), ferroptosis in macrophages (**Figure 4F**), necroptosis in neutrophils (**Figure 4G**), and both apoptosis and necroptosis in NK/T cells (**Figure 4H**).

### PCD score based on single-cell data

**Figure 5:**
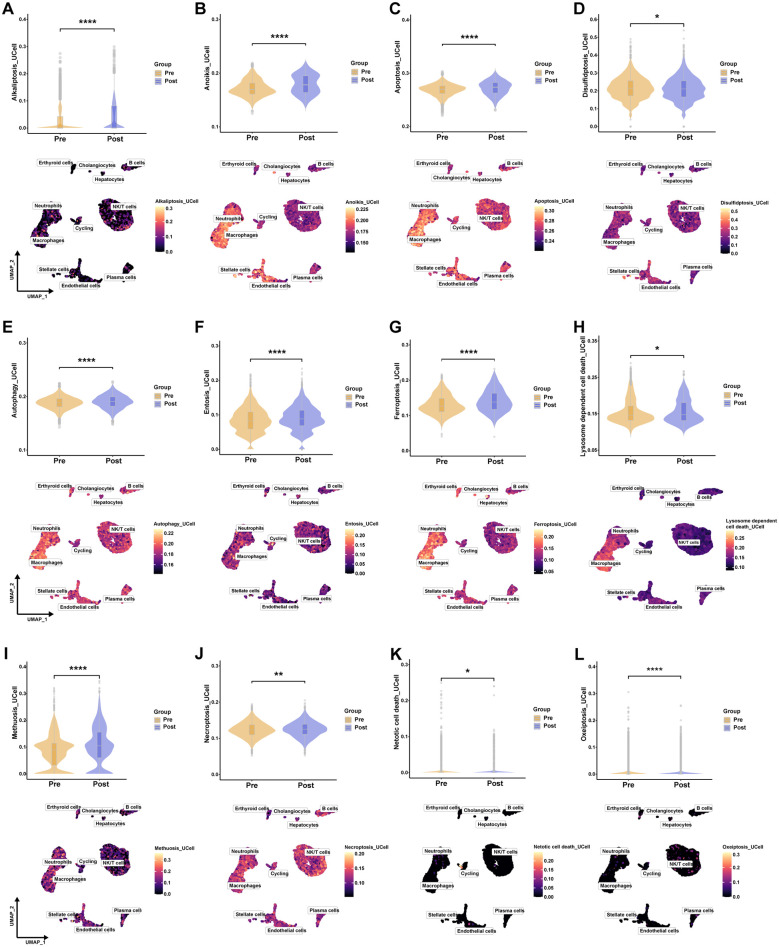
PCD score based on single-cell data. **(A–L)** Violin plots and UMAP plots compared Alkaliptosis_UCell, Anoikis_UCell, Apoptosis_UCell, Disulfidptosis_UCell, Autophagy_UCell, Entosis_UCell, Ferroptosis_UCell, Lysosome-dependent cell death_UCell, Methuosis_UCell, Necroptosis_UCell, Netotic cell death_UCell and Oxeiptosis_UCell scores between Pre sample and Post sample.

In prior analyses, we identified distinct PCD patterns across various immune cells. To comprehensively characterize the PCD landscape in HIRI, we employed the “UCell” algorithm to calculate scores for 19 PCD pathways in each cell. The results revealed a significant enrichment of 12 PCD pathways in post-reperfusion samples compared to pre-transplant samples. These pathways included Alkaliptosis (**Figure 5A**, Cohen’s d = 0.367, 95% CI [0.324, 0.411]), Anoikis (**Figure 5B**, Cohen’s d = 0.459, 95% CI [0.416, 0.503]), Apoptosis (**Figure 5C**, Cohen’s d = 0.417, 95% CI [0.374, 0.461]), Disulfidptosis (**Figure 5D**, Cohen’s d = -0.045, 95% CI [-0.088, -0.002]), Autophagy (**Figure 5E**, Cohen’s d = 0.18, 95% CI [0.137, 0.224]), Entosis (**Figure 5F**, Cohen’s d = 0.165, 95% CI [0.121, 0.208]), Ferroptosis (**Figure 5G**, Cohen’s d = 0.383, 95% CI [0.339, 0.427]), Lysosome-dependent cell death (**Figure 5H**, Cohen’s d = 0.045, 95% CI [0.002, 0.089]), Methuosis (**Figure 5I**, Cohen’s d = 0.335, 95% CI [0.291, 0.378]), Necroptosis (**Figure 5J**, Cohen’s d = 0.059, 95% CI [0.016, 0.102]), Netotic cell death (**Figure 5K**, Cohen’s d = -0.052, 95% CI [-0.095, -0.009]), and Oxeiptosis (**Figure 5L**, Cohen’s d = -0.153, 95% CI [-0.197, -0.11]). Specifically, among the PCD pathways examined, Anoikis (Cohen’s d = 0.459) and Apoptosis (Cohen’s d = 0.417) exhibited the most substantial increases in activity, with effect sizes approaching the medium threshold. This provides strong, quantitative evidence for the targeted and specific activation of these PCD patterns in HIRI. Furthermore, we observed the co-occurrence of multiple PCD patterns in neutrophils and macrophages, specifically Anoikis (**Figure 5B**), Apoptosis (**Figure 5C**), Autophagy (**Figure 5E**), Ferroptosis (**Figure 5G**), Lysosome-dependent cell death (**Figure 5H**), and Necroptosis (**Figure 5J**).

### Analysis of hub DE-PCDRGs expression and cellular communication

**Figure 6:**
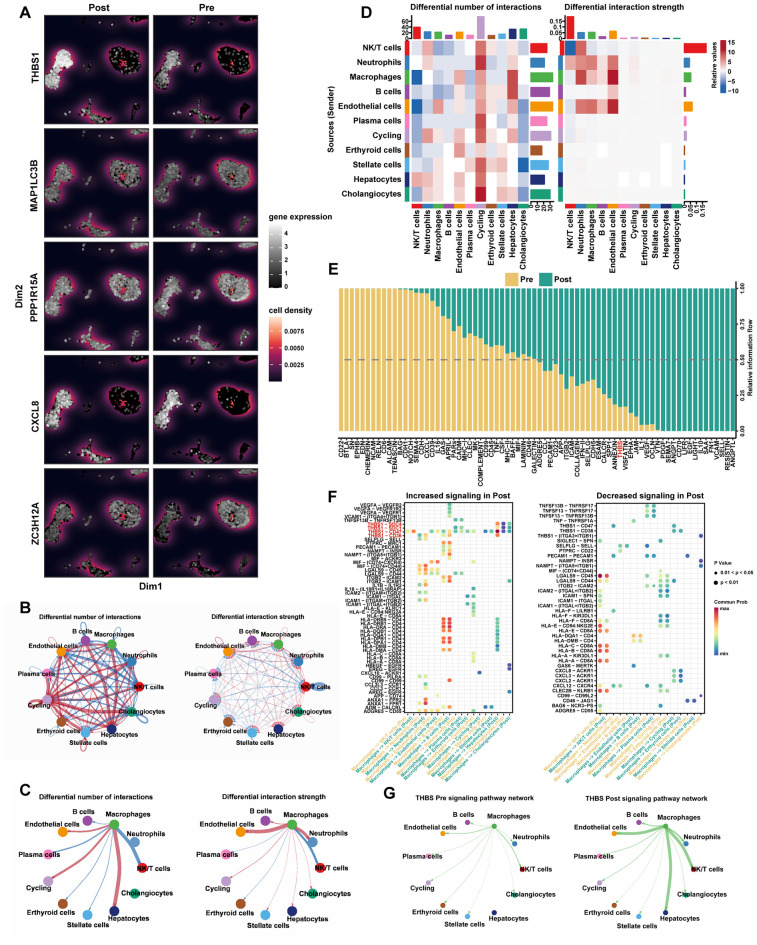
Analysis of hub DE-PCDRGs expression and cellular communication. **(A)** UMAP plots of 5 hub DE-PCDRGs expression distribution in the Pre and Post samples. **(B)** Comparison of cellular interaction quantity and strength between Pre sample and Post sample. **(C)** Comparison of cellular interaction quantity and strength between macrophages and other cell types in Pre and Post samples. **(D)** A comparison heat map of cellular interaction quantity and strength between Pre sample and Post sample. **(E)** Comparison of the relative information flow of each signaling pathway between Pre sample and Post sample. **(F)** Compared with Pre sample, the increased and decreased signaling of intercellular ligand-receptor pairs in Post sample. **(G)** The interaction network diagrams of THBS signaling pathway between macrophages and other cell types in Pre and Post samples.

As previously mentioned, five hub DE-PCDRGs were identified. However, the expression distributions of these genes remained unclear. Therefore, we visualized these genes using single-cell data. As shown in **Figure 6A**, hub DE-PCDRGs were expressed across multiple cell types in both pre-transplant and post-reperfusion samples. Consistent with prior findings, THBS1 expression was markedly elevated in macrophages from post-reperfusion samples. Subsequently, we compared cell-cell communication between pre-transplant and post-reperfusion samples. **Figures 6B, C**, and 6D illustrate the differences in the frequency and strength of cell-cell interactions between the two groups. Compared to pre-transplant samples, the frequency and strength of interactions between macrophages and hepatocytes, endothelial cells, and cycling increased in post-reperfusion samples. Several ligand-receptor-mediated cell interactions were significantly enhanced in post-reperfusion samples, including THBS1-mediated intercellular signaling (**Figure 6E**). We further analyzed the specific pathways of interactions among various cell types in post-reperfusion samples (**Figure 6F**). The results demonstrated that, compared to pre-transplant samples, macrophages delivered stronger signals to hepatocytes in THBS1-related pathways, with the THBS1-CD47 axis emerging as the dominant pathway. **Figure 6G** further supports the enhancement of THBS1-related pathways in post-reperfusion samples.

### THBS1 upregulation in macrophages during HIRI

**Figure 7:**
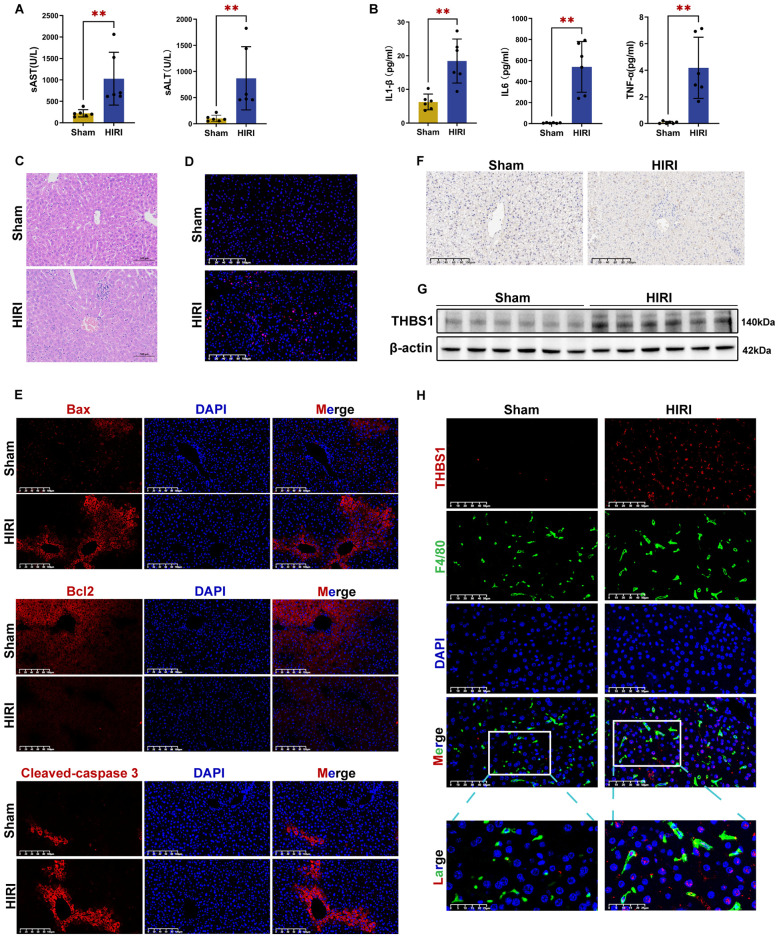
THBS1 upregulation in macrophages during HIRI. **(A)** sALT and sAST levels in Sham and HIRI groups. **(B)** Serum IL-1β, IL-6, and TNF-α levels in Sham and HIRI groups. **(C)** HE staining of liver tissue from the Sham and HIRI groups. Scale bar = 100μm. **(D)** TUNEL staining of liver tissue from the Sham and HIRI groups. **(E)** Immunofluorescence staining showed the expressions of pro-apoptotic proteins Bax, Cleaved-caspase 3, and anti-apoptotic Bcl2 protein in liver tissues between the Sham and HIRI groups. **(F)** Immunohistochemical staining of THBS1 protein in liver tissue from the Sham and HIRI groups. **(G)** THBS1 protein expression in liver tissues of the Sham and HIRI groups. **(H)** Immunofluorescence double staining revealed THBS1 expression on F4/80-labeled macrophages between the Sham and HIRI groups. HIRI, hepatic ischemia-reperfusion injury.

Previously, single-cell sequencing data revealed increased THBS1 expression in macrophages within post-reperfusion liver tissues. Subsequently, a murine HIRI model was successfully established. As shown in **Figures 7A, B**, serum alanine aminotransferase (sALT), aspartate aminotransferase (sAST), and pro-inflammatory cytokine (IL-1β, IL-6, TNF-α) levels were significantly elevated in the HIRI group compared to the Sham group. HE staining demonstrated hepatocellular necrosis and inflammatory cell infiltration in the HIRI group (**Figure 7C**). TUNEL staining and immunofluorescence assays further revealed increased apoptosis, elevated expression of pro-apoptotic proteins Bax and Cleaved-caspase 3, and reduced anti-apoptotic Bcl2 expression in the HIRI group (**Figures 7D, E**, **Supplementary Figures 3**, **S4**). These findings collectively indicate that HIRI induces liver dysfunction, tissue inflammation, and apoptosis in mice. Furthermore, immunohistochemical staining identified upregulated THBS1 expression in hepatic sinusoids following HIRI (**Figure 7F**). Western blot analysis confirmed increased THBS1 protein levels in the HIRI group (**Figure 7G**). Finally, immunofluorescence double staining demonstrated enhanced THBS1 expression on F4/80-labeled macrophages in the HIRI group compared to the Sham group (**Figure 7H**, **Supplementary Figure 5**).

### OGD/R model induced THBS1 expression in RAW264.7 cells and apoptosis in AML12 cells

**Figure 8:**
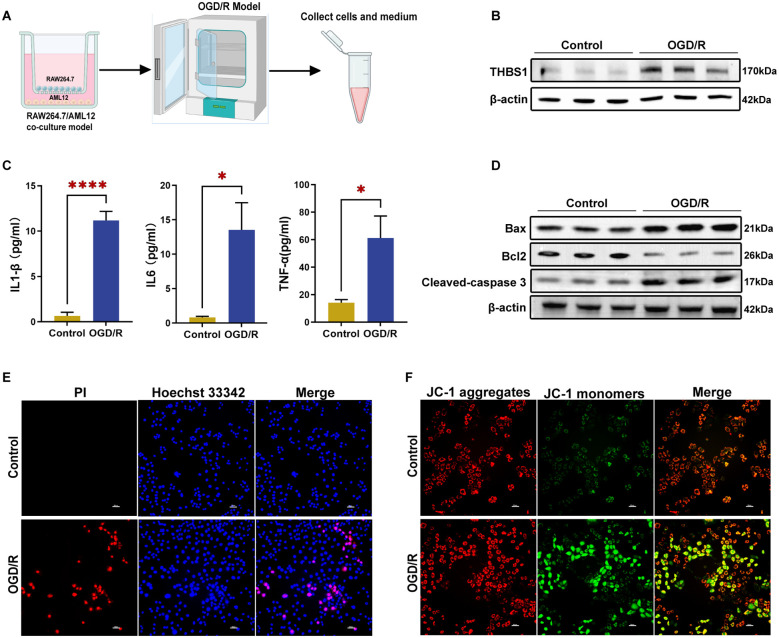
OGD/R model induced THBS1 expression in RAW264.7 cells and apoptosis in AML12 cells. **(A)** Schematic of OGD/R treatment. AML12 cells (lower chamber) and RAW264.7 cells (upper chamber) were co-cultured in serum-free, glucose-free DMEM under hypoxic conditions (1% O2) for 6 hours, followed by re-oxygenation in complete medium for another 3 hours. Control cells were cultured in complete medium under normoxic conditions. **(B)** THBS1 protein expression in RAW264.7 cells between Control and OGD/R groups. **(C)** The contents of IL-1β, IL-6, and TNF-α in RAW264.7 cell culture medium between Control and OGD/R groups. **(D)** The expressions of pro-apoptotic proteins Bax, Cleaved-caspase 3, and anti-apoptotic Bcl2 protein in AML12 cells between Control and OGD/R groups. **(E)** Cell death in the Control and OGD/R groups was assessed using Hoechst 33342 and PI double staining. Scale bar = 50μm. **(F)** The mitochondrial membrane potential (ΔψM) of the Control and OGD/R groups was detected using JC-1 staining. Scale bar = 50μm.

To simulate the *in vivo* microenvironment, we established a cell co-culture model using transwell chambers, followed by OGD/R modeling (**Figure 8A**). OGD/R treatment significantly increased THBS1 protein expression in RAW264.7 cells (**Figure 8B**), consistent with previous experimental findings. Meanwhile, the secretion of pro-inflammatory cytokines (IL-1β, IL-6, TNF-α) by RAW264.7 cells was also significantly elevated (**Figure 8C**). Furthermore, OGD/R-exposed AML12 cells exhibited upregulated expression of pro-apoptotic proteins Bax and Cleaved-caspase 3, alongside reduced anti-apoptotic Bcl2 levels (**Figure 8D**). Hoechst 33342-PI double staining demonstrated increased AML12 cell death following OGD/R (**Figure 8E**, **Supplementary Figure 6**). Finally, JC-1 staining revealed a significant decline in mitochondrial membrane potential (ΔψM) in AML12 cells after OGD/R (**Figure 8F**, **Supplementary Figure 7**). These findings preliminarily validate that the OGD/R model can induce elevated THBS1 expression in macrophages and hepatocyte apoptosis.

### RAW264.7-specific THBS1 siRNA treatment alleviated OGD/R-induced apoptosis in AML12 cells

**Figure 9:**
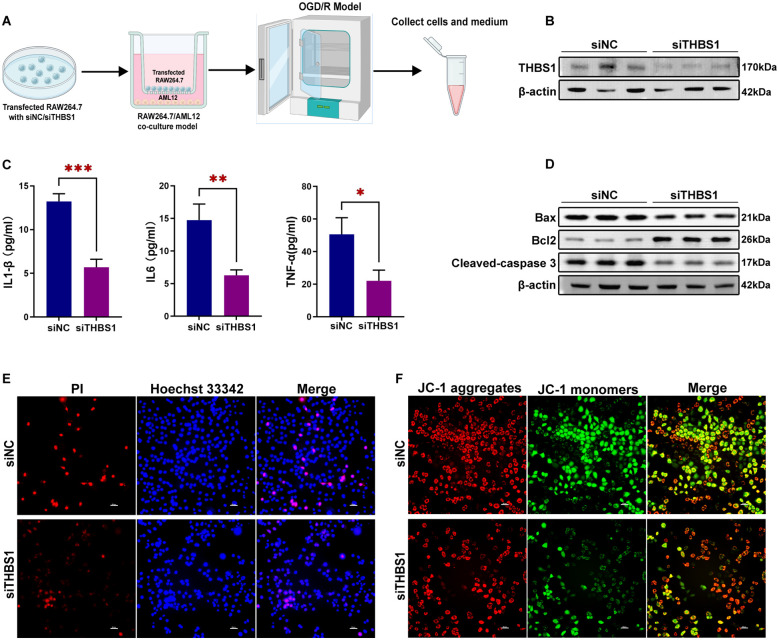
RAW264.7-specific THBS1 siRNA treatment alleviated OGD/R-induced apoptosis in AML12 cells. **(A)** Schematic of OGD/R treatment. RAW264.7 cells, separately transfected with siRNA-NC and siRNA-THBS1, were seeded in the upper chamber and co-cultured with AML12 cells (lower chamber) under hypoxic conditions (1% O2) in serum-free, glucose-free DMEM for 6 hours, followed by reoxygenation in complete medium for another 3 hours. **(B)** THBS1 protein expression in RAW264.7 cells between siRNA-NC and siRNA-THBS1 groups. **(C)** The contents of IL-1β, IL-6, and TNF-α in RAW264.7 cell culture medium between siRNA-NC and siRNA-THBS1 groups. **(D)** The expression of pro-apoptotic proteins Bax, Cleaved-caspase 3, and anti-apoptotic Bcl2 protein in AML12 cells between siRNA-NC and siRNA-THBS1 groups. **(E)** Cell death in the siRNA-NC and siRNA-THBS1 groups was assessed using Hoechst 33342 and PI double staining. Scale bar = 50μm. **(F)** The mitochondrial membrane potential (ΔψM) of the siRNA-NC and siRNA-THBS1 groups was detected using JC-1 staining. Scale bar = 50μm.

To investigate the impact of macrophage-derived THBS1 on AML12 apoptosis, RAW264.7 cells were transfected with siRNA targeting THBS1 to assess its role in OGD/R-induced AML12 cells (**Figure 9A**). siRNA treatment significantly reduced THBS1 mRNA and protein expression in RAW264.7 cells (**Supplementary Figure 8**, **Figure 9B**). Furthermore, THBS1 knockdown markedly decreased the secretion of pro-inflammatory cytokines (IL-1β, IL-6, TNF-α) by RAW264.7 cells (**Figure 9C**). Macrophage-specific THBS1 knockdown attenuated OGD/R-induced upregulation of pro-apoptotic proteins Bax and Cleaved-caspase 3, while increasing anti-apoptotic Bcl2 expression in AML12 cells (**Figure 9D**). Concurrently, THBS1 knockdown in RAW264.7 cells reduced AML12 cell death (**Figure 9E**, **Supplementary Figure 9**) and restored mitochondrial membrane potential (ΔψM) (**Figure 9F**, **Supplementary Figure 10**). These results demonstrate that macrophage-specific THBS1 knockdown mitigates OGD/R-induced hepatocyte apoptosis.

### THBS1 promoted hepatocyte apoptosis by inhibiting PI3K-AKT- NF-κB signaling

**Figure 10:**
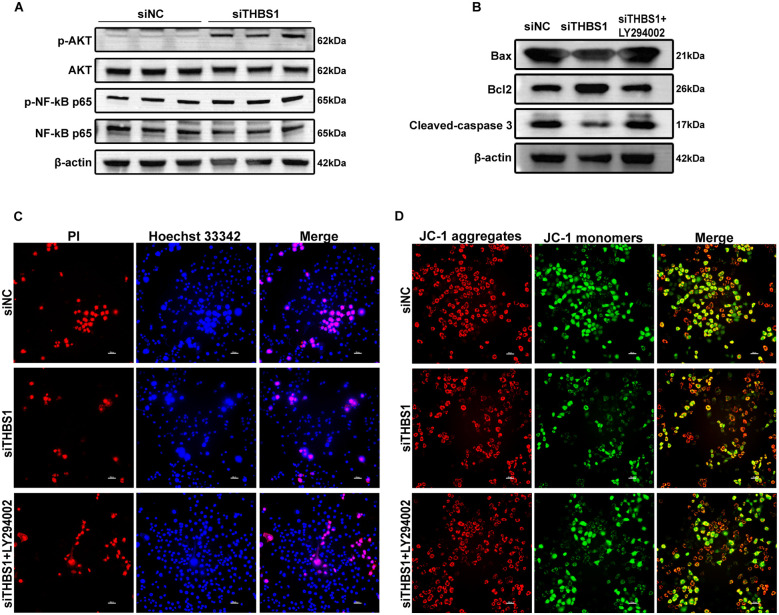
THBS1 promoted hepatocyte apoptosis by inhibiting PI3K-AKT- NF-κB signaling. **(A)** The expression of p-AKT, AKT, p-NF-κB p65 and NF-κB p65 proteins in AML12 cells between siRNA-NC and siRNA-THBS1 groups. **(B)** The expression of pro-apoptotic proteins Bax, Cleaved-caspase 3, and anti-apoptotic Bcl2 protein in AML12 cells among the siRNA-NC, siRNA-THBS1 and siRNA-THBS1+ LY294002 groups. **(C)** Cell death in the siRNA-NC, siRNA-THBS1 and siRNA-THBS1+ LY294002 groups was assessed using Hoechst 33342 and PI double staining. Scale bar = 50μm. **(D)** The mitochondrial membrane potential (ΔψM) of the siRNA-NC, siRNA-THBS1 and siRNA-THBS1+ LY294002 was detected using JC-1 staining. Scale bar = 50μm.

Our findings indicate that macrophage-derived THBS1 modulates hepatocyte apoptosis, with the PI3K-AKT-NF-κB signaling pathway closely associated with this process. To elucidate the potential mechanism, we assessed PI3K-AKT-NF-κB pathway activation in AML12 cells following RAW264.7-specific THBS1 knockdown via Western blot analysis. THBS1 knockdown increased phosphorylation of AKT and NF-κB p65 in AML12 cells (**Figure 10A**). To confirm pathway dependency, co-cultured cells were treated with LY294002 (a PI3K/AKT pathway inhibitor, 50µM), which reversed the THBS1 knockdown-mediated reductions in apoptosis and cell death, as well as the restoration of mitochondrial membrane potential (ΔψM) in AML12 cells (**Figures 10B–D**, **Supplementary Figure 11, 12**).

### THBS1-mediated hepatocyte apoptosis depended on the CD47 receptor

**Figure 11:**
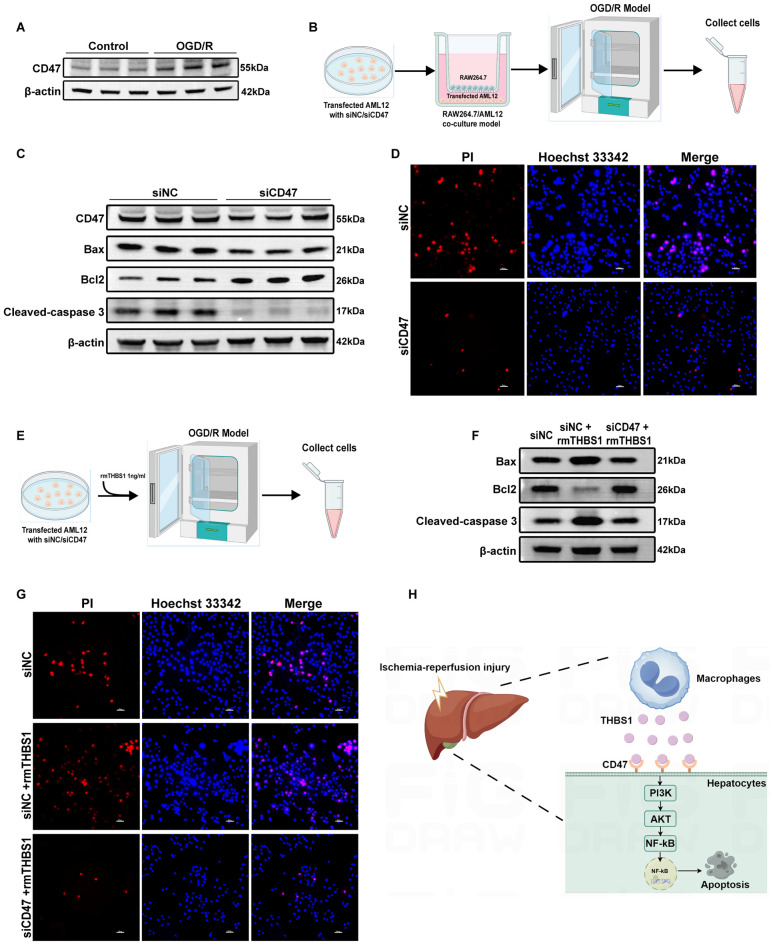
THBS1-mediated hepatocyte apoptosis depended on the CD47 receptor. **(A)** CD47 protein expression in AML12 cells between Control and OGD/R groups. **(B)** Schematic of OGD/R treatment. AML12 cells, separately transfected with siRNA-NC and siRNA-CD47, were seeded in the lower chamber and co-cultured with RAW264.7 cells (upper chamber) under hypoxic conditions (1% O2) in serum-free, glucose-free DMEM for 6 hours, followed by reoxygenation in complete medium for another 3 hours. **(C)** The expression of pro-apoptotic proteins Bax, Cleaved-caspase 3, and anti-apoptotic Bcl2 protein in AML12 cells between siRNA-NC and siRNA-CD47 groups. **(D)** Cell death in the siRNA-NC and siRNA-CD47 groups was assessed using Hoechst 33342 and PI double staining. Scale bar = 50μm. **(E)** Schematic of OGD/R treatment. AML12 cells were separately transfected with siRNA-NC and siRNA-CD47, respectively, then cultured in serum-free, glucose-free DMEM supplemented with 1 ng/mL rmTHBS1 under hypoxic conditions (1% O2) for 6 hours, followed by reoxygenation in complete medium for another 3 hours. **(F)** The expression of pro-apoptotic proteins Bax, Cleaved-caspase 3, and anti-apoptotic Bcl2 protein in AML12 cells among the siRNA-NC, siRNA-NC+ rmTHBS1 and siRNA-CD47+ rmTHBS1 groups. **(G)** Cell death in the siRNA-NC, siRNA-NC+ rmTHBS1 and siRNA-CD47+ rmTHBS1 groups was assessed using Hoechst 33342 and PI double staining. Scale bar = 50μm. **(H)** Schematic diagram of the THBS1-CD47 axis’s role in macrophage-hepatocyte crosstalk during HIRI.

THBS1, an extracellular matrix protein, functions through interactions with various receptors and proteins (22–24). Prior single-cell sequencing data revealed enhanced THBS1-CD47 signaling between macrophages and hepatocytes in post-reperfusion samples. To determine whether macrophage-derived THBS1 modulates apoptosis via hepatocyte CD47, we evaluated CD47 expression in AML12 cells after OGD/R. CD47 protein expression was significantly elevated in AML12 cells after OGD/R (**Figure 11A**). Specific CD47 knockdown (**Supplementary Figure 13**) in AML12 cells reduced pro-apoptotic Bax and Cleaved-caspase 3 expression while increasing anti-apoptotic Bcl2 levels after OGD/R (**Figures 11B, C**). Additionally, OGD/R-induced necrosis in AML12 cells was attenuated by CD47 knockdown (**Figure 11D**, **Supplementary Figure 14**). Recombinant mouse THBS1 (rmTHBS1) protein exacerbated apoptosis and cell death in AML12 cells, whereas CD47 knockdown mitigated rmTHBS1-aggregated apoptosis and cell death (**Figures 11E–G**, **Supplementary Figure 15**).

Finally, this study demonstrated that macrophage-derived THBS1 regulates hepatocyte apoptosis through the CD47 receptor via PI3K-AKT-NF-κB signaling pathway (**Figure 11H**).

## Discussion

Liver transplantation is a primary therapeutic intervention for end-stage liver diseases and hepatocellular carcinoma (25, 26). However, HIRI poses a significant risk for early allograft dysfunction, and is a major contributor to both acute and chronic rejection (27, 28). Current therapeutic strategies for HIRI include scavenging reactive oxygen species (ROS), blocking sterile inflammatory responses, and modulating cytokine storms (1, 29, 30). An increasing number of studies focus on elucidating the biological mechanisms underlying the initiation and progression of HIRI. PCD has emerged as a critical pathway mediating tissue damage. This study integrated bulk and single-cell RNA sequencing data to delineate the heterogeneity of the PCD landscape in HIRI and demonstrated through *in vitro* and *in vivo* experiments that macrophage-derived THBS1 mediated hepatocyte apoptosis via the CD47 receptor through the PI3K-AKT-NF-κB signaling pathway.

Using bulk RNA sequencing data, we identified 25 upregulated DE-PCDRGs across multiple HIRI cohorts. Prior research has shown that the mechanisms underlying HIRI primarily involve microcirculatory dysfunction, hypoxia, oxidative stress, and the activation of cell death signaling pathways (31, 32). These DE-PCDRGs were closely associated with biological processes such as apoptosis, autophagy, and oxidative stress. Subsequently, we developed a robust HIRI risk prediction model using machine learning algorithms. This model was based on five hub genes: THBS1, MAP1LC3B, PPP1R15A, CXCL8, and ZC3H12A, providing a novel assessment framework for clinical HIRI prediction. As determined by the prediction model, the correlation of these hub genes with anoikis, immunogenic cell death, NETosis, netotic cell death, and pyroptosis in high-risk patients supports the notion that diverse PCD patterns drive HIRI progression (3, 33). Furthermore, high-risk patients exhibited M2 macrophage depletion and upregulation of pro-inflammatory cytokines, highlighting the role of immune dysregulation and PCD heterogeneity in HIRI progression.

At single-cell resolution, we identified 12 distinct PCD patterns within the HIRI microenvironment, including ferroptosis in macrophages and necroptosis in neutrophils. This spatial complexity suggests the activation of multiple deaths (such as concurrent autophagy and lysosome-dependent death in neutrophils) may amplify the inflammatory cascade (34–37). Further analysis revealed upregulated expression of THBS1 in macrophages within the tissue microenvironment following reperfusion. Enhanced THBS1-CD47 interactions between macrophages and hepatocytes were validated in subsequent murine HIRI models and clinical liver transplant samples. THBS1, a matricellular protein highly expressed during inflammation, is known for its roles in angiogenesis, platelet aggregation, and immunomodulation, though its function during HIRI remains unclear (38–40). In murine HIRI samples, THBS1 expression in liver tissue macrophages correlated with hepatocyte apoptosis, consistent with observations in acute-on-chronic liver failure (41). We also identified that THBS1 targets the hepatocyte surface receptor CD47, suggesting HIRI-specific mechanisms.

*In vitro*, siRNA-mediated THBS1 targeting significantly reduced apoptosis and decreased pro-inflammatory cytokine secretion, further confirming the specificity of THBS1. Notably, siRNA-mediated silencing of CD47 on hepatocytes also reduced apoptosis, consistent with a recent study in a murine cardiac ischemia-reperfusion model (42), which demonstrated that CD47 blockade reduced apoptosis-related biomarkers, oxidative stress, and improved donor heart preservation. Furthermore, we found that the interaction between macrophage-derived THBS1 and hepatocyte CD47 suppresses the PI3K-AKT-NF-κB signaling pathway. Prior research has demonstrated that activation of the PI3K-AKT-NF-κB pathway can mitigate HIRI by exerting anti-inflammatory, antioxidant, anti-apoptotic, and autophagy-regulatory effects via downstream targets (43, 44). This study expands upon existing findings by identifying the THBS1-CD47 axis as a negative upstream regulator of this signaling cascade in macrophage-hepatocyte interactions. Importantly, the regulation of PI3K-AKT-NF-κB signaling by the THBS1-CD47 axis aligns with recent reports indicating that THBS1 or CD47 blockade inhibits apoptosis through PI3K-AKT pathway activation in glioblastoma and spinal cord injury (45, 46), further supporting the potential of the THBS1-CD47 axis as a modulator of apoptotic signaling in liver injury. While CD47 typically mediates tumor immune evasion through “don’t eat me” signals via binding to SIRPα on macrophages (47, 48), its interaction with THBS1 in regulating apoptosis highlights the importance of assessing disease-specific approaches. Notably, existing studies have shown that THBS1 binding to CD47 increases ROS production and AKT and NF-κB activity, which is consistent with our observation that THBS1 could regulate the phosphorylation of AKT and NF-κB p65 (49). Although this framework plausibly accounts for our data, the precise causal hierarchy remains incompletely defined and warrants future investigation, using THBS1/CD47 blocking antibodies or phosphoproteomic approaches, to validate the role of this signaling pathway in the context of HIRI.

Despite these findings, this study has certain limitations. A limitation of this study pertains to the integration of transcriptomic data obtained from heterogeneous technological platforms (RNA seq and microarray). Despite using validated, platform-specific normalization to reduce batch effects, inherent differences in dynamic range, gene coverage, and sensitivity may affect differential expression results. Future research should adopt a standardized, single-platform approach for validation and extension. Although the integration of integrated transcriptomic approaches provides robust insights into the characterization of PCD patterns in HIRI, the research analyzes a limited number of public datasets with relatively small sample sizes and may be subject to unavoidable selection bias. Furthermore, the functional experiments are conducted using immortalized cell lines, which may not fully recapitulate the complexity of primary cells or human tissues; therefore, primary mouse hepatocytes/macrophages and human-derived cells should be incorporated. Finally, due to the lack of long-term clinical outcome data, we could not perform a correlation analysis to determine whether the THBS1-CD47 axis is associated with adverse outcomes such as early allograft dysfunction or graft survival. The clinical prognostic value of this axis still needs to be verified in future prospective cohorts.

## Conclusion

By integrating bioinformatic analyses with experimental validation, we systematically mapped the heterogeneity of PCD in HIRI and confirmed that the THBS1-CD47 axis regulates macrophage-hepatocyte crosstalk by suppressing PI3K-AKT-NF-κB signaling, thereby driving apoptosis. Targeting this signaling axis may represent a candidate therapeutic strategy, but its application value requires further validation in clinical patients.

## Data Availability

The original contributions presented in the study are included in the article/**Supplementary Material**. Further inquiries can be directed to the corresponding author/s.
