# Supplementary material for: Integrated transcriptomic profiling of programmed cell death patterns unveils macrophage-hepatocyte crosstalk via THBS1-CD47 axis in hepatic ischemia-reperfusion injury
Source: Front Immunol. 2026 May 19;17:1769849. doi: 10.3389/fimmu.2026.1769849 (PMC13225957; doi:10.3389/fimmu.2026.1769849)

**Supplementary Figures**

Figure S1 The bootstrap-corrected calibration curve in training set.

**
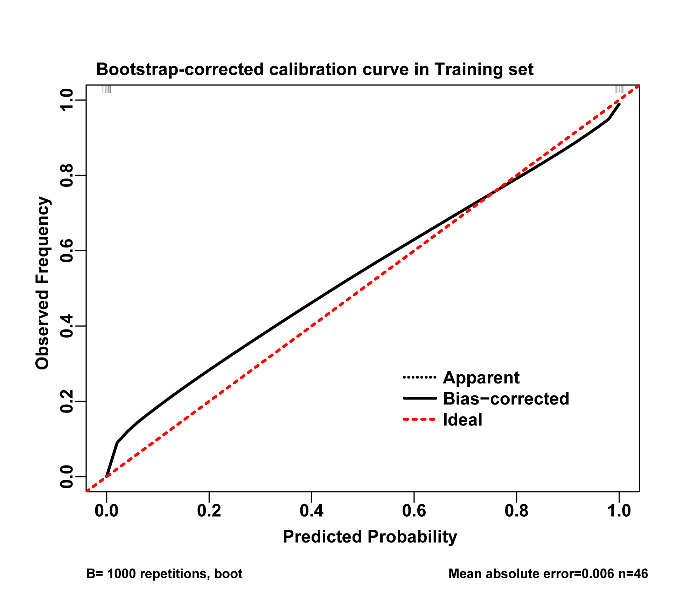
**

Figure S2 The bootstrap-corrected calibration curve in testing set.

**
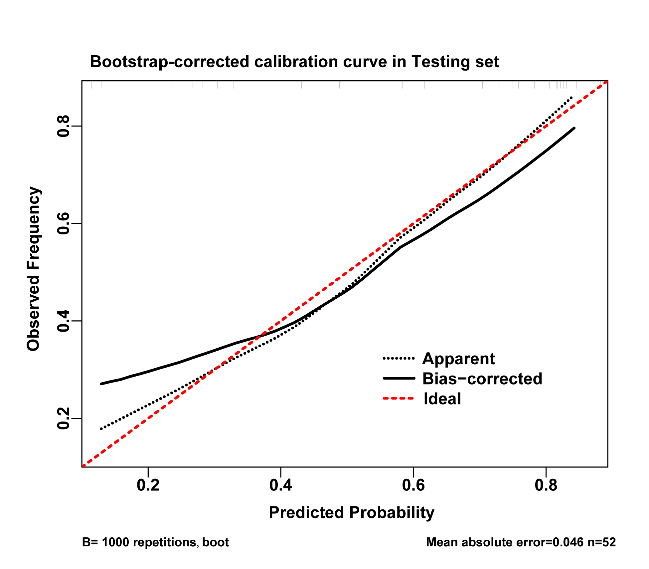
**

Figure S3 TUNEL staining of liver tissue from the Sham and HIRI groups.


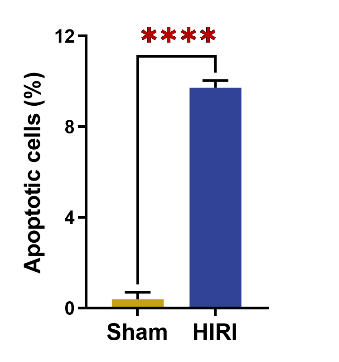


Figure S4 Immunofluorescence staining showed the expressions of pro-apoptotic proteins Bax, Cleaved-caspase 3, and anti-apoptotic Bcl2 protein in liver tissues between the Sham and HIRI groups.


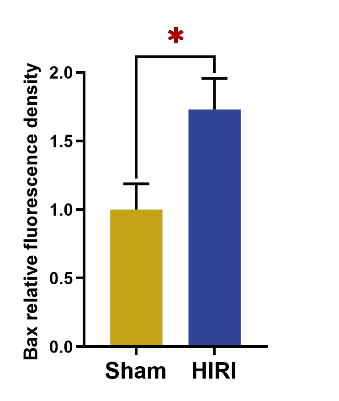

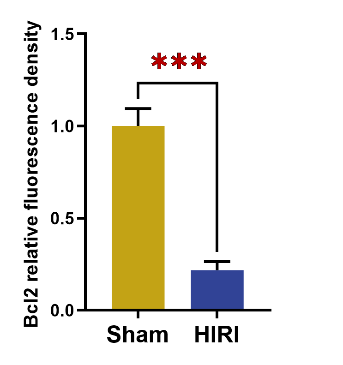

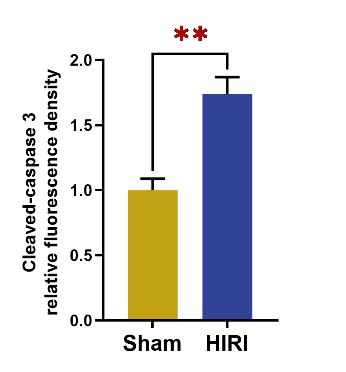


Figure S5 Immunofluorescence double staining revealed THBS1 expression on F4/80-labeled macrophages between the Sham and HIRI groups.


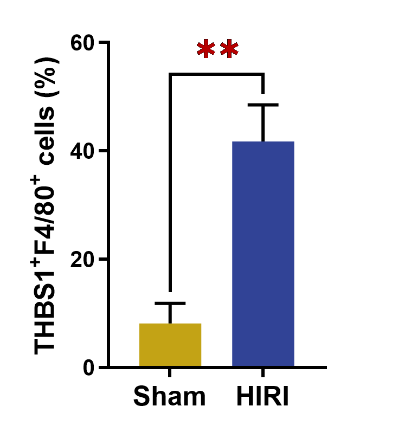


Figure S6 Cell death in the Control and OGD/R groups was assessed using Hoechst 33342 and PI double staining.


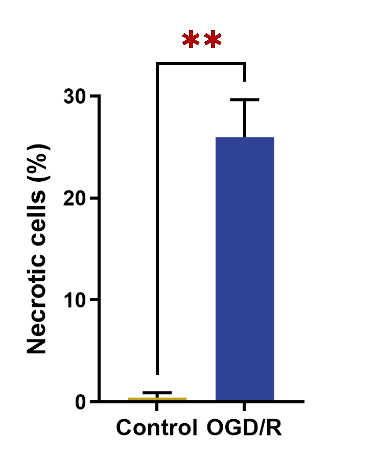


Figure S7 The mitochondrial membrane potential (ΔψM) of the Control and OGD/R groups was detected using JC-1 staining.


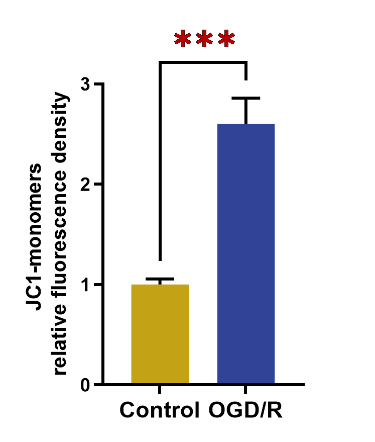


Figure S8 Expression of THBS1 in cells was measured via qRT-PCR.


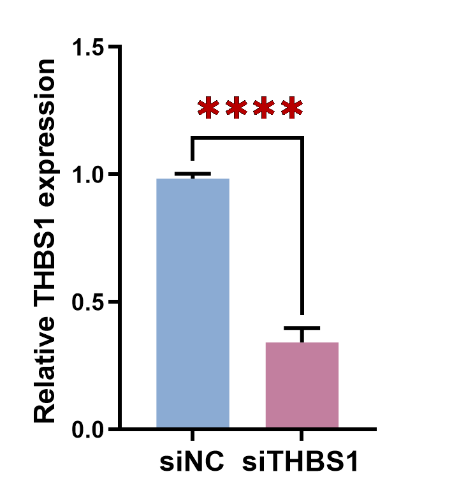


Figure S9 Cell death in the siRNA-NC and siRNA-THBS1 groups was assessed using Hoechst 33342 and PI double staining.


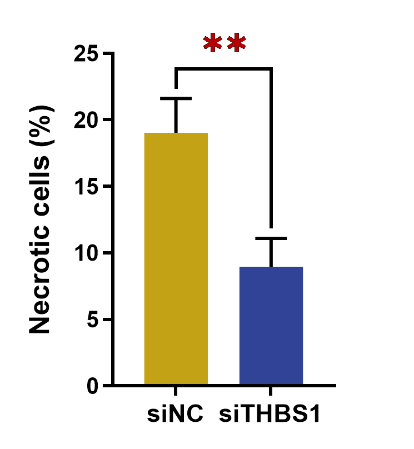


Figure S10 The mitochondrial membrane potential (ΔψM) of the siRNA-NC and siRNA-THBS1 groups was detected using JC-1 staining.


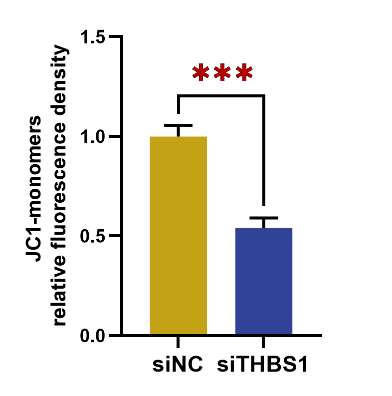


Figure S11 Cell death in the siRNA-NC, siRNA-THBS1 and siRNA-THBS1+ LY294002 groups was assessed using Hoechst 33342 and PI double staining.


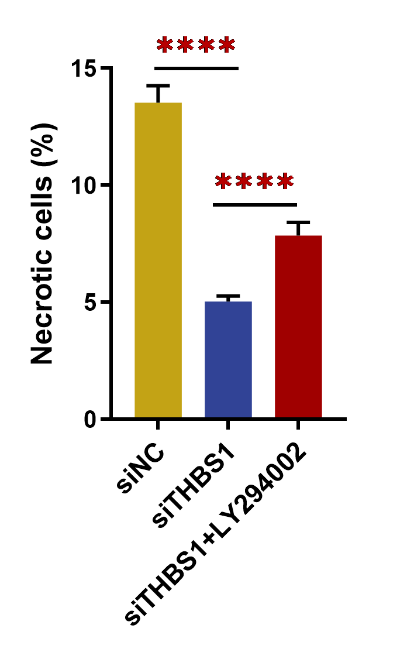


Figure S12 The mitochondrial membrane potential (ΔψM) of the siRNA-NC, siRNA-THBS1 and siRNA-THBS1+ LY294002 was detected using JC-1 staining.


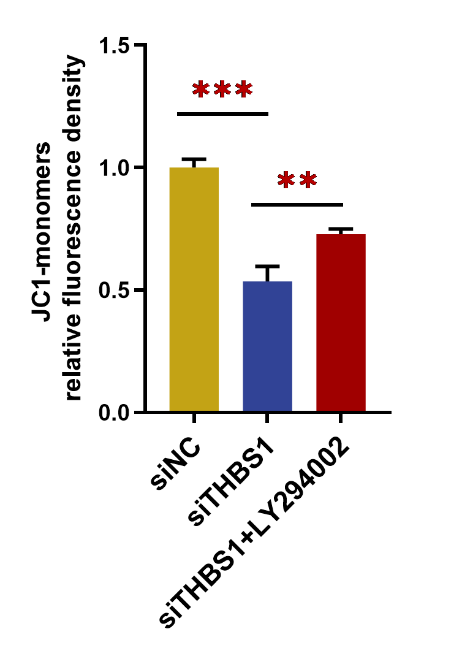


Figure S13 Expression of CD47 in cells was measured via qRT-PCR.


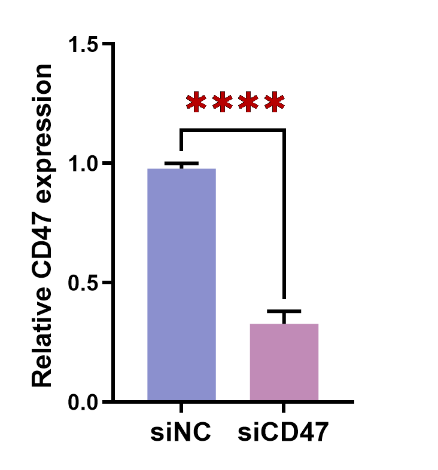


Figure S14 Cell death in the siRNA-NC and siRNA-CD47 groups was assessed using Hoechst 33342 and PI double staining.


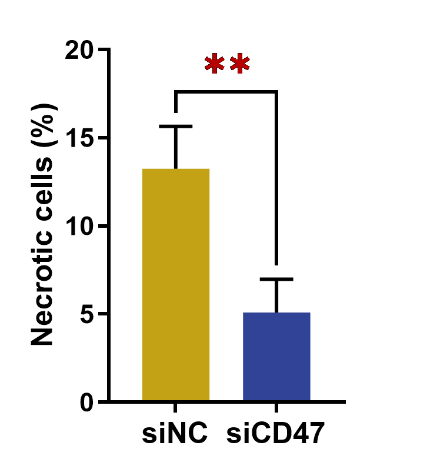


Figure S15 Cell death in the siRNA-NC, siRNA-NC+ rmTHBS1 and siRNA-CD47+ rmTHBS1 groups was assessed using Hoechst 33342 and PI double staining.


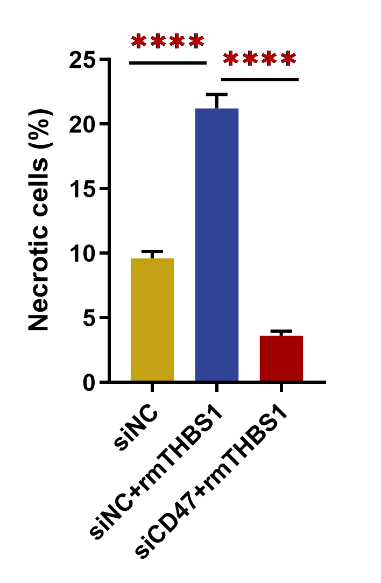

Supplement: Supplementary file 1 [file SupplementaryFile1.docx]
