# Supplementary material for: Integrated transcriptomic profiling of programmed cell death patterns unveils macrophage-hepatocyte crosstalk via THBS1-CD47 axis in hepatic ischemia-reperfusion injury
Source: Front Immunol. 2026 May 19;17:1769849. doi: 10.3389/fimmu.2026.1769849 (PMC13225957; doi:10.3389/fimmu.2026.1769849)
Supplement: Supplementary file 2 [file Table1.docx]

| **Table S1. Main features of the 19 programmed cell death (PCD) categories.** |
| --- |

| **Terms** | **Features** | **Key regulators** |
| --- | --- | --- |
| Apoptosis | Principal pathways encompass the mitochondrial pathway, the death receptor pathway, and the endoplasmic reticulum stress pathway; involving cellular matrix cleavage, nuclear condensation, DNA cleavage, and plasma membrane contraction, culminating in the generation of a conglomerate of apoptotic vesicles | Bax, Bcl-2 family, caspase-2/3/6/7/8/9/10 and p53 |
| Pyroptosis | A type of PCD that critically depends on the formation of plasma membrane pores by members of the gasdermin protein family, often (but not always) as a consequence of inflammatory caspase activation | Caspase-1/3/4/5/8/11, Inflammasomes, GSDM protein family |
| Ferroptosis | A form of PCD initiated by oxidative perturbations of the intracellular microenvironment that is under constitutive control by GPX4 and can be inhibited by iron chelators and lipophilic antioxidants. | SystemXc-/GSH/GPX4, GCH1/DHFR/BH4, FSP1/NADPH/CoQ10 and DHODH/CoQ10 |
| Autophagy | Genetically orchestrated spontaneous cell death, characterized by lysosomal phagocytosis and recycling of autophagic vesicles that encapsulate cytoplasmic contents for subsequent reuse | Autolysosomes, mTOR and ATGs |
| Necroptosis | A modality of PCD triggered by perturbations of extracellular or intracellular homeostasis that critically depends on MLKL, RIPK3, and (at least in some settings) on the kinase activity of RIPK1 | RIPK1/RIPK3/MLKL |
| Cuproptosis | A specific PCD initiated by copper overload and activated by lipid peroxidation and mitochondrial dysfunction | FDX1/DLAT/LIAS/LIPT1、DLD/ATP7A/ATP7B |
| Parthanatos | A modality of PCD initiated by PARP1 hyperactivation and precipitated by the consequent bioenergetic catastrophe coupled to AIF-dependent and MIF-dependent DNA degradation | NAD+ and ATP depletion, PARP1/AIF/MIF |
| Entotic cell death | A type of PCD that originates from actomyosin-dependent cell-in-cell internalization (entosis) and is executed by lysosomes | Cellular engulfment, cell adhesion and cytoskeletal remodeling |
| Netotic cell death | A ROS-dependent modality of RCD restricted to cells of hematopoietic derivation and associated with NET extrusion | The formation and release of extracellular traps (NETs), NET formation-related proteins (such as PAD4), cell death signaling pathways (such as RIPK1) |
| Lysosome-dependent cell death | Arises from the disruption of lysosomal function, leading to the release of its contents | Primary LMP, cathepsins, MOMP and caspases. |
| Alkaliptosis | Provoked by alkaline conditions, is characterized by elevated intracellular pH | Mitochondrial dysfunction, and cell membrane rupture |
| Oxeiptosis | An apoptotic-like cell death pathway | Increased ROS generation, independently of caspase activation |
| NETosis | In response to several stimuli, neutrophils and eosinophils can release the so-called neutrophil extracellular traps (NETs), that is, microbicidal structures composed of nuclear chromatin, histones and granular antimicrobial proteins | Caspase inhibition, NADPH oxidase activation, NET release (in some instances) |
| Immunogenic cell death | A form of PCD that is sufficient to activate an adaptive immune response in immunocompetent hosts | Intracellular signaling pathways activation (endoplasmic reticulum stress, mitochondrial dysfunction), intracellular proteins release and immune responses |
| Anoikis | Specific variant of intrinsic apoptosis initiated by the loss of integrin-dependent anchorage | EGFR, ERK1, β1-integrin, BIM, Caspase-3 (-6,-7). |
| Paraptosis | Distinguished by cell swelling, mitochondrial enlargement, and endoplasmic reticulum breakdown, constitutes a non-apoptotic form of cell death | Mediation by MAP kinases and inhibition by AIP-1/Alix |
| Methuosis | Involved lysosomal enlargement, cytoplasmic dissolution, and subsequent cell breakdown and death | RAS/Rac1/Arf6, macropinosome |
| Entosis | A cell death mechanism linked to the ‘cell-in-cell' phenotype that is frequently exhibited by non-phagocytic cells in clinical tumor samples | RHO and ROCK1 activation |
| Disulfidptosis | Promoted cell demise through the generation and disruption of intracellular disulfide bonds, | Oxidative stress, cell membrane rupture, and the release of cellular contents |
