# Supplementary material for: Integrated transcriptomic profiling of programmed cell death patterns unveils macrophage-hepatocyte crosstalk via THBS1-CD47 axis in hepatic ischemia-reperfusion injury
Source: Front Immunol. 2026 May 19;17:1769849. doi: 10.3389/fimmu.2026.1769849 (PMC13225957; doi:10.3389/fimmu.2026.1769849)
Supplement: Supplementary file 3 [file Table2.docx]

| **Table S2. Differentially expressed genes in GSE151648.** | | | | | | | |
| --- | --- | --- | --- | --- | --- | --- | --- |
|  | logFC | AveExpr | t | P.Value | adj.P.Val | B | change |
| HAS1 | 4.384331 | -2.95172 | 11.92936 | 1.96E-15 | 1.05E-10 | 24.52928 | UP |
| C11orf91 | 4.449503 | -2.59128 | 11.71714 | 3.59E-15 | 1.05E-10 | 23.97136 | UP |
| CSF3 | 5.236828 | -2.50361 | 11.41596 | 8.61E-15 | 1.67E-10 | 22.92353 | UP |
| EGR4 | 4.045125 | -3.18116 | 11.18261 | 1.71E-14 | 2.41E-10 | 22.36632 | UP |
| PLK2 | 2.498141 | 5.058214 | 11.1183 | 2.06E-14 | 2.41E-10 | 21.41208 | UP |
| IER5 | 2.360321 | 3.614834 | 10.65947 | 8.13E-14 | 7.58E-10 | 20.23669 | UP |
| TNFAIP3 | 2.52768 | 5.465602 | 10.6224 | 9.10E-14 | 7.58E-10 | 19.85075 | UP |
| NFKB2 | 1.801801 | 4.84891 | 10.29784 | 2.44E-13 | 1.70E-09 | 18.92466 | UP |
| RIPK2 | 1.455602 | 2.896744 | 10.15513 | 3.79E-13 | 2.21E-09 | 18.76976 | UP |
| TNFRSF10B | 1.159857 | 6.407262 | 10.27507 | 2.62E-13 | 1.70E-09 | 18.61605 | UP |
| CDKN1A | 2.864512 | 7.267245 | 10.08976 | 4.63E-13 | 2.46E-09 | 17.94666 | UP |
| CCL2 | 3.999776 | 4.120285 | 9.91574 | 7.95E-13 | 3.67E-09 | 17.91308 | UP |
| TGFB3 | 2.527908 | 3.773979 | 9.906279 | 8.18E-13 | 3.67E-09 | 17.87999 | UP |
| SPRY2 | 1.414548 | 4.518103 | 9.863103 | 9.36E-13 | 3.76E-09 | 17.60661 | UP |
| ARID5B | 1.813651 | 4.425524 | 9.852335 | 9.68E-13 | 3.76E-09 | 17.59379 | UP |
| SERTAD1 | 2.545314 | 3.596923 | 9.780019 | 1.21E-12 | 4.16E-09 | 17.50875 | UP |
| IL6 | 6.003703 | -1.58697 | 9.516455 | 2.78E-12 | 6.56E-09 | 17.22054 | UP |
| RP11-473M20.5 | 2.299562 | -4.55809 | 9.495816 | 2.97E-12 | 6.65E-09 | 17.11652 | UP |
| KDM6B | 2.158905 | 6.383898 | 9.751238 | 1.33E-12 | 4.30E-09 | 16.99234 | UP |
| FOSL2 | 2.18107 | 6.284625 | 9.722424 | 1.45E-12 | 4.46E-09 | 16.91581 | UP |
| ELMSAN1 | 2.403067 | 5.795244 | 9.697734 | 1.57E-12 | 4.58E-09 | 16.91456 | UP |
| PSMD10P2 | 3.141845 | -3.59652 | 9.34928 | 4.72E-12 | 9.18E-09 | 16.79859 | UP |
| MAP1LC3B | 1.989381 | 5.689601 | 9.5815 | 2.26E-12 | 5.74E-09 | 16.5502 | UP |
| CTD-3247F14.2 | 2.76459 | -3.86737 | 9.252322 | 6.43E-12 | 1.01E-08 | 16.50206 | UP |
| TRIM40 | 2.691994 | -4.46699 | 9.291555 | 5.68E-12 | 1.00E-08 | 16.47552 | UP |
| CCDC9 | 1.311258 | 4.111837 | 9.481553 | 3.10E-12 | 6.70E-09 | 16.45323 | UP |
| NFKBIA | 1.275559 | 7.269166 | 9.584123 | 2.25E-12 | 5.74E-09 | 16.31645 | UP |
| UBC | 1.228431 | 8.369883 | 9.619782 | 2.01E-12 | 5.57E-09 | 16.26948 | UP |
| DUSP5 | 2.78704 | 4.436922 | 9.397571 | 4.05E-12 | 8.14E-09 | 16.16938 | UP |
| ICAM1 | 3.419904 | 7.140658 | 9.51291 | 2.81E-12 | 6.56E-09 | 16.15654 | UP |
| PLK3 | 3.13449 | 3.593695 | 9.263687 | 6.20E-12 | 1.00E-08 | 15.8828 | UP |
| MMP10 | 3.9673 | -3.28833 | 9.063335 | 1.18E-11 | 1.68E-08 | 15.7754 | UP |
| KRT16 | 2.748228 | -4.35484 | 9.039659 | 1.27E-11 | 1.72E-08 | 15.73484 | UP |
| STC2 | 4.129075 | -1.65144 | 8.982489 | 1.53E-11 | 1.94E-08 | 15.71307 | UP |
| UGCG | 1.415347 | 4.721854 | 9.275422 | 5.98E-12 | 1.00E-08 | 15.70183 | UP |
| ZC3H12A | 3.231019 | 4.93104 | 9.26906 | 6.10E-12 | 1.00E-08 | 15.69457 | UP |
| USP36 | 1.220525 | 5.092564 | 9.272432 | 6.03E-12 | 1.00E-08 | 15.63465 | UP |
| XIRP1 | 3.328436 | -3.62085 | 8.998863 | 1.45E-11 | 1.92E-08 | 15.6328 | UP |
| ADRB2 | 2.113606 | 3.274643 | 9.17756 | 8.17E-12 | 1.22E-08 | 15.62031 | UP |
| CALCA | 5.133584 | 2.236979 | 9.04741 | 1.24E-11 | 1.72E-08 | 15.51205 | UP |
| ITPKC | 2.459903 | 4.923909 | 9.208117 | 7.41E-12 | 1.14E-08 | 15.47528 | UP |
| BTG3 | 1.75476 | 1.373799 | 8.959843 | 1.64E-11 | 2.04E-08 | 15.21784 | UP |
| MCL1 | 1.588436 | 8.621104 | 9.289046 | 5.72E-12 | 1.00E-08 | 15.17775 | UP |
| RELB | 2.756236 | 2.751181 | 8.988061 | 1.50E-11 | 1.94E-08 | 15.11233 | UP |
| PER2 | 1.68813 | 4.502889 | 9.074649 | 1.14E-11 | 1.66E-08 | 15.08917 | UP |
| MTHFD2 | 2.087628 | 1.426647 | 8.907716 | 1.95E-11 | 2.18E-08 | 15.04797 | UP |
| HAS2 | 4.612552 | -1.15959 | 8.735141 | 3.40E-11 | 3.31E-08 | 14.88487 | UP |
| SPHK1 | 3.580835 | 1.472224 | 8.82731 | 2.52E-11 | 2.58E-08 | 14.83589 | UP |
| DNAJA4 | 3.928595 | 3.33041 | 8.914108 | 1.91E-11 | 2.18E-08 | 14.82826 | UP |
| RP11-667K14.3 | 3.586706 | -2.861 | 8.71606 | 3.62E-11 | 3.31E-08 | 14.79051 | UP |
| CDC42EP2 | 2.031239 | 2.731416 | 8.875575 | 2.16E-11 | 2.37E-08 | 14.72452 | UP |
| UPP1 | 1.90015 | 4.4938 | 8.942208 | 1.74E-11 | 2.07E-08 | 14.66379 | UP |
| PLEKHG2 | 1.088208 | 3.973486 | 8.914513 | 1.90E-11 | 2.18E-08 | 14.63881 | UP |
| PRR7-AS1 | 2.422838 | -4.39129 | 8.654238 | 4.43E-11 | 3.86E-08 | 14.45581 | UP |
| CKS2 | 2.216401 | 2.28078 | 8.719124 | 3.59E-11 | 3.31E-08 | 14.2931 | UP |
| ELL | 1.219166 | 4.621622 | 8.834215 | 2.47E-11 | 2.57E-08 | 14.28094 | UP |
| WEE1 | 2.477712 | 4.885219 | 8.812013 | 2.65E-11 | 2.67E-08 | 14.19473 | UP |
| HSPH1 | 2.862341 | 6.25766 | 8.842779 | 2.40E-11 | 2.54E-08 | 14.10146 | UP |
| GJA1 | 2.428317 | 2.2799 | 8.64911 | 4.50E-11 | 3.86E-08 | 14.07189 | UP |
| ALOXE3 | 2.92612 | -3.95187 | 8.492981 | 7.50E-11 | 5.91E-08 | 13.9952 | UP |
| THBS1 | 2.926878 | 7.324301 | 8.85708 | 2.29E-11 | 2.47E-08 | 13.99415 | UP |
| CXCL8 | 4.938729 | 1.821537 | 8.546413 | 6.30E-11 | 5.10E-08 | 13.92895 | UP |
| PLAUR | 3.305675 | 2.886995 | 8.598202 | 5.32E-11 | 4.49E-08 | 13.83814 | UP |
| NOP58 | 1.097386 | 5.062006 | 8.716138 | 3.62E-11 | 3.31E-08 | 13.82724 | UP |
| ICAM4 | 5.909919 | -0.8974 | 8.410332 | 9.84E-11 | 7.26E-08 | 13.77457 | UP |
| BIRC3 | 3.727874 | 4.603959 | 8.652721 | 4.45E-11 | 3.86E-08 | 13.75506 | UP |
| ASTL | 2.914555 | -3.55011 | 8.396769 | 1.03E-10 | 7.31E-08 | 13.74021 | UP |
| MAFK | 1.856838 | 5.605265 | 8.704569 | 3.76E-11 | 3.37E-08 | 13.72067 | UP |
| CTD-2306A12.1 | 2.412764 | -4.34634 | 8.423659 | 9.42E-11 | 7.08E-08 | 13.71788 | UP |
| CCNL1 | 1.28777 | 6.651823 | 8.714946 | 3.64E-11 | 3.31E-08 | 13.59197 | UP |
| ARL14 | 5.696189 | -1.59454 | 8.374687 | 1.11E-10 | 7.63E-08 | 13.58418 | UP |
| ADAMTS4 | 4.335189 | 3.960922 | 8.478416 | 7.87E-11 | 6.12E-08 | 13.31555 | UP |
| NUAK2 | 2.324661 | 2.280556 | 8.399881 | 1.02E-10 | 7.31E-08 | 13.24401 | UP |
| PTGS2 | 4.508118 | 1.185715 | 8.311468 | 1.36E-10 | 8.36E-08 | 13.23077 | UP |
| BAG3 | 3.038388 | 6.005128 | 8.560896 | 6.01E-11 | 4.93E-08 | 13.21783 | UP |
| OLR1 | 3.704384 | -0.99879 | 8.223106 | 1.82E-10 | 1.00E-07 | 13.19296 | UP |
| CCL8 | 3.542208 | -2.60884 | 8.186847 | 2.05E-10 | 1.11E-07 | 13.09762 | UP |
| TP53BP2 | 1.773959 | 5.241586 | 8.495246 | 7.45E-11 | 5.91E-08 | 13.08341 | UP |
| JMJD6 | 1.13463 | 3.899784 | 8.435707 | 9.05E-11 | 6.94E-08 | 13.07667 | UP |
| MAFF | 3.75032 | 4.357251 | 8.373066 | 1.11E-10 | 7.63E-08 | 12.87536 | UP |
| HCAR2 | 3.83873 | -1.59626 | 8.1083 | 2.66E-10 | 1.29E-07 | 12.85082 | UP |
| DNAJB5 | 1.114497 | 2.254825 | 8.277617 | 1.52E-10 | 9.06E-08 | 12.81245 | UP |
| RNF19B | 1.35135 | 3.660562 | 8.342499 | 1.23E-10 | 8.09E-08 | 12.80743 | UP |
| PELI1 | 1.650577 | 4.472857 | 8.341194 | 1.23E-10 | 8.09E-08 | 12.68496 | UP |
| NCOA7 | 2.406977 | 5.496031 | 8.349807 | 1.20E-10 | 8.09E-08 | 12.57667 | UP |
| MMP19 | 2.336535 | 4.13109 | 8.280962 | 1.51E-10 | 9.05E-08 | 12.55298 | UP |
| MIDN | 1.814144 | 6.945837 | 8.40294 | 1.01E-10 | 7.31E-08 | 12.52752 | UP |
| RALGDS | 3.130752 | 5.340522 | 8.314146 | 1.35E-10 | 8.36E-08 | 12.50339 | UP |
| BMP2 | 1.907074 | 3.238413 | 8.226103 | 1.80E-10 | 1.00E-07 | 12.4995 | UP |
| RP11-47I22.2 | 2.384277 | -4.28702 | 8.045445 | 3.28E-10 | 1.52E-07 | 12.49249 | UP |
| PDGFA | 1.799359 | 2.127119 | 8.153788 | 2.29E-10 | 1.19E-07 | 12.43654 | UP |
| SLC20A1 | 3.045648 | 6.105688 | 8.315603 | 1.34E-10 | 8.36E-08 | 12.3918 | UP |
| COQ10B | 1.082982 | 4.62577 | 8.251797 | 1.66E-10 | 9.48E-08 | 12.35717 | UP |
| IL1RN | 3.023889 | 6.86097 | 8.318358 | 1.33E-10 | 8.36E-08 | 12.2892 | UP |
| KLHL21 | 1.578177 | 6.058184 | 8.292993 | 1.45E-10 | 8.79E-08 | 12.28871 | UP |
| TNFRSF12A | 3.360888 | 4.064841 | 8.175097 | 2.14E-10 | 1.14E-07 | 12.24452 | UP |
| TNFRSF10D | 1.959372 | 4.814663 | 8.204371 | 1.94E-10 | 1.06E-07 | 12.18496 | UP |
| IRF1 | 2.078737 | 5.581759 | 8.23735 | 1.74E-10 | 9.84E-08 | 12.18263 | UP |
| RIPK4 | 2.229062 | 5.49907 | 8.226612 | 1.80E-10 | 1.00E-07 | 12.16319 | UP |
| MYC | 2.727509 | 6.251047 | 8.255507 | 1.64E-10 | 9.45E-08 | 12.16178 | UP |
| LIF | 6.597085 | 0.810756 | 7.938412 | 4.68E-10 | 1.99E-07 | 12.13763 | UP |
| HSP90AB1 | 1.332802 | 8.430428 | 8.336738 | 1.25E-10 | 8.12E-08 | 12.08429 | UP |
| SERPINB9 | 2.133115 | 4.476076 | 8.148591 | 2.33E-10 | 1.20E-07 | 12.05401 | UP |
| FSTL3 | 1.696313 | 4.17856 | 8.115742 | 2.60E-10 | 1.27E-07 | 11.9798 | UP |
| PLEKHB2 | 1.064295 | 5.083469 | 8.153632 | 2.29E-10 | 1.19E-07 | 11.96201 | UP |
| FOXC1 | 2.098625 | 0.661013 | 7.925214 | 4.89E-10 | 2.04E-07 | 11.92729 | UP |
| TSC22D2 | 1.974769 | 5.492239 | 8.146579 | 2.35E-10 | 1.20E-07 | 11.89238 | UP |
| TIPARP | 1.947216 | 5.388375 | 8.140419 | 2.40E-10 | 1.21E-07 | 11.8865 | UP |
| HSPB8 | 1.350984 | 1.35409 | 7.948625 | 4.53E-10 | 1.95E-07 | 11.87294 | UP |
| AKAP12 | 2.569707 | 6.204226 | 8.163889 | 2.22E-10 | 1.17E-07 | 11.85906 | UP |
| MLKL | 1.357555 | 3.241671 | 8.022138 | 3.55E-10 | 1.60E-07 | 11.80542 | UP |
| MIR222HG | 3.067306 | -0.19323 | 7.833344 | 6.65E-10 | 2.53E-07 | 11.7804 | UP |
| ANKRD1 | 3.713649 | -2.75389 | 7.801144 | 7.40E-10 | 2.72E-07 | 11.77379 | UP |
| ARL5B | 2.336346 | 6.024097 | 8.12866 | 2.49E-10 | 1.23E-07 | 11.76235 | UP |
| GPR183 | 2.006658 | 1.30908 | 7.90206 | 5.29E-10 | 2.14E-07 | 11.74007 | UP |
| CTD-2369P2.8 | 3.731532 | 0.476711 | 7.843111 | 6.43E-10 | 2.48E-07 | 11.73699 | UP |
| MAP3K8 | 1.386469 | 3.605599 | 7.997732 | 3.85E-10 | 1.70E-07 | 11.66798 | UP |
| ZBTB43 | 1.206282 | 4.216694 | 8.020124 | 3.57E-10 | 1.60E-07 | 11.64739 | UP |
| ATP1B3 | 1.424727 | 3.459025 | 7.975484 | 4.14E-10 | 1.81E-07 | 11.61666 | UP |
| TRAF4 | 1.034029 | 5.631973 | 8.064598 | 3.08E-10 | 1.45E-07 | 11.58322 | UP |
| HAPLN3 | 2.306742 | -0.47364 | 7.765867 | 8.33E-10 | 3.00E-07 | 11.57675 | UP |
| BDKRB2 | 3.173053 | -1.54443 | 7.718237 | 9.76E-10 | 3.35E-07 | 11.55578 | UP |
| JUND | 1.956862 | 7.007733 | 8.102759 | 2.71E-10 | 1.31E-07 | 11.5232 | UP |
| MAT2A | 1.057039 | 6.239797 | 8.060191 | 3.13E-10 | 1.46E-07 | 11.47923 | UP |
| DNAJB1 | 3.162044 | 7.344831 | 8.087358 | 2.86E-10 | 1.35E-07 | 11.45125 | UP |
| SDC4 | 2.077309 | 8.533495 | 8.133935 | 2.45E-10 | 1.23E-07 | 11.40642 | UP |
| GPCPD1 | 1.842387 | 4.392688 | 7.938192 | 4.69E-10 | 1.99E-07 | 11.35733 | UP |
| GEM | 2.259878 | 2.23892 | 7.828305 | 6.76E-10 | 2.53E-07 | 11.3446 | UP |
| H3F3B | 1.218072 | 7.101347 | 8.039669 | 3.35E-10 | 1.54E-07 | 11.28643 | UP |
| RP11-462D18.2 | 2.107417 | -4.89747 | 7.731507 | 9.34E-10 | 3.26E-07 | 11.26164 | UP |
| PPRC1 | 1.865242 | 4.936852 | 7.930298 | 4.81E-10 | 2.02E-07 | 11.24924 | UP |
| EMP1 | 3.003755 | 4.376047 | 7.877616 | 5.73E-10 | 2.27E-07 | 11.18867 | UP |
| CRISPLD2 | 1.466175 | 4.754849 | 7.891993 | 5.47E-10 | 2.20E-07 | 11.14099 | UP |
| RASL11A | 2.12252 | 3.511226 | 7.829828 | 6.73E-10 | 2.53E-07 | 11.13519 | UP |
| ADAMTS1 | 2.487167 | 5.665828 | 7.912206 | 5.11E-10 | 2.11E-07 | 11.09409 | UP |
| CCRL2 | 1.959288 | 1.070788 | 7.695083 | 1.06E-09 | 3.56E-07 | 11.08443 | UP |
| PHACTR1 | 1.993529 | 1.249157 | 7.694973 | 1.06E-09 | 3.56E-07 | 11.0555 | UP |
| DCUN1D3 | 1.016594 | 4.426972 | 7.849398 | 6.30E-10 | 2.46E-07 | 11.04151 | UP |
| BCL2A1 | 2.667156 | 0.877134 | 7.66676 | 1.16E-09 | 3.86E-07 | 11.04114 | UP |
| MESDC1 | 1.495794 | 3.9508 | 7.788505 | 7.72E-10 | 2.81E-07 | 10.91531 | UP |
| FAM46A | 2.019325 | 5.565909 | 7.825808 | 6.82E-10 | 2.53E-07 | 10.80832 | UP |
| CXCL5 | 3.084305 | -2.56708 | 7.497941 | 2.04E-09 | 6.17E-07 | 10.80478 | UP |
| AEN | 1.63916 | 3.967391 | 7.729057 | 9.42E-10 | 3.27E-07 | 10.71521 | UP |
| MSX1 | 2.477671 | -1.1136 | 7.473685 | 2.22E-09 | 6.60E-07 | 10.68735 | UP |
| SBNO2 | 1.128858 | 4.44321 | 7.724834 | 9.55E-10 | 3.29E-07 | 10.62154 | UP |
| RELT | 1.552384 | 2.077154 | 7.596812 | 1.47E-09 | 4.70E-07 | 10.57213 | UP |
| LDLR | 2.1662 | 7.870572 | 7.854488 | 6.19E-10 | 2.44E-07 | 10.57028 | UP |
| MYADM | 1.380497 | 5.875588 | 7.753045 | 8.69E-10 | 3.09E-07 | 10.50726 | UP |
| SEMA6B | 1.63733 | 3.5506 | 7.645104 | 1.25E-09 | 4.08E-07 | 10.49702 | UP |
| GAL | 2.943702 | -4.06384 | 7.440975 | 2.48E-09 | 7.22E-07 | 10.48059 | UP |
| PGM2 | 1.015918 | 4.195469 | 7.641392 | 1.26E-09 | 4.11E-07 | 10.37643 | UP |
| PHLDA1 | 2.19588 | 6.737383 | 7.745662 | 8.91E-10 | 3.13E-07 | 10.37074 | UP |
| SLC41A1 | 1.090726 | 3.406479 | 7.591774 | 1.49E-09 | 4.75E-07 | 10.33018 | UP |
| KRT16P3 | 5.007388 | -2.399 | 7.441117 | 2.47E-09 | 7.22E-07 | 10.32804 | UP |
| B3GNT5 | 3.304547 | 1.394845 | 7.470345 | 2.24E-09 | 6.64E-07 | 10.32094 | UP |
| IRF8 | 1.193311 | 4.271574 | 7.617978 | 1.37E-09 | 4.40E-07 | 10.28787 | UP |
| SNAI1 | 1.702437 | 2.312794 | 7.519774 | 1.90E-09 | 5.80E-07 | 10.27537 | UP |
| DYRK3 | 1.556414 | 2.364252 | 7.522757 | 1.88E-09 | 5.77E-07 | 10.27387 | UP |
| LAMC2 | 3.58386 | -0.54573 | 7.362045 | 3.23E-09 | 9.05E-07 | 10.25818 | UP |
| STX11 | 1.792865 | 2.818437 | 7.536963 | 1.79E-09 | 5.59E-07 | 10.25274 | UP |
| RGCC | 3.342437 | 0.739617 | 7.417457 | 2.68E-09 | 7.66E-07 | 10.24907 | UP |
| NOLC1 | 1.332303 | 5.839855 | 7.667123 | 1.16E-09 | 3.86E-07 | 10.22203 | UP |
| RELL1 | 1.482344 | 2.243525 | 7.494313 | 2.07E-09 | 6.22E-07 | 10.19707 | UP |
| CILP2 | 3.015023 | -2.77164 | 7.301389 | 3.96E-09 | 1.07E-06 | 10.17076 | UP |
| GTPBP1 | 1.063689 | 5.310194 | 7.625208 | 1.33E-09 | 4.32E-07 | 10.15619 | UP |
| C5orf52 | 3.380899 | -3.59844 | 7.337465 | 3.51E-09 | 9.72E-07 | 10.08942 | UP |
| CHSY1 | 1.15852 | 4.020886 | 7.541708 | 1.76E-09 | 5.53E-07 | 10.06803 | UP |
| PIGA | 1.380641 | 3.478604 | 7.509994 | 1.96E-09 | 5.96E-07 | 10.04767 | UP |
| CD83 | 3.389255 | 1.936096 | 7.407174 | 2.77E-09 | 7.89E-07 | 10.02404 | UP |
| NDEL1 | 1.05821 | 5.569983 | 7.587909 | 1.51E-09 | 4.79E-07 | 9.991607 | UP |
| FGF19 | 4.064695 | -3.08071 | 7.316867 | 3.76E-09 | 1.03E-06 | 9.986708 | UP |
| TRIB1 | 2.224473 | 7.406477 | 7.658836 | 1.19E-09 | 3.95E-07 | 9.981108 | UP |
| GPR4 | 1.464991 | 2.789575 | 7.430723 | 2.56E-09 | 7.39E-07 | 9.890538 | UP |
| VPS37B | 1.137153 | 4.139705 | 7.445741 | 2.44E-09 | 7.17E-07 | 9.725627 | UP |
| MAP3K14 | 2.08919 | 4.341594 | 7.426056 | 2.60E-09 | 7.47E-07 | 9.645303 | UP |
| AC112198.1 | 2.783 | -4.37715 | 7.195516 | 5.66E-09 | 1.41E-06 | 9.573851 | UP |
| TNFRSF9 | 3.800767 | -1.63866 | 7.129913 | 7.07E-09 | 1.68E-06 | 9.569077 | UP |
| C2CD4B | 3.598876 | -0.01474 | 7.171276 | 6.15E-09 | 1.47E-06 | 9.541867 | UP |
| ENO1-IT1 | 2.614626 | -2.63251 | 7.094701 | 7.97E-09 | 1.84E-06 | 9.515548 | UP |
| TMEM88 | 1.606931 | 2.065541 | 7.253284 | 4.66E-09 | 1.23E-06 | 9.414379 | UP |
| ZNF878 | 3.585035 | -2.52392 | 7.086525 | 8.19E-09 | 1.87E-06 | 9.410936 | UP |
| NFKBIE | 1.202013 | 2.461149 | 7.251228 | 4.69E-09 | 1.23E-06 | 9.330935 | UP |
| CHORDC1 | 1.526485 | 3.786332 | 7.310797 | 3.84E-09 | 1.05E-06 | 9.328399 | UP |
| AC058791.1 | 2.80832 | -0.12377 | 7.110244 | 7.56E-09 | 1.76E-06 | 9.327112 | UP |
| ZNF165 | 2.754738 | 0.971234 | 7.160291 | 6.38E-09 | 1.52E-06 | 9.321399 | UP |
| VNN3 | 1.891437 | 3.82625 | 7.293953 | 4.06E-09 | 1.09E-06 | 9.272561 | UP |
| RUNX1 | 1.465957 | 3.347551 | 7.243401 | 4.82E-09 | 1.24E-06 | 9.167912 | UP |
| PIM3 | 1.457238 | 6.517138 | 7.381693 | 3.02E-09 | 8.55E-07 | 9.159828 | UP |
| RRAGC | 1.021775 | 2.870334 | 7.19537 | 5.67E-09 | 1.41E-06 | 9.072837 | UP |
| PDLIM3 | 1.639556 | 2.646748 | 7.178157 | 6.01E-09 | 1.45E-06 | 9.062356 | UP |
| RP11-249C24.10 | 2.168372 | -4.67907 | 7.046442 | 9.38E-09 | 2.06E-06 | 9.020734 | UP |
| EPHA2 | 2.559384 | 5.613201 | 7.293466 | 4.07E-09 | 1.09E-06 | 9.018171 | UP |
| GLA | 1.06116 | 3.391653 | 7.202232 | 5.54E-09 | 1.39E-06 | 9.014103 | UP |
| KLHL15 | 1.815127 | 4.438571 | 7.242447 | 4.83E-09 | 1.24E-06 | 9.003201 | UP |
| MIR23A | 2.39751 | -4.16496 | 6.991764 | 1.13E-08 | 2.35E-06 | 8.986714 | UP |
| CDC37L1 | 1.005444 | 5.40179 | 7.277841 | 4.29E-09 | 1.14E-06 | 8.967193 | UP |
| MID1IP1 | 1.402461 | 5.996233 | 7.299075 | 3.99E-09 | 1.08E-06 | 8.955696 | UP |
| MIR221 | 2.706559 | -2.60338 | 6.932434 | 1.38E-08 | 2.69E-06 | 8.933627 | UP |
| CCDC71L | 1.933985 | 5.297728 | 7.25842 | 4.58E-09 | 1.21E-06 | 8.930379 | UP |
| C15orf39 | 1.122483 | 3.611735 | 7.185709 | 5.86E-09 | 1.44E-06 | 8.92512 | UP |
| CLCF1 | 3.7443 | 1.197733 | 7.038234 | 9.64E-09 | 2.09E-06 | 8.912304 | UP |
| IL1RL1 | 2.017261 | 4.854751 | 7.225233 | 5.12E-09 | 1.30E-06 | 8.886097 | UP |
| RP4-541C22.5 | 2.347553 | -3.78104 | 6.897331 | 1.55E-08 | 2.94E-06 | 8.749684 | UP |
| CASP4 | 1.224096 | 5.451709 | 7.209713 | 5.40E-09 | 1.36E-06 | 8.730777 | UP |
| IFFO2 | 1.316396 | 3.384352 | 7.114395 | 7.45E-09 | 1.74E-06 | 8.720849 | UP |
| PTX3 | 4.005925 | -1.56423 | 6.868893 | 1.71E-08 | 3.18E-06 | 8.674697 | UP |
| TGIF1 | 1.480496 | 4.782681 | 7.161475 | 6.35E-09 | 1.52E-06 | 8.670813 | UP |
| ADM | 1.750616 | 5.125889 | 7.171587 | 6.14E-09 | 1.47E-06 | 8.65798 | UP |
| CTD-2382E5.6 | 2.507589 | -3.59195 | 6.859391 | 1.77E-08 | 3.27E-06 | 8.620547 | UP |
| RP11-383G6.3 | 2.168748 | -4.2373 | 6.897395 | 1.55E-08 | 2.94E-06 | 8.612499 | UP |
| PIM2 | 1.050193 | 2.805524 | 7.04369 | 9.47E-09 | 2.06E-06 | 8.569234 | UP |
| RND1 | 3.089298 | 6.152979 | 7.178806 | 5.99E-09 | 1.45E-06 | 8.564006 | UP |
| RP11-214O1.2 | 2.569425 | 0.516627 | 6.896478 | 1.56E-08 | 2.94E-06 | 8.492627 | UP |
| RP11-242C19.2 | 2.45799 | -0.9909 | 6.824867 | 1.99E-08 | 3.59E-06 | 8.477554 | UP |
| SOX17 | 2.938796 | 1.483344 | 6.934548 | 1.37E-08 | 2.68E-06 | 8.475891 | UP |
| GFPT2 | 2.33089 | 0.888658 | 6.909948 | 1.49E-08 | 2.84E-06 | 8.468872 | UP |
| SRF | 1.110393 | 5.463828 | 7.125116 | 7.19E-09 | 1.69E-06 | 8.44092 | UP |
| RP11-439L18.1 | 2.463392 | -4.01441 | 6.798927 | 2.17E-08 | 3.80E-06 | 8.365247 | UP |
| AC092066.1 | 1.062186 | 2.892987 | 6.979492 | 1.18E-08 | 2.42E-06 | 8.3374 | UP |
| TAC1 | 2.876399 | -4.00816 | 6.804688 | 2.13E-08 | 3.76E-06 | 8.331744 | UP |
| PTP4A1P7 | 1.798457 | -4.83724 | 6.8567 | 1.78E-08 | 3.29E-06 | 8.327889 | UP |
| RBM38 | 1.092463 | 3.619149 | 7.006182 | 1.07E-08 | 2.27E-06 | 8.313683 | UP |
| BATF3 | 2.397923 | -0.0253 | 6.818495 | 2.03E-08 | 3.63E-06 | 8.309484 | UP |
| RANGAP1 | 1.102915 | 6.066572 | 7.112185 | 7.51E-09 | 1.75E-06 | 8.307852 | UP |
| VGF | 2.307289 | -4.87331 | 6.854696 | 1.80E-08 | 3.30E-06 | 8.304006 | UP |
| FAM110C | 1.096407 | 4.553686 | 7.044538 | 9.44E-09 | 2.06E-06 | 8.302491 | UP |
| B4GALT5 | 1.57298 | 5.541712 | 7.085359 | 8.22E-09 | 1.87E-06 | 8.300516 | UP |
| TIFA | 1.211079 | 3.092281 | 6.975198 | 1.19E-08 | 2.44E-06 | 8.292535 | UP |
| PFKFB3 | 1.800278 | 6.003441 | 7.099139 | 7.85E-09 | 1.82E-06 | 8.28384 | UP |
| F3 | 1.586651 | 2.067959 | 6.919095 | 1.44E-08 | 2.79E-06 | 8.27964 | UP |
| RP11-701P16.5 | 3.209983 | -2.47962 | 6.751012 | 2.55E-08 | 4.30E-06 | 8.255916 | UP |
| EIF4A1 | 1.515569 | 2.930774 | 6.953393 | 1.29E-08 | 2.56E-06 | 8.250481 | UP |
| H3F3C | 2.06278 | -4.19437 | 6.777509 | 2.33E-08 | 4.02E-06 | 8.236749 | UP |
| MAP2K3 | 1.221595 | 6.169067 | 7.091551 | 8.05E-09 | 1.85E-06 | 8.223906 | UP |
| DNAJC27 | 1.052865 | 2.084784 | 6.893399 | 1.58E-08 | 2.96E-06 | 8.177198 | UP |
| ARHGEF5 | 1.225876 | 2.530339 | 6.909221 | 1.49E-08 | 2.84E-06 | 8.160011 | UP |
| MAMSTR | 2.173934 | -2.15505 | 6.692353 | 3.12E-08 | 4.97E-06 | 8.151777 | UP |
| DUSP6 | 1.94284 | 6.552199 | 7.07943 | 8.39E-09 | 1.90E-06 | 8.137988 | UP |
| ITPRIP | 1.492012 | 5.027379 | 7.011202 | 1.06E-08 | 2.25E-06 | 8.123731 | UP |
| CCL20 | 6.51268 | 0.233607 | 6.733934 | 2.71E-08 | 4.52E-06 | 8.121389 | UP |
| YBX3 | 1.752801 | 5.000042 | 7.007639 | 1.07E-08 | 2.27E-06 | 8.120038 | UP |
| DDX21 | 1.456335 | 6.2632 | 7.059278 | 8.98E-09 | 1.99E-06 | 8.103467 | UP |
| ZSCAN12P1 | 3.178753 | -0.42198 | 6.731995 | 2.73E-08 | 4.54E-06 | 8.097442 | UP |
| DUSP8 | 3.775774 | -1.22009 | 6.697417 | 3.07E-08 | 4.95E-06 | 8.08026 | UP |
| EREG | 3.033482 | -2.01082 | 6.679429 | 3.26E-08 | 5.12E-06 | 8.07949 | UP |
| ADCYAP1 | 2.181391 | -4.00369 | 6.703772 | 3.00E-08 | 4.90E-06 | 8.074181 | UP |
| LONRF1 | 1.029788 | 3.466854 | 6.925284 | 1.41E-08 | 2.75E-06 | 8.061725 | UP |
| NIPAL4 | 2.388121 | -4.24142 | 6.719777 | 2.84E-08 | 4.69E-06 | 8.037489 | UP |
| SLC2A3 | 2.486652 | 4.144242 | 6.921907 | 1.43E-08 | 2.77E-06 | 7.97643 | UP |
| G0S2 | 2.571707 | 6.610704 | 7.028359 | 9.97E-09 | 2.14E-06 | 7.968883 | UP |
| FRMD4B | 1.402802 | 5.563845 | 6.986146 | 1.15E-08 | 2.38E-06 | 7.957709 | UP |
| SERPINB8 | 1.367184 | 3.555882 | 6.883318 | 1.63E-08 | 3.06E-06 | 7.910163 | UP |
| FAM131A | 1.148356 | 2.220631 | 6.818653 | 2.03E-08 | 3.63E-06 | 7.902475 | UP |
| ATF3 | 3.711151 | 5.567672 | 6.946627 | 1.32E-08 | 2.60E-06 | 7.884365 | UP |
| PRSS22 | 3.227257 | 0.373276 | 6.692523 | 3.12E-08 | 4.97E-06 | 7.844759 | UP |
| TM4SF1 | 2.79447 | 6.022841 | 6.963564 | 1.24E-08 | 2.50E-06 | 7.842947 | UP |
| SOCS3 | 3.114699 | 6.937056 | 6.996045 | 1.11E-08 | 2.32E-06 | 7.826851 | UP |
| MXD1 | 1.939625 | 4.871957 | 6.914226 | 1.47E-08 | 2.82E-06 | 7.825847 | UP |
| C5AR1 | 1.676891 | 4.890515 | 6.916381 | 1.46E-08 | 2.80E-06 | 7.824796 | UP |
| NAMPT | 1.347712 | 8.495648 | 7.071739 | 8.61E-09 | 1.93E-06 | 7.817967 | UP |
| RND3 | 1.304451 | 6.097135 | 6.967116 | 1.23E-08 | 2.48E-06 | 7.812866 | UP |
| SERPINE1 | 4.377499 | 7.463233 | 6.996315 | 1.11E-08 | 2.32E-06 | 7.799789 | UP |
| BTBD19 | 1.58568 | 3.229618 | 6.830949 | 1.95E-08 | 3.54E-06 | 7.78755 | UP |
| KRT80 | 3.116024 | -1.21465 | 6.610221 | 4.12E-08 | 6.24E-06 | 7.782802 | UP |
| ODC1 | 2.180189 | 6.217951 | 6.95443 | 1.28E-08 | 2.56E-06 | 7.76624 | UP |
| NFATC1 | 1.184769 | 2.509875 | 6.78265 | 2.29E-08 | 3.96E-06 | 7.732117 | UP |
| MIR146B | 4.80364 | -1.9954 | 6.615209 | 4.05E-08 | 6.19E-06 | 7.729837 | UP |
| NEURL3 | 2.418266 | -0.43536 | 6.627046 | 3.89E-08 | 5.97E-06 | 7.723711 | UP |
| MIR29A | 2.193547 | -4.68372 | 6.667619 | 3.39E-08 | 5.29E-06 | 7.716494 | UP |
| DCSTAMP | 3.036409 | -3.3392 | 6.611744 | 4.10E-08 | 6.24E-06 | 7.688872 | UP |
| TACSTD2 | 2.178752 | 1.490317 | 6.702685 | 3.01E-08 | 4.90E-06 | 7.660635 | UP |
| MB21D1 | 1.527668 | 0.208638 | 6.639366 | 3.73E-08 | 5.74E-06 | 7.644611 | UP |
| ODF3L1 | 3.930871 | -1.48704 | 6.562394 | 4.85E-08 | 7.06E-06 | 7.635591 | UP |
| HSPB1P2 | 2.190394 | -4.24104 | 6.598604 | 4.29E-08 | 6.39E-06 | 7.629905 | UP |
| TLE3 | 1.892808 | 5.159588 | 6.853598 | 1.80E-08 | 3.31E-06 | 7.575468 | UP |
| RP11-49O14.2 | 2.791129 | -3.40253 | 6.539963 | 5.24E-08 | 7.44E-06 | 7.538146 | UP |
| RRP12 | 1.523825 | 3.961339 | 6.784095 | 2.28E-08 | 3.96E-06 | 7.512884 | UP |
| KLF6 | 2.127099 | 7.83857 | 6.943311 | 1.33E-08 | 2.62E-06 | 7.491273 | UP |
| GCNT3 | 3.175593 | -2.43456 | 6.504581 | 5.90E-08 | 8.18E-06 | 7.480374 | UP |
| PPP1R15A | 2.166541 | 5.871943 | 6.852475 | 1.81E-08 | 3.31E-06 | 7.471813 | UP |
| FAM71A | 2.843432 | -4.368 | 6.561607 | 4.86E-08 | 7.06E-06 | 7.43279 | UP |
| IL4I1 | 2.188261 | -1.42109 | 6.486501 | 6.28E-08 | 8.52E-06 | 7.380861 | UP |
| IFRD1 | 1.280826 | 4.514163 | 6.759041 | 2.49E-08 | 4.21E-06 | 7.340088 | UP |
| STK17A | 1.51569 | 3.057021 | 6.689236 | 3.15E-08 | 5.01E-06 | 7.331366 | UP |
| TUBB6 | 1.460615 | 3.935932 | 6.725724 | 2.78E-08 | 4.61E-06 | 7.316953 | UP |
| KBTBD8 | 1.374838 | 0.484491 | 6.554703 | 4.98E-08 | 7.17E-06 | 7.308208 | UP |
| PIM1 | 2.429274 | 5.594026 | 6.788094 | 2.25E-08 | 3.92E-06 | 7.299301 | UP |
| KCNE4 | 2.431913 | 1.451493 | 6.589889 | 4.42E-08 | 6.55E-06 | 7.292207 | UP |
| STK40 | 1.042949 | 6.3081 | 6.820388 | 2.02E-08 | 3.63E-06 | 7.279534 | UP |
| THAP2 | 1.462425 | 1.757261 | 6.606073 | 4.18E-08 | 6.30E-06 | 7.265854 | UP |
| LRRC32 | 1.246253 | 5.054195 | 6.753905 | 2.53E-08 | 4.27E-06 | 7.241175 | UP |
| SOX7 | 1.571764 | 1.914426 | 6.605212 | 4.19E-08 | 6.30E-06 | 7.238354 | UP |
| PTPN1 | 1.045786 | 5.368712 | 6.761313 | 2.47E-08 | 4.21E-06 | 7.217303 | UP |
| CTC-444N24.11 | 1.129607 | 3.556592 | 6.676658 | 3.29E-08 | 5.15E-06 | 7.20355 | UP |
| RP11-519G16.3 | 2.695502 | -4.67312 | 6.510613 | 5.78E-08 | 8.03E-06 | 7.189869 | UP |
| KLHL6 | 1.429986 | 2.01678 | 6.561168 | 4.87E-08 | 7.06E-06 | 7.067501 | UP |
| IL1B | 3.457614 | 1.139829 | 6.494143 | 6.12E-08 | 8.38E-06 | 7.05999 | UP |
| ZNF267 | 1.007909 | 2.555703 | 6.585974 | 4.48E-08 | 6.61E-06 | 7.052994 | UP |
| FFAR3 | 2.553442 | -3.83761 | 6.413513 | 8.05E-08 | 1.03E-05 | 7.051759 | UP |
| CREM | 1.172999 | 3.810675 | 6.639689 | 3.73E-08 | 5.74E-06 | 7.038978 | UP |
| TFAP2C | 2.229031 | -4.24864 | 6.41926 | 7.89E-08 | 1.02E-05 | 7.018593 | UP |
| ARC | 3.950392 | -0.69499 | 6.387157 | 8.80E-08 | 1.10E-05 | 6.980205 | UP |
| RP11-437B10.1 | 1.565585 | -1.33583 | 6.369396 | 9.35E-08 | 1.16E-05 | 6.968281 | UP |
| HBEGF | 2.659055 | 2.359978 | 6.520182 | 5.60E-08 | 7.85E-06 | 6.911344 | UP |
| PLEK | 1.436033 | 3.805776 | 6.594089 | 4.36E-08 | 6.48E-06 | 6.888281 | UP |
| DLGAP1-AS2 | 2.456745 | -0.87716 | 6.359714 | 9.66E-08 | 1.18E-05 | 6.882889 | UP |
| CD300E | 2.766264 | 1.90591 | 6.486311 | 6.28E-08 | 8.52E-06 | 6.874516 | UP |
| MLF1 | 2.225634 | -0.85219 | 6.339903 | 1.03E-07 | 1.24E-05 | 6.808855 | UP |
| CH25H | 5.011713 | -0.44601 | 6.334868 | 1.05E-07 | 1.25E-05 | 6.794358 | UP |
| HNRNPA1P7 | 2.423231 | -4.23736 | 6.352021 | 9.92E-08 | 1.20E-05 | 6.781075 | UP |
| CDC42EP1 | 1.021842 | 6.742922 | 6.687757 | 3.17E-08 | 5.01E-06 | 6.763851 | UP |
| SOCS1 | 3.100985 | 2.609125 | 6.481588 | 6.38E-08 | 8.60E-06 | 6.751129 | UP |
| IER3 | 2.603073 | -0.03313 | 6.355436 | 9.81E-08 | 1.19E-05 | 6.742946 | UP |
| PMAIP1 | 2.709817 | -0.99937 | 6.310929 | 1.14E-07 | 1.32E-05 | 6.738596 | UP |
| KRT16P1 | 5.299548 | -1.96598 | 6.355261 | 9.81E-08 | 1.19E-05 | 6.730432 | UP |
| ANXA1 | 1.39064 | 3.996246 | 6.553239 | 5.00E-08 | 7.18E-06 | 6.719781 | UP |
| SERPINB1 | 1.355277 | 5.209167 | 6.606656 | 4.17E-08 | 6.30E-06 | 6.718039 | UP |
| SLCO4A1 | 1.598317 | 3.486763 | 6.528095 | 5.45E-08 | 7.71E-06 | 6.71583 | UP |
| INAFM1 | 1.026832 | 1.975876 | 6.453772 | 7.02E-08 | 9.30E-06 | 6.701443 | UP |
| CA13 | 1.175112 | 2.565841 | 6.479843 | 6.42E-08 | 8.63E-06 | 6.692934 | UP |
| ISG20 | 1.344196 | 2.928371 | 6.470489 | 6.63E-08 | 8.87E-06 | 6.60446 | UP |
| ETS2 | 1.362205 | 8.515559 | 6.715505 | 2.88E-08 | 4.74E-06 | 6.603381 | UP |
| CLDN4 | 2.327861 | 1.058014 | 6.365827 | 9.47E-08 | 1.16E-05 | 6.594541 | UP |
| ANKRD33 | 3.355541 | -3.13157 | 6.272879 | 1.30E-07 | 1.48E-05 | 6.588882 | UP |
| MMP1 | 4.09652 | -2.96359 | 6.323459 | 1.09E-07 | 1.28E-05 | 6.583325 | UP |
| RP11-248J18.2 | 1.828641 | -2.69586 | 6.210349 | 1.61E-07 | 1.74E-05 | 6.561658 | UP |
| CCL18 | 2.26012 | -4.17115 | 6.265871 | 1.33E-07 | 1.50E-05 | 6.521116 | UP |
| LIMK2 | 1.604105 | 4.434656 | 6.511203 | 5.77E-08 | 8.03E-06 | 6.513215 | UP |
| FEZF1-AS1 | 2.030419 | -4.37212 | 6.287301 | 1.24E-07 | 1.43E-05 | 6.502941 | UP |
| JUN | 3.181734 | 7.098699 | 6.610324 | 4.12E-08 | 6.24E-06 | 6.494247 | UP |
| GPR56 | 1.489646 | 3.080898 | 6.441489 | 7.32E-08 | 9.61E-06 | 6.484587 | UP |
| RFX2 | 2.070952 | 1.72995 | 6.36807 | 9.39E-08 | 1.16E-05 | 6.47782 | UP |
| HSP90AA1 | 1.282036 | 8.951543 | 6.697286 | 3.07E-08 | 4.95E-06 | 6.476561 | UP |
| KLF5 | 2.118753 | 2.366411 | 6.392807 | 8.64E-08 | 1.09E-05 | 6.456206 | UP |
| SLC35E4 | 1.50793 | -0.38356 | 6.258155 | 1.37E-07 | 1.53E-05 | 6.447002 | UP |
| LINC-PINT | 1.229275 | 1.558733 | 6.345262 | 1.02E-07 | 1.22E-05 | 6.409259 | UP |
| ELL2 | 1.106173 | 7.262614 | 6.600692 | 4.26E-08 | 6.37E-06 | 6.392561 | UP |
| SPRY4 | 1.135744 | 4.664672 | 6.483917 | 6.33E-08 | 8.57E-06 | 6.379496 | UP |
| C3orf52 | 3.45226 | -1.90733 | 6.182694 | 1.76E-07 | 1.86E-05 | 6.378079 | UP |
| HSPA4L | 1.247758 | 3.692982 | 6.429194 | 7.63E-08 | 9.91E-06 | 6.342431 | UP |
| HSPE1P26 | 2.397832 | -4.46883 | 6.237379 | 1.47E-07 | 1.61E-05 | 6.340394 | UP |
| FOXK2 | 1.065826 | 5.237015 | 6.493822 | 6.12E-08 | 8.38E-06 | 6.326756 | UP |
| C11orf96 | 2.957798 | 4.760853 | 6.459297 | 6.89E-08 | 9.17E-06 | 6.321084 | UP |
| SOX8 | 2.855039 | -2.56024 | 6.15258 | 1.95E-07 | 2.01E-05 | 6.309256 | UP |
| ZNF460 | 1.507024 | 1.194804 | 6.293851 | 1.21E-07 | 1.40E-05 | 6.303606 | UP |
| RP11-274H2.5 | 2.406983 | -0.78283 | 6.188234 | 1.73E-07 | 1.85E-05 | 6.288412 | UP |
| RNA5SP155 | 2.093538 | -4.66926 | 6.239209 | 1.46E-07 | 1.61E-05 | 6.284325 | UP |
| RP11-186B7.4 | 2.145131 | -3.80913 | 6.157726 | 1.92E-07 | 1.98E-05 | 6.276064 | UP |
| MAFA | 1.994354 | -4.24298 | 6.198574 | 1.67E-07 | 1.79E-05 | 6.266663 | UP |
| LINC00641 | 1.324939 | 2.247008 | 6.336037 | 1.05E-07 | 1.25E-05 | 6.260573 | UP |
| SAMSN1 | 1.648217 | 3.022831 | 6.370869 | 9.31E-08 | 1.15E-05 | 6.257188 | UP |
| TNFAIP6 | 2.64038 | -1.62468 | 6.144438 | 2.01E-07 | 2.06E-05 | 6.245469 | UP |
| TMC1 | 2.038045 | -4.17923 | 6.180441 | 1.78E-07 | 1.87E-05 | 6.21175 | UP |
| CD55 | 1.154234 | 4.858498 | 6.439084 | 7.38E-08 | 9.64E-06 | 6.19802 | UP |
| ADAMTS9 | 1.786421 | 3.602935 | 6.379363 | 9.04E-08 | 1.13E-05 | 6.196992 | UP |
| KIAA0040 | 1.37351 | 4.994956 | 6.437394 | 7.42E-08 | 9.68E-06 | 6.174356 | UP |
| IER5L | 2.544861 | 3.028057 | 6.337711 | 1.04E-07 | 1.24E-05 | 6.168527 | UP |
| LRRC8B | 1.538172 | 3.080996 | 6.344643 | 1.02E-07 | 1.22E-05 | 6.156857 | UP |
| NOD2 | 1.964198 | 0.784843 | 6.207758 | 1.62E-07 | 1.75E-05 | 6.092494 | UP |
| EGR3 | 3.572604 | 1.126504 | 6.20492 | 1.64E-07 | 1.76E-05 | 6.084656 | UP |
| NXT1 | 1.132321 | 2.151412 | 6.270898 | 1.31E-07 | 1.49E-05 | 6.051161 | UP |
| RRAD | 3.54139 | -0.19762 | 6.130017 | 2.11E-07 | 2.15E-05 | 6.032464 | UP |
| ARHGAP23 | 1.362784 | 1.189411 | 6.212133 | 1.60E-07 | 1.73E-05 | 6.024094 | UP |
| CXCL3 | 3.074081 | -0.21144 | 6.121019 | 2.18E-07 | 2.20E-05 | 5.992951 | UP |
| CD274 | 2.570239 | 2.269789 | 6.238051 | 1.46E-07 | 1.61E-05 | 5.962277 | UP |
| HIF1A | 1.41497 | 6.266765 | 6.427439 | 7.68E-08 | 9.94E-06 | 5.952799 | UP |
| CXCL1 | 2.170525 | 1.176961 | 6.182419 | 1.77E-07 | 1.86E-05 | 5.946333 | UP |
| TP53INP2 | 1.040044 | 6.189938 | 6.419979 | 7.87E-08 | 1.02E-05 | 5.934531 | UP |
| DUSP2 | 2.780135 | 0.555207 | 6.139434 | 2.04E-07 | 2.09E-05 | 5.923859 | UP |
| PPP1R18 | 1.13602 | 0.209664 | 6.130567 | 2.11E-07 | 2.15E-05 | 5.911966 | UP |
| EHD4 | 1.65285 | 5.123698 | 6.364286 | 9.52E-08 | 1.17E-05 | 5.911179 | UP |
| RP11-443B7.3 | 2.55392 | -4.19182 | 6.08053 | 2.50E-07 | 2.44E-05 | 5.866104 | UP |
| GBP1 | 1.273134 | 5.281535 | 6.359343 | 9.68E-08 | 1.18E-05 | 5.865254 | UP |
| PMEPA1 | 1.160372 | 2.046072 | 6.209712 | 1.61E-07 | 1.74E-05 | 5.862536 | UP |
| GDF15 | 2.369975 | 2.566992 | 6.221356 | 1.55E-07 | 1.69E-05 | 5.847327 | UP |
| AC009014.3 | 2.255901 | -4.42683 | 6.090399 | 2.42E-07 | 2.38E-05 | 5.83257 | UP |
| CTD-2033D15.1 | 3.279391 | -1.64583 | 6.01416 | 3.13E-07 | 2.94E-05 | 5.801195 | UP |
| RP11-806H10.4 | 2.633551 | -2.21408 | 5.988674 | 3.41E-07 | 3.14E-05 | 5.759468 | UP |
| HCAR3 | 3.21646 | -1.37657 | 6.004155 | 3.24E-07 | 3.00E-05 | 5.746989 | UP |
| RPS20P22 | 2.60357 | -4.3251 | 6.048905 | 2.78E-07 | 2.66E-05 | 5.737391 | UP |
| CCL4L1 | 2.495679 | -3.53039 | 5.997768 | 3.31E-07 | 3.05E-05 | 5.727628 | UP |
| GNA13 | 1.234406 | 5.495523 | 6.321807 | 1.10E-07 | 1.28E-05 | 5.705324 | UP |
| GPR132 | 1.487883 | 0.405728 | 6.065023 | 2.63E-07 | 2.54E-05 | 5.662957 | UP |
| SNORA80B | 2.116445 | -4.19892 | 6.004062 | 3.24E-07 | 3.00E-05 | 5.623841 | UP |
| PRR7 | 2.010004 | -1.09086 | 5.973352 | 3.59E-07 | 3.28E-05 | 5.598988 | UP |
| CREB5 | 1.634166 | 1.786818 | 6.114373 | 2.23E-07 | 2.24E-05 | 5.595169 | UP |
| MB21D2 | 1.846951 | -0.11578 | 6.00584 | 3.22E-07 | 3.00E-05 | 5.555718 | UP |
| KCNN4 | 2.625856 | -1.56193 | 5.939665 | 4.03E-07 | 3.57E-05 | 5.547979 | UP |
| FOSL1 | 5.373532 | 1.743553 | 6.045848 | 2.81E-07 | 2.68E-05 | 5.539971 | UP |
| LINC00473 | 2.076957 | -4.46974 | 6.008829 | 3.19E-07 | 2.98E-05 | 5.531654 | UP |
| B3GNT7 | 1.114038 | 1.556847 | 6.081516 | 2.49E-07 | 2.44E-05 | 5.512187 | UP |
| NRIP3 | 2.874264 | -2.08619 | 5.913319 | 4.41E-07 | 3.83E-05 | 5.496316 | UP |
| MIR155HG | 2.512693 | -2.34804 | 5.903742 | 4.55E-07 | 3.91E-05 | 5.485469 | UP |
| CGA | 2.606932 | -4.20174 | 5.965718 | 3.69E-07 | 3.33E-05 | 5.484291 | UP |
| BHLHE41 | 1.460172 | -0.77405 | 5.949416 | 3.90E-07 | 3.47E-05 | 5.464094 | UP |
| FEM1C | 1.138428 | 5.41871 | 6.244102 | 1.43E-07 | 1.59E-05 | 5.451609 | UP |
| FAM183DP | 2.194409 | -4.07423 | 5.929016 | 4.18E-07 | 3.67E-05 | 5.425862 | UP |
| TEAD4 | 1.700745 | 1.806443 | 6.060061 | 2.68E-07 | 2.58E-05 | 5.408804 | UP |
| LIPN | 2.710003 | -3.33366 | 5.895244 | 4.69E-07 | 4.01E-05 | 5.392841 | UP |
| KRT8P41 | 1.819982 | -4.27144 | 5.935371 | 4.09E-07 | 3.61E-05 | 5.382921 | UP |
| ICAM5 | 2.98244 | -0.20251 | 5.939449 | 4.03E-07 | 3.57E-05 | 5.37329 | UP |
| RP11-7K24.3 | 2.024157 | -4.65685 | 5.953404 | 3.85E-07 | 3.43E-05 | 5.300271 | UP |
| ZBTB21 | 1.556277 | 5.204147 | 6.185895 | 1.75E-07 | 1.85E-05 | 5.291728 | UP |
| KRT18 | 1.038653 | 7.603471 | 6.285801 | 1.24E-07 | 1.43E-05 | 5.270637 | UP |
| ZSWIM6 | 1.026825 | 3.973868 | 6.126053 | 2.14E-07 | 2.17E-05 | 5.266932 | UP |
| PLAU | 2.355768 | 1.068275 | 5.966128 | 3.68E-07 | 3.33E-05 | 5.237002 | UP |
| PDCL3P5 | 2.171875 | -4.14711 | 5.879593 | 4.94E-07 | 4.18E-05 | 5.227325 | UP |
| CTD-2033D15.3 | 2.341587 | -2.28872 | 5.811771 | 6.22E-07 | 5.04E-05 | 5.18035 | UP |
| HES4 | 1.634116 | 0.774454 | 5.935347 | 4.09E-07 | 3.61E-05 | 5.164528 | UP |
| PNP | 1.20539 | 5.87389 | 6.177381 | 1.80E-07 | 1.88E-05 | 5.158156 | UP |
| BACH1-IT2 | 1.964761 | -3.64462 | 5.809925 | 6.26E-07 | 5.07E-05 | 5.155008 | UP |
| ITGAX | 1.71806 | 4.05298 | 6.079186 | 2.51E-07 | 2.45E-05 | 5.106439 | UP |
| USP2 | 1.910376 | 4.3048 | 6.088358 | 2.43E-07 | 2.39E-05 | 5.102623 | UP |
| RP11-197M22.2 | 2.444656 | -4.10571 | 5.83379 | 5.77E-07 | 4.76E-05 | 5.088415 | UP |
| THBD | 1.519724 | 5.100088 | 6.11636 | 2.21E-07 | 2.23E-05 | 5.070783 | UP |
| MCAM | 1.213648 | 3.566998 | 6.033894 | 2.93E-07 | 2.77E-05 | 5.019227 | UP |
| DUOXA2 | 2.65102 | -3.99147 | 5.815796 | 6.13E-07 | 4.99E-05 | 5.005683 | UP |
| PROB1 | 1.037299 | 0.98034 | 5.90084 | 4.60E-07 | 3.95E-05 | 5.001622 | UP |
| CTD-3137H5.4 | 2.043938 | -3.84427 | 5.777907 | 6.97E-07 | 5.52E-05 | 4.989707 | UP |
| FJX1 | 2.340346 | 1.208011 | 5.895784 | 4.68E-07 | 4.01E-05 | 4.97555 | UP |
| GK-AS1 | 2.260946 | -3.67548 | 5.767511 | 7.22E-07 | 5.68E-05 | 4.964909 | UP |
| SOX4 | 1.686741 | 1.75463 | 5.922634 | 4.27E-07 | 3.74E-05 | 4.954511 | UP |
| HSP90AB3P | 1.325383 | -1.55506 | 5.764654 | 7.29E-07 | 5.71E-05 | 4.954273 | UP |
| TNFRSF1B | 1.261459 | 5.945376 | 6.101673 | 2.32E-07 | 2.31E-05 | 4.89156 | UP |
| MMP3 | 3.977186 | -3.19663 | 5.80268 | 6.41E-07 | 5.16E-05 | 4.866254 | UP |
| SNHG17 | 1.12194 | 2.954191 | 5.95386 | 3.84E-07 | 3.43E-05 | 4.844117 | UP |
| RP11-359E10.1 | 3.066186 | -2.23115 | 5.715932 | 8.60E-07 | 6.55E-05 | 4.828087 | UP |
| RGS2 | 1.83237 | 3.830492 | 5.979017 | 3.53E-07 | 3.23E-05 | 4.804515 | UP |
| FOSB | 5.209598 | 5.955031 | 6.032379 | 2.94E-07 | 2.78E-05 | 4.790301 | UP |
| RP11-206L10.1 | 2.465595 | -4.90256 | 5.820534 | 6.04E-07 | 4.93E-05 | 4.788977 | UP |
| AP001046.5 | 1.808742 | -0.71546 | 5.749674 | 7.67E-07 | 5.97E-05 | 4.786996 | UP |
| UNC13A | 2.796131 | -4.25755 | 5.758816 | 7.44E-07 | 5.81E-05 | 4.776046 | UP |
| FLRT3 | 1.283373 | 3.755221 | 5.965211 | 3.69E-07 | 3.33E-05 | 4.759282 | UP |
| KB-1410C5.5 | 2.617099 | -4.05422 | 5.722032 | 8.43E-07 | 6.45E-05 | 4.730172 | UP |
| RP11-462L8.1 | 3.309568 | -2.08548 | 5.689233 | 9.41E-07 | 7.03E-05 | 4.726186 | UP |
| PTGES | 2.532568 | -3.45777 | 5.695586 | 9.21E-07 | 6.92E-05 | 4.717483 | UP |
| IL15RA | 1.008573 | 4.020882 | 5.965551 | 3.69E-07 | 3.33E-05 | 4.715767 | UP |
| PNPLA5 | 3.415373 | -3.30415 | 5.724829 | 8.35E-07 | 6.41E-05 | 4.707816 | UP |
| RNF122 | 1.597196 | 2.326882 | 5.876572 | 4.99E-07 | 4.21E-05 | 4.696426 | UP |
| PTPRZ1 | 2.153976 | -4.16822 | 5.720273 | 8.48E-07 | 6.48E-05 | 4.679112 | UP |
| OSR1 | 4.302645 | -1.83116 | 5.695728 | 9.21E-07 | 6.92E-05 | 4.669383 | UP |
| RP1-102K2.8 | 2.816591 | -4.76916 | 5.769644 | 7.17E-07 | 5.66E-05 | 4.667914 | UP |
| RNF19A | 1.066291 | 5.521004 | 6.0161 | 3.11E-07 | 2.92E-05 | 4.662241 | UP |
| LMNB1 | 1.124505 | 2.735029 | 5.883956 | 4.87E-07 | 4.15E-05 | 4.643467 | UP |
| SYT5 | 2.548992 | -4.545 | 5.735279 | 8.06E-07 | 6.24E-05 | 4.62874 | UP |
| AC016831.7 | 2.678309 | -1.49532 | 5.667522 | 1.01E-06 | 7.41E-05 | 4.626596 | UP |
| ELF3 | 2.038674 | 5.608938 | 6.001887 | 3.26E-07 | 3.02E-05 | 4.616188 | UP |
| STC1 | 2.54392 | -0.21199 | 5.711505 | 8.73E-07 | 6.63E-05 | 4.597897 | UP |
| FILIP1L | 1.165755 | 3.845661 | 5.911596 | 4.43E-07 | 3.85E-05 | 4.56218 | UP |
| RP11-79H23.3 | 3.009979 | -0.43747 | 5.682713 | 9.62E-07 | 7.11E-05 | 4.54678 | UP |
| SLC8A3 | 2.517622 | -3.40926 | 5.635278 | 1.13E-06 | 8.07E-05 | 4.537375 | UP |
| MIR6728 | 2.455008 | -4.74244 | 5.716345 | 8.59E-07 | 6.55E-05 | 4.501954 | UP |
| HIVEP2 | 1.545344 | 4.22097 | 5.907618 | 4.49E-07 | 3.87E-05 | 4.497207 | UP |
| METRNL | 1.365837 | 1.017092 | 5.749249 | 7.68E-07 | 5.97E-05 | 4.489426 | UP |
| TUFT1 | 1.457179 | 2.486068 | 5.822566 | 5.99E-07 | 4.91E-05 | 4.483461 | UP |
| DUOXA1 | 2.247294 | -4.25409 | 5.664362 | 1.02E-06 | 7.46E-05 | 4.463324 | UP |
| TSPYL2 | 1.443375 | 4.779581 | 5.908323 | 4.48E-07 | 3.87E-05 | 4.413069 | UP |
| BMP8A | 2.473628 | -4.21341 | 5.635446 | 1.13E-06 | 8.07E-05 | 4.401993 | UP |
| TMEM217 | 2.525037 | -1.76893 | 5.590624 | 1.31E-06 | 9.10E-05 | 4.396575 | UP |
| GPR3 | 2.56512 | -1.61371 | 5.594972 | 1.29E-06 | 8.98E-05 | 4.396086 | UP |
| DUSP4 | 1.915205 | 0.585089 | 5.694612 | 9.24E-07 | 6.94E-05 | 4.394228 | UP |
| SNHG15 | 1.351254 | 2.234484 | 5.781693 | 6.88E-07 | 5.48E-05 | 4.386294 | UP |
| RP11-766F14.2 | 1.770857 | -4.5128 | 5.643854 | 1.10E-06 | 7.93E-05 | 4.354045 | UP |
| ATP1B1P1 | 1.818663 | -4.49967 | 5.640465 | 1.11E-06 | 8.00E-05 | 4.344415 | UP |
| KRT7 | 1.310039 | 2.772024 | 5.792418 | 6.64E-07 | 5.33E-05 | 4.332014 | UP |
| SNORD69 | 1.934325 | -1.75013 | 5.570692 | 1.40E-06 | 9.59E-05 | 4.330594 | UP |
| KRT19 | 1.760581 | 1.460431 | 5.7209 | 8.46E-07 | 6.47E-05 | 4.325982 | UP |
| GRHL1 | 1.039161 | 3.687962 | 5.831719 | 5.81E-07 | 4.78E-05 | 4.315092 | UP |
| RP11-84A19.4 | 2.110474 | -3.54962 | 5.554398 | 1.48E-06 | 0.0001 | 4.296711 | UP |
| SPSB1 | 1.810021 | 6.09557 | 5.924128 | 4.25E-07 | 3.73E-05 | 4.276857 | UP |
| AREG | 3.083968 | 0.064219 | 5.623187 | 1.18E-06 | 8.34E-05 | 4.27329 | UP |
| GPR84 | 3.098908 | -1.74415 | 5.538515 | 1.57E-06 | 0.000104 | 4.214083 | UP |
| ANKRD33B | 1.696245 | 0.498033 | 5.63572 | 1.13E-06 | 8.07E-05 | 4.205637 | UP |
| RP11-667F14.1 | 2.438139 | -4.17642 | 5.571148 | 1.40E-06 | 9.59E-05 | 4.194844 | UP |
| MAP1LC3B2 | 1.717728 | -1.0966 | 5.55004 | 1.51E-06 | 0.000101 | 4.174102 | UP |
| BACH1 | 1.0754 | 5.113282 | 5.841898 | 5.61E-07 | 4.64E-05 | 4.134235 | UP |
| ARRDC4 | 1.29148 | 4.568402 | 5.815196 | 6.15E-07 | 5.00E-05 | 4.128428 | UP |
| C6orf132 | 1.939605 | -1.22361 | 5.52248 | 1.65E-06 | 0.000108 | 4.103932 | UP |
| RP11-214O1.3 | 1.603378 | -1.03508 | 5.529879 | 1.61E-06 | 0.000106 | 4.097137 | UP |
| AGAP1-IT1 | 2.013884 | -3.07358 | 5.472818 | 1.95E-06 | 0.000124 | 4.077225 | UP |
| RP11-149I9.2 | 2.563185 | -4.11652 | 5.531797 | 1.60E-06 | 0.000106 | 4.070347 | UP |
| EID3 | 1.803262 | -2.21986 | 5.470405 | 1.97E-06 | 0.000125 | 4.044618 | UP |
| RP11-617F23.2 | 2.877852 | -4.48312 | 5.544454 | 1.53E-06 | 0.000102 | 3.995423 | UP |
| IFNGR1 | 1.000004 | 6.251199 | 5.85031 | 5.46E-07 | 4.54E-05 | 3.992789 | UP |
| VCAN | 1.74772 | 3.688863 | 5.72991 | 8.20E-07 | 6.33E-05 | 3.98432 | UP |
| HSPA6 | 4.579329 | 1.856292 | 5.59779 | 1.28E-06 | 8.93E-05 | 3.967923 | UP |
| TMEM2 | 1.125621 | 6.73517 | 5.853252 | 5.40E-07 | 4.51E-05 | 3.933134 | UP |
| RGS16 | 2.554259 | 1.986571 | 5.621952 | 1.18E-06 | 8.36E-05 | 3.931053 | UP |
| CTD-3203P2.3 | 2.140761 | -3.2352 | 5.430006 | 2.26E-06 | 0.000139 | 3.913305 | UP |
| TSLP | 1.354722 | 1.976932 | 5.626228 | 1.16E-06 | 8.27E-05 | 3.908309 | UP |
| EGR2 | 3.891118 | 1.823452 | 5.585367 | 1.34E-06 | 9.24E-05 | 3.901502 | UP |
| CTD-2262B20.1 | 2.762795 | -4.63562 | 5.529164 | 1.62E-06 | 0.000106 | 3.88452 | UP |
| ZNF674-AS1 | 1.182507 | 1.257113 | 5.571355 | 1.40E-06 | 9.59E-05 | 3.847596 | UP |
| SLC7A1 | 2.593073 | 2.350968 | 5.609237 | 1.23E-06 | 8.65E-05 | 3.826665 | UP |
| RP11-589N15.2 | 1.247881 | 0.151542 | 5.504695 | 1.75E-06 | 0.000114 | 3.818021 | UP |
| REL | 1.355243 | 2.966177 | 5.642045 | 1.10E-06 | 7.97E-05 | 3.79644 | UP |
| AGPAT9 | 1.722085 | 3.069428 | 5.644423 | 1.10E-06 | 7.93E-05 | 3.79539 | UP |
| BCL3 | 1.32332 | 6.663262 | 5.808284 | 6.29E-07 | 5.08E-05 | 3.793903 | UP |
| GPRC5A | 2.893866 | 0.435535 | 5.495942 | 1.81E-06 | 0.000116 | 3.780013 | UP |
| SERPINA3 | 1.70322 | 7.109532 | 5.819301 | 6.06E-07 | 4.95E-05 | 3.771058 | UP |
| HMGA1 | 1.253753 | 4.138515 | 5.687121 | 9.48E-07 | 7.05E-05 | 3.761722 | UP |
| HES2 | 2.170973 | -3.11423 | 5.387243 | 2.60E-06 | 0.000155 | 3.744687 | UP |
| EVA1C | 1.123036 | 1.779184 | 5.566023 | 1.43E-06 | 9.71E-05 | 3.736265 | UP |
| RPL29P14 | 1.907022 | -4.66692 | 5.475542 | 1.94E-06 | 0.000123 | 3.7294 | UP |
| RP11-2B6.2 | 1.014207 | 0.691192 | 5.501161 | 1.78E-06 | 0.000115 | 3.710063 | UP |
| MAK | 2.241963 | -1.66536 | 5.364986 | 2.81E-06 | 0.000165 | 3.636322 | UP |
| EMILIN2 | 1.180655 | 2.463739 | 5.569456 | 1.41E-06 | 9.61E-05 | 3.63085 | UP |
| IFI30 | 1.558243 | -0.46042 | 5.405136 | 2.45E-06 | 0.000148 | 3.591207 | UP |
| JAK3 | 1.144828 | 2.770227 | 5.554675 | 1.48E-06 | 0.0001 | 3.530468 | UP |
| AC002511.3 | 1.916852 | -4.19154 | 5.38159 | 2.65E-06 | 0.000158 | 3.525031 | UP |
| RCC2P6 | 2.51127 | -4.55623 | 5.407951 | 2.43E-06 | 0.000147 | 3.523349 | UP |
| RP11-274H2.3 | 2.040402 | -2.76773 | 5.305412 | 3.43E-06 | 0.000194 | 3.522136 | UP |
| RP11-670E13.6 | 2.53943 | -1.74564 | 5.31282 | 3.34E-06 | 0.00019 | 3.470913 | UP |
| IFNG | 1.988081 | -4.16645 | 5.35769 | 2.88E-06 | 0.000167 | 3.46541 | UP |
| NABP1 | 1.452818 | 4.655164 | 5.612397 | 1.22E-06 | 8.59E-05 | 3.43536 | UP |
| OSM | 3.201424 | -1.00894 | 5.319219 | 3.27E-06 | 0.000188 | 3.415876 | UP |
| SBK3 | 2.452152 | -3.50991 | 5.311223 | 3.36E-06 | 0.000191 | 3.412928 | UP |
| IL4R | 1.050551 | 7.007352 | 5.697007 | 9.17E-07 | 6.91E-05 | 3.365757 | UP |
| PTGER2 | 1.18644 | 0.975535 | 5.411618 | 2.40E-06 | 0.000146 | 3.364416 | UP |
| MFI2 | 1.423244 | 0.875159 | 5.404719 | 2.46E-06 | 0.000148 | 3.363617 | UP |
| KLF4 | 1.742074 | 4.560243 | 5.584839 | 1.34E-06 | 9.24E-05 | 3.362843 | UP |
| HIVEP3 | 1.185317 | 2.630896 | 5.488134 | 1.86E-06 | 0.000119 | 3.332312 | UP |
| NCBP2-AS1 | 1.916569 | -4.73475 | 5.357684 | 2.88E-06 | 0.000167 | 3.315125 | UP |
| ADAMTSL4-AS1 | 1.787287 | 0.850959 | 5.384604 | 2.63E-06 | 0.000156 | 3.309385 | UP |
| GRAMD4 | 1.904991 | 6.948653 | 5.673738 | 9.92E-07 | 7.31E-05 | 3.308758 | UP |
| IRF4 | 3.719986 | 2.459788 | 5.442089 | 2.17E-06 | 0.000134 | 3.302643 | UP |
| NR4A3 | 4.09397 | 2.206618 | 5.407985 | 2.43E-06 | 0.000147 | 3.253368 | UP |
| ESM1 | 2.422872 | -2.61142 | 5.227696 | 4.45E-06 | 0.00024 | 3.241775 | UP |
| ARG2 | 1.851729 | 1.747559 | 5.405167 | 2.45E-06 | 0.000148 | 3.222456 | UP |
| ARNTL2 | 1.624769 | 2.859063 | 5.463715 | 2.01E-06 | 0.000127 | 3.222128 | UP |
| SPATA6L | 1.372559 | 2.386097 | 5.437278 | 2.20E-06 | 0.000136 | 3.206935 | UP |
| BHLHE40 | 1.816015 | 7.714144 | 5.667366 | 1.01E-06 | 7.41E-05 | 3.173006 | UP |
| LINC01220 | 2.128312 | -4.05921 | 5.255244 | 4.05E-06 | 0.000222 | 3.157705 | UP |
| SGMS2 | 2.251188 | 5.163716 | 5.542183 | 1.55E-06 | 0.000103 | 3.139908 | UP |
| SERPINB2 | 2.687267 | -3.84198 | 5.248845 | 4.14E-06 | 0.000226 | 3.12909 | UP |
| RP11-253E3.3 | 1.532578 | 0.230456 | 5.30014 | 3.49E-06 | 0.000197 | 3.128537 | UP |
| AP001615.9 | 2.653028 | -2.21266 | 5.197627 | 4.91E-06 | 0.00026 | 3.118052 | UP |
| ITGB8 | 1.511376 | 0.261012 | 5.285748 | 3.66E-06 | 0.000205 | 3.075918 | UP |
| CTRC | 1.901441 | -4.51728 | 5.276108 | 3.78E-06 | 0.00021 | 3.07063 | UP |
| C6orf141 | 2.694936 | 0.647095 | 5.294224 | 3.56E-06 | 0.0002 | 3.070087 | UP |
| SNHG4 | 1.793168 | -1.7182 | 5.188583 | 5.07E-06 | 0.000266 | 3.059772 | UP |
| KB-1471A8.1 | 2.100839 | -4.28365 | 5.227481 | 4.45E-06 | 0.00024 | 3.048821 | UP |
| NAMPTP1 | 1.42996 | 3.841041 | 5.458844 | 2.05E-06 | 0.000129 | 3.045639 | UP |
| RP11-631M6.3 | 2.309505 | -4.18856 | 5.233202 | 4.36E-06 | 0.000237 | 3.042284 | UP |
| RP4-647J21.1 | 2.230996 | -2.46154 | 5.163241 | 5.51E-06 | 0.000285 | 3.038682 | UP |
| GCNT4 | 1.833794 | 0.664286 | 5.290526 | 3.60E-06 | 0.000203 | 3.029395 | UP |
| FCAR | 2.247663 | -1.99133 | 5.163043 | 5.52E-06 | 0.000285 | 3.001911 | UP |
| SLC22A4 | 1.643987 | -0.66747 | 5.215372 | 4.63E-06 | 0.000247 | 2.997462 | UP |
| SPRR2A | 2.144373 | -5.03499 | 5.305065 | 3.43E-06 | 0.000194 | 2.992684 | UP |
| RP5-1182A14.5 | 2.50797 | -4.35601 | 5.222487 | 4.52E-06 | 0.000243 | 2.984913 | UP |
| LL22NC03-102D1.18 | 2.136715 | -3.81441 | 5.169498 | 5.40E-06 | 0.00028 | 2.981169 | UP |
| TNFSF9 | 2.949506 | -2.29836 | 5.154735 | 5.67E-06 | 0.000291 | 2.969209 | UP |
| GADD45B | 1.598199 | 7.953243 | 5.615998 | 1.21E-06 | 8.51E-05 | 2.961986 | UP |
| RP11-290L1.3 | 2.729983 | -0.24363 | 5.214655 | 4.64E-06 | 0.000248 | 2.953021 | UP |
| RN7SL368P | 1.71567 | -4.19657 | 5.203346 | 4.82E-06 | 0.000255 | 2.951288 | UP |
| PPARD | 1.326621 | 5.567426 | 5.50344 | 1.76E-06 | 0.000114 | 2.932584 | UP |
| USP12 | 1.016755 | 4.735849 | 5.461778 | 2.03E-06 | 0.000128 | 2.913939 | UP |
| KRT8P45 | 1.64428 | -3.37155 | 5.105106 | 6.69E-06 | 0.000331 | 2.875749 | UP |
| SLC19A2 | 1.200649 | 5.01483 | 5.455163 | 2.07E-06 | 0.00013 | 2.852355 | UP |
| EMR2 | 1.084384 | 3.132701 | 5.348273 | 2.97E-06 | 0.000172 | 2.782854 | UP |
| MSC | 1.258259 | 0.774125 | 5.222904 | 4.52E-06 | 0.000243 | 2.774583 | UP |
| FRMD6 | 1.49975 | 3.005735 | 5.335033 | 3.10E-06 | 0.000179 | 2.767135 | UP |
| AC144831.1 | 1.455397 | 0.06201 | 5.178096 | 5.25E-06 | 0.000274 | 2.752873 | UP |
| RP11-803P9.1 | 1.725855 | -4.39866 | 5.150212 | 5.76E-06 | 0.000294 | 2.748774 | UP |
| FOXC2 | 1.96164 | -3.73743 | 5.092753 | 6.97E-06 | 0.000339 | 2.745437 | UP |
| CIRBP-AS1 | 2.046094 | -0.22663 | 5.146679 | 5.83E-06 | 0.000296 | 2.706534 | UP |
| LINC00152 | 2.354505 | -1.05879 | 5.099704 | 6.81E-06 | 0.000335 | 2.687109 | UP |
| RP11-67L3.4 | 2.318038 | -4.00579 | 5.098816 | 6.83E-06 | 0.000335 | 2.684479 | UP |
| C15orf48 | 2.142542 | -1.446 | 5.082899 | 7.20E-06 | 0.000348 | 2.680978 | UP |
| NPTX2 | 2.544552 | 0.528362 | 5.169539 | 5.40E-06 | 0.00028 | 2.66998 | UP |
| ISM1-AS1 | 1.979082 | -4.05726 | 5.097432 | 6.86E-06 | 0.000336 | 2.665756 | UP |
| RP11-672A2.4 | 1.515923 | -1.40703 | 5.081292 | 7.24E-06 | 0.00035 | 2.665753 | UP |
| CDCA4P4 | 2.806648 | -4.18403 | 5.115197 | 6.47E-06 | 0.000322 | 2.664408 | UP |
| RP11-463O12.5 | 1.335583 | 1.260356 | 5.21045 | 4.71E-06 | 0.000251 | 2.651436 | UP |
| RP11-680F20.12 | 1.654927 | -4.74082 | 5.154944 | 5.67E-06 | 0.000291 | 2.642328 | UP |
| RP4-539M6.22 | 1.993796 | -4.06199 | 5.087377 | 7.10E-06 | 0.000344 | 2.626848 | UP |
| CYR61 | 2.502178 | 5.676813 | 5.400723 | 2.49E-06 | 0.00015 | 2.598555 | UP |
| RP3-508I15.21 | 1.853923 | -1.35258 | 5.058668 | 7.81E-06 | 0.00037 | 2.587456 | UP |
| TRPV6 | 1.932051 | -1.60179 | 5.047859 | 8.09E-06 | 0.000381 | 2.584604 | UP |
| PAX9 | 1.710671 | -4.97707 | 5.169033 | 5.41E-06 | 0.00028 | 2.576614 | UP |
| CCL7 | 3.04412 | -3.70215 | 5.099026 | 6.83E-06 | 0.000335 | 2.574342 | UP |
| HSPA8P5 | 1.486087 | -3.1358 | 5.00372 | 9.37E-06 | 0.000426 | 2.555975 | UP |
| BLZF2P | 1.795526 | -4.7686 | 5.125308 | 6.25E-06 | 0.000314 | 2.533201 | UP |
| SOCS2 | 1.449451 | 4.969638 | 5.350231 | 2.95E-06 | 0.000171 | 2.512487 | UP |
| CSRNP1 | 1.840716 | 6.459531 | 5.411624 | 2.40E-06 | 0.000146 | 2.502232 | UP |
| C2CD4A | 4.656028 | -0.17888 | 5.048292 | 8.08E-06 | 0.000381 | 2.456044 | UP |
| SMOX | 1.880757 | 2.301293 | 5.190545 | 5.03E-06 | 0.000264 | 2.417212 | UP |
| IER2 | 1.648954 | 6.406235 | 5.38196 | 2.65E-06 | 0.000158 | 2.407859 | UP |
| HSPB1 | 1.474763 | 7.055469 | 5.41135 | 2.40E-06 | 0.000146 | 2.407187 | UP |
| RP11-212D19.4 | 1.597873 | -4.76785 | 5.079552 | 7.28E-06 | 0.00035 | 2.392601 | UP |
| HNRNPUP1 | 2.056386 | -4.36545 | 5.028922 | 8.62E-06 | 0.0004 | 2.384809 | UP |
| KIAA0226L | 1.73462 | 0.479725 | 5.081058 | 7.25E-06 | 0.00035 | 2.36786 | UP |
| RP11-400N13.3 | 2.457595 | -4.41397 | 5.033087 | 8.50E-06 | 0.000396 | 2.364027 | UP |
| ARL4C | 1.022957 | 3.525469 | 5.220132 | 4.56E-06 | 0.000245 | 2.296022 | UP |
| RASD1 | 2.773458 | 5.841824 | 5.314698 | 3.32E-06 | 0.00019 | 2.294441 | UP |
| NR1D1 | 1.71264 | 5.671911 | 5.308923 | 3.39E-06 | 0.000192 | 2.274584 | UP |
| SLC7A5P1 | 2.252244 | -4.24252 | 4.982278 | 1.01E-05 | 0.00045 | 2.249115 | UP |
| PTPRE | 1.162541 | 3.468273 | 5.201813 | 4.85E-06 | 0.000256 | 2.246484 | UP |
| HTRA4 | 1.778562 | -3.88634 | 4.946997 | 1.13E-05 | 0.000496 | 2.234116 | UP |
| RPRD1B | 1.539407 | 6.260264 | 5.318992 | 3.28E-06 | 0.000188 | 2.217925 | UP |
| RP11-779O18.2 | 2.414681 | -3.63133 | 4.937055 | 1.17E-05 | 0.000509 | 2.210033 | UP |
| NOS3 | 1.492179 | 4.021028 | 5.209711 | 4.72E-06 | 0.000251 | 2.191009 | UP |
| TNFAIP8 | 1.120174 | 2.227267 | 5.12337 | 6.30E-06 | 0.000315 | 2.189367 | UP |
| RP1-167G20.1 | 1.875326 | -5.04326 | 5.063687 | 7.68E-06 | 0.000365 | 2.182679 | UP |
| PDLIM4 | 2.288825 | -1.81923 | 4.913563 | 1.26E-05 | 0.000541 | 2.171177 | UP |
| MIRLET7I | 1.667346 | -4.72301 | 5.00559 | 9.31E-06 | 0.000424 | 2.163369 | UP |
| FAM222A | 1.194152 | 1.984013 | 5.098335 | 6.84E-06 | 0.000335 | 2.149893 | UP |
| RP11-54A4.2 | 1.879406 | -0.17008 | 4.975638 | 1.03E-05 | 0.000458 | 2.13597 | UP |
| RP11-276H7.3 | 2.306219 | -2.72173 | 4.872553 | 1.44E-05 | 0.000603 | 2.091367 | UP |
| RP11-320G24.1 | 1.655934 | -4.87427 | 5.003225 | 9.38E-06 | 0.000426 | 2.088471 | UP |
| ACTRT3 | 1.37617 | 1.561844 | 5.05548 | 7.89E-06 | 0.000373 | 2.087205 | UP |
| SOD2 | 1.538047 | 9.699297 | 5.421983 | 2.32E-06 | 0.000142 | 2.055961 | UP |
| TRIM15 | 1.931278 | -0.26292 | 4.942257 | 1.15E-05 | 0.000501 | 2.043318 | UP |
| MUC13 | 2.550814 | -1.19005 | 4.895546 | 1.34E-05 | 0.000567 | 2.041323 | UP |
| RP11-45M22.2 | 1.164225 | 0.527485 | 4.982172 | 1.01E-05 | 0.00045 | 2.026971 | UP |
| HIST1H2AD | 2.249358 | -2.20316 | 4.846089 | 1.58E-05 | 0.000645 | 1.984298 | UP |
| RP3-453C12.14 | 2.539638 | -0.96126 | 4.886061 | 1.38E-05 | 0.00058 | 1.981011 | UP |
| ETV7 | 2.480936 | -0.43891 | 4.908352 | 1.28E-05 | 0.00055 | 1.973184 | UP |
| MIR320A | 1.761153 | -4.76404 | 4.955299 | 1.10E-05 | 0.000485 | 1.970717 | UP |
| FAM43A | 1.18294 | 2.976108 | 5.085983 | 7.13E-06 | 0.000345 | 1.944144 | UP |
| C1orf106 | 1.705173 | -1.5705 | 4.846006 | 1.58E-05 | 0.000645 | 1.924133 | UP |
| ULBP2 | 1.773421 | -2.21249 | 4.82062 | 1.71E-05 | 0.000689 | 1.913415 | UP |
| HIST1H2BG | 2.279296 | -2.26514 | 4.818757 | 1.72E-05 | 0.000691 | 1.900325 | UP |
| CD3EAP | 1.380283 | 2.4974 | 5.047716 | 8.10E-06 | 0.000381 | 1.900314 | UP |
| RP11-697E2.6 | 2.168975 | -4.08673 | 4.866593 | 1.47E-05 | 0.000613 | 1.889524 | UP |
| LUCAT1 | 2.857266 | -1.14664 | 4.841974 | 1.60E-05 | 0.000653 | 1.866999 | UP |
| LITAF | 1.055601 | 6.588554 | 5.226944 | 4.46E-06 | 0.00024 | 1.858535 | UP |
| HAS3 | 1.291218 | 0.619749 | 4.930048 | 1.20E-05 | 0.000517 | 1.842165 | UP |
| RP11-631M6.2 | 2.820181 | -4.80695 | 4.915836 | 1.25E-05 | 0.000538 | 1.836332 | UP |
| RP11-652L8.4 | 2.546477 | -4.14589 | 4.850258 | 1.55E-05 | 0.000639 | 1.835376 | UP |
| SOWAHC | 1.150719 | 6.045807 | 5.188141 | 5.07E-06 | 0.000266 | 1.811738 | UP |
| CCL4 | 2.651037 | -1.62019 | 4.798236 | 1.84E-05 | 0.000727 | 1.776774 | UP |
| APOLD1 | 2.078636 | 3.434825 | 5.051716 | 7.99E-06 | 0.000377 | 1.77657 | UP |
| CCRN4L | 2.067598 | 4.21452 | 5.069973 | 7.52E-06 | 0.00036 | 1.714737 | UP |
| RNU6-570P | 2.226505 | -4.92845 | 4.891618 | 1.36E-05 | 0.000573 | 1.695051 | UP |
| RP11-62H20.1 | 1.956471 | -4.18969 | 4.80582 | 1.80E-05 | 0.000712 | 1.686877 | UP |
| RP11-613D13.8 | 2.494575 | -0.97883 | 4.789042 | 1.90E-05 | 0.000744 | 1.668998 | UP |
| INPP5J | 1.623222 | -2.35373 | 4.728747 | 2.32E-05 | 0.000867 | 1.633243 | UP |
| NFIL3 | 1.319883 | 6.258712 | 5.140943 | 5.94E-06 | 0.000301 | 1.626611 | UP |
| GK-IT1 | 1.771805 | -3.31204 | 4.73328 | 2.28E-05 | 0.00086 | 1.617758 | UP |
| RP11-465B22.3 | 1.942412 | -1.94364 | 4.735958 | 2.26E-05 | 0.000853 | 1.613133 | UP |
| ZFAND2A | 1.070242 | 4.407028 | 5.04758 | 8.10E-06 | 0.000381 | 1.593095 | UP |
| ZNF469 | 1.238485 | 1.863522 | 4.91833 | 1.24E-05 | 0.000534 | 1.584975 | UP |
| RP11-317F20.2 | 1.747837 | -4.96749 | 4.862956 | 1.49E-05 | 0.000619 | 1.576881 | UP |
| CCL11 | 1.165851 | -4.50778 | 4.830844 | 1.66E-05 | 0.000673 | 1.575503 | UP |
| TREM1 | 1.999261 | 0.977139 | 4.858336 | 1.51E-05 | 0.000626 | 1.563472 | UP |
| RP11-144L1.8 | 1.419143 | -2.99616 | 4.690672 | 2.62E-05 | 0.000959 | 1.553434 | UP |
| ANKRD44-IT1 | 1.589552 | -4.77046 | 4.82227 | 1.70E-05 | 0.000687 | 1.552302 | UP |
| FGF18 | 2.035129 | -4.27781 | 4.768797 | 2.03E-05 | 0.000782 | 1.547284 | UP |
| PTGES3P1 | 1.153088 | -0.64275 | 4.773443 | 2.00E-05 | 0.000774 | 1.546323 | UP |
| EFNB2 | 1.061473 | 3.948051 | 5.006966 | 9.27E-06 | 0.000422 | 1.530116 | UP |
| RP11-557J10.4 | 2.255399 | -4.27912 | 4.757309 | 2.11E-05 | 0.000806 | 1.522404 | UP |
| BCL6B | 1.243262 | 2.501289 | 4.932416 | 1.19E-05 | 0.000514 | 1.520212 | UP |
| RP1-34L19.1 | 2.042273 | -4.94195 | 4.838326 | 1.62E-05 | 0.000659 | 1.514597 | UP |
| RP1-86C11.7 | 2.191639 | -4.49272 | 4.766437 | 2.05E-05 | 0.000787 | 1.495569 | UP |
| RIN1 | 1.40032 | 0.586282 | 4.820364 | 1.72E-05 | 0.000689 | 1.494685 | UP |
| RP11-314N13.10 | 1.771387 | -2.69505 | 4.669687 | 2.81E-05 | 0.001016 | 1.463731 | UP |
| CTD-2033D15.2 | 2.196113 | -1.98893 | 4.683894 | 2.68E-05 | 0.000977 | 1.448153 | UP |
| ACHE | 1.329382 | 0.534428 | 4.796217 | 1.86E-05 | 0.00073 | 1.42582 | UP |
| CCL3 | 2.725932 | -1.38669 | 4.693282 | 2.60E-05 | 0.000954 | 1.415624 | UP |
| DNAJB4 | 1.764456 | 4.199456 | 4.97671 | 1.02E-05 | 0.000457 | 1.404328 | UP |
| LGALS9C | 2.870283 | -4.2564 | 4.719691 | 2.39E-05 | 0.000889 | 1.401938 | UP |
| LINC00346 | 1.515323 | -0.9896 | 4.707471 | 2.48E-05 | 0.000921 | 1.393729 | UP |
| GZMB | 2.089705 | -1.10234 | 4.694428 | 2.59E-05 | 0.000951 | 1.375628 | UP |
| UBE2FP1 | 1.10938 | -0.38063 | 4.732657 | 2.29E-05 | 0.00086 | 1.372781 | UP |
| SYTL3 | 1.279337 | 0.778632 | 4.78333 | 1.94E-05 | 0.000757 | 1.339769 | UP |
| EPS8L3 | 2.666571 | -4.06805 | 4.691437 | 2.62E-05 | 0.000959 | 1.331391 | UP |
| MYO1G | 1.266012 | 2.020707 | 4.84614 | 1.58E-05 | 0.000645 | 1.32261 | UP |
| MYBPH | 3.138806 | -2.89862 | 4.657547 | 2.92E-05 | 0.001047 | 1.318533 | UP |
| RP11-268P4.4 | 1.706773 | -4.82005 | 4.752909 | 2.14E-05 | 0.000816 | 1.300995 | UP |
| SH3RF3-AS1 | 1.964155 | -2.91327 | 4.618539 | 3.32E-05 | 0.001157 | 1.296668 | UP |
| HMGN2P46 | 1.909204 | -4.22436 | 4.691025 | 2.62E-05 | 0.000959 | 1.291541 | UP |
| INHBB | 1.401079 | 3.385159 | 4.900939 | 1.32E-05 | 0.000559 | 1.27757 | UP |
| RP1-239B22.5 | 1.759683 | -0.94131 | 4.669645 | 2.81E-05 | 0.001016 | 1.268952 | UP |
| KCNK1 | 2.057632 | 2.312571 | 4.83847 | 1.62E-05 | 0.000659 | 1.268693 | UP |
| MBOAT4 | 2.202951 | -3.04785 | 4.6122 | 3.39E-05 | 0.001177 | 1.260264 | UP |
| RPA4 | 1.907352 | -4.06652 | 4.662089 | 2.88E-05 | 0.001036 | 1.238364 | UP |
| NAT16 | 1.705405 | -4.76507 | 4.719732 | 2.39E-05 | 0.000889 | 1.223447 | UP |
| ARHGAP31-AS1 | 1.450065 | -5.06471 | 4.77541 | 1.99E-05 | 0.000771 | 1.214057 | UP |
| RP11-532F12.5 | 1.539077 | -4.26561 | 4.651564 | 2.98E-05 | 0.001062 | 1.199652 | UP |
| FCAMR | 2.211067 | 0.911333 | 4.73916 | 2.24E-05 | 0.000845 | 1.196081 | UP |
| RP11-325F22.2 | 1.915768 | -2.96516 | 4.582067 | 3.73E-05 | 0.001269 | 1.184348 | UP |
| RP11-373D23.3 | 1.966843 | -3.2979 | 4.57995 | 3.76E-05 | 0.001275 | 1.155833 | UP |
| RP1-34B20.4 | 2.511005 | -4.19098 | 4.634823 | 3.15E-05 | 0.001109 | 1.141292 | UP |
| CD68 | 1.588442 | -1.54434 | 4.601928 | 3.50E-05 | 0.001205 | 1.137766 | UP |
| LYPD1 | 1.59475 | -1.1911 | 4.6121 | 3.39E-05 | 0.001177 | 1.119912 | UP |
| ADRA1D | 2.431176 | -3.66319 | 4.592138 | 3.61E-05 | 0.001235 | 1.094076 | UP |
| LENG8-AS1 | 1.588295 | -5.10847 | 4.743738 | 2.20E-05 | 0.000834 | 1.090726 | UP |
| FAM107A | 1.222094 | 2.922401 | 4.820366 | 1.72E-05 | 0.000689 | 1.087435 | UP |
| AIRE | 1.51015 | -5.11338 | 4.744064 | 2.20E-05 | 0.000833 | 1.086574 | UP |
| TMEM171 | 1.482043 | 0.700299 | 4.694658 | 2.59E-05 | 0.000951 | 1.071249 | UP |
| PDE4B | 1.330587 | 3.056939 | 4.819546 | 1.72E-05 | 0.00069 | 1.06502 | UP |
| EGOT | 1.999525 | -4.55499 | 4.645055 | 3.04E-05 | 0.00108 | 1.064485 | UP |
| RP11-271C24.3 | 1.561874 | -4.59151 | 4.638332 | 3.11E-05 | 0.0011 | 1.053663 | UP |
| AC008440.5 | 1.615498 | -1.44081 | 4.576775 | 3.80E-05 | 0.001285 | 1.044459 | UP |
| ELOVL7 | 1.17021 | -0.21888 | 4.632712 | 3.17E-05 | 0.001115 | 1.02709 | UP |
| RP11-444D3.1 | 1.196253 | -0.7785 | 4.593872 | 3.59E-05 | 0.001229 | 0.995298 | UP |
| RP11-265O12.1 | 1.217278 | -5.08488 | 4.707896 | 2.48E-05 | 0.00092 | 0.986513 | UP |
| TNIP3 | 3.268124 | -1.81359 | 4.543647 | 4.23E-05 | 0.001396 | 0.969344 | UP |
| PNRC1 | 1.127251 | 7.37498 | 4.989104 | 9.83E-06 | 0.000443 | 0.961885 | UP |
| ACTBP11 | 1.503262 | -2.56294 | 4.50779 | 4.75E-05 | 0.001529 | 0.950204 | UP |
| SNORA77 | 1.983475 | -3.91814 | 4.548293 | 4.16E-05 | 0.001383 | 0.940947 | UP |
| FAM167A | 2.097585 | -0.80623 | 4.562047 | 3.98E-05 | 0.001338 | 0.911382 | UP |
| RP11-474P2.7 | 1.733728 | -4.50712 | 4.579145 | 3.77E-05 | 0.001278 | 0.900754 | UP |
| LINC01118 | 1.944935 | -4.22424 | 4.55358 | 4.09E-05 | 0.001366 | 0.894167 | UP |
| CTLA4 | 1.73092 | -4.02833 | 4.542282 | 4.25E-05 | 0.001402 | 0.892811 | UP |
| LINC00887 | 1.801151 | -4.4421 | 4.569804 | 3.88E-05 | 0.001307 | 0.886506 | UP |
| KIAA1211L | 1.165079 | -0.9935 | 4.547786 | 4.17E-05 | 0.001383 | 0.882249 | UP |
| PCDH17 | 1.124276 | 1.125748 | 4.659045 | 2.91E-05 | 0.001044 | 0.877274 | UP |
| MIR6730 | 2.167634 | -4.33134 | 4.556077 | 4.06E-05 | 0.00136 | 0.87608 | UP |
| HIGD1AP1 | 1.449758 | -4.79159 | 4.613373 | 3.37E-05 | 0.001173 | 0.870079 | UP |
| CDC7 | 1.364174 | 0.109855 | 4.599466 | 3.53E-05 | 0.00121 | 0.867742 | UP |
| AKR7A2P1 | 1.554509 | -4.85572 | 4.6216 | 3.28E-05 | 0.001148 | 0.86718 | UP |
| GBP2 | 1.090557 | 5.187075 | 4.856978 | 1.52E-05 | 0.000628 | 0.854988 | UP |
| FFAR2 | 1.51633 | -0.06005 | 4.583396 | 3.72E-05 | 0.001264 | 0.848139 | UP |
| FGF14-IT1 | 2.708404 | -4.24389 | 4.539595 | 4.28E-05 | 0.001411 | 0.825111 | UP |
| LINC01215 | 1.938871 | -4.43578 | 4.549556 | 4.15E-05 | 0.001379 | 0.821253 | UP |
| PVRL3-AS1 | 1.522481 | -3.90093 | 4.48868 | 5.05E-05 | 0.001612 | 0.778108 | UP |
| RP11-399C16.3 | 2.273246 | -4.56128 | 4.545456 | 4.20E-05 | 0.001392 | 0.770828 | UP |
| NFKBIZ | 1.391819 | 6.290251 | 4.88033 | 1.41E-05 | 0.000589 | 0.77036 | UP |
| FA2H | 1.78072 | -3.18621 | 4.447098 | 5.77E-05 | 0.001795 | 0.763685 | UP |
| HK2 | 1.129104 | 1.588299 | 4.64364 | 3.06E-05 | 0.001083 | 0.745884 | UP |
| SOX30 | 1.827214 | -3.88767 | 4.476149 | 5.26E-05 | 0.001664 | 0.743938 | UP |
| PANX1 | 1.067348 | 4.179923 | 4.772821 | 2.00E-05 | 0.000775 | 0.734662 | UP |
| RP4-607I7.1 | 1.241532 | -5.16282 | 4.64928 | 3.00E-05 | 0.001067 | 0.723947 | UP |
| MIR4435-1HG | 1.206606 | 0.428783 | 4.571644 | 3.86E-05 | 0.001303 | 0.722689 | UP |
| SNORD11 | 2.383079 | -4.24064 | 4.499209 | 4.88E-05 | 0.001567 | 0.707869 | UP |
| GK | 1.069349 | 3.515866 | 4.729924 | 2.31E-05 | 0.000867 | 0.69888 | UP |
| RP11-1299A16.3 | 1.508603 | -4.55812 | 4.527908 | 4.45E-05 | 0.001454 | 0.697096 | UP |
| F2RL1 | 1.428908 | 3.002314 | 4.701111 | 2.53E-05 | 0.000935 | 0.694638 | UP |
| ZFP36L2 | 1.139323 | 7.268047 | 4.879029 | 1.41E-05 | 0.000591 | 0.619552 | UP |
| SH2D5 | 3.262531 | -2.50269 | 4.429268 | 6.11E-05 | 0.001875 | 0.615539 | UP |
| TMPRSS2 | 1.165007 | 5.532235 | 4.797994 | 1.85E-05 | 0.000727 | 0.613608 | UP |
| KRT8P48 | 2.168293 | -4.54546 | 4.493804 | 4.97E-05 | 0.00159 | 0.613478 | UP |
| TNFRSF10A | 1.186291 | 2.64652 | 4.656047 | 2.94E-05 | 0.00105 | 0.603919 | UP |
| RP11-151N17.1 | 1.652373 | -3.32283 | 4.392955 | 6.87E-05 | 0.002056 | 0.59147 | UP |
| RP11-253M7.6 | 1.159255 | -5.16789 | 4.607093 | 3.44E-05 | 0.00119 | 0.582926 | UP |
| NPM1P37 | 1.997753 | -4.11662 | 4.445543 | 5.80E-05 | 0.001802 | 0.575416 | UP |
| RP11-473C18.3 | 1.763094 | -3.96659 | 4.420903 | 6.28E-05 | 0.001912 | 0.546536 | UP |
| KLF7P1 | 2.169762 | -4.57765 | 4.474008 | 5.29E-05 | 0.001673 | 0.544281 | UP |
| CTC-250I14.6 | 1.858791 | 1.107484 | 4.548041 | 4.17E-05 | 0.001383 | 0.54317 | UP |
| RP11-373D23.2 | 2.07336 | -4.15838 | 4.450617 | 5.71E-05 | 0.001778 | 0.533378 | UP |
| RP11-430E17.1 | 1.573383 | -4.88705 | 4.51745 | 4.60E-05 | 0.001493 | 0.519133 | UP |
| PRR26 | 1.10976 | 3.120576 | 4.65312 | 2.96E-05 | 0.001059 | 0.516109 | UP |
| RP11-439L18.2 | 2.024598 | -3.44384 | 4.379591 | 7.17E-05 | 0.002131 | 0.514574 | UP |
| BTG2 | 1.797069 | 4.170625 | 4.69828 | 2.56E-05 | 0.000941 | 0.50869 | UP |
| BATF | 1.546418 | -0.56926 | 4.439759 | 5.91E-05 | 0.001832 | 0.478205 | UP |
| RP11-400F19.18 | 1.41726 | -0.47067 | 4.444472 | 5.82E-05 | 0.001808 | 0.477022 | UP |
| AC116366.6 | 2.160316 | -2.49109 | 4.363171 | 7.55E-05 | 0.002211 | 0.476025 | UP |
| CFLAR-AS1 | 1.579479 | -2.35145 | 4.354109 | 7.77E-05 | 0.002264 | 0.450818 | UP |
| SIGLEC5 | 1.633279 | -1.34987 | 4.390604 | 6.92E-05 | 0.002069 | 0.445766 | UP |
| RP11-611O2.1 | 1.920061 | -4.31706 | 4.415431 | 6.39E-05 | 0.001939 | 0.435569 | UP |
| KCNE1 | 1.156398 | 1.755024 | 4.551159 | 4.13E-05 | 0.001374 | 0.422667 | UP |
| AC079305.10 | 1.544575 | -4.39774 | 4.409514 | 6.51E-05 | 0.001967 | 0.398196 | UP |
| LETM2 | 1.778878 | -1.64203 | 4.361295 | 7.60E-05 | 0.002221 | 0.394833 | UP |
| FHL2 | 1.560784 | 2.744062 | 4.592364 | 3.61E-05 | 0.001235 | 0.39256 | UP |
| FPR2 | 1.522931 | 1.836392 | 4.538833 | 4.29E-05 | 0.001413 | 0.377556 | UP |
| ROS1 | 2.160068 | -4.4829 | 4.412936 | 6.44E-05 | 0.001951 | 0.370678 | UP |
| DCDC2B | 1.576666 | -4.56612 | 4.416887 | 6.36E-05 | 0.001932 | 0.362235 | UP |
| SRSF12 | 2.148252 | -2.06186 | 4.335641 | 8.25E-05 | 0.002377 | 0.358889 | UP |
| SERPINH1 | 1.259432 | 5.593232 | 4.720667 | 2.38E-05 | 0.000888 | 0.356308 | UP |
| HOMER1 | 1.444494 | 0.109195 | 4.431656 | 6.06E-05 | 0.001865 | 0.33993 | UP |
| RP11-1100L3.8 | 2.902684 | 0.304618 | 4.428942 | 6.12E-05 | 0.001875 | 0.335466 | UP |
| VDR | 1.54838 | 0.313679 | 4.437337 | 5.96E-05 | 0.001842 | 0.324442 | UP |
| CAMK2A | 1.669722 | -4.15753 | 4.369943 | 7.39E-05 | 0.002176 | 0.319075 | UP |
| LINC00311 | 1.69662 | -4.60419 | 4.410678 | 6.49E-05 | 0.00196 | 0.309427 | UP |
| S100A2 | 1.604268 | -4.47183 | 4.402934 | 6.65E-05 | 0.002001 | 0.287501 | UP |
| HSPD1P6 | 1.312081 | -3.70387 | 4.30049 | 9.22E-05 | 0.002592 | 0.272757 | UP |
| KRT16P2 | 6.94002 | -0.26049 | 4.363445 | 7.55E-05 | 0.002211 | 0.270843 | UP |
| MEDAG | 2.828814 | -1.73466 | 4.31776 | 8.73E-05 | 0.002491 | 0.266374 | UP |
| RP11-92G12.3 | 1.662026 | -4.47124 | 4.37726 | 7.22E-05 | 0.002142 | 0.263823 | UP |
| KCNH1 | 2.165264 | -3.28755 | 4.300278 | 9.23E-05 | 0.002592 | 0.261631 | UP |
| CHRNA10 | 1.29485 | -0.57921 | 4.369422 | 7.40E-05 | 0.002178 | 0.257511 | UP |
| RTEL1P1 | 2.529348 | -4.06675 | 4.333497 | 8.30E-05 | 0.002391 | 0.248609 | UP |
| CEMIP | 2.087479 | -3.23624 | 4.287709 | 9.60E-05 | 0.002673 | 0.237669 | UP |
| NR4A1 | 2.858193 | 5.975525 | 4.689344 | 2.63E-05 | 0.000962 | 0.234943 | UP |
| CH17-360D5.3 | 2.250178 | -4.12028 | 4.332728 | 8.32E-05 | 0.002395 | 0.222996 | UP |
| CCDC150P1 | 2.028604 | -2.78988 | 4.271589 | 0.000101 | 0.002779 | 0.200388 | UP |
| LINC00877 | 1.620373 | -3.22521 | 4.260765 | 0.000105 | 0.002854 | 0.190867 | UP |
| RP5-1077I2.3 | 1.684392 | -4.50052 | 4.372868 | 7.32E-05 | 0.002163 | 0.186478 | UP |
| RP11-769O8.3 | 1.562819 | -1.95176 | 4.28159 | 9.79E-05 | 0.002711 | 0.185738 | UP |
| C19orf26 | 1.431775 | -0.50971 | 4.342995 | 8.05E-05 | 0.002331 | 0.165828 | UP |
| AP002954.3 | 1.734871 | -3.06783 | 4.248974 | 0.000109 | 0.002936 | 0.154224 | UP |
| RP11-1029J19.2 | 1.587172 | -5.03905 | 4.43434 | 6.01E-05 | 0.001853 | 0.150125 | UP |
| RP11-499O7.7 | 2.160909 | -3.90402 | 4.285328 | 9.68E-05 | 0.002688 | 0.140734 | UP |
| KRT8P43 | 1.247445 | -4.95432 | 4.411125 | 6.48E-05 | 0.001959 | 0.136614 | UP |
| HILPDA | 1.154784 | 0.821083 | 4.406623 | 6.57E-05 | 0.00198 | 0.132859 | UP |
| HSP90AB2P | 1.713522 | -2.18774 | 4.256658 | 0.000106 | 0.002884 | 0.132261 | UP |
| MAB21L3 | 2.010126 | 0.402436 | 4.376157 | 7.24E-05 | 0.002146 | 0.129157 | UP |
| EFHC2 | 1.994799 | -4.67221 | 4.361751 | 7.59E-05 | 0.00222 | 0.122352 | UP |
| RP11-399K21.12 | 1.6949 | -4.64677 | 4.345348 | 7.99E-05 | 0.002318 | 0.110572 | UP |
| AC073052.1 | 1.861422 | -4.30397 | 4.299582 | 9.25E-05 | 0.002593 | 0.094601 | UP |
| MT-TS2 | 1.768709 | -4.3446 | 4.307312 | 9.02E-05 | 0.002547 | 0.092836 | UP |
| HIST1H2BF | 1.959414 | -4.73571 | 4.356582 | 7.71E-05 | 0.00225 | 0.085443 | UP |
| CYP27B1 | 1.641869 | -0.58471 | 4.297436 | 9.31E-05 | 0.002607 | 0.040507 | UP |
| ZNF367 | 1.268862 | 2.165039 | 4.451452 | 5.69E-05 | 0.001774 | 0.038839 | UP |
| PIP5KL1 | 1.565022 | -1.95754 | 4.232988 | 0.000114 | 0.003056 | 0.036395 | UP |
| SLC8A2 | 1.909384 | -3.4512 | 4.21607 | 0.00012 | 0.003189 | 0.002817 | UP |
| TBC1D30 | 1.133314 | 1.026018 | 4.375637 | 7.26E-05 | 0.002147 | -0.00091 | UP |
| RP11-757F18.5 | 1.638747 | -4.12851 | 4.266623 | 0.000103 | 0.002812 | -0.02116 | UP |
| MIR5587 | 2.196017 | -4.24574 | 4.256073 | 0.000106 | 0.002888 | -0.02579 | UP |
| LA16c-329F2.2 | 1.545133 | -5.04801 | 4.375948 | 7.25E-05 | 0.002146 | -0.03127 | UP |
| MIR3176 | 2.075962 | -3.86232 | 4.202277 | 0.000126 | 0.003299 | -0.09233 | UP |
| ADAM19 | 1.38982 | 4.353136 | 4.520566 | 4.56E-05 | 0.001483 | -0.09301 | UP |
| IRAK2 | 1.222386 | 3.673817 | 4.481203 | 5.17E-05 | 0.001643 | -0.11564 | UP |
| RP11-823E8.3 | 1.206497 | 1.420985 | 4.355033 | 7.75E-05 | 0.002258 | -0.13339 | UP |
| AC145676.2 | 1.997518 | -3.78432 | 4.184532 | 0.000133 | 0.003433 | -0.13644 | UP |
| MAB21L2 | 1.604482 | 0.149969 | 4.279584 | 9.85E-05 | 0.002724 | -0.13915 | UP |
| TTC22 | 1.678538 | 2.847208 | 4.425283 | 6.19E-05 | 0.001891 | -0.15056 | UP |
| RP11-30K9.4 | 1.524085 | -4.86656 | 4.299538 | 9.25E-05 | 0.002593 | -0.15195 | UP |
| CCDC141 | 1.881263 | -2.57075 | 4.150416 | 0.000148 | 0.003727 | -0.16736 | UP |
| LYPD5 | 2.670171 | -2.42616 | 4.162523 | 0.000143 | 0.003614 | -0.16906 | UP |
| ANXA2 | 1.048 | 5.353119 | 4.541056 | 4.26E-05 | 0.001406 | -0.1832 | UP |
| FOXJ1 | 1.566963 | -3.49245 | 4.143571 | 0.000151 | 0.003795 | -0.19053 | UP |
| PLD5 | 1.970785 | -4.52035 | 4.227164 | 0.000116 | 0.003101 | -0.20082 | UP |
| BCLAF1P1 | 1.602883 | -4.53792 | 4.223153 | 0.000118 | 0.003129 | -0.21174 | UP |
| RP11-7I15.3 | 1.777369 | -3.52867 | 4.142603 | 0.000152 | 0.003804 | -0.21414 | UP |
| RP1-47A17.1 | 1.760132 | -3.88474 | 4.171491 | 0.000139 | 0.00354 | -0.21659 | UP |
| RP11-820I16.4 | 1.624735 | -4.50365 | 4.218923 | 0.000119 | 0.003165 | -0.21972 | UP |
| RP4-796I17.5 | 1.399513 | -4.6539 | 4.235134 | 0.000113 | 0.003039 | -0.22335 | UP |
| LZTS1 | 1.042823 | 0.634548 | 4.277231 | 9.93E-05 | 0.00274 | -0.23809 | UP |
| GAREM | 1.236528 | 4.435987 | 4.465423 | 5.44E-05 | 0.001709 | -0.28176 | UP |
| RP4-756H11.5 | 1.289634 | -1.03104 | 4.165114 | 0.000141 | 0.003595 | -0.30123 | UP |
| RP11-63P12.7 | 1.635098 | -2.53256 | 4.102534 | 0.000172 | 0.004193 | -0.30802 | UP |
| NLRP3 | 1.367504 | 0.947683 | 4.269924 | 0.000102 | 0.002789 | -0.30987 | UP |
| FPR1 | 1.111322 | 4.064229 | 4.436836 | 5.96E-05 | 0.001844 | -0.31701 | UP |
| TMSB15A | 1.552364 | -4.90171 | 4.240908 | 0.000111 | 0.002989 | -0.35251 | UP |
| RP11-276H7.2 | 2.147546 | -2.16489 | 4.10082 | 0.000173 | 0.00421 | -0.35334 | UP |
| ATP2C2 | 1.838124 | -2.29585 | 4.08419 | 0.000182 | 0.0044 | -0.38537 | UP |
| RNASEH2B-AS1 | 1.532538 | -4.19299 | 4.129517 | 0.000158 | 0.003933 | -0.39822 | UP |
| MMP9 | 1.90802 | 3.135655 | 4.359151 | 7.65E-05 | 0.002234 | -0.39879 | UP |
| MIR7106 | 1.834359 | -4.50619 | 4.158327 | 0.000145 | 0.003656 | -0.4028 | UP |
| RP11-697N18.3 | 1.977612 | -2.94883 | 4.069067 | 0.000191 | 0.004561 | -0.41149 | UP |
| STAR | 1.741311 | -3.8273 | 4.090021 | 0.000179 | 0.004331 | -0.42112 | UP |
| PPP1R1B | 1.701225 | -4.27947 | 4.140136 | 0.000153 | 0.003827 | -0.42484 | UP |
| SPRED3 | 2.18284 | -1.72304 | 4.077137 | 0.000186 | 0.004471 | -0.46751 | UP |
| 3-Mar | 1.115694 | 0.37297 | 4.184457 | 0.000133 | 0.003433 | -0.47677 | UP |
| RP11-405M12.3 | 1.155045 | -5.06475 | 4.242067 | 0.000111 | 0.002983 | -0.48168 | UP |
| A3GALT2 | 1.734919 | -4.14158 | 4.098744 | 0.000174 | 0.004234 | -0.48813 | UP |
| MTND6P3 | 1.479279 | -4.4177 | 4.120506 | 0.000163 | 0.004015 | -0.49945 | UP |
| RP11-29G8.3 | 1.407995 | -1.59609 | 4.071935 | 0.000189 | 0.004526 | -0.50007 | UP |
| LA16c-431H6.6 | 1.472104 | -3.97007 | 4.076 | 0.000187 | 0.004479 | -0.50958 | UP |
| SPEF1 | 2.305713 | -4.42016 | 4.109526 | 0.000168 | 0.00413 | -0.5282 | UP |
| TFPI2 | 1.504497 | 2.975222 | 4.310252 | 8.94E-05 | 0.002531 | -0.53376 | UP |
| RP3-416H24.1 | 1.528937 | -3.58412 | 4.02797 | 0.000217 | 0.005032 | -0.5429 | UP |
| CCDC65 | 1.496191 | -2.74636 | 4.01443 | 0.000227 | 0.005208 | -0.55819 | UP |
| MEFV | 1.126253 | 1.298308 | 4.199882 | 0.000127 | 0.003317 | -0.59195 | UP |
| RP11-532M24.1 | 1.615305 | -3.77946 | 4.023247 | 0.00022 | 0.005088 | -0.59762 | UP |
| CTD-2012K14.6 | 1.706747 | -4.52626 | 4.095051 | 0.000176 | 0.004279 | -0.60366 | UP |
| HS3ST1 | 1.562732 | -0.67506 | 4.082578 | 0.000183 | 0.004418 | -0.60472 | UP |
| LPAR3 | 1.421276 | -4.7757 | 4.13067 | 0.000158 | 0.003924 | -0.62035 | UP |
| DLGAP1-AS1 | 1.102571 | 0.680903 | 4.151265 | 0.000148 | 0.003722 | -0.63206 | UP |
| SLAMF1 | 1.852372 | -1.27555 | 4.038031 | 0.000211 | 0.004912 | -0.64385 | UP |
| NCR3LG1 | 1.669725 | 0.643275 | 4.140075 | 0.000153 | 0.003827 | -0.65001 | UP |
| RP11-106D4.2 | 1.516739 | -1.20098 | 4.03972 | 0.000209 | 0.0049 | -0.65394 | UP |
| TNFSF15 | 2.034166 | -3.05695 | 3.986404 | 0.000247 | 0.005602 | -0.66061 | UP |
| RBM24 | 1.659204 | -1.6024 | 4.013351 | 0.000227 | 0.005218 | -0.67496 | UP |
| ZFP36 | 1.344453 | 8.448884 | 4.523145 | 4.52E-05 | 0.001473 | -0.69206 | UP |
| ZNF331 | 1.406452 | 3.724048 | 4.29728 | 9.32E-05 | 0.002607 | -0.69582 | UP |
| RP11-1143G9.2 | 1.987115 | -4.24571 | 4.030919 | 0.000215 | 0.004994 | -0.70947 | UP |
| GPR52 | 1.666981 | -4.43322 | 4.08041 | 0.000185 | 0.004436 | -0.70998 | UP |
| EPPK1 | 1.515753 | -0.25973 | 4.065902 | 0.000193 | 0.004599 | -0.72352 | UP |
| CXCL10 | 2.003721 | -0.99215 | 4.023225 | 0.00022 | 0.005088 | -0.72906 | UP |
| RP11-255P5.3 | 2.000629 | -4.19108 | 4.040474 | 0.000209 | 0.004892 | -0.73835 | UP |
| AGPAT4-IT1 | 1.405466 | -3.36203 | 3.951941 | 0.000275 | 0.006052 | -0.73979 | UP |
| SLC34A2 | 1.22398 | 0.731623 | 4.117976 | 0.000164 | 0.004037 | -0.74104 | UP |
| RP11-196B3.1 | 1.230129 | -4.98727 | 4.114695 | 0.000166 | 0.004072 | -0.80236 | UP |
| SNORA31 | 1.586476 | -2.90018 | 3.928684 | 0.000295 | 0.006391 | -0.8089 | UP |
| SPOCD1 | 2.625879 | -2.17633 | 3.952884 | 0.000274 | 0.006039 | -0.80916 | UP |
| ANKRD36C | 1.307162 | -1.26231 | 3.98 | 0.000252 | 0.005687 | -0.82491 | UP |
| CHMP4BP1 | 1.62693 | -3.77262 | 3.949028 | 0.000277 | 0.006087 | -0.8276 | UP |
| PODXL | 1.242171 | 3.207473 | 4.227585 | 0.000116 | 0.003099 | -0.83163 | UP |
| RP11-16E23.4 | 1.674305 | -3.65381 | 3.931965 | 0.000292 | 0.006341 | -0.83686 | UP |
| MTND5P14 | 1.983685 | -4.75741 | 4.043525 | 0.000207 | 0.00486 | -0.86451 | UP |
| RP11-800A18.3 | 1.778163 | -3.98844 | 3.949905 | 0.000277 | 0.006074 | -0.87468 | UP |
| SPRY4-IT1 | 1.46657 | -1.24284 | 3.955202 | 0.000272 | 0.005999 | -0.9005 | UP |
| FCN1 | 1.24998 | 2.928982 | 4.189984 | 0.000131 | 0.003387 | -0.9028 | UP |
| TAF4B | 1.319767 | -1.17909 | 3.952716 | 0.000274 | 0.00604 | -0.91932 | UP |
| SFN | 2.551895 | -0.89851 | 3.956477 | 0.000271 | 0.005982 | -0.93246 | UP |
| CTD-3252C9.4 | 1.166377 | 2.106225 | 4.135155 | 0.000155 | 0.003876 | -0.93325 | UP |
| LINC00628 | 1.55123 | -4.4532 | 3.98198 | 0.000251 | 0.005657 | -0.93353 | UP |
| LRFN5 | 2.511783 | -4.3481 | 3.96736 | 0.000262 | 0.005842 | -0.93922 | UP |
| RP11-278L15.6 | 1.018947 | 1.253049 | 4.080473 | 0.000184 | 0.004436 | -0.95118 | UP |
| AC012363.4 | 1.421706 | -4.49755 | 3.973336 | 0.000257 | 0.005768 | -0.96163 | UP |
| PHLDA2 | 2.020285 | -3.27859 | 3.908086 | 0.000315 | 0.006709 | -0.9674 | UP |
| RP11-106M3.1 | 1.320163 | -4.99034 | 4.055904 | 0.000199 | 0.00471 | -0.98468 | UP |
| AC022816.2 | 1.175373 | -0.77984 | 3.949976 | 0.000277 | 0.006074 | -0.99173 | UP |
| RP11-139H15.5 | 1.392925 | -3.79318 | 3.888605 | 0.000334 | 0.006987 | -1.00456 | UP |
| CDCP1 | 1.057176 | 0.762098 | 4.028192 | 0.000217 | 0.00503 | -1.02072 | UP |
| RP11-360N9.2 | 2.121831 | -3.81609 | 3.887985 | 0.000335 | 0.006998 | -1.02481 | UP |
| URB2 | 1.00363 | 2.458114 | 4.120174 | 0.000163 | 0.004018 | -1.04201 | UP |
| RP3-467N11.2 | 1.493285 | -4.21508 | 3.905039 | 0.000318 | 0.006759 | -1.07565 | UP |
| HSP90AA2P | 1.418371 | -0.64842 | 3.926251 | 0.000298 | 0.006425 | -1.07936 | UP |
| STARD13-IT1 | 1.31785 | -4.8878 | 3.999776 | 0.000237 | 0.005406 | -1.07948 | UP |
| UBBP2 | 1.457825 | -4.20646 | 3.901232 | 0.000321 | 0.006805 | -1.08222 | UP |
| INSL3 | 1.719223 | -4.55677 | 3.934862 | 0.00029 | 0.006301 | -1.09959 | UP |
| TLK2P1 | 1.442534 | -4.33758 | 3.909617 | 0.000313 | 0.006693 | -1.10017 | UP |
| AP000240.9 | 2.226262 | -3.8928 | 3.865115 | 0.000359 | 0.007392 | -1.10812 | UP |
| GABRR3 | 2.151293 | -4.21754 | 3.914393 | 0.000309 | 0.00661 | -1.12407 | UP |
| SLC35F1 | 1.662771 | -4.32465 | 3.904881 | 0.000318 | 0.006759 | -1.12423 | UP |
| SNORD83A | 1.615519 | -2.15175 | 3.83698 | 0.000391 | 0.007874 | -1.13416 | UP |
| INHBA | 1.385237 | 3.257798 | 4.129745 | 0.000158 | 0.003932 | -1.13719 | UP |
| RP1-67K17.3 | 1.813495 | -3.67868 | 3.837268 | 0.000391 | 0.007874 | -1.139 | UP |
| FAM43B | 1.533805 | -1.61875 | 3.8424 | 0.000385 | 0.007794 | -1.18163 | UP |
| JUNB | 1.642017 | 7.411184 | 4.315694 | 8.79E-05 | 0.002502 | -1.18672 | UP |
| RP11-121A14.3 | 1.793757 | -4.35874 | 3.879619 | 0.000343 | 0.007134 | -1.20956 | UP |
| MYF6 | 1.47498 | -4.21899 | 3.888892 | 0.000334 | 0.006984 | -1.21016 | UP |
| RP11-139K1.2 | 1.612132 | -3.981 | 3.850832 | 0.000375 | 0.007626 | -1.21969 | UP |
| RP11-75C10.6 | 1.589028 | -2.64599 | 3.791862 | 0.000449 | 0.008755 | -1.22498 | UP |
| RP11-53I6.3 | 2.017884 | -4.43402 | 3.870803 | 0.000353 | 0.007295 | -1.23683 | UP |
| NACAD | 1.586417 | -2.38471 | 3.785916 | 0.000457 | 0.008887 | -1.26059 | UP |
| B3GNTL1P1 | 1.190917 | -4.92867 | 3.945839 | 0.00028 | 0.006132 | -1.27062 | UP |
| SULT2B1 | 1.601926 | -4.24113 | 3.834676 | 0.000394 | 0.007922 | -1.28103 | UP |
| RP11-163F15.1 | 1.338527 | -4.99133 | 3.957825 | 0.00027 | 0.005966 | -1.28315 | UP |
| TMEFF2 | 1.307615 | -4.8879 | 3.930802 | 0.000293 | 0.006357 | -1.29042 | UP |
| APOBEC3A | 1.73914 | -1.7677 | 3.79775 | 0.000441 | 0.008638 | -1.29253 | UP |
| EYS | 1.463362 | -1.54142 | 3.806103 | 0.00043 | 0.008472 | -1.29804 | UP |
| CTD-2260A17.1 | 1.352665 | -1.06944 | 3.826072 | 0.000404 | 0.008068 | -1.31047 | UP |
| SLC25A2 | 1.379754 | -4.91022 | 3.927168 | 0.000297 | 0.006416 | -1.31179 | UP |
| ENDOU | 1.698719 | -3.76608 | 3.772083 | 0.000476 | 0.009188 | -1.33595 | UP |
| RP3-508I15.19 | 1.066664 | -0.83701 | 3.829541 | 0.0004 | 0.008009 | -1.3398 | UP |
| KLK10 | 1.825821 | -3.51072 | 3.765038 | 0.000487 | 0.00934 | -1.34508 | UP |
| RP11-153M3.1 | 1.135954 | -2.65997 | 3.743071 | 0.00052 | 0.00982 | -1.35499 | UP |
| AL133243.4 | 2.026226 | -4.21869 | 3.800984 | 0.000436 | 0.008573 | -1.36661 | UP |
| GPR97 | 1.5406 | 1.983805 | 3.97663 | 0.000255 | 0.005729 | -1.38287 | UP |
| WNT9A | 1.705265 | -2.38542 | 3.741456 | 0.000523 | 0.00985 | -1.3907 | UP |
| RN7SL559P | 1.186289 | -5.00022 | 3.919788 | 0.000304 | 0.006523 | -1.39593 | UP |
| LILRP2 | 1.479554 | -4.67418 | 3.850601 | 0.000375 | 0.007626 | -1.40239 | UP |
| CCR7 | 1.686583 | -2.50923 | 3.726791 | 0.000546 | 0.010176 | -1.42532 | UP |
| SDCBP2 | 1.000925 | 1.723334 | 3.948974 | 0.000277 | 0.006087 | -1.4306 | UP |
| DDIT3 | 1.008724 | 3.44832 | 4.042646 | 0.000208 | 0.004866 | -1.43812 | UP |
| CTA-941F9.10 | 1.182685 | -2.10123 | 3.726666 | 0.000547 | 0.010176 | -1.45757 | UP |
| FAM57A | 1.271758 | 1.047988 | 3.895977 | 0.000327 | 0.006872 | -1.46355 | UP |
| LL21NC02-21A1.1 | 1.260151 | -1.50979 | 3.750984 | 0.000508 | 0.00965 | -1.46469 | UP |
| KIAA1644 | 1.149475 | -1.04455 | 3.772178 | 0.000476 | 0.009188 | -1.47409 | UP |
| SLC7A11 | 1.901823 | -3.14303 | 3.70723 | 0.000579 | 0.010623 | -1.47637 | UP |
| AC003104.1 | 1.7148 | -3.99862 | 3.743609 | 0.000519 | 0.009807 | -1.47992 | UP |
| HSPA7 | 2.175021 | -0.42458 | 3.792123 | 0.000448 | 0.008753 | -1.49897 | UP |
| NPAS1 | 1.725197 | -3.46048 | 3.693609 | 0.000604 | 0.010967 | -1.51958 | UP |
| RP4-710M16.2 | 1.07292 | 1.430898 | 3.898108 | 0.000324 | 0.006858 | -1.52864 | UP |
| RP11-39K24.12 | 1.390355 | -4.62469 | 3.792713 | 0.000448 | 0.008747 | -1.54276 | UP |
| GJB3 | 2.0611 | -0.95055 | 3.74811 | 0.000512 | 0.009715 | -1.54637 | UP |
| SLC52A3 | 1.499864 | -2.99889 | 3.671744 | 0.000645 | 0.011523 | -1.55395 | UP |
| RP11-349F21.4 | 1.72601 | -2.22043 | 3.687704 | 0.000614 | 0.011093 | -1.56207 | UP |
| FAM60A | 1.563063 | -1.8706 | 3.693064 | 0.000605 | 0.010975 | -1.5833 | UP |
| RP11-737O24.3 | 1.365484 | -1.5525 | 3.704105 | 0.000585 | 0.010686 | -1.59376 | UP |
| TM4SF1-AS1 | 1.952889 | -4.35049 | 3.740971 | 0.000523 | 0.009859 | -1.59554 | UP |
| HPDL | 1.672752 | -3.86469 | 3.697786 | 0.000596 | 0.010858 | -1.59755 | UP |
| CATSPER1 | 1.80265 | -4.44243 | 3.761253 | 0.000492 | 0.009421 | -1.59782 | UP |
| ANXA2P2 | 1.199935 | -2.59705 | 3.659406 | 0.000669 | 0.011812 | -1.60213 | UP |
| SH2D2A | 1.066861 | 0.37271 | 3.808031 | 0.000427 | 0.008431 | -1.60646 | UP |
| SEMA3C | 1.128885 | -0.95678 | 3.73088 | 0.00054 | 0.010086 | -1.60819 | UP |
| SNORD70 | 1.153696 | -5.13306 | 3.890983 | 0.000332 | 0.006954 | -1.60842 | UP |
| PFKP | 1.265778 | 1.925873 | 3.897312 | 0.000325 | 0.00686 | -1.6156 | UP |
| CTB-181H17.1 | 1.424426 | -4.41111 | 3.746419 | 0.000515 | 0.009749 | -1.61697 | UP |
| MIR548V | 1.709718 | -4.30419 | 3.716475 | 0.000564 | 0.010394 | -1.63052 | UP |
| RP11-392A14.9 | 1.438706 | -4.18158 | 3.714464 | 0.000567 | 0.010434 | -1.63318 | UP |
| MAGI1-IT1 | 1.493791 | -2.23501 | 3.658922 | 0.00067 | 0.011822 | -1.64054 | UP |
| RP11-484N16.1 | 1.741298 | -0.74375 | 3.725349 | 0.000549 | 0.010208 | -1.64964 | UP |
| MDFI | 1.979199 | -2.95456 | 3.644353 | 0.0007 | 0.012229 | -1.65904 | UP |
| CTA-414D7.1 | 2.54934 | -3.96586 | 3.677592 | 0.000633 | 0.011362 | -1.65979 | UP |
| RRN3P2 | 1.30365 | -3.46515 | 3.639568 | 0.00071 | 0.012356 | -1.66371 | UP |
| PLEKHN1 | 1.758389 | -1.20558 | 3.693615 | 0.000604 | 0.010967 | -1.66998 | UP |
| MACC1 | 1.299839 | -0.88327 | 3.712612 | 0.00057 | 0.010482 | -1.67046 | UP |
| CTD-2342N23.1 | 1.527957 | -4.6057 | 3.744011 | 0.000519 | 0.009801 | -1.68229 | UP |
| TMEM151A | 1.337958 | -3.29874 | 3.622586 | 0.000747 | 0.012853 | -1.68974 | UP |
| CTB-131B5.2 | 1.685241 | -4.56217 | 3.73131 | 0.000539 | 0.010081 | -1.69058 | UP |
| RP11-456P18.2 | 1.26043 | -1.83541 | 3.651242 | 0.000685 | 0.012042 | -1.70765 | UP |
| KCNK15 | 1.86116 | -2.94961 | 3.622947 | 0.000746 | 0.012847 | -1.71268 | UP |
| RP11-850A17.1 | 1.3058 | -4.58283 | 3.722309 | 0.000554 | 0.010262 | -1.72162 | UP |
| RP11-351C21.2 | 1.475299 | -3.30501 | 3.612996 | 0.000768 | 0.013136 | -1.72866 | UP |
| CH17-140K24.2 | 1.606769 | -5.01647 | 3.812804 | 0.000421 | 0.008341 | -1.73428 | UP |
| RP11-34P13.16 | 1.216049 | -2.21391 | 3.622554 | 0.000747 | 0.012853 | -1.74398 | UP |
| ANKRD2 | 1.757424 | -4.17839 | 3.664425 | 0.000659 | 0.011689 | -1.74603 | UP |
| NOX4 | 1.396351 | -3.88903 | 3.646917 | 0.000694 | 0.012158 | -1.74651 | UP |
| KRT23 | 1.733683 | -3.57842 | 3.614061 | 0.000766 | 0.013113 | -1.75134 | UP |
| NTS | 1.618849 | -4.1641 | 3.659607 | 0.000668 | 0.011808 | -1.7693 | UP |
| HSPE1 | 1.064856 | 3.24701 | 3.921032 | 0.000302 | 0.006502 | -1.76989 | UP |
| IL10 | 1.452926 | 1.420263 | 3.809608 | 0.000425 | 0.008407 | -1.78068 | UP |
| ITGB3 | 1.325162 | 1.352515 | 3.803369 | 0.000433 | 0.008525 | -1.7894 | UP |
| OXCT2 | 1.374122 | -4.29754 | 3.661975 | 0.000664 | 0.011754 | -1.79833 | UP |
| KLF3P1 | 1.342302 | -4.9507 | 3.770642 | 0.000479 | 0.009219 | -1.80534 | UP |
| S100A12 | 1.419034 | 2.259405 | 3.85133 | 0.000374 | 0.007624 | -1.80752 | UP |
| FLNC | 1.61893 | -0.64296 | 3.674881 | 0.000639 | 0.011443 | -1.81395 | UP |
| API5P2 | 1.523798 | -4.22372 | 3.646003 | 0.000696 | 0.012184 | -1.81405 | UP |
| RP11-71H17.8 | 1.380289 | -2.94535 | 3.577867 | 0.000853 | 0.014191 | -1.81863 | UP |
| SERPINB10 | 1.722282 | -4.38251 | 3.669073 | 0.00065 | 0.011591 | -1.82194 | UP |
| SCARNA5 | 2.216483 | -3.87777 | 3.618148 | 0.000756 | 0.012986 | -1.83541 | UP |
| GGN | 1.543851 | -3.63146 | 3.588176 | 0.000827 | 0.013892 | -1.84209 | UP |
| CTD-2313J17.1 | 1.777944 | -3.95461 | 3.614332 | 0.000765 | 0.013111 | -1.84476 | UP |
| RP1 | 1.712565 | -4.47897 | 3.65926 | 0.000669 | 0.011814 | -1.86712 | UP |
| BACH1-IT1 | 1.581663 | -3.45931 | 3.56807 | 0.000878 | 0.014527 | -1.86754 | UP |
| RP11-923I11.8 | 1.57008 | -3.75725 | 3.585673 | 0.000833 | 0.013955 | -1.86826 | UP |
| RP11-973D8.4 | 1.434862 | -4.19379 | 3.624307 | 0.000743 | 0.012817 | -1.87863 | UP |
| GJC1 | 1.286683 | -0.81593 | 3.643673 | 0.000701 | 0.012248 | -1.88138 | UP |
| HLA-DQA2 | 1.47833 | -4.44508 | 3.648391 | 0.000691 | 0.012116 | -1.88992 | UP |
| ITGA2 | 1.928108 | -0.37025 | 3.661393 | 0.000665 | 0.011771 | -1.89002 | UP |
| CTD-2526A2.2 | 1.269841 | -4.8393 | 3.714568 | 0.000567 | 0.010434 | -1.8916 | UP |
| TMCC3 | 1.001995 | 2.85123 | 3.85634 | 0.000369 | 0.007539 | -1.89812 | UP |
| FCGR1C | 1.806176 | -3.81458 | 3.581294 | 0.000844 | 0.014078 | -1.90905 | UP |
| NTNG2 | 1.663203 | -0.6206 | 3.641781 | 0.000705 | 0.012302 | -1.90987 | UP |
| RP11-359K18.4 | 1.069467 | -1.88758 | 3.560431 | 0.000898 | 0.014787 | -1.95898 | UP |
| C10orf10 | 1.476356 | 6.847024 | 4.031638 | 0.000215 | 0.004985 | -1.97642 | UP |
| RP11-755B10.2 | 1.464808 | -4.92525 | 3.702531 | 0.000588 | 0.010722 | -1.98637 | UP |
| RP11-540O11.4 | 1.635946 | -4.8451 | 3.682528 | 0.000624 | 0.011229 | -1.98793 | UP |
| UCA1 | 1.641463 | -0.98826 | 3.590844 | 0.00082 | 0.013833 | -1.99862 | UP |
| RP11-46J23.1 | 1.482507 | -3.37761 | 3.528696 | 0.000986 | 0.015836 | -2.00163 | UP |
| AC011753.5 | 1.400889 | -5.05555 | 3.729366 | 0.000542 | 0.010114 | -2.00465 | UP |
| FUT1 | 1.033332 | 0.762286 | 3.690797 | 0.000609 | 0.011036 | -2.0157 | UP |
| SH3D21 | 1.430602 | 1.214963 | 3.715009 | 0.000566 | 0.010427 | -2.01805 | UP |
| DNAJC19P5 | 1.315095 | -2.58812 | 3.51164 | 0.001036 | 0.016449 | -2.02399 | UP |
| AP001189.4 | 1.394629 | -3.62796 | 3.517806 | 0.001018 | 0.016226 | -2.02665 | UP |
| RP11-297L17.6 | 1.17725 | -4.08258 | 3.553641 | 0.000916 | 0.015019 | -2.02693 | UP |
| RPS4XP17 | 1.497533 | -4.79929 | 3.652691 | 0.000682 | 0.012001 | -2.03775 | UP |
| FGF23 | 2.526404 | -1.50372 | 3.547713 | 0.000932 | 0.015199 | -2.04098 | UP |
| IL24 | 1.920746 | -3.73007 | 3.567472 | 0.000879 | 0.01454 | -2.04296 | UP |
| RP11-154H23.3 | 2.00317 | -4.01173 | 3.538322 | 0.000958 | 0.015504 | -2.05691 | UP |
| LGALS12 | 1.877256 | -4.06353 | 3.545312 | 0.000939 | 0.015285 | -2.06114 | UP |
| PRKCG | 1.249526 | -4.40597 | 3.632825 | 0.000724 | 0.012556 | -2.06711 | UP |
| RP11-61L19.3 | 1.458916 | -3.90604 | 3.541777 | 0.000949 | 0.015407 | -2.07487 | UP |
| RP11-638I2.4 | 1.502915 | -4.08653 | 3.540828 | 0.000951 | 0.015433 | -2.07826 | UP |
| CTB-50E14.5 | 1.112252 | 2.085148 | 3.749581 | 0.00051 | 0.009682 | -2.07976 | UP |
| YPEL2 | 1.089044 | 5.564215 | 3.934002 | 0.000291 | 0.006315 | -2.08413 | UP |
| TMEM158 | 1.19787 | -1.06955 | 3.554093 | 0.000915 | 0.015003 | -2.0952 | UP |
| ARRDC3 | 1.182303 | 6.291152 | 3.960893 | 0.000267 | 0.005928 | -2.11094 | UP |
| RP11-435O5.7 | 1.711717 | -3.70026 | 3.486231 | 0.001117 | 0.017378 | -2.1289 | UP |
| AC017101.10 | 1.265006 | -4.74484 | 3.608167 | 0.000779 | 0.013283 | -2.12988 | UP |
| MCF2L2 | 1.42974 | -3.91459 | 3.520684 | 0.001009 | 0.016098 | -2.13661 | UP |
| SCARNA6 | 2.138344 | -4.21125 | 3.524409 | 0.000998 | 0.015963 | -2.14897 | UP |
| RP11-66D17.5 | 1.331685 | -3.98179 | 3.506837 | 0.001051 | 0.016616 | -2.15317 | UP |
| CCDC177 | 2.286786 | -1.29291 | 3.511087 | 0.001038 | 0.016464 | -2.1706 | UP |
| AL513327.1 | 1.245342 | -2.61812 | 3.451274 | 0.001237 | 0.018736 | -2.18779 | UP |
| RP11-264I13.2 | 1.85403 | -4.64559 | 3.567702 | 0.000879 | 0.014534 | -2.18976 | UP |
| CFAP45 | 1.504585 | -3.47434 | 3.466453 | 0.001183 | 0.01816 | -2.19548 | UP |
| MIAT | 1.114471 | 0.300782 | 3.598498 | 0.000802 | 0.013595 | -2.19748 | UP |
| VCAN-AS1 | 1.72845 | -4.18211 | 3.505258 | 0.001056 | 0.016674 | -2.20799 | UP |
| SNORA60 | 1.45603 | -4.0832 | 3.489462 | 0.001106 | 0.017271 | -2.20887 | UP |
| FAM72B | 1.336513 | -4.04994 | 3.487136 | 0.001114 | 0.017355 | -2.22024 | UP |
| PCDH10 | 1.657552 | -3.78272 | 3.474716 | 0.001155 | 0.017848 | -2.23598 | UP |
| IL32 | 1.436097 | 6.043358 | 3.900688 | 0.000322 | 0.006811 | -2.25057 | UP |
| PLOD2 | 1.053008 | 6.039232 | 3.900146 | 0.000322 | 0.00682 | -2.25581 | UP |
| CLCN1 | 1.577749 | -3.71969 | 3.453686 | 0.001228 | 0.018634 | -2.26062 | UP |
| RPL34P18 | 1.372868 | -4.90408 | 3.601989 | 0.000794 | 0.013482 | -2.26319 | UP |
| CTD-2286N8.1 | 1.122651 | -5.12872 | 3.663485 | 0.000661 | 0.011708 | -2.26836 | UP |
| OASL | 1.079384 | 2.413953 | 3.702942 | 0.000587 | 0.010714 | -2.27298 | UP |
| RP11-21K12.3 | 1.406014 | -3.90291 | 3.459205 | 0.001208 | 0.018442 | -2.27771 | UP |
| HIF1A-AS1 | 1.491611 | -2.95275 | 3.413717 | 0.001379 | 0.020332 | -2.28033 | UP |
| RP11-59D5__B.2 | 1.518163 | -3.29468 | 3.421066 | 0.00135 | 0.020045 | -2.28267 | UP |
| CRABP2 | 1.089919 | -1.59686 | 3.454535 | 0.001225 | 0.018602 | -2.29541 | UP |
| FOXQ1 | 2.326521 | -0.31631 | 3.516093 | 0.001023 | 0.016272 | -2.3017 | UP |
| WI2-87327B8.2 | 1.493054 | -3.49566 | 3.405204 | 0.001414 | 0.020688 | -2.32107 | UP |
| CRIP1 | 1.282118 | -2.36018 | 3.40887 | 0.001399 | 0.020541 | -2.32768 | UP |
| RP11-91J19.2 | 1.944576 | -3.88454 | 3.431838 | 0.001309 | 0.019617 | -2.3384 | UP |
| AC004490.1 | 1.383015 | -2.91001 | 3.38965 | 0.001479 | 0.021409 | -2.34443 | UP |
| HIST1H2BE | 1.766358 | -4.33398 | 3.488218 | 0.00111 | 0.017314 | -2.35501 | UP |
| ANKRD18A | 1.57631 | -2.02823 | 3.410139 | 0.001394 | 0.020493 | -2.36274 | UP |
| CCL3L3 | 1.445491 | -4.22569 | 3.485971 | 0.001118 | 0.017382 | -2.36639 | UP |
| NSRP1P1 | 1.231039 | -4.5761 | 3.495684 | 0.001086 | 0.017049 | -2.37032 | UP |
| KLF10 | 1.080005 | 6.119053 | 3.861268 | 0.000363 | 0.007456 | -2.3827 | UP |
| RPL4P1 | 1.208987 | -4.70101 | 3.50898 | 0.001045 | 0.016534 | -2.38329 | UP |
| RP11-127I20.8 | 1.588404 | -3.70857 | 3.403781 | 0.00142 | 0.020763 | -2.38344 | UP |
| RP11-925D8.2 | 1.255715 | -4.85356 | 3.536109 | 0.000964 | 0.01557 | -2.41034 | UP |
| MEIOB | 1.97258 | -3.85009 | 3.39972 | 0.001436 | 0.02094 | -2.42139 | UP |
| MALL | 1.542493 | -2.28938 | 3.377788 | 0.00153 | 0.021926 | -2.42384 | UP |
| SNORA46 | 1.449719 | -3.70831 | 3.388498 | 0.001484 | 0.021459 | -2.43016 | UP |
| SNORD19 | 1.56291 | -3.09671 | 3.356841 | 0.001626 | 0.02293 | -2.43731 | UP |
| RP11-203M5.8 | 1.362123 | -0.87274 | 3.433791 | 0.001301 | 0.019546 | -2.4601 | UP |
| TPPP3 | 1.021331 | -0.23458 | 3.470403 | 0.00117 | 0.018025 | -2.46767 | UP |
| RP5-943J3.2 | 1.51303 | -3.55557 | 3.350593 | 0.001655 | 0.02325 | -2.47765 | UP |
| TREML2 | 1.471602 | -0.86185 | 3.423604 | 0.00134 | 0.019967 | -2.48876 | UP |
| CITED1 | 1.612964 | -4.38617 | 3.420549 | 0.001352 | 0.020065 | -2.48914 | UP |
| AKAP2 | 1.631299 | -1.11599 | 3.402754 | 0.001424 | 0.02082 | -2.50483 | UP |
| FAP | 1.315537 | -4.06114 | 3.382489 | 0.00151 | 0.021732 | -2.50884 | UP |
| FAM134B | 1.225945 | 5.197926 | 3.76899 | 0.000481 | 0.009259 | -2.51448 | UP |
| RAB7B | 1.35701 | -2.116 | 3.348903 | 0.001663 | 0.023335 | -2.51902 | UP |
| SCHIP1 | 1.030698 | -1.19703 | 3.395196 | 0.001455 | 0.021142 | -2.51943 | UP |
| ARHGEF34P | 1.240548 | -0.15201 | 3.454196 | 0.001226 | 0.018616 | -2.52444 | UP |
| TNFRSF18 | 1.487839 | -3.30403 | 3.321611 | 0.001799 | 0.024817 | -2.54065 | UP |
| AC000403.4 | 1.45051 | -3.87875 | 3.356558 | 0.001627 | 0.022943 | -2.54241 | UP |
| AC002401.1 | 1.640667 | -3.86759 | 3.352928 | 0.001644 | 0.023139 | -2.55088 | UP |
| KLF7-IT1 | 1.46463 | -3.22765 | 3.31427 | 0.001837 | 0.025231 | -2.5528 | UP |
| RP1-281H8.3 | 1.139995 | -4.90958 | 3.498387 | 0.001078 | 0.016949 | -2.55587 | UP |
| RP11-506K6.4 | 1.87758 | -4.13239 | 3.364066 | 0.001592 | 0.022545 | -2.55754 | UP |
| HIST1H3D | 1.649183 | -2.65988 | 3.318403 | 0.001815 | 0.024993 | -2.56121 | UP |
| KCNH4 | 1.373715 | -3.31626 | 3.308882 | 0.001865 | 0.025494 | -2.56478 | UP |
| CCL22 | 1.93589 | -3.79569 | 3.340245 | 0.001705 | 0.023763 | -2.56996 | UP |
| DHDH | 1.50758 | -2.54962 | 3.314317 | 0.001837 | 0.025231 | -2.57369 | UP |
| RP11-465L10.10 | 1.586637 | -3.17487 | 3.308336 | 0.001868 | 0.025512 | -2.57479 | UP |
| PCAT1 | 1.303647 | -4.39614 | 3.396582 | 0.00145 | 0.021084 | -2.57903 | UP |
| RN7SKP16 | 1.125562 | -1.76843 | 3.341872 | 0.001697 | 0.023674 | -2.58114 | UP |
| CTD-2616J11.16 | 1.096565 | -4.35843 | 3.433587 | 0.001302 | 0.01955 | -2.61204 | UP |
| RP5-858L17.1 | 1.463279 | -2.92615 | 3.28611 | 0.001991 | 0.026768 | -2.62799 | UP |
| LA16c-431H6.7 | 1.053409 | -2.43763 | 3.294905 | 0.001941 | 0.026275 | -2.62864 | UP |
| CTD-2357A8.2 | 1.27488 | -4.66892 | 3.413812 | 0.001379 | 0.020332 | -2.63028 | UP |
| EEF1DP3 | 1.250672 | -2.3198 | 3.299255 | 0.001917 | 0.026011 | -2.63095 | UP |
| FOXP1-IT1 | 1.608333 | -4.35074 | 3.359745 | 0.001612 | 0.022783 | -2.65125 | UP |
| RTKN2 | 1.808875 | -4.49681 | 3.375917 | 0.001539 | 0.022007 | -2.66084 | UP |
| CLEC5A | 1.605154 | -1.42837 | 3.324819 | 0.001782 | 0.024637 | -2.67276 | UP |
| RNVU1-19 | 1.501216 | -4.86475 | 3.438184 | 0.001285 | 0.019353 | -2.68877 | UP |
| AC005776.1 | 1.368181 | -4.59793 | 3.396237 | 0.001451 | 0.021098 | -2.69035 | UP |
| P2RY6 | 1.001877 | -0.18697 | 3.387619 | 0.001488 | 0.021507 | -2.70555 | UP |
| AC067945.3 | 1.264164 | -4.36925 | 3.34566 | 0.001679 | 0.02348 | -2.70708 | UP |
| FABP5 | 1.065089 | -0.14723 | 3.387541 | 0.001488 | 0.021507 | -2.7123 | UP |
| RP11-70L8.4 | 1.747445 | -4.34014 | 3.329719 | 0.001757 | 0.02434 | -2.71275 | UP |
| RP11-1036E20.7 | 2.210054 | 0.565922 | 3.424399 | 0.001337 | 0.019933 | -2.71303 | UP |
| AC007278.2 | 1.446585 | -3.91112 | 3.30198 | 0.001902 | 0.025858 | -2.71348 | UP |
| KCNS1 | 1.181833 | -4.91891 | 3.443248 | 0.001266 | 0.019119 | -2.71765 | UP |
| AP001271.3 | 1.586898 | -3.67758 | 3.268342 | 0.002094 | 0.027797 | -2.72034 | UP |
| FUT2 | 1.401345 | -4.58739 | 3.372273 | 0.001555 | 0.022182 | -2.72328 | UP |
| RP11-212I21.2 | 1.358725 | -4.32562 | 3.337586 | 0.001718 | 0.023899 | -2.72646 | UP |
| NEFM | 1.104166 | -4.40913 | 3.396169 | 0.001451 | 0.021098 | -2.7296 | UP |
| CTB-109A12.1 | 1.233297 | -4.60019 | 3.367875 | 0.001575 | 0.022381 | -2.7314 | UP |
| MIR6821 | 1.492894 | -2.95341 | 3.248525 | 0.002215 | 0.028994 | -2.73309 | UP |
| RP11-483H20.4 | 1.162752 | -4.86952 | 3.423304 | 0.001342 | 0.019967 | -2.7377 | UP |
| RP3-340N1.2 | 1.444725 | -0.2045 | 3.362098 | 0.001601 | 0.022646 | -2.76678 | UP |
| RP11-775C24.5 | 1.413444 | -1.78865 | 3.269364 | 0.002088 | 0.027741 | -2.77423 | UP |
| RP13-516M14.10 | 1.348024 | -2.85064 | 3.227007 | 0.002354 | 0.03037 | -2.78568 | UP |
| RP13-216E22.4 | 1.069916 | -2.53101 | 3.23125 | 0.002326 | 0.030083 | -2.79116 | UP |
| WNT7B | 1.543041 | -3.54862 | 3.239067 | 0.002275 | 0.029582 | -2.79465 | UP |
| PACERR | 1.145197 | -4.78032 | 3.380586 | 0.001518 | 0.021804 | -2.79555 | UP |
| AC147651.4 | 1.504129 | -2.51716 | 3.230159 | 0.002333 | 0.030148 | -2.80155 | UP |
| RP11-503N18.1 | 1.646786 | -3.85703 | 3.259159 | 0.002149 | 0.028331 | -2.80294 | UP |
| CDH6 | 1.176093 | 0.066804 | 3.366417 | 0.001581 | 0.022448 | -2.80438 | UP |
| CLEC4D | 1.549378 | -0.97765 | 3.299658 | 0.001915 | 0.025995 | -2.80789 | UP |
| ATG9B | 1.442778 | -1.75275 | 3.254422 | 0.002178 | 0.028617 | -2.81893 | UP |
| CTA-363E19.2 | 1.150219 | -4.33691 | 3.287764 | 0.001981 | 0.026673 | -2.82942 | UP |
| RN7SL336P | 1.756566 | -3.82532 | 3.244779 | 0.002238 | 0.029196 | -2.83386 | UP |
| TREML3P | 1.339506 | -3.63337 | 3.227163 | 0.002353 | 0.03037 | -2.83717 | UP |
| HIST1H3A | 1.75389 | -3.83271 | 3.245924 | 0.002231 | 0.02915 | -2.8378 | UP |
| MIR6753 | 1.169359 | -2.66202 | 3.209088 | 0.002476 | 0.03147 | -2.84121 | UP |
| RP11-797A18.6 | 1.245278 | -3.82129 | 3.242016 | 0.002256 | 0.029379 | -2.84765 | UP |
| TEX14 | 1.219323 | -0.46556 | 3.316836 | 0.001823 | 0.025087 | -2.84979 | UP |
| CTD-2542L18.1 | 1.627496 | -3.86631 | 3.241969 | 0.002256 | 0.029379 | -2.8543 | UP |
| LRRC43 | 1.169124 | -3.21164 | 3.195199 | 0.002574 | 0.032378 | -2.85751 | UP |
| WTAPP1 | 1.486208 | -3.48802 | 3.204964 | 0.002505 | 0.031749 | -2.8599 | UP |
| LOXL4 | 1.206886 | 1.628235 | 3.441729 | 0.001272 | 0.019189 | -2.87425 | UP |
| RP11-294J22.7 | 1.969742 | -4.17053 | 3.2532 | 0.002186 | 0.02869 | -2.87504 | UP |
| CTD-2357A8.3 | 1.255531 | -5.09231 | 3.434821 | 0.001297 | 0.019508 | -2.87976 | UP |
| CTC-490G23.2 | 1.507167 | -3.79388 | 3.224216 | 0.002372 | 0.030531 | -2.88112 | UP |
| RP11-295M3.4 | 1.355944 | -1.11984 | 3.262911 | 0.002126 | 0.028082 | -2.88747 | UP |
| MT1JP | 1.41104 | 0.606985 | 3.367715 | 0.001576 | 0.022385 | -2.89103 | UP |
| OR52K3P | 1.283784 | -4.58739 | 3.304619 | 0.001888 | 0.025712 | -2.89187 | UP |
| RP11-582E3.4 | 1.672108 | -4.66877 | 3.328172 | 0.001765 | 0.024442 | -2.89241 | UP |
| MCEMP1 | 1.204693 | 0.231543 | 3.340899 | 0.001702 | 0.023729 | -2.90328 | UP |
| AC078852.1 | 2.138492 | -4.54625 | 3.2941 | 0.001946 | 0.026317 | -2.90497 | UP |
| DNLZ | 1.311283 | -2.64991 | 3.17932 | 0.002692 | 0.033471 | -2.92279 | UP |
| CASC19 | 1.842283 | -3.30167 | 3.185125 | 0.002648 | 0.033086 | -2.92395 | UP |
| RP11-545P7.9 | 1.287971 | -4.29877 | 3.249999 | 0.002206 | 0.028906 | -2.93104 | UP |
| AF127936.7 | 1.242514 | -3.12149 | 3.167501 | 0.002782 | 0.03434 | -2.93194 | UP |
| RP11-616M22.7 | 1.097669 | -2.39745 | 3.180068 | 0.002686 | 0.033424 | -2.93994 | UP |
| CTC-820M8.1 | 1.719697 | -4.24321 | 3.23718 | 0.002287 | 0.029714 | -2.94144 | UP |
| RP11-537I16.2 | 1.334651 | -4.17677 | 3.236346 | 0.002292 | 0.029769 | -2.94554 | UP |
| RPL21P7 | 1.321033 | -4.2178 | 3.226451 | 0.002357 | 0.030411 | -2.94654 | UP |
| RP11-147G7.3 | 1.332743 | -4.67686 | 3.313197 | 0.001842 | 0.025278 | -2.94975 | UP |
| RP4-620E11.8 | 1.202803 | -3.36495 | 3.162106 | 0.002824 | 0.034715 | -2.95719 | UP |
| WIPF3 | 1.150249 | -0.55413 | 3.271501 | 0.002075 | 0.027649 | -2.95753 | UP |
| RP11-752L20.3 | 1.054339 | -2.17725 | 3.181573 | 0.002675 | 0.033317 | -2.9591 | UP |
| APOBEC3B | 1.325715 | -2.10317 | 3.18475 | 0.002651 | 0.033106 | -2.96091 | UP |
| ERBB4 | 1.213741 | -4.44379 | 3.256263 | 0.002167 | 0.028519 | -2.96396 | UP |
| HAP1 | 1.405337 | -3.27715 | 3.153909 | 0.00289 | 0.035326 | -2.97764 | UP |
| TTLL10 | 1.417345 | -4.95995 | 3.357528 | 0.001622 | 0.022901 | -2.98173 | UP |
| DDN | 1.857337 | -4.29987 | 3.263474 | 0.002123 | 0.028059 | -2.98281 | UP |
| RP11-69L16.5 | 1.412162 | -3.85818 | 3.193887 | 0.002584 | 0.032461 | -2.98356 | UP |
| KRT18P52 | 1.30171 | -4.91391 | 3.341059 | 0.001701 | 0.023724 | -2.98707 | UP |
| MT1L | 1.155054 | 2.720962 | 3.467761 | 0.001179 | 0.018122 | -2.99103 | UP |
| RP11-161M6.2 | 1.391611 | -2.30631 | 3.16225 | 0.002823 | 0.034715 | -2.99829 | UP |
| PPIAP2 | 1.436627 | -4.76877 | 3.30312 | 0.001896 | 0.025804 | -3.00601 | UP |
| CD200 | 1.394439 | -3.36464 | 3.141931 | 0.002988 | 0.036192 | -3.01006 | UP |
| RAB33A | 1.370872 | -2.58821 | 3.137809 | 0.003023 | 0.036481 | -3.03761 | UP |
| RP11-378J18.9 | 1.238836 | -3.59333 | 3.139408 | 0.003009 | 0.036363 | -3.04083 | UP |
| CTD-2562G15.3 | 1.988569 | -3.90416 | 3.160792 | 0.002835 | 0.034825 | -3.05597 | UP |
| RP13-638C3.2 | 1.481214 | -2.22995 | 3.139929 | 0.003005 | 0.03632 | -3.06666 | UP |
| PI3 | 1.564474 | -3.2021 | 3.12416 | 0.00314 | 0.037605 | -3.07473 | UP |
| HIST2H2BF | 1.224418 | -2.92012 | 3.103506 | 0.003325 | 0.039289 | -3.1048 | UP |
| TVP23CP1 | 1.131915 | -5.02856 | 3.331596 | 0.001748 | 0.024233 | -3.10498 | UP |
| DHRS9 | 1.209161 | -0.04961 | 3.246651 | 0.002227 | 0.029104 | -3.10854 | UP |
| PLEKHG4B | 1.455736 | -3.3191 | 3.10537 | 0.003308 | 0.039142 | -3.10863 | UP |
| CTC-1337H24.4 | 1.303477 | -4.64932 | 3.231229 | 0.002326 | 0.030083 | -3.11313 | UP |
| SPTBN5 | 1.310286 | 1.518504 | 3.345784 | 0.001678 | 0.023477 | -3.11422 | UP |
| FBN3 | 1.279668 | -4.11794 | 3.160874 | 0.002834 | 0.034825 | -3.11457 | UP |
| ULBP1 | 1.629569 | -2.81564 | 3.102771 | 0.003331 | 0.039342 | -3.12412 | UP |
| MTND4P14 | 1.069687 | -4.85388 | 3.276094 | 0.002048 | 0.027403 | -3.12699 | UP |
| RP11-126K1.6 | 1.101386 | -4.71639 | 3.239326 | 0.002273 | 0.029573 | -3.12929 | UP |
| RP11-317F20.3 | 1.153265 | -4.8462 | 3.272701 | 0.002068 | 0.027583 | -3.13257 | UP |
| WNT10A | 1.459264 | -4.71643 | 3.237767 | 0.002283 | 0.029671 | -3.13318 | UP |
| RP5-837J1.4 | 1.186354 | -5.04382 | 3.311411 | 0.001852 | 0.025372 | -3.15921 | UP |
| RP11-297C4.2 | 1.212565 | -4.62525 | 3.205094 | 0.002504 | 0.031749 | -3.17123 | UP |
| AQP7P2 | 1.336831 | -3.88466 | 3.12341 | 0.003146 | 0.037652 | -3.17544 | UP |
| RGS4 | 1.084706 | 0.324367 | 3.245328 | 0.002235 | 0.029179 | -3.1786 | UP |
| SYN1 | 1.171904 | 0.661572 | 3.265478 | 0.002111 | 0.027947 | -3.18273 | UP |
| STPG2 | 1.401615 | -3.69297 | 3.097745 | 0.003378 | 0.039784 | -3.18432 | UP |
| FBXW4P1 | 1.282806 | -1.72823 | 3.116762 | 0.003205 | 0.038201 | -3.18641 | UP |
| GS1-166A23.2 | 1.331888 | -4.74171 | 3.217481 | 0.002418 | 0.030903 | -3.20596 | UP |
| SCUBE3 | 1.265127 | -2.45124 | 3.076675 | 0.003581 | 0.041539 | -3.20665 | UP |
| ACKR3 | 1.476593 | 3.267876 | 3.418077 | 0.001362 | 0.020163 | -3.2125 | UP |
| CTD-3099C6.9 | 1.17637 | -2.17438 | 3.083694 | 0.003512 | 0.040958 | -3.21621 | UP |
| MATN4 | 1.676846 | -2.82328 | 3.067666 | 0.00367 | 0.04235 | -3.21659 | UP |
| RP11-367G6.3 | 1.44931 | -4.00782 | 3.111082 | 0.003256 | 0.038669 | -3.21804 | UP |
| RP11-131M11.3 | 1.173357 | -4.56349 | 3.172097 | 0.002747 | 0.034017 | -3.23184 | UP |
| AIM1L | 1.426318 | -3.52575 | 3.071031 | 0.003637 | 0.042014 | -3.24016 | UP |
| RP11-44N11.1 | 1.242315 | -4.29663 | 3.13456 | 0.00305 | 0.036762 | -3.24267 | UP |
| TMEM71 | 1.011891 | 0.310103 | 3.216777 | 0.002423 | 0.030938 | -3.2531 | UP |
| SOX2-OT | 1.675764 | -3.71673 | 3.075685 | 0.00359 | 0.04161 | -3.25718 | UP |
| RP11-2C24.4 | 1.102548 | -5.12617 | 3.309261 | 0.001863 | 0.02548 | -3.26192 | UP |
| RASEF | 1.145497 | 1.024262 | 3.25795 | 0.002156 | 0.028409 | -3.26598 | UP |
| DUOX2 | 2.462177 | -2.39442 | 3.065248 | 0.003695 | 0.042503 | -3.27226 | UP |
| RP11-267J23.1 | 1.091565 | -4.59998 | 3.160653 | 0.002836 | 0.034827 | -3.27622 | UP |
| CCDC173 | 1.457527 | -2.2121 | 3.059255 | 0.003756 | 0.043014 | -3.27717 | UP |
| LA16c-306A4.2 | 1.517849 | -4.71718 | 3.18424 | 0.002655 | 0.033132 | -3.27735 | UP |
| CTA-373H7.7 | 1.28968 | -3.53269 | 3.048124 | 0.003873 | 0.044081 | -3.28177 | UP |
| CD177 | 1.311865 | 1.389329 | 3.271284 | 0.002076 | 0.027655 | -3.29579 | UP |
| RPS20P33 | 1.318834 | -4.00117 | 3.074677 | 0.0036 | 0.041701 | -3.29751 | UP |
| CTB-25B13.5 | 1.147084 | -3.89936 | 3.065482 | 0.003693 | 0.042499 | -3.31555 | UP |
| CABYR | 1.490539 | -2.6633 | 3.027939 | 0.004093 | 0.045941 | -3.31955 | UP |
| MTCYBP4 | 1.432333 | -4.55464 | 3.136457 | 0.003034 | 0.036611 | -3.32198 | UP |
| LINC00313 | 1.443417 | -3.36831 | 3.026136 | 0.004113 | 0.046097 | -3.32313 | UP |
| DNAJA1P3 | 1.173055 | -2.64797 | 3.022563 | 0.004154 | 0.046425 | -3.32799 | UP |
| CRPP1 | 2.122759 | -2.17477 | 3.042162 | 0.003937 | 0.044581 | -3.33598 | UP |
| DKK1 | 1.668345 | -3.60013 | 3.024908 | 0.004127 | 0.04619 | -3.34762 | UP |
| SNORD46 | 1.535474 | -3.58466 | 3.020443 | 0.004178 | 0.04661 | -3.3489 | UP |
| RP11-386J22.3 | 1.137287 | -4.76328 | 3.168106 | 0.002777 | 0.034304 | -3.3505 | UP |
| RNU4-80P | 1.009447 | -5.13105 | 3.276894 | 0.002043 | 0.027347 | -3.35567 | UP |
| RP11-619A14.2 | 1.070876 | -4.92231 | 3.209291 | 0.002474 | 0.031459 | -3.35779 | UP |
| MT1P1 | 1.113741 | -2.17419 | 3.028367 | 0.004088 | 0.045923 | -3.3581 | UP |
| ACKR1 | 1.54389 | -3.25964 | 3.006729 | 0.004337 | 0.047832 | -3.36552 | UP |
| IPO4 | 1.35429 | -2.18578 | 3.022828 | 0.004151 | 0.046409 | -3.37243 | UP |
| KCNN1 | 1.331329 | -4.67469 | 3.139917 | 0.003005 | 0.03632 | -3.37482 | UP |
| RP11-1379J22.2 | 1.117393 | -1.99647 | 3.025595 | 0.00412 | 0.04613 | -3.38674 | UP |
| LONRF2 | 1.153057 | 1.19934 | 3.218668 | 0.00241 | 0.030861 | -3.40491 | UP |
| ST6GALNAC4 | 1.123753 | 2.454944 | 3.298505 | 0.001921 | 0.026061 | -3.41214 | UP |
| LGALS2 | 1.58453 | -3.70204 | 3.011044 | 0.004286 | 0.047489 | -3.42547 | UP |
| C2CD4D | 1.090064 | -4.60437 | 3.104729 | 0.003313 | 0.039196 | -3.42723 | UP |
| SNORA33 | 1.051734 | -2.04297 | 3.003539 | 0.004375 | 0.04815 | -3.43701 | UP |
| AC079354.5 | 1.512937 | -4.54201 | 3.087288 | 0.003477 | 0.040648 | -3.44436 | UP |
| ANKRD37 | 1.30064 | 2.645467 | 3.295025 | 0.001941 | 0.026272 | -3.44955 | UP |
| FCER1A | 1.463923 | -4.4252 | 3.067414 | 0.003673 | 0.042354 | -3.47313 | UP |
| RGS8 | 1.088696 | -4.26415 | 3.090971 | 0.003442 | 0.040365 | -3.47719 | UP |
| RP11-383B4.4 | 1.133899 | -1.85996 | 2.996841 | 0.004456 | 0.048824 | -3.47765 | UP |
| PGK1P2 | 1.435651 | -3.71595 | 2.988169 | 0.004562 | 0.049634 | -3.478 | UP |
| KLF2 | 1.277669 | 4.216623 | 3.374216 | 0.001546 | 0.022088 | -3.48415 | UP |
| RP1-20B21.4 | 1.271519 | -3.94528 | 3.003852 | 0.004371 | 0.048127 | -3.48647 | UP |
| HDC | 1.339011 | -1.84387 | 2.987222 | 0.004574 | 0.049718 | -3.50419 | UP |
| CTB-119C2.1 | 1.000802 | -0.73413 | 3.052332 | 0.003829 | 0.04366 | -3.50718 | UP |
| PARD3-AS1 | 1.44574 | -4.32697 | 3.012436 | 0.00427 | 0.047372 | -3.53397 | UP |
| GPR85 | 1.047083 | -0.78851 | 3.037399 | 0.003989 | 0.045013 | -3.53734 | UP |
| PADI4 | 1.43551 | 1.097241 | 3.151422 | 0.00291 | 0.035505 | -3.55948 | UP |
| SLITRK3 | 1.205159 | 3.745144 | 3.307162 | 0.001875 | 0.025568 | -3.59566 | UP |
| AC137932.6 | 1.210413 | -4.24183 | 3.027646 | 0.004097 | 0.045969 | -3.61146 | UP |
| RP11-396O20.2 | 1.604555 | -4.7267 | 3.06101 | 0.003738 | 0.042875 | -3.61988 | UP |
| PARD6G-AS1 | 1.228515 | 0.515339 | 3.079954 | 0.003548 | 0.041254 | -3.64617 | UP |
| IL2RA | 1.44586 | 0.798816 | 3.088723 | 0.003463 | 0.040541 | -3.66979 | UP |
| AC011343.1 | 1.019355 | -5.02332 | 3.102729 | 0.003332 | 0.039342 | -3.7195 | UP |
| GPRIN3 | 1.092125 | 2.21614 | 3.165916 | 0.002795 | 0.034457 | -3.72535 | UP |
| RP11-355N15.1 | 1.326187 | -4.84322 | 3.042284 | 0.003936 | 0.044581 | -3.73206 | UP |
| ACTL7B | 1.044998 | -5.10432 | 3.110651 | 0.003259 | 0.038699 | -3.77568 | UP |
| RP11-91K9.1 | 1.039318 | 0.845319 | 3.046859 | 0.003886 | 0.044199 | -3.79265 | UP |
| SLC16A8 | 1.188093 | -4.74898 | 2.99233 | 0.004511 | 0.049253 | -3.79268 | UP |
| RNA5SP404 | 1.387247 | -4.87279 | 3.018956 | 0.004195 | 0.04675 | -3.81391 | UP |
| GAPDHP62 | 1.411937 | -4.92939 | 3.035289 | 0.004012 | 0.045218 | -3.8157 | UP |
| CYP51A1P3 | 1.431631 | -5.16067 | 3.098102 | 0.003375 | 0.039776 | -3.86066 | UP |
| HIST1H3I | 1.471633 | -4.87319 | 2.987133 | 0.004575 | 0.049718 | -3.89484 | UP |
| LCN2 | 1.182899 | 3.673253 | 3.156267 | 0.002871 | 0.035146 | -3.9884 | UP |
| NR4A2 | 1.722676 | 3.758551 | 3.063714 | 0.003711 | 0.042674 | -4.23138 | UP |
| MT2A | 1.043151 | 9.834913 | 3.391871 | 0.001469 | 0.021304 | -4.27051 | UP |
| MT1A | 1.431462 | 4.163928 | 3.063162 | 0.003716 | 0.042714 | -4.30365 | UP |
| EGR1 | 1.71911 | 7.855967 | 3.140839 | 0.002997 | 0.036257 | -4.64541 | UP |
| GIMAP7 | -1.34318 | 2.096442 | -8.94998 | 1.70E-11 | 2.06E-08 | 15.06343 | DOWN |
| LINC01089 | -1.39979 | 2.027765 | -7.97201 | 4.19E-10 | 1.82E-07 | 11.84596 | DOWN |
| CTC-429P9.3 | -1.11694 | 0.785877 | -7.90839 | 5.18E-10 | 2.12E-07 | 11.8372 | DOWN |
| FAM212A | -2.73476 | -2.31818 | -7.18791 | 5.81E-09 | 1.44E-06 | 9.745802 | DOWN |
| PPP1R3E | -1.0094 | 3.046588 | -7.36261 | 3.22E-09 | 9.05E-07 | 9.616582 | DOWN |
| ZNF552 | -1.0325 | 1.380604 | -7.22373 | 5.15E-09 | 1.31E-06 | 9.426537 | DOWN |
| C14orf132 | -1.28312 | 2.762854 | -7.06531 | 8.80E-09 | 1.97E-06 | 8.659823 | DOWN |
| GIMAP1 | -1.02517 | 1.665517 | -6.97489 | 1.20E-08 | 2.44E-06 | 8.533099 | DOWN |
| AP006621.6 | -2.47856 | -1.51645 | -6.8246 | 1.99E-08 | 3.59E-06 | 8.528998 | DOWN |
| RP11-74E22.3 | -2.66195 | -2.81089 | -6.80312 | 2.14E-08 | 3.76E-06 | 8.451186 | DOWN |
| HAND2 | -1.89588 | 2.309168 | -6.94018 | 1.34E-08 | 2.64E-06 | 8.331491 | DOWN |
| ADCY1 | -1.35424 | 6.315775 | -6.96663 | 1.23E-08 | 2.48E-06 | 7.784707 | DOWN |
| RNA5SP163 | -2.61914 | -2.99283 | -6.58022 | 4.57E-08 | 6.72E-06 | 7.700296 | DOWN |
| GS1-358P8.4 | -1.20594 | 2.83176 | -6.77345 | 2.37E-08 | 4.05E-06 | 7.655365 | DOWN |
| TBC1D16 | -1.07894 | 5.429587 | -6.87023 | 1.70E-08 | 3.17E-06 | 7.583372 | DOWN |
| GIMAP8 | -1.02311 | 3.590093 | -6.77563 | 2.35E-08 | 4.03E-06 | 7.538714 | DOWN |
| BNIPL | -2.70572 | -2.38743 | -6.47167 | 6.60E-08 | 8.85E-06 | 7.321152 | DOWN |
| RIPPLY3 | -2.13525 | -3.54091 | -6.40727 | 8.22E-08 | 1.05E-05 | 7.131747 | DOWN |
| RP11-77K12.9 | -1.59261 | -0.97526 | -6.34934 | 1.00E-07 | 1.20E-05 | 6.852154 | DOWN |
| RP11-1109F11.3 | -2.35056 | -3.13467 | -6.2839 | 1.25E-07 | 1.44E-05 | 6.714743 | DOWN |
| ZFP14 | -1.41957 | 0.870598 | -6.36325 | 9.55E-08 | 1.17E-05 | 6.599311 | DOWN |
| CTD-2227E11.1 | -2.24931 | -1.33454 | -6.24445 | 1.43E-07 | 1.59E-05 | 6.547841 | DOWN |
| CTD-2299I21.1 | -3.00042 | -2.46625 | -6.23815 | 1.46E-07 | 1.61E-05 | 6.504615 | DOWN |
| RP11-1151B14.3 | -1.40818 | 4.910062 | -6.49329 | 6.14E-08 | 8.38E-06 | 6.384128 | DOWN |
| GPR34 | -1.38153 | 1.977256 | -6.3231 | 1.09E-07 | 1.28E-05 | 6.273892 | DOWN |
| SH3GL1P1 | -2.33019 | -2.81986 | -6.14587 | 2.00E-07 | 2.06E-05 | 6.241336 | DOWN |
| MIR143HG | -2.25692 | -0.55525 | -6.16948 | 1.85E-07 | 1.92E-05 | 6.192236 | DOWN |
| RP11-21L23.4 | -2.55075 | -1.86647 | -6.12227 | 2.17E-07 | 2.19E-05 | 6.161693 | DOWN |
| RP11-394O4.5 | -3.09505 | 0.358538 | -6.19284 | 1.70E-07 | 1.82E-05 | 6.159126 | DOWN |
| RP11-476H16.1 | -2.03903 | -3.23062 | -6.11074 | 2.25E-07 | 2.26E-05 | 6.14826 | DOWN |
| LINC00939 | -1.98648 | 0.673197 | -6.21268 | 1.59E-07 | 1.73E-05 | 6.14001 | DOWN |
| SIT1 | -2.18239 | -3.08061 | -6.09828 | 2.35E-07 | 2.33E-05 | 6.095302 | DOWN |
| RP11-263G22.1 | -2.73701 | -2.80962 | -6.10439 | 2.30E-07 | 2.30E-05 | 6.076499 | DOWN |
| CMB9-94B1.2 | -1.96459 | -3.9766 | -6.08964 | 2.42E-07 | 2.38E-05 | 6.016176 | DOWN |
| RP11-295H24.4 | -2.07918 | -3.5627 | -6.0654 | 2.63E-07 | 2.54E-05 | 5.98133 | DOWN |
| CMB9-55F22.1 | -1.77923 | 0.632196 | -6.13434 | 2.08E-07 | 2.13E-05 | 5.875915 | DOWN |
| RP11-197N18.2 | -2.24041 | -3.42918 | -6.03135 | 2.95E-07 | 2.79E-05 | 5.86211 | DOWN |
| FAM78A | -1.02001 | 1.477836 | -6.1679 | 1.86E-07 | 1.92E-05 | 5.823645 | DOWN |
| HNF1A-AS1 | -1.49221 | 2.149525 | -6.18574 | 1.75E-07 | 1.85E-05 | 5.780225 | DOWN |
| RP4-695O20.1 | -2.54489 | -2.73086 | -6.01018 | 3.17E-07 | 2.97E-05 | 5.769908 | DOWN |
| HAND2-AS1 | -1.19496 | 2.01933 | -6.13078 | 2.11E-07 | 2.15E-05 | 5.608062 | DOWN |
| RP11-715J22.6 | -1.35861 | 0.372097 | -5.9934 | 3.36E-07 | 3.09E-05 | 5.429113 | DOWN |
| RP11-998D10.1 | -1.23755 | -4.75636 | -5.88771 | 4.81E-07 | 4.10E-05 | 5.192987 | DOWN |
| RP11-21L23.3 | -2.00616 | -3.03866 | -5.81713 | 6.11E-07 | 4.98E-05 | 5.156319 | DOWN |
| ZNF345 | -1.14236 | 0.325229 | -5.90573 | 4.52E-07 | 3.89E-05 | 5.138295 | DOWN |
| SLC25A30-AS1 | -2.24839 | -2.81677 | -5.75763 | 7.47E-07 | 5.83E-05 | 4.937004 | DOWN |
| PKI55 | -1.29948 | 0.527459 | -5.84489 | 5.56E-07 | 4.60E-05 | 4.900804 | DOWN |
| HOXB4 | -1.1286 | -0.05425 | -5.80875 | 6.28E-07 | 5.08E-05 | 4.874477 | DOWN |
| RP4-583P15.15 | -1.52099 | -0.68251 | -5.74305 | 7.85E-07 | 6.09E-05 | 4.759994 | DOWN |
| HOXB3 | -1.42344 | 1.079711 | -5.81404 | 6.17E-07 | 5.01E-05 | 4.706859 | DOWN |
| CXCR2P1 | -1.32647 | 1.924752 | -5.84781 | 5.50E-07 | 4.56E-05 | 4.670573 | DOWN |
| TDRD6 | -1.82707 | -0.48796 | -5.71629 | 8.59E-07 | 6.55E-05 | 4.646891 | DOWN |
| RP11-250B2.5 | -2.01224 | -1.50276 | -5.66808 | 1.01E-06 | 7.41E-05 | 4.622991 | DOWN |
| DICER1-AS1 | -1.25953 | -0.01685 | -5.70079 | 9.05E-07 | 6.84E-05 | 4.508717 | DOWN |
| TSC22D1-AS1 | -2.04719 | -3.42082 | -5.57489 | 1.39E-06 | 9.50E-05 | 4.343515 | DOWN |
| RP1-20C7.6 | -1.98529 | -3.62392 | -5.55421 | 1.49E-06 | 0.0001 | 4.258933 | DOWN |
| C9orf139 | -1.79119 | -1.73329 | -5.55012 | 1.51E-06 | 0.000101 | 4.25277 | DOWN |
| RP11-131L12.3 | -2.25581 | -3.30161 | -5.52597 | 1.63E-06 | 0.000107 | 4.170383 | DOWN |
| SLC22A25 | -1.23313 | 3.826171 | -5.77563 | 7.03E-07 | 5.56E-05 | 4.113135 | DOWN |
| SHC3 | -2.17975 | -2.69308 | -5.49647 | 1.80E-06 | 0.000116 | 4.070368 | DOWN |
| RP11-390P24.1 | -1.45117 | -0.42826 | -5.54756 | 1.52E-06 | 0.000102 | 4.065458 | DOWN |
| LPIN3 | -1.0969 | 5.894118 | -5.84935 | 5.47E-07 | 4.55E-05 | 4.047553 | DOWN |
| RP11-10K16.1 | -1.8768 | -3.58837 | -5.46069 | 2.04E-06 | 0.000128 | 3.966399 | DOWN |
| U3 | -2.32491 | -2.85421 | -5.45019 | 2.11E-06 | 0.000131 | 3.908535 | DOWN |
| RP11-535A5.1 | -1.67931 | -3.81659 | -5.44337 | 2.16E-06 | 0.000134 | 3.90453 | DOWN |
| CTD-3076M17.1 | -1.79072 | 0.366083 | -5.51312 | 1.71E-06 | 0.000111 | 3.826325 | DOWN |
| TRIM60P18 | -1.9915 | -3.39576 | -5.38908 | 2.59E-06 | 0.000155 | 3.730942 | DOWN |
| CCDC30 | -1.24254 | -1.1508 | -5.40093 | 2.49E-06 | 0.00015 | 3.68279 | DOWN |
| C1QTNF3 | -1.06449 | 0.110301 | -5.45741 | 2.06E-06 | 0.000129 | 3.669408 | DOWN |
| RP11-544L8__B.4 | -2.02253 | -3.42717 | -5.36793 | 2.78E-06 | 0.000164 | 3.656605 | DOWN |
| RAD51-AS1 | -1.24794 | 0.164316 | -5.42942 | 2.26E-06 | 0.000139 | 3.570924 | DOWN |
| CD3G | -2.32769 | -2.57911 | -5.34365 | 3.02E-06 | 0.000174 | 3.561412 | DOWN |
| IGIP | -1.08279 | 1.795665 | -5.50709 | 1.74E-06 | 0.000113 | 3.54306 | DOWN |
| MIR639 | -1.61197 | -4.97284 | -5.44431 | 2.15E-06 | 0.000134 | 3.494216 | DOWN |
| LINC00264 | -2.07909 | -2.74832 | -5.31651 | 3.30E-06 | 0.000189 | 3.478952 | DOWN |
| MS4A4E | -1.60464 | -1.51097 | -5.32268 | 3.23E-06 | 0.000186 | 3.473624 | DOWN |
| RP11-426C22.1 | -2.20137 | -3.18968 | -5.2846 | 3.67E-06 | 0.000206 | 3.372873 | DOWN |
| NIPSNAP3B | -1.31007 | -1.65783 | -5.2791 | 3.74E-06 | 0.000208 | 3.347179 | DOWN |
| CAHM | -1.90152 | -3.2169 | -5.24667 | 4.17E-06 | 0.000227 | 3.264226 | DOWN |
| ZNF169 | -1.19367 | -0.73247 | -5.28904 | 3.62E-06 | 0.000203 | 3.248673 | DOWN |
| SLC10A4 | -1.40956 | -5.04444 | -5.36516 | 2.81E-06 | 0.000165 | 3.196923 | DOWN |
| DCLK3 | -2.21761 | -2.8886 | -5.22679 | 4.46E-06 | 0.00024 | 3.175115 | DOWN |
| EEF1DP4 | -1.67318 | -4.93924 | -5.34444 | 3.01E-06 | 0.000174 | 3.165434 | DOWN |
| RP11-392O17.2 | -2.05004 | -3.59277 | -5.18484 | 5.13E-06 | 0.000268 | 3.042206 | DOWN |
| NAPSA | -1.89056 | -1.4561 | -5.18285 | 5.16E-06 | 0.00027 | 3.006115 | DOWN |
| CMKLR1 | -1.14127 | 3.13648 | -5.41208 | 2.40E-06 | 0.000146 | 3.002017 | DOWN |
| RP11-98D18.9 | -2.11132 | -3.03155 | -5.15429 | 5.68E-06 | 0.000291 | 2.945175 | DOWN |
| FMR1-IT1 | -1.92735 | -3.83655 | -5.16017 | 5.57E-06 | 0.000287 | 2.935898 | DOWN |
| AC016995.3 | -1.04879 | -4.9025 | -5.23044 | 4.40E-06 | 0.000238 | 2.930292 | DOWN |
| Z97634.3 | -1.98213 | -2.93258 | -5.14799 | 5.80E-06 | 0.000295 | 2.928944 | DOWN |
| CPO | -2.01062 | -3.2279 | -5.12821 | 6.19E-06 | 0.000311 | 2.870376 | DOWN |
| AP000320.6 | -2.07879 | -2.72826 | -5.1291 | 6.18E-06 | 0.000311 | 2.861937 | DOWN |
| RP11-57H14.4 | -1.40287 | 0.214638 | -5.21619 | 4.62E-06 | 0.000247 | 2.857959 | DOWN |
| OSGEPL1-AS1 | -1.81376 | -3.69896 | -5.11605 | 6.45E-06 | 0.000321 | 2.828348 | DOWN |
| STRADBP1 | -1.38597 | -5.03496 | -5.25448 | 4.06E-06 | 0.000223 | 2.821747 | DOWN |
| SMAD6 | -1.159 | 3.493566 | -5.37098 | 2.75E-06 | 0.000163 | 2.808152 | DOWN |
| TRAV1-2 | -1.81625 | -3.98994 | -5.10677 | 6.65E-06 | 0.00033 | 2.738566 | DOWN |
| RP11-347I19.8 | -1.90356 | -3.88068 | -5.10031 | 6.80E-06 | 0.000335 | 2.724704 | DOWN |
| KCNJ10 | -1.559 | -0.16503 | -5.15098 | 5.74E-06 | 0.000293 | 2.709002 | DOWN |
| RP11-622A1.1 | -1.93733 | -4.15324 | -5.10551 | 6.68E-06 | 0.000331 | 2.703189 | DOWN |
| ZNF214 | -1.24189 | -0.20962 | -5.14433 | 5.87E-06 | 0.000298 | 2.688473 | DOWN |
| RP11-119F7.5 | -2.09201 | -2.70663 | -5.07418 | 7.41E-06 | 0.000355 | 2.686744 | DOWN |
| RP11-94C24.13 | -2.23939 | -2.94605 | -5.07627 | 7.36E-06 | 0.000353 | 2.682412 | DOWN |
| NEURL1B | -1.04439 | 4.204785 | -5.36426 | 2.81E-06 | 0.000165 | 2.67401 | DOWN |
| RP1-80N2.3 | -1.91599 | -2.9071 | -5.05638 | 7.87E-06 | 0.000372 | 2.629288 | DOWN |
| RP11-23J9.5 | -1.0991 | -0.81841 | -5.09729 | 6.87E-06 | 0.000336 | 2.628843 | DOWN |
| RP11-522B15.3 | -1.64799 | -1.94761 | -5.04607 | 8.14E-06 | 0.000382 | 2.607644 | DOWN |
| ZNF491 | -1.6691 | -1.17143 | -5.06637 | 7.61E-06 | 0.000363 | 2.586951 | DOWN |
| RP5-1068E13.7 | -1.65199 | -1.91767 | -5.01993 | 8.88E-06 | 0.00041 | 2.516112 | DOWN |
| C9orf173-AS1 | -1.81846 | 1.447707 | -5.16678 | 5.45E-06 | 0.000282 | 2.494315 | DOWN |
| GULOP | -1.71072 | -3.76692 | -5.00537 | 9.32E-06 | 0.000424 | 2.462225 | DOWN |
| VIPR1-AS1 | -1.51771 | -2.15532 | -4.98766 | 9.88E-06 | 0.000445 | 2.43581 | DOWN |
| RP11-70D24.3 | -1.49872 | -4.23753 | -5.0125 | 9.10E-06 | 0.000418 | 2.422218 | DOWN |
| CTD-2278I10.4 | -1.84988 | -3.30998 | -4.98211 | 1.01E-05 | 0.00045 | 2.401577 | DOWN |
| CTD-2224J9.8 | -1.93115 | -3.32912 | -4.97993 | 1.01E-05 | 0.000453 | 2.388978 | DOWN |
| RP1-278C19.8 | -1.94433 | -3.4565 | -4.97823 | 1.02E-05 | 0.000455 | 2.380208 | DOWN |
| AP000439.1 | -2.00765 | -3.21467 | -4.96818 | 1.05E-05 | 0.000468 | 2.345848 | DOWN |
| RP11-84C13.2 | -2.11674 | -2.83692 | -4.96929 | 1.05E-05 | 0.000467 | 2.334425 | DOWN |
| CTC-366B18.4 | -1.85523 | -3.28019 | -4.96071 | 1.08E-05 | 0.000478 | 2.332485 | DOWN |
| CTD-2240J17.2 | -2.39188 | -2.73805 | -4.966 | 1.06E-05 | 0.000471 | 2.304902 | DOWN |
| SIRPG | -1.78561 | -2.90748 | -4.91326 | 1.26E-05 | 0.000541 | 2.177196 | DOWN |
| RP1-47M23.3 | -2.0545 | -2.9056 | -4.91589 | 1.25E-05 | 0.000538 | 2.168672 | DOWN |
| RP11-520H14.7 | -1.66766 | -4.29398 | -4.93545 | 1.17E-05 | 0.00051 | 2.144951 | DOWN |
| ZNF287 | -1.7431 | -2.07378 | -4.89844 | 1.33E-05 | 0.000563 | 2.134004 | DOWN |
| RP11-513M16.8 | -1.98779 | -2.9146 | -4.90193 | 1.31E-05 | 0.000558 | 2.123888 | DOWN |
| SNX15 | -1.36851 | -0.89945 | -4.93595 | 1.17E-05 | 0.00051 | 2.118281 | DOWN |
| NHLRC4 | -1.86159 | -3.43027 | -4.88796 | 1.37E-05 | 0.000578 | 2.095421 | DOWN |
| RPS4XP16 | -1.9158 | -3.46183 | -4.88283 | 1.40E-05 | 0.000585 | 2.076774 | DOWN |
| P2RY13 | -1.05643 | 2.418588 | -5.09552 | 6.91E-06 | 0.000337 | 2.07095 | DOWN |
| RP11-731C17.2 | -1.58786 | -1.9858 | -4.86826 | 1.47E-05 | 0.00061 | 2.030965 | DOWN |
| SKIDA1 | -1.33131 | 1.04335 | -5.00736 | 9.25E-06 | 0.000422 | 2.028422 | DOWN |
| RP11-395I6.3 | -1.64814 | -2.44126 | -4.83825 | 1.62E-05 | 0.000659 | 1.955335 | DOWN |
| RP11-389C8.2 | -1.10028 | 0.207319 | -4.93669 | 1.17E-05 | 0.000509 | 1.937163 | DOWN |
| RP11-173P15.5 | -1.85335 | -3.0643 | -4.83653 | 1.63E-05 | 0.000662 | 1.926141 | DOWN |
| CTD-2528L19.6 | -1.79793 | -2.36082 | -4.82649 | 1.68E-05 | 0.000681 | 1.909096 | DOWN |
| AP000472.2 | -1.19525 | -0.30985 | -4.88768 | 1.37E-05 | 0.000578 | 1.865794 | DOWN |
| BMP4 | -1.18892 | 0.568042 | -4.92942 | 1.20E-05 | 0.000517 | 1.85342 | DOWN |
| MLLT4-AS1 | -1.6381 | -0.92987 | -4.85111 | 1.55E-05 | 0.000639 | 1.852042 | DOWN |
| RP11-666A8.8 | -1.86618 | -3.49355 | -4.81273 | 1.76E-05 | 0.000699 | 1.850085 | DOWN |
| RP11-579D7.2 | -1.92624 | -3.21286 | -4.80996 | 1.77E-05 | 0.000704 | 1.838401 | DOWN |
| SPIC | -2.05018 | -1.56772 | -4.82034 | 1.72E-05 | 0.000689 | 1.83512 | DOWN |
| CCER2 | -1.85416 | -2.84811 | -4.80378 | 1.81E-05 | 0.000716 | 1.821668 | DOWN |
| RNF43 | -1.37486 | 3.322777 | -5.05999 | 7.77E-06 | 0.000369 | 1.812789 | DOWN |
| TUBA3FP | -1.86782 | -2.51056 | -4.78939 | 1.90E-05 | 0.000744 | 1.785139 | DOWN |
| ZNF630 | -1.04602 | -0.8043 | -4.83423 | 1.64E-05 | 0.000666 | 1.770782 | DOWN |
| ADAM20P1 | -1.95815 | -3.06395 | -4.78555 | 1.92E-05 | 0.000752 | 1.755885 | DOWN |
| AP000695.6 | -1.12834 | -0.31886 | -4.85084 | 1.55E-05 | 0.000639 | 1.747329 | DOWN |
| LRRC66 | -1.95438 | -3.23551 | -4.77877 | 1.97E-05 | 0.000765 | 1.736196 | DOWN |
| RP4-694B14.8 | -1.74879 | -3.46436 | -4.77145 | 2.01E-05 | 0.000777 | 1.72522 | DOWN |
| RP11-408A13.4 | -1.915 | -3.15876 | -4.76717 | 2.04E-05 | 0.000785 | 1.701174 | DOWN |
| ZNF594 | -1.17744 | -0.07457 | -4.84194 | 1.60E-05 | 0.000653 | 1.679264 | DOWN |
| XXbac-B444P24.13 | -1.87121 | -3.48706 | -4.75337 | 2.14E-05 | 0.000815 | 1.659239 | DOWN |
| BGLAP | -2.07331 | -2.88049 | -4.75821 | 2.10E-05 | 0.000805 | 1.654213 | DOWN |
| MIR503HG | -1.52132 | 1.287553 | -4.90224 | 1.31E-05 | 0.000558 | 1.647613 | DOWN |
| RP11-1055B8.4 | -1.32038 | 0.319393 | -4.83809 | 1.62E-05 | 0.000659 | 1.601844 | DOWN |
| RPS18P12 | -1.54988 | -2.49505 | -4.70542 | 2.50E-05 | 0.000924 | 1.535702 | DOWN |
| LINC00271 | -1.696 | -3.16104 | -4.71115 | 2.45E-05 | 0.000913 | 1.531395 | DOWN |
| Y_RNA | -1.79757 | -3.45442 | -4.7086 | 2.47E-05 | 0.000919 | 1.521284 | DOWN |
| FFAR4 | -1.87094 | -3.43768 | -4.70225 | 2.53E-05 | 0.000932 | 1.496285 | DOWN |
| RP11-383C6.2 | -1.08021 | -5.26636 | -4.90783 | 1.29E-05 | 0.00055 | 1.476808 | DOWN |
| RP11-365D23.4 | -1.69837 | -4.88133 | -4.81782 | 1.73E-05 | 0.000692 | 1.474035 | DOWN |
| NFYC-AS1 | -1.06396 | -0.66561 | -4.74618 | 2.19E-05 | 0.000829 | 1.464792 | DOWN |
| OMG | -2.06 | -3.10811 | -4.69096 | 2.62E-05 | 0.000959 | 1.449718 | DOWN |
| DLEU1 | -1.05508 | 0.461832 | -4.75997 | 2.09E-05 | 0.000802 | 1.322428 | DOWN |
| RP11-774O3.3 | -1.04868 | 0.197384 | -4.74164 | 2.22E-05 | 0.000839 | 1.308112 | DOWN |
| RP11-569G13.3 | -1.73479 | -3.78917 | -4.63394 | 3.15E-05 | 0.001112 | 1.267423 | DOWN |
| C8orf31 | -1.52619 | -3.93755 | -4.63498 | 3.14E-05 | 0.001109 | 1.26609 | DOWN |
| RP11-523H20.3 | -2.01649 | -3.16608 | -4.62652 | 3.23E-05 | 0.001135 | 1.247018 | DOWN |
| ST6GAL2 | -1.65049 | 1.475683 | -4.78175 | 1.95E-05 | 0.000759 | 1.232123 | DOWN |
| CTD-2561B21.8 | -1.79563 | -1.76901 | -4.62329 | 3.27E-05 | 0.001145 | 1.225679 | DOWN |
| ABC12-49244600F4.4 | -1.18099 | -0.78752 | -4.66253 | 2.87E-05 | 0.001035 | 1.217428 | DOWN |
| RP4-647C14.2 | -1.52014 | -2.60797 | -4.60416 | 3.47E-05 | 0.001199 | 1.216464 | DOWN |
| TMEM240 | -1.71612 | -3.45992 | -4.60593 | 3.46E-05 | 0.001194 | 1.197749 | DOWN |
| GS1-114I9.1 | -1.84623 | -3.06345 | -4.59801 | 3.55E-05 | 0.001214 | 1.160585 | DOWN |
| AC005363.11 | -1.46147 | -5.04154 | -4.73091 | 2.30E-05 | 0.000864 | 1.104242 | DOWN |
| PNMT | -1.99665 | -3.22822 | -4.57146 | 3.86E-05 | 0.001303 | 1.074295 | DOWN |
| RP11-295I5.3 | -1.8186 | -3.36997 | -4.56077 | 4.00E-05 | 0.001343 | 1.050304 | DOWN |
| TBC1D29 | -1.89964 | -3.17508 | -4.5592 | 4.02E-05 | 0.001349 | 1.03953 | DOWN |
| MIR3936 | -1.80553 | -3.08982 | -4.5567 | 4.05E-05 | 0.001359 | 1.034716 | DOWN |
| RP11-244O19.1 | -1.40627 | 0.103957 | -4.64395 | 3.05E-05 | 0.001082 | 1.018603 | DOWN |
| RP11-617F23.1 | -1.03338 | 2.628312 | -4.77951 | 1.96E-05 | 0.000764 | 1.005887 | DOWN |
| RP5-901A4.1 | -1.61978 | -4.9284 | -4.66101 | 2.89E-05 | 0.001039 | 0.93747 | DOWN |
| SALL2 | -1.49566 | -0.78352 | -4.57266 | 3.85E-05 | 0.001299 | 0.936769 | DOWN |
| RP3-431P23.5 | -1.55484 | 0.071358 | -4.61178 | 3.39E-05 | 0.001177 | 0.921979 | DOWN |
| RP5-1056H1.2 | -1.0986 | -5.20086 | -4.71571 | 2.42E-05 | 0.0009 | 0.920925 | DOWN |
| AL358852.1 | -1.64234 | -3.18318 | -4.51404 | 4.65E-05 | 0.001505 | 0.908425 | DOWN |
| FLJ46284 | -1.79818 | -3.04919 | -4.51093 | 4.70E-05 | 0.001517 | 0.887984 | DOWN |
| RP3-400B16.1 | -1.68063 | -3.21438 | -4.5046 | 4.80E-05 | 0.001544 | 0.878594 | DOWN |
| KLF15 | -1.17266 | 6.359622 | -4.9151 | 1.26E-05 | 0.000539 | 0.875173 | DOWN |
| CTB-191K22.6 | -1.70892 | -3.64191 | -4.50093 | 4.85E-05 | 0.00156 | 0.859568 | DOWN |
| RP5-1092A3.4 | -1.89359 | -0.79179 | -4.53582 | 4.34E-05 | 0.001424 | 0.826377 | DOWN |
| LRRC4 | -1.15321 | 1.949791 | -4.67325 | 2.78E-05 | 0.001007 | 0.783734 | DOWN |
| CTD-2651B20.4 | -1.85378 | -3.54056 | -4.47329 | 5.31E-05 | 0.001675 | 0.769838 | DOWN |
| SCT | -1.40146 | -5.16245 | -4.63779 | 3.12E-05 | 0.001101 | 0.74403 | DOWN |
| CTSLP7 | -1.76041 | -3.43691 | -4.46147 | 5.51E-05 | 0.001726 | 0.739172 | DOWN |
| CALML3-AS1 | -2.12585 | -2.84031 | -4.46059 | 5.53E-05 | 0.00173 | 0.706778 | DOWN |
| LINC00639 | -1.67772 | -3.2241 | -4.44948 | 5.73E-05 | 0.001783 | 0.705562 | DOWN |
| UBXN10-AS1 | -1.23345 | -5.07108 | -4.61637 | 3.34E-05 | 0.001164 | 0.704754 | DOWN |
| RP1-168L15.5 | -1.87748 | -3.51842 | -4.43931 | 5.92E-05 | 0.001834 | 0.661278 | DOWN |
| RP4-561L24.3 | -1.77468 | -3.11857 | -4.4362 | 5.98E-05 | 0.001845 | 0.658212 | DOWN |
| RP11-37C7.3 | -1.97951 | -2.32077 | -4.43584 | 5.98E-05 | 0.001846 | 0.653685 | DOWN |
| RP11-156E8.1 | -1.48711 | -2.12768 | -4.42894 | 6.12E-05 | 0.001875 | 0.648967 | DOWN |
| RP11-15A1.8 | -1.60614 | -3.92052 | -4.43546 | 5.99E-05 | 0.001847 | 0.6295 | DOWN |
| CECR7 | -1.67244 | -4.86435 | -4.5377 | 4.31E-05 | 0.001417 | 0.623809 | DOWN |
| LHX2 | -1.02003 | 1.695187 | -4.60457 | 3.47E-05 | 0.001198 | 0.606134 | DOWN |
| ZNF366 | -1.1515 | 0.23149 | -4.51882 | 4.58E-05 | 0.001489 | 0.595129 | DOWN |
| KLHL33 | -1.7791 | -3.87271 | -4.42758 | 6.14E-05 | 0.001879 | 0.580732 | DOWN |
| RP11-981G7.1 | -1.61695 | -4.0803 | -4.43182 | 6.06E-05 | 0.001865 | 0.579219 | DOWN |
| RP11-197N18.7 | -1.94445 | -3.49486 | -4.41372 | 6.42E-05 | 0.001947 | 0.575068 | DOWN |
| RP11-66N11.8 | -1.65683 | -2.94631 | -4.39702 | 6.78E-05 | 0.002035 | 0.540944 | DOWN |
| RGSL1 | -1.68453 | -4.01751 | -4.40897 | 6.52E-05 | 0.001969 | 0.509066 | DOWN |
| HOXA4 | -1.6051 | -3.1535 | -4.38062 | 7.14E-05 | 0.002125 | 0.491196 | DOWN |
| RP11-267M23.4 | -1.64576 | -3.64119 | -4.37657 | 7.24E-05 | 0.002144 | 0.474754 | DOWN |
| RP11-3M1.1 | -1.85538 | -3.28013 | -4.37686 | 7.23E-05 | 0.002143 | 0.470777 | DOWN |
| LINC00563 | -1.65081 | -3.35906 | -4.37099 | 7.37E-05 | 0.002173 | 0.461948 | DOWN |
| RP1-193H18.2 | -1.38696 | -0.99149 | -4.41212 | 6.46E-05 | 0.001955 | 0.460757 | DOWN |
| RP11-258C19.7 | -1.03822 | 0.395337 | -4.48035 | 5.19E-05 | 0.001647 | 0.443106 | DOWN |
| FAM162B | -1.77402 | -3.17078 | -4.36115 | 7.60E-05 | 0.002221 | 0.424139 | DOWN |
| LIME1 | -1.2944 | 1.943776 | -4.55497 | 4.08E-05 | 0.001362 | 0.412518 | DOWN |
| ADRA2B | -1.17263 | 2.921832 | -4.60786 | 3.43E-05 | 0.001188 | 0.411031 | DOWN |
| RP11-371I20.2 | -1.73343 | -3.86601 | -4.36676 | 7.47E-05 | 0.002193 | 0.403178 | DOWN |
| RN7SL541P | -1.1569 | -4.96897 | -4.49845 | 4.89E-05 | 0.00157 | 0.399866 | DOWN |
| RP11-316M1.3 | -1.62349 | -3.77581 | -4.34843 | 7.92E-05 | 0.002299 | 0.376362 | DOWN |
| CD160 | -1.68272 | -2.22688 | -4.33221 | 8.34E-05 | 0.002397 | 0.346721 | DOWN |
| RP11-434B12.1 | -1.07665 | -0.70947 | -4.38982 | 6.93E-05 | 0.002072 | 0.342618 | DOWN |
| AC006273.5 | -1.81864 | -3.73801 | -4.33691 | 8.21E-05 | 0.002369 | 0.332723 | DOWN |
| TBX2-AS1 | -1.61419 | -2.91221 | -4.32555 | 8.51E-05 | 0.002439 | 0.3248 | DOWN |
| M1AP | -1.75183 | -3.30892 | -4.32121 | 8.63E-05 | 0.002469 | 0.30247 | DOWN |
| RP11-1020A11.1 | -1.69611 | -3.35906 | -4.31876 | 8.70E-05 | 0.002485 | 0.297967 | DOWN |
| B3GALT2 | -1.18613 | -1.37844 | -4.34043 | 8.12E-05 | 0.002345 | 0.290997 | DOWN |
| RP11-430C7.4 | -1.75174 | -3.40786 | -4.30702 | 9.03E-05 | 0.002547 | 0.258858 | DOWN |
| RP11-401P9.5 | -1.79829 | -3.3222 | -4.30781 | 9.01E-05 | 0.002546 | 0.258714 | DOWN |
| RPSAP31 | -1.3056 | -5.06089 | -4.46815 | 5.39E-05 | 0.001697 | 0.257822 | DOWN |
| CAPZA3 | -1.56777 | -4.21714 | -4.33511 | 8.26E-05 | 0.00238 | 0.257054 | DOWN |
| RP11-429K17.1 | -1.60049 | -3.53916 | -4.30157 | 9.19E-05 | 0.002587 | 0.248409 | DOWN |
| RP5-882C2.2 | -1.13932 | -0.85187 | -4.34137 | 8.10E-05 | 0.00234 | 0.216029 | DOWN |
| C21orf58 | -1.11308 | -0.22097 | -4.3701 | 7.39E-05 | 0.002176 | 0.203772 | DOWN |
| RP11-179K3.2 | -1.79493 | -3.19886 | -4.28764 | 9.61E-05 | 0.002673 | 0.196538 | DOWN |
| RP11-435F17.3 | -1.66058 | -3.11572 | -4.28526 | 9.68E-05 | 0.002688 | 0.192799 | DOWN |
| CTD-2621I17.3 | -1.50891 | -1.68236 | -4.29552 | 9.37E-05 | 0.002618 | 0.191796 | DOWN |
| RP11-185E8.2 | -1.68213 | -3.2698 | -4.28421 | 9.71E-05 | 0.002693 | 0.190917 | DOWN |
| LINC00883 | -1.16683 | -1.14651 | -4.31279 | 8.87E-05 | 0.002519 | 0.171846 | DOWN |
| RP11-690I21.2 | -1.76621 | -3.56011 | -4.27416 | 0.0001 | 0.002762 | 0.155904 | DOWN |
| AC009120.3 | -1.83641 | -2.83493 | -4.27473 | 0.0001 | 0.002758 | 0.153763 | DOWN |
| PCAT14 | -1.89983 | -3.92452 | -4.28822 | 9.59E-05 | 0.002672 | 0.127811 | DOWN |
| RP11-517H2.6 | -1.46371 | -3.86238 | -4.26198 | 0.000104 | 0.002847 | 0.113348 | DOWN |
| MESTIT1 | -1.59083 | -3.90242 | -4.26182 | 0.000104 | 0.002847 | 0.098945 | DOWN |
| RP1-40G4P.1 | -1.26805 | -4.20865 | -4.27056 | 0.000101 | 0.002785 | 0.093029 | DOWN |
| CCDC180 | -1.79077 | -1.69472 | -4.25855 | 0.000105 | 0.002868 | 0.077316 | DOWN |
| RP5-1057J7.7 | -1.55759 | -2.18473 | -4.24957 | 0.000108 | 0.002933 | 0.074649 | DOWN |
| RP11-96D1.7 | -1.69377 | -3.0488 | -4.24692 | 0.000109 | 0.00295 | 0.069919 | DOWN |
| RP11-767N6.7 | -1.32434 | -1.99913 | -4.24429 | 0.00011 | 0.002968 | 0.068508 | DOWN |
| RP11-650K20.3 | -1.62906 | -3.34684 | -4.24135 | 0.000111 | 0.002986 | 0.062587 | DOWN |
| LOC100421166 | -1.808 | -1.99673 | -4.24562 | 0.00011 | 0.002961 | 0.056891 | DOWN |
| RP4-583P15.10 | -1.71735 | -3.12857 | -4.23857 | 0.000112 | 0.003009 | 0.044267 | DOWN |
| FAM65C | -1.03799 | 4.299847 | -4.55517 | 4.07E-05 | 0.001362 | 0.024315 | DOWN |
| CTB-12O2.1 | -1.11693 | -5.00298 | -4.38975 | 6.94E-05 | 0.002072 | 0.020972 | DOWN |
| SLC26A10 | -1.4257 | -1.18887 | -4.25442 | 0.000107 | 0.002897 | -0.00063 | DOWN |
| LRCOL1 | -1.26523 | 2.31365 | -4.4383 | 5.94E-05 | 0.001837 | -0.02159 | DOWN |
| NAP1L3 | -1.52227 | -0.30299 | -4.28707 | 9.62E-05 | 0.002677 | -0.03432 | DOWN |
| RP11-131K5.2 | -1.79019 | -2.95691 | -4.21075 | 0.000123 | 0.003232 | -0.04677 | DOWN |
| RP11-800A3.4 | -1.21576 | 0.806729 | -4.34572 | 7.98E-05 | 0.002317 | -0.0489 | DOWN |
| RUNX1T1 | -1.08348 | 0.417908 | -4.3146 | 8.82E-05 | 0.002508 | -0.07841 | DOWN |
| RP11-181G12.4 | -1.63141 | -3.83366 | -4.20263 | 0.000126 | 0.003299 | -0.09223 | DOWN |
| AC006277.2 | -1.51708 | -3.38913 | -4.18855 | 0.000131 | 0.003398 | -0.09397 | DOWN |
| LRRC69 | -1.65373 | -2.42282 | -4.18486 | 0.000133 | 0.003433 | -0.10184 | DOWN |
| RP11-834C11.7 | -1.53905 | -4.21238 | -4.21612 | 0.00012 | 0.003189 | -0.1116 | DOWN |
| KARSP2 | -1.32756 | -1.14335 | -4.2116 | 0.000122 | 0.003227 | -0.13869 | DOWN |
| MANEA-AS1 | -1.06349 | -1.68624 | -4.18345 | 0.000134 | 0.003437 | -0.15184 | DOWN |
| RP3-508I15.9 | -1.45606 | -2.25103 | -4.16596 | 0.000141 | 0.003589 | -0.15532 | DOWN |
| RP11-1081L13.4 | -1.77449 | -3.09776 | -4.17412 | 0.000138 | 0.003517 | -0.15539 | DOWN |
| NOXRED1 | -1.15732 | -2.35281 | -4.15962 | 0.000144 | 0.003642 | -0.15899 | DOWN |
| RPL29P24 | -1.21141 | -4.53085 | -4.2124 | 0.000122 | 0.003221 | -0.18611 | DOWN |
| RP11-33O4.1 | -1.132 | -1.25363 | -4.19107 | 0.00013 | 0.003377 | -0.18801 | DOWN |
| RP11-459I19.1 | -1.93651 | -2.00379 | -4.16502 | 0.000142 | 0.003595 | -0.19107 | DOWN |
| AC002117.1 | -1.14099 | -1.37693 | -4.18351 | 0.000134 | 0.003437 | -0.19341 | DOWN |
| RP4-621B10.8 | -1.13698 | -4.70235 | -4.23079 | 0.000115 | 0.003073 | -0.19586 | DOWN |
| RP11-439E19.9 | -1.52378 | -2.26253 | -4.15333 | 0.000147 | 0.003704 | -0.19646 | DOWN |
| TPPP2 | -1.266 | 0.048746 | -4.25068 | 0.000108 | 0.002924 | -0.21066 | DOWN |
| RP11-318K12.3 | -1.68821 | -4.8622 | -4.26957 | 0.000102 | 0.002789 | -0.22207 | DOWN |
| RP11-370B11.3 | -1.44374 | -4.84786 | -4.25552 | 0.000106 | 0.002891 | -0.22238 | DOWN |
| AP003774.4 | -1.47057 | -4.09926 | -4.16639 | 0.000141 | 0.003586 | -0.22581 | DOWN |
| RP11-188P17.2 | -1.55984 | -3.92111 | -4.15509 | 0.000146 | 0.003688 | -0.22682 | DOWN |
| RP11-278C7.3 | -1.57195 | -3.23777 | -4.14242 | 0.000152 | 0.003805 | -0.23859 | DOWN |
| FLRT2 | -1.01715 | 1.115137 | -4.28798 | 9.60E-05 | 0.002673 | -0.28444 | DOWN |
| APC2 | -1.63874 | -2.76373 | -4.12083 | 0.000163 | 0.004015 | -0.29565 | DOWN |
| CTD-2649C14.2 | -1.32762 | -1.52973 | -4.13923 | 0.000153 | 0.003835 | -0.3067 | DOWN |
| RP11-333E1.1 | -1.00428 | -0.87394 | -4.17054 | 0.000139 | 0.003547 | -0.30939 | DOWN |
| HSPD1P11 | -1.64193 | -2.98211 | -4.11375 | 0.000166 | 0.00408 | -0.32757 | DOWN |
| C1QTNF4 | -1.7144 | -3.05175 | -4.11575 | 0.000165 | 0.004062 | -0.33076 | DOWN |
| RP11-477D19.2 | -1.19836 | -1.4152 | -4.12495 | 0.000161 | 0.003983 | -0.36631 | DOWN |
| RP11-666O2.2 | -1.70362 | -3.07822 | -4.10315 | 0.000172 | 0.00419 | -0.36835 | DOWN |
| DYNLRB2 | -1.44629 | -3.78879 | -4.0941 | 0.000177 | 0.004287 | -0.389 | DOWN |
| RPL29P11 | -1.09621 | -5.04753 | -4.24919 | 0.000109 | 0.002935 | -0.41403 | DOWN |
| AC002550.6 | -1.42887 | -4.75896 | -4.18414 | 0.000133 | 0.003433 | -0.41884 | DOWN |
| ZNF208 | -1.56436 | -3.49113 | -4.07603 | 0.000187 | 0.004479 | -0.43901 | DOWN |
| LINC01558 | -1.36116 | 1.493351 | -4.25333 | 0.000107 | 0.002904 | -0.45049 | DOWN |
| ZNF415 | -1.03824 | -0.49845 | -4.14205 | 0.000152 | 0.003808 | -0.45489 | DOWN |
| RP5-1112D6.8 | -1.13655 | -0.83681 | -4.12201 | 0.000162 | 0.004008 | -0.46112 | DOWN |
| LINC01124 | -1.24477 | -0.46622 | -4.14003 | 0.000153 | 0.003827 | -0.46357 | DOWN |
| DGCR12 | -1.7208 | -2.98653 | -4.069 | 0.000191 | 0.004561 | -0.47246 | DOWN |
| RP11-127B20.3 | -1.57486 | -3.55168 | -4.06504 | 0.000194 | 0.004602 | -0.47303 | DOWN |
| IGHV3-33 | -1.69825 | -3.52839 | -4.0653 | 0.000193 | 0.004601 | -0.47837 | DOWN |
| NANOGP5 | -1.43016 | -5.02737 | -4.22462 | 0.000117 | 0.003122 | -0.47975 | DOWN |
| RP11-175B9.3 | -1.50323 | -4.91219 | -4.20259 | 0.000126 | 0.003299 | -0.48869 | DOWN |
| RP11-686D22.10 | -1.44011 | -3.92609 | -4.06406 | 0.000194 | 0.004611 | -0.50075 | DOWN |
| GPR68 | -1.46215 | -3.31147 | -4.05214 | 0.000202 | 0.004746 | -0.50684 | DOWN |
| PDCD6IPP2 | -1.64048 | -3.73075 | -4.05441 | 0.0002 | 0.004728 | -0.52207 | DOWN |
| RP11-855A2.2 | -1.60257 | -3.67721 | -4.04184 | 0.000208 | 0.004875 | -0.55042 | DOWN |
| CPA5 | -1.22938 | -5.12665 | -4.22659 | 0.000117 | 0.003104 | -0.55722 | DOWN |
| RPL34-AS1 | -1.63692 | -3.38252 | -4.03684 | 0.000211 | 0.004926 | -0.55919 | DOWN |
| RP4-592A1.2 | -1.30149 | -3.17356 | -4.0327 | 0.000214 | 0.004976 | -0.55919 | DOWN |
| ASB16 | -1.47808 | -3.87361 | -4.03775 | 0.000211 | 0.004914 | -0.57935 | DOWN |
| RP11-218F10.3 | -1.71343 | -3.15452 | -4.03062 | 0.000215 | 0.004996 | -0.58483 | DOWN |
| ISLR2 | -1.57058 | -3.81907 | -4.0345 | 0.000213 | 0.00495 | -0.58684 | DOWN |
| CDC42P4 | -1.23617 | -4.87955 | -4.16246 | 0.000143 | 0.003614 | -0.59167 | DOWN |
| RP11-343J3.2 | -1.07972 | -5.05156 | -4.19327 | 0.000129 | 0.003362 | -0.59825 | DOWN |
| RP5-1074L1.4 | -1.49876 | -1.51491 | -4.04079 | 0.000209 | 0.004889 | -0.60638 | DOWN |
| RPL19P11 | -1.45272 | -4.79383 | -4.12522 | 0.00016 | 0.003982 | -0.63362 | DOWN |
| RP11-655M14.13 | -1.62521 | -3.98742 | -4.02669 | 0.000218 | 0.005046 | -0.64818 | DOWN |
| ANKRD23 | -1.13127 | -1.25597 | -4.03895 | 0.00021 | 0.004902 | -0.64953 | DOWN |
| RPL23AP46 | -1.04513 | -5.07119 | -4.18465 | 0.000133 | 0.003433 | -0.65586 | DOWN |
| ST20-MTHFS | -1.43219 | -4.80505 | -4.11496 | 0.000166 | 0.00407 | -0.65985 | DOWN |
| CYSRT1 | -1.26913 | -1.59419 | -4.01555 | 0.000226 | 0.005192 | -0.67257 | DOWN |
| FAAHP1 | -1.48316 | -3.9822 | -4.00056 | 0.000237 | 0.005399 | -0.70692 | DOWN |
| GPR123-AS1 | -1.64865 | -3.75672 | -3.99403 | 0.000241 | 0.005486 | -0.70875 | DOWN |
| RP11-399K21.13 | -1.21377 | -5.13595 | -4.17537 | 0.000137 | 0.003506 | -0.72238 | DOWN |
| CTC-559E9.1 | -1.32562 | -0.95585 | -4.02638 | 0.000218 | 0.005049 | -0.7293 | DOWN |
| RP11-152N13.5 | -1.62593 | -2.95495 | -3.97982 | 0.000252 | 0.005688 | -0.7322 | DOWN |
| AC092171.4 | -1.61864 | -2.66666 | -3.97424 | 0.000257 | 0.005758 | -0.7346 | DOWN |
| AC105760.2 | -1.692 | -3.0311 | -3.97741 | 0.000254 | 0.005723 | -0.74809 | DOWN |
| RP11-588G21.2 | -1.32383 | -4.94201 | -4.12086 | 0.000163 | 0.004015 | -0.75469 | DOWN |
| CXXC4 | -1.21132 | 0.111993 | -4.07429 | 0.000188 | 0.004497 | -0.76029 | DOWN |
| RP11-18C24.8 | -1.40125 | -3.898 | -3.97063 | 0.00026 | 0.005802 | -0.77275 | DOWN |
| RBMS3-AS3 | -1.46052 | -3.92544 | -3.97297 | 0.000258 | 0.00577 | -0.77318 | DOWN |
| LAT | -1.74853 | -2.85503 | -3.96621 | 0.000263 | 0.005853 | -0.77682 | DOWN |
| XXbac-BPGBPG55C20.2 | -1.61767 | -2.33468 | -3.95766 | 0.00027 | 0.005967 | -0.78571 | DOWN |
| RP11-235E17.4 | -1.44004 | -3.63511 | -3.95863 | 0.000269 | 0.005962 | -0.78921 | DOWN |
| RP11-394I13.2 | -1.45643 | -3.84949 | -3.96498 | 0.000264 | 0.005871 | -0.79046 | DOWN |
| ASIC1 | -1.80784 | -2.93016 | -3.96357 | 0.000265 | 0.005894 | -0.79614 | DOWN |
| C14orf180 | -1.56382 | -1.71591 | -3.96827 | 0.000261 | 0.005832 | -0.80011 | DOWN |
| RP5-881L22.5 | -2.45643 | -2.07354 | -3.96852 | 0.000261 | 0.005829 | -0.80223 | DOWN |
| RP11-234G16.5 | -1.25725 | -4.93412 | -4.10415 | 0.000171 | 0.004183 | -0.80371 | DOWN |
| CTD-2376I4.1 | -1.60659 | -3.1381 | -3.95628 | 0.000271 | 0.005982 | -0.8042 | DOWN |
| RP11-97O12.6 | -1.56029 | -2.07493 | -3.95789 | 0.00027 | 0.005966 | -0.80456 | DOWN |
| RP11-715J22.2 | -1.46694 | -4.3063 | -3.99253 | 0.000243 | 0.005505 | -0.80825 | DOWN |
| RP11-83N9.5 | -1.45215 | -3.55752 | -3.95038 | 0.000276 | 0.006072 | -0.81195 | DOWN |
| TRAPPC3L | -1.55413 | -2.90924 | -3.94738 | 0.000279 | 0.00611 | -0.82086 | DOWN |
| HSPA8P15 | -1.44375 | -3.84492 | -3.9508 | 0.000276 | 0.006067 | -0.82571 | DOWN |
| GS1-293C5.1 | -1.70392 | -2.26044 | -3.94341 | 0.000282 | 0.006169 | -0.83505 | DOWN |
| ZNF257 | -1.61422 | -3.13306 | -3.93927 | 0.000286 | 0.006237 | -0.85415 | DOWN |
| CTD-2619J13.19 | -1.50229 | -4.01489 | -3.95125 | 0.000275 | 0.006063 | -0.85423 | DOWN |
| RP1-120G22.11 | -1.45612 | -2.61066 | -3.93031 | 0.000294 | 0.006364 | -0.85462 | DOWN |
| KCNH7 | -1.56066 | -1.00479 | -3.98111 | 0.000251 | 0.00567 | -0.85526 | DOWN |
| FAM47E | -1.25673 | -0.74381 | -3.99299 | 0.000242 | 0.005499 | -0.86314 | DOWN |
| LINC01573 | -1.70176 | -1.22059 | -3.96716 | 0.000262 | 0.005843 | -0.86518 | DOWN |
| RP11-330L19.2 | -1.23484 | -4.80568 | -4.04819 | 0.000204 | 0.004797 | -0.86885 | DOWN |
| CTB-92J24.2 | -1.77116 | -2.99029 | -3.93147 | 0.000293 | 0.006346 | -0.89 | DOWN |
| RP11-69M1.6 | -1.5072 | -3.06575 | -3.92441 | 0.000299 | 0.006449 | -0.89273 | DOWN |
| ZBTB22 | -1.51622 | -3.38732 | -3.91895 | 0.000304 | 0.006537 | -0.90702 | DOWN |
| LINC01372 | -1.45266 | -3.79302 | -3.92195 | 0.000302 | 0.006486 | -0.90902 | DOWN |
| RP11-455G16.1 | -1.51756 | -4.81558 | -4.03611 | 0.000212 | 0.004933 | -0.91681 | DOWN |
| MIR6848 | -1.20378 | -5.1114 | -4.09393 | 0.000177 | 0.004287 | -0.9254 | DOWN |
| PPP5D1 | -1.58562 | -2.9974 | -3.90482 | 0.000318 | 0.006759 | -0.9516 | DOWN |
| ESRRB | -1.60955 | -3.63033 | -3.90333 | 0.000319 | 0.00677 | -0.96285 | DOWN |
| RP11-680H20.1 | -1.2995 | -3.78312 | -3.89723 | 0.000325 | 0.00686 | -0.97164 | DOWN |
| BTF3P12 | -1.34974 | -4.85925 | -4.0278 | 0.000217 | 0.005032 | -0.98571 | DOWN |
| CTC-523E23.5 | -1.47413 | -4.02307 | -3.90825 | 0.000315 | 0.006708 | -0.98621 | DOWN |
| RP11-701H16.4 | -1.61596 | -2.93576 | -3.89151 | 0.000331 | 0.006951 | -0.99014 | DOWN |
| OR10J6P | -1.20847 | 3.057431 | -4.16536 | 0.000141 | 0.003594 | -0.99207 | DOWN |
| SLC25A34 | -1.20801 | 2.515234 | -4.13562 | 0.000155 | 0.003872 | -0.99408 | DOWN |
| RP11-816J8.1 | -1.37245 | -5.02871 | -4.05921 | 0.000197 | 0.004668 | -0.99481 | DOWN |
| RP11-595B24.2 | -1.63065 | -3.20342 | -3.891 | 0.000332 | 0.006954 | -0.99742 | DOWN |
| AC007193.8 | -1.5048 | -3.22264 | -3.88363 | 0.000339 | 0.007057 | -1.013 | DOWN |
| RNASE13 | -1.4855 | -3.7826 | -3.88578 | 0.000337 | 0.007028 | -1.0169 | DOWN |
| HOXC4 | -1.42078 | -3.99879 | -3.89269 | 0.00033 | 0.00693 | -1.01724 | DOWN |
| ZNF30-AS1 | -1.35845 | -4.223 | -3.90377 | 0.000319 | 0.006769 | -1.02499 | DOWN |
| HSD3BP2 | -1.50781 | -4.86051 | -4.00219 | 0.000235 | 0.005376 | -1.03824 | DOWN |
| AP000355.2 | -1.09347 | 1.824847 | -4.08277 | 0.000183 | 0.004418 | -1.03966 | DOWN |
| RP11-944C7.1 | -1.75224 | -4.83258 | -4.00374 | 0.000234 | 0.005354 | -1.0414 | DOWN |
| RP11-10N16.3 | -1.42258 | -4.23464 | -3.88467 | 0.000338 | 0.007039 | -1.08884 | DOWN |
| RNY4P7 | -1.03152 | -5.28587 | -4.10279 | 0.000172 | 0.004193 | -1.09275 | DOWN |
| RP11-472B18.1 | -1.43032 | -2.62423 | -3.84708 | 0.000379 | 0.007699 | -1.0983 | DOWN |
| BX255923.2 | -1.80387 | -4.8715 | -3.98531 | 0.000248 | 0.00561 | -1.1027 | DOWN |
| CTD-2517M22.17 | -1.03104 | -1.46729 | -3.87019 | 0.000353 | 0.007304 | -1.12325 | DOWN |
| ZNF534 | -1.26071 | -4.74941 | -3.94324 | 0.000282 | 0.00617 | -1.13597 | DOWN |
| LINC01529 | -1.49931 | -2.00799 | -3.84485 | 0.000382 | 0.007744 | -1.13679 | DOWN |
| RP11-80A15.1 | -1.57084 | -3.65491 | -3.84266 | 0.000384 | 0.00779 | -1.13941 | DOWN |
| CBLN4 | -1.76785 | -3.09245 | -3.84518 | 0.000382 | 0.007739 | -1.14101 | DOWN |
| CTB-50L17.8 | -1.46405 | -2.63414 | -3.83247 | 0.000397 | 0.007959 | -1.1413 | DOWN |
| RP3-476K8.3 | -1.32229 | -4.94647 | -3.98546 | 0.000248 | 0.005609 | -1.14366 | DOWN |
| RP11-318E3.9 | -1.62239 | -3.10635 | -3.84145 | 0.000386 | 0.007803 | -1.14454 | DOWN |
| UBA52P6 | -1.0277 | -5.01764 | -4.00521 | 0.000233 | 0.005336 | -1.14779 | DOWN |
| RPL14P3 | -1.33668 | -3.66151 | -3.83561 | 0.000393 | 0.007905 | -1.14886 | DOWN |
| RP3-342P20.2 | -1.74403 | 1.246492 | -4.00474 | 0.000233 | 0.00534 | -1.15498 | DOWN |
| RP11-399K21.14 | -1.22082 | -2.50124 | -3.81759 | 0.000415 | 0.008231 | -1.17715 | DOWN |
| MTND4P20 | -1.99434 | -0.24758 | -3.90843 | 0.000314 | 0.006708 | -1.18194 | DOWN |
| CTD-2574D22.2 | -1.30733 | -2.40113 | -3.82965 | 0.0004 | 0.008009 | -1.18216 | DOWN |
| PTPRVP | -1.33095 | -1.97888 | -3.82609 | 0.000404 | 0.008068 | -1.19106 | DOWN |
| GZMK | -1.18622 | -0.92212 | -3.87392 | 0.000349 | 0.007236 | -1.19115 | DOWN |
| ASCL2 | -1.16215 | -1.92197 | -3.82713 | 0.000403 | 0.008055 | -1.1918 | DOWN |
| PAXBP1-AS1 | -1.07816 | -1.25224 | -3.85314 | 0.000372 | 0.007595 | -1.2039 | DOWN |
| MSS51 | -1.2022 | -1.13538 | -3.85504 | 0.00037 | 0.007554 | -1.2145 | DOWN |
| RFPL4A | -1.86839 | -3.21337 | -3.81748 | 0.000415 | 0.008231 | -1.22663 | DOWN |
| CTD-2248H3.1 | -1.57048 | -3.60158 | -3.80952 | 0.000425 | 0.008407 | -1.23381 | DOWN |
| RP11-269F19.2 | -1.36328 | -4.95942 | -3.96788 | 0.000262 | 0.005834 | -1.23464 | DOWN |
| THRB-AS1 | -1.14506 | -0.8733 | -3.8583 | 0.000367 | 0.007502 | -1.24581 | DOWN |
| RPS28P7 | -1.46817 | -3.3657 | -3.80216 | 0.000435 | 0.008551 | -1.24941 | DOWN |
| KCNJ2-AS1 | -1.47711 | -3.14909 | -3.79789 | 0.000441 | 0.008638 | -1.2657 | DOWN |
| VN1R83P | -1.3251 | -3.83972 | -3.79928 | 0.000439 | 0.008612 | -1.26608 | DOWN |
| CTD-2589H19.6 | -1.50907 | -2.82166 | -3.79205 | 0.000448 | 0.008753 | -1.26638 | DOWN |
| SLC25A41 | -1.69664 | -3.19141 | -3.79866 | 0.00044 | 0.008625 | -1.27166 | DOWN |
| RN7SKP70 | -1.34917 | -3.88516 | -3.78771 | 0.000454 | 0.008848 | -1.30448 | DOWN |
| TAPT1-AS1 | -1.18927 | -0.51612 | -3.85581 | 0.000369 | 0.007544 | -1.30978 | DOWN |
| RPSAP9 | -1.49469 | -3.35277 | -3.78048 | 0.000464 | 0.008999 | -1.31427 | DOWN |
| RP1-300G12.2 | -1.24968 | -4.86031 | -3.91391 | 0.000309 | 0.006615 | -1.32487 | DOWN |
| RP11-503C24.6 | -1.35203 | -0.7645 | -3.83471 | 0.000394 | 0.007922 | -1.32844 | DOWN |
| TXNIP | -1.1316 | 7.321453 | -4.26448 | 0.000103 | 0.002828 | -1.33512 | DOWN |
| C6orf52 | -1.51523 | -3.38541 | -3.77163 | 0.000477 | 0.009198 | -1.33953 | DOWN |
| RNU6-312P | -1.30411 | -3.92728 | -3.77531 | 0.000472 | 0.009114 | -1.34321 | DOWN |
| HIPK1-AS1 | -1.35455 | -2.71124 | -3.76028 | 0.000494 | 0.009432 | -1.34691 | DOWN |
| RP4-657E11.10 | -1.33337 | -3.64431 | -3.76084 | 0.000493 | 0.009425 | -1.36747 | DOWN |
| RP11-1376P16.2 | -1.61758 | -3.2402 | -3.7607 | 0.000493 | 0.009426 | -1.37742 | DOWN |
| RP11-173A6.2 | -1.57368 | -4.85906 | -3.89639 | 0.000326 | 0.006869 | -1.37869 | DOWN |
| HRH4 | -1.51492 | -3.43747 | -3.75663 | 0.000499 | 0.009518 | -1.38334 | DOWN |
| RP5-1120P11.4 | -1.0801 | -4.79263 | -3.87094 | 0.000353 | 0.007295 | -1.41081 | DOWN |
| RD3L | -1.57537 | -3.78668 | -3.75405 | 0.000503 | 0.009569 | -1.41311 | DOWN |
| GAS1 | -1.11947 | 0.049508 | -3.85059 | 0.000375 | 0.007626 | -1.41943 | DOWN |
| RP11-566K19.6 | -1.32074 | -4.98712 | -3.90449 | 0.000318 | 0.006759 | -1.42892 | DOWN |
| SERTAD4 | -1.66527 | -4.89827 | -3.88474 | 0.000338 | 0.007039 | -1.43681 | DOWN |
| AC018647.3 | -1.70789 | -3.91877 | -3.75461 | 0.000502 | 0.009564 | -1.4407 | DOWN |
| RP11-255H23.2 | -1.45986 | -3.75781 | -3.73525 | 0.000533 | 0.009986 | -1.45391 | DOWN |
| RP11-178L8.8 | -1.55997 | -3.48319 | -3.73127 | 0.000539 | 0.010081 | -1.4598 | DOWN |
| RP11-396C23.4 | -1.37784 | -4.24019 | -3.76291 | 0.00049 | 0.009385 | -1.46026 | DOWN |
| RP11-941H19.1 | -1.21224 | -3.77239 | -3.72316 | 0.000552 | 0.010251 | -1.4768 | DOWN |
| RP11-1348G14.4 | -1.34128 | -2.71807 | -3.71299 | 0.00057 | 0.010474 | -1.48448 | DOWN |
| GDNF-AS1 | -1.51769 | -3.16234 | -3.72203 | 0.000554 | 0.010262 | -1.48814 | DOWN |
| RP5-881L22.6 | -1.41215 | -1.19165 | -3.75524 | 0.000501 | 0.009552 | -1.49625 | DOWN |
| PRSS42 | -1.46664 | -3.18904 | -3.71803 | 0.000561 | 0.010358 | -1.49637 | DOWN |
| INHA | -1.60622 | -3.34634 | -3.71867 | 0.00056 | 0.010348 | -1.49704 | DOWN |
| LINC00957 | -1.33212 | -1.54145 | -3.73499 | 0.000533 | 0.009991 | -1.50739 | DOWN |
| RP11-266L9.5 | -1.45897 | -3.39322 | -3.70981 | 0.000575 | 0.010558 | -1.51643 | DOWN |
| RP11-417L19.2 | -1.617 | -3.48222 | -3.71246 | 0.00057 | 0.010484 | -1.51704 | DOWN |
| RP11-45A17.3 | -1.45393 | -3.91175 | -3.71665 | 0.000563 | 0.010392 | -1.52247 | DOWN |
| RP3-525N10.2 | -1.49842 | -3.67654 | -3.7068 | 0.00058 | 0.010634 | -1.53254 | DOWN |
| MTND6P21 | -1.48561 | -2.13137 | -3.70507 | 0.000583 | 0.010668 | -1.53443 | DOWN |
| LURAP1 | -1.54504 | -3.72079 | -3.70248 | 0.000588 | 0.010722 | -1.55109 | DOWN |
| AXIN2 | -1.08105 | 0.222875 | -3.8061 | 0.00043 | 0.008472 | -1.57909 | DOWN |
| AC078942.1 | -1.5556 | -3.0464 | -3.68538 | 0.000619 | 0.011161 | -1.59311 | DOWN |
| RP11-1275H24.1 | -1.02582 | -1.92102 | -3.68861 | 0.000613 | 0.011081 | -1.59342 | DOWN |
| RP11-182J1.3 | -1.03385 | -0.68159 | -3.74393 | 0.000519 | 0.009801 | -1.61098 | DOWN |
| CH17-472G23.4 | -1.1145 | 1.131202 | -3.84638 | 0.00038 | 0.007713 | -1.61929 | DOWN |
| TMEM178B | -1.55819 | -3.6042 | -3.67619 | 0.000636 | 0.011406 | -1.62014 | DOWN |
| KCNIP2-AS1 | -1.4235 | -3.49795 | -3.67322 | 0.000642 | 0.011488 | -1.62121 | DOWN |
| RP11-640L9.2 | -1.52652 | -1.33493 | -3.70459 | 0.000584 | 0.010674 | -1.62211 | DOWN |
| MIR148A | -1.26571 | -4.49907 | -3.72822 | 0.000544 | 0.010145 | -1.63295 | DOWN |
| CTC-529L17.2 | -1.5416 | -4.99087 | -3.83704 | 0.000391 | 0.007874 | -1.63373 | DOWN |
| MIR6757 | -1.55682 | -2.51194 | -3.66401 | 0.00066 | 0.011697 | -1.63763 | DOWN |
| DLEU2L | -1.38261 | -3.72802 | -3.66695 | 0.000654 | 0.011643 | -1.64312 | DOWN |
| SPAG8 | -1.02554 | -1.97687 | -3.66837 | 0.000651 | 0.011608 | -1.64548 | DOWN |
| DUX4L27 | -1.55905 | -3.58635 | -3.66601 | 0.000656 | 0.011662 | -1.64931 | DOWN |
| RPL7P44 | -1.01962 | -5.14047 | -3.8725 | 0.000351 | 0.007262 | -1.65242 | DOWN |
| RN7SL151P | -1.73981 | -4.97112 | -3.82499 | 0.000406 | 0.008084 | -1.65363 | DOWN |
| CTC-559E9.5 | -1.45172 | -2.18167 | -3.66107 | 0.000666 | 0.011778 | -1.65469 | DOWN |
| GSG2 | -1.43143 | -3.45927 | -3.66077 | 0.000666 | 0.011785 | -1.65652 | DOWN |
| RP11-111M22.2 | -1.16007 | -1.9568 | -3.66447 | 0.000659 | 0.011689 | -1.66032 | DOWN |
| RP11-182J1.17 | -1.4556 | -3.55636 | -3.66001 | 0.000668 | 0.011798 | -1.66173 | DOWN |
| AC010980.2 | -1.17577 | -4.71669 | -3.74821 | 0.000512 | 0.009715 | -1.66468 | DOWN |
| RP13-582O9.7 | -1.36863 | -3.87214 | -3.66007 | 0.000668 | 0.011798 | -1.67345 | DOWN |
| ZNF781 | -1.54474 | -3.13625 | -3.65581 | 0.000676 | 0.011911 | -1.68007 | DOWN |
| LINC00202-1 | -1.40206 | -1.41292 | -3.68012 | 0.000629 | 0.011297 | -1.68255 | DOWN |
| RP11-455I9.1 | -1.55735 | -3.62744 | -3.65405 | 0.00068 | 0.011956 | -1.68536 | DOWN |
| UPK3B | -1.28628 | -4.11119 | -3.66624 | 0.000655 | 0.011661 | -1.68681 | DOWN |
| GAPDHP23 | -1.47006 | -3.27307 | -3.64841 | 0.000691 | 0.012116 | -1.69415 | DOWN |
| RP11-388C12.1 | -1.50799 | -3.39559 | -3.64111 | 0.000706 | 0.012321 | -1.71559 | DOWN |
| RP11-691N7.6 | -1.49714 | -3.22118 | -3.63553 | 0.000718 | 0.012482 | -1.73333 | DOWN |
| RP11-704M14.1 | -1.3501 | -1.05759 | -3.67869 | 0.000631 | 0.011338 | -1.73808 | DOWN |
| GOLGA6A | -1.41217 | -3.7436 | -3.6338 | 0.000722 | 0.012526 | -1.74114 | DOWN |
| KIAA1024 | -1.37895 | -3.8031 | -3.63082 | 0.000728 | 0.012612 | -1.75208 | DOWN |
| RP11-96D1.10 | -1.04944 | -1.39334 | -3.65654 | 0.000675 | 0.011896 | -1.75516 | DOWN |
| RP11-260M19.2 | -1.64983 | -3.21766 | -3.62699 | 0.000737 | 0.012738 | -1.76479 | DOWN |
| RPS4XP5 | -1.47079 | -3.76676 | -3.62237 | 0.000747 | 0.012853 | -1.77857 | DOWN |
| CD200R1 | -1.07943 | -0.53428 | -3.69334 | 0.000604 | 0.010971 | -1.78063 | DOWN |
| RP11-175P19.2 | -1.09605 | -4.69621 | -3.70351 | 0.000586 | 0.010702 | -1.78522 | DOWN |
| RNU6-564P | -1.63553 | -3.15311 | -3.6169 | 0.000759 | 0.01303 | -1.79378 | DOWN |
| DGAT2L7P | -1.20683 | -4.78727 | -3.73528 | 0.000533 | 0.009986 | -1.79663 | DOWN |
| EDARADD | -1.54024 | -3.24687 | -3.61367 | 0.000767 | 0.013121 | -1.79766 | DOWN |
| CTD-3092A11.1 | -1.04124 | -1.59958 | -3.63157 | 0.000727 | 0.012595 | -1.7977 | DOWN |
| AGRP | -1.08568 | -4.8855 | -3.75981 | 0.000494 | 0.009442 | -1.79808 | DOWN |
| RP13-1016M1.2 | -1.11569 | -4.63553 | -3.68469 | 0.00062 | 0.011178 | -1.80223 | DOWN |
| SHMT1P1 | -1.18334 | -3.579 | -3.60701 | 0.000782 | 0.013306 | -1.80287 | DOWN |
| LINC01126 | -1.35392 | -1.70363 | -3.62433 | 0.000743 | 0.012817 | -1.80519 | DOWN |
| SP5 | -1.13771 | -0.32097 | -3.69652 | 0.000598 | 0.010889 | -1.80706 | DOWN |
| RPS19P3 | -1.20208 | -3.75472 | -3.6073 | 0.000781 | 0.013306 | -1.8079 | DOWN |
| ZNF85 | -1.14919 | -4.97992 | -3.77731 | 0.000469 | 0.009065 | -1.81047 | DOWN |
| RP11-474L23.3 | -1.23133 | -4.58947 | -3.67505 | 0.000638 | 0.011441 | -1.81096 | DOWN |
| LINC00925 | -1.4665 | -3.78289 | -3.61229 | 0.00077 | 0.013159 | -1.81112 | DOWN |
| SPATA45 | -1.35639 | -5.04522 | -3.79581 | 0.000443 | 0.00868 | -1.81881 | DOWN |
| DIRAS2 | -1.79638 | -3.22421 | -3.61016 | 0.000775 | 0.01322 | -1.81947 | DOWN |
| HEXDC-IT1 | -1.61865 | -3.08587 | -3.60699 | 0.000782 | 0.013306 | -1.82298 | DOWN |
| CTD-2623N2.3 | -1.51128 | -3.45045 | -3.6035 | 0.00079 | 0.013433 | -1.8239 | DOWN |
| RP11-685B14.3 | -1.47906 | -3.55684 | -3.59964 | 0.000799 | 0.013556 | -1.83474 | DOWN |
| DUSP15 | -1.57902 | -3.0879 | -3.60262 | 0.000792 | 0.01346 | -1.83568 | DOWN |
| AP000476.1 | -1.47644 | -3.67865 | -3.59781 | 0.000804 | 0.013611 | -1.84386 | DOWN |
| RPL13AP2 | -1.43364 | -3.25581 | -3.59564 | 0.000809 | 0.013677 | -1.84452 | DOWN |
| CRTC3-AS1 | -1.37814 | -3.74503 | -3.59485 | 0.000811 | 0.013699 | -1.85054 | DOWN |
| CD27 | -1.26336 | -1.97684 | -3.59632 | 0.000807 | 0.013659 | -1.85337 | DOWN |
| RP1-283E3.4 | -1.41673 | -3.48182 | -3.58744 | 0.000829 | 0.013911 | -1.86553 | DOWN |
| ZNF812 | -1.31473 | -0.89858 | -3.64197 | 0.000705 | 0.012298 | -1.86862 | DOWN |
| CTC-523E23.3 | -1.36472 | -2.54901 | -3.61152 | 0.000772 | 0.013178 | -1.87034 | DOWN |
| C11orf94 | -1.37107 | -3.33049 | -3.58515 | 0.000834 | 0.013965 | -1.87046 | DOWN |
| CD40LG | -1.50676 | -3.30654 | -3.58621 | 0.000832 | 0.013943 | -1.87235 | DOWN |
| PGAM1P7 | -1.33959 | -4.84571 | -3.72197 | 0.000554 | 0.010262 | -1.87737 | DOWN |
| SHISA3 | -1.5632 | -3.33129 | -3.58445 | 0.000836 | 0.013982 | -1.88 | DOWN |
| ATE1-AS1 | -1.46475 | -3.90817 | -3.59078 | 0.000821 | 0.013833 | -1.89183 | DOWN |
| RP11-131L12.4 | -1.36196 | -4.25222 | -3.6097 | 0.000776 | 0.013234 | -1.89283 | DOWN |
| RP11-791G16.2 | -1.39983 | -4.09197 | -3.59791 | 0.000803 | 0.013611 | -1.89522 | DOWN |
| RP11-388C12.8 | -1.73175 | -3.43904 | -3.57817 | 0.000852 | 0.014186 | -1.9063 | DOWN |
| GZMA | -1.21718 | -1.20532 | -3.6118 | 0.000771 | 0.013175 | -1.90877 | DOWN |
| RP11-424N24.2 | -1.30454 | -3.92628 | -3.57811 | 0.000852 | 0.014186 | -1.9103 | DOWN |
| NYAP1 | -1.36257 | -1.64967 | -3.58857 | 0.000826 | 0.013892 | -1.91341 | DOWN |
| RP11-2E11.6 | -1.57775 | -3.22485 | -3.57261 | 0.000866 | 0.01437 | -1.91598 | DOWN |
| BSN-AS2 | -1.25529 | -4.98953 | -3.74418 | 0.000518 | 0.0098 | -1.91738 | DOWN |
| AC010524.2 | -1.66569 | -3.04856 | -3.57294 | 0.000865 | 0.01436 | -1.92409 | DOWN |
| PKDREJ | -1.30635 | -3.74923 | -3.56682 | 0.000881 | 0.014556 | -1.927 | DOWN |
| AC087350.1 | -1.49376 | -4.93246 | -3.72206 | 0.000554 | 0.010262 | -1.9371 | DOWN |
| TLX1 | -1.03892 | -0.8128 | -3.62339 | 0.000745 | 0.012845 | -1.93801 | DOWN |
| RPL39P40 | -1.50843 | -3.7105 | -3.56256 | 0.000892 | 0.014707 | -1.94953 | DOWN |
| ZNF681 | -1.50829 | -2.89933 | -3.55146 | 0.000922 | 0.015067 | -1.96004 | DOWN |
| RP11-222K16.2 | -1.34649 | -4.04549 | -3.56822 | 0.000877 | 0.014527 | -1.9609 | DOWN |
| RP11-396O20.1 | -1.42243 | -3.31441 | -3.55106 | 0.000923 | 0.015078 | -1.96931 | DOWN |
| C2orf81 | -1.47381 | -2.49695 | -3.54132 | 0.00095 | 0.01542 | -1.98389 | DOWN |
| CTB-33G10.1 | -1.1559 | -3.23086 | -3.54087 | 0.000951 | 0.015433 | -1.98554 | DOWN |
| ZNF93 | -1.18274 | -1.70304 | -3.55942 | 0.0009 | 0.014819 | -1.98896 | DOWN |
| MDP1 | -1.36193 | -2.76654 | -3.53446 | 0.000969 | 0.015621 | -1.99415 | DOWN |
| GATSL3 | -1.32486 | -0.6721 | -3.60955 | 0.000776 | 0.013236 | -1.99551 | DOWN |
| RP11-236L14.2 | -1.46399 | -3.58248 | -3.53896 | 0.000956 | 0.01549 | -2.00563 | DOWN |
| RP11-500B12.1 | -1.49715 | -3.38672 | -3.53851 | 0.000958 | 0.0155 | -2.00687 | DOWN |
| C16orf47 | -1.02722 | -5.25218 | -3.79217 | 0.000448 | 0.008753 | -2.00869 | DOWN |
| RP11-421N8.1 | -1.18947 | -4.13232 | -3.55047 | 0.000925 | 0.015097 | -2.0138 | DOWN |
| RP11-367J11.3 | -1.19434 | -2.59253 | -3.51752 | 0.001019 | 0.016231 | -2.03248 | DOWN |
| RP11-659E9.2 | -1.64511 | -2.82201 | -3.52454 | 0.000998 | 0.015963 | -2.0482 | DOWN |
| RP11-463O9.9 | -1.40308 | -3.90579 | -3.52564 | 0.000995 | 0.01593 | -2.06096 | DOWN |
| ERICH3 | -1.20728 | -1.30009 | -3.55318 | 0.000917 | 0.015025 | -2.06124 | DOWN |
| POU5F1B | -1.34744 | -3.5469 | -3.51663 | 0.001021 | 0.016264 | -2.06335 | DOWN |
| ZNF623 | -1.47424 | -3.81066 | -3.52021 | 0.001011 | 0.016116 | -2.07124 | DOWN |
| CTB-79E8.2 | -1.46279 | -3.86838 | -3.52489 | 0.000997 | 0.015961 | -2.07263 | DOWN |
| LIPJ | -1.37139 | -3.85612 | -3.51763 | 0.001018 | 0.01623 | -2.07974 | DOWN |
| MIR635 | -1.53363 | -2.7755 | -3.50564 | 0.001055 | 0.016666 | -2.08422 | DOWN |
| C10orf62 | -1.56484 | -3.23064 | -3.51081 | 0.001039 | 0.016464 | -2.08913 | DOWN |
| RP3-331H24.7 | -1.22609 | -1.02026 | -3.55743 | 0.000906 | 0.01489 | -2.08999 | DOWN |
| ZNF300P1 | -1.50498 | -2.21504 | -3.50752 | 0.001049 | 0.016597 | -2.09031 | DOWN |
| RP11-150C16.1 | -1.31584 | -3.74944 | -3.50719 | 0.00105 | 0.016608 | -2.09479 | DOWN |
| RP3-394A18.1 | -1.07657 | 2.7165 | -3.77964 | 0.000466 | 0.009016 | -2.0949 | DOWN |
| CTB-13F3.1 | -1.37856 | -3.50808 | -3.50259 | 0.001064 | 0.016779 | -2.10272 | DOWN |
| RP11-1082L8.3 | -1.20096 | -4.13924 | -3.51618 | 0.001023 | 0.016272 | -2.10966 | DOWN |
| NANOGP4 | -1.53776 | -3.35398 | -3.50172 | 0.001067 | 0.016813 | -2.11116 | DOWN |
| PRR36 | -1.31699 | -2.90216 | -3.49092 | 0.001101 | 0.017223 | -2.11656 | DOWN |
| AP001626.1 | -1.6781 | -3.05405 | -3.50435 | 0.001059 | 0.016711 | -2.11714 | DOWN |
| CTD-2545H1.1 | -1.23445 | -4.86111 | -3.64106 | 0.000707 | 0.012321 | -2.12668 | DOWN |
| RP1-30M3.6 | -1.24349 | -2.53707 | -3.48524 | 0.00112 | 0.0174 | -2.12728 | DOWN |
| RP11-778D9.4 | -1.21676 | -3.16708 | -3.48934 | 0.001107 | 0.017271 | -2.12863 | DOWN |
| AC083843.4 | -1.33392 | -0.06805 | -3.59346 | 0.000814 | 0.013743 | -2.13924 | DOWN |
| VWA5B2 | -1.79529 | -2.93161 | -3.49824 | 0.001078 | 0.016949 | -2.14086 | DOWN |
| RP11-288I21.1 | -1.54352 | -3.0436 | -3.48936 | 0.001106 | 0.017271 | -2.14613 | DOWN |
| RP11-21A7A.3 | -1.40438 | -3.74351 | -3.48641 | 0.001116 | 0.017378 | -2.15476 | DOWN |
| AC000068.9 | -1.4674 | -3.05793 | -3.48533 | 0.00112 | 0.0174 | -2.15498 | DOWN |
| RP1-197B17.4 | -1.31946 | -3.22612 | -3.47937 | 0.001139 | 0.01765 | -2.16819 | DOWN |
| RP11-634H22.1 | -1.4554 | -3.30984 | -3.47884 | 0.001141 | 0.017673 | -2.17267 | DOWN |
| RP11-23J18.1 | -1.38938 | -4.54448 | -3.54129 | 0.00095 | 0.01542 | -2.17466 | DOWN |
| WARS2-IT1 | -1.29399 | -4.42947 | -3.52665 | 0.000992 | 0.0159 | -2.17656 | DOWN |
| RAD51AP2 | -1.61709 | -3.23109 | -3.47989 | 0.001138 | 0.017633 | -2.17712 | DOWN |
| RP11-295P9.2 | -1.12825 | -5.19172 | -3.70915 | 0.000576 | 0.010575 | -2.18177 | DOWN |
| ZNF391 | -1.14926 | -2.20205 | -3.47039 | 0.00117 | 0.018025 | -2.18543 | DOWN |
| RCOR2 | -1.39117 | -3.5481 | -3.46886 | 0.001175 | 0.018085 | -2.1982 | DOWN |
| RP11-435O5.5 | -1.3276 | -3.56469 | -3.46793 | 0.001178 | 0.018122 | -2.19888 | DOWN |
| GPR82 | -1.22269 | -0.61086 | -3.53987 | 0.000954 | 0.015472 | -2.20336 | DOWN |
| EMILIN3 | -1.09496 | -0.84807 | -3.52657 | 0.000992 | 0.0159 | -2.20564 | DOWN |
| ST6GALNAC4P1 | -1.10148 | -4.62473 | -3.5424 | 0.000947 | 0.015383 | -2.20642 | DOWN |
| RP11-6B6.3 | -1.08843 | -4.72072 | -3.57576 | 0.000858 | 0.014257 | -2.21456 | DOWN |
| RP11-793H13.3 | -1.3679 | -2.23008 | -3.46365 | 0.001193 | 0.018261 | -2.21709 | DOWN |
| CYP4F23P | -1.32278 | -4.5357 | -3.52247 | 0.001004 | 0.016031 | -2.21909 | DOWN |
| RP11-412D9.4 | -1.13996 | -1.48942 | -3.48624 | 0.001117 | 0.017378 | -2.22288 | DOWN |
| HMGB1P8 | -1.33203 | -3.46672 | -3.45848 | 0.001211 | 0.018457 | -2.22394 | DOWN |
| RP1-167A14.2 | -1.27505 | -3.99074 | -3.46763 | 0.001179 | 0.018122 | -2.22766 | DOWN |
| RP11-783K16.13 | -1.29804 | -2.54516 | -3.44696 | 0.001252 | 0.018929 | -2.23692 | DOWN |
| RN7SKP80 | -1.10475 | -4.22545 | -3.47375 | 0.001158 | 0.017885 | -2.24258 | DOWN |
| RPL24P8 | -1.0531 | -4.67138 | -3.5389 | 0.000957 | 0.01549 | -2.24574 | DOWN |
| F10-AS1 | -1.30314 | -3.98687 | -3.46169 | 0.0012 | 0.018337 | -2.24578 | DOWN |
| RP11-642P15.1 | -1.09852 | -1.06724 | -3.50024 | 0.001072 | 0.016868 | -2.24598 | DOWN |
| AC083843.2 | -1.28443 | -4.17374 | -3.47205 | 0.001164 | 0.01797 | -2.25079 | DOWN |
| MYCBP2-AS1 | -1.20039 | -2.05967 | -3.44721 | 0.001251 | 0.01892 | -2.26276 | DOWN |
| RNA5SP111 | -1.41693 | -3.971 | -3.45747 | 0.001215 | 0.018506 | -2.26576 | DOWN |
| TRGC1 | -1.21149 | -3.94134 | -3.45101 | 0.001238 | 0.018745 | -2.26668 | DOWN |
| RP4-601P9.2 | -1.318 | -3.84944 | -3.44297 | 0.001267 | 0.01913 | -2.28169 | DOWN |
| CTD-3035D6.2 | -1.58113 | -3.26957 | -3.44005 | 0.001278 | 0.019278 | -2.28631 | DOWN |
| RP11-420A6.2 | -1.29521 | -4.85404 | -3.57723 | 0.000854 | 0.014207 | -2.29616 | DOWN |
| RP11-89N17.1 | -1.33808 | -4.05681 | -3.45027 | 0.00124 | 0.018781 | -2.30024 | DOWN |
| GCSHP5 | -1.02838 | -4.47063 | -3.48144 | 0.001132 | 0.017567 | -2.30428 | DOWN |
| RP11-365H22.2 | -1.33365 | -4.65486 | -3.51157 | 0.001037 | 0.016449 | -2.30506 | DOWN |
| CTD-3193O13.1 | -1.31652 | -1.63113 | -3.4438 | 0.001264 | 0.019093 | -2.32165 | DOWN |
| SCARNA12 | -1.602 | -3.17597 | -3.42671 | 0.001328 | 0.019845 | -2.32511 | DOWN |
| RP1-257A7.5 | -1.41233 | -3.41249 | -3.42259 | 0.001344 | 0.019983 | -2.32634 | DOWN |
| CTC-429P9.5 | -1.25908 | -1.79834 | -3.43406 | 0.0013 | 0.019536 | -2.32766 | DOWN |
| RP11-700H6.1 | -1.2959 | -1.80952 | -3.43355 | 0.001302 | 0.01955 | -2.32821 | DOWN |
| ABCA11P | -1.33147 | -3.80243 | -3.4244 | 0.001337 | 0.019933 | -2.33061 | DOWN |
| RUFY4 | -1.34735 | -3.41435 | -3.41941 | 0.001357 | 0.020101 | -2.33296 | DOWN |
| ESPNP | -1.03551 | -1.42573 | -3.44598 | 0.001256 | 0.018978 | -2.34417 | DOWN |
| RP11-531A24.5 | -1.09763 | -2.34762 | -3.40933 | 0.001397 | 0.020524 | -2.34994 | DOWN |
| AL133493.2 | -1.30669 | -3.81506 | -3.4169 | 0.001367 | 0.020207 | -2.35151 | DOWN |
| RAB11FIP1P1 | -1.34537 | -3.92068 | -3.42129 | 0.001349 | 0.020042 | -2.35362 | DOWN |
| AC012668.2 | -1.39884 | -3.45956 | -3.40254 | 0.001425 | 0.020822 | -2.38142 | DOWN |
| RP11-1143G9.5 | -1.31323 | -0.79259 | -3.46297 | 0.001195 | 0.018283 | -2.38846 | DOWN |
| RP4-635E18.6 | -1.29762 | -3.7302 | -3.40002 | 0.001435 | 0.020936 | -2.39048 | DOWN |
| MEOX2 | -1.5463 | -3.53312 | -3.40066 | 0.001433 | 0.020914 | -2.39428 | DOWN |
| CTD-2538C1.2 | -1.46802 | -3.13946 | -3.40026 | 0.001434 | 0.020934 | -2.39467 | DOWN |
| RP11-503P10.1 | -1.28744 | -3.80901 | -3.39974 | 0.001436 | 0.02094 | -2.39538 | DOWN |
| RP11-1055B8.8 | -1.29567 | -3.86274 | -3.40132 | 0.00143 | 0.020885 | -2.39688 | DOWN |
| LINC00652 | -1.21526 | -3.84001 | -3.39753 | 0.001446 | 0.021045 | -2.39966 | DOWN |
| RPL3P9 | -1.03363 | -4.5591 | -3.45563 | 0.001221 | 0.01858 | -2.42046 | DOWN |
| RP11-121M22.1 | -1.52476 | -3.21563 | -3.39022 | 0.001476 | 0.021384 | -2.4229 | DOWN |
| YWHAEP5 | -1.02623 | -4.28864 | -3.41374 | 0.001379 | 0.020332 | -2.42377 | DOWN |
| AGAP5 | -1.4475 | -2.89277 | -3.38208 | 0.001512 | 0.021747 | -2.42384 | DOWN |
| STK32A | -1.42018 | -3.15792 | -3.38746 | 0.001488 | 0.021507 | -2.42659 | DOWN |
| RP11-452H21.4 | -1.30322 | -2.9107 | -3.37714 | 0.001533 | 0.021954 | -2.4293 | DOWN |
| AOX3P | -1.48783 | -3.31651 | -3.38478 | 0.0015 | 0.021616 | -2.43378 | DOWN |
| CTC-435M10.6 | -1.22255 | -4.88761 | -3.53628 | 0.000964 | 0.015567 | -2.43549 | DOWN |
| RP11-278H7.3 | -1.51027 | -3.27731 | -3.38307 | 0.001507 | 0.021702 | -2.44047 | DOWN |
| C1QTNF9 | -1.36939 | -3.63478 | -3.38093 | 0.001517 | 0.021788 | -2.44208 | DOWN |
| RP5-836J3.1 | -1.40768 | -3.76876 | -3.3815 | 0.001514 | 0.021773 | -2.44923 | DOWN |
| TRAIP | -1.16313 | -1.67316 | -3.39383 | 0.001461 | 0.02121 | -2.45407 | DOWN |
| RP13-580F15.2 | -1.44525 | -1.81846 | -3.38717 | 0.00149 | 0.021515 | -2.45598 | DOWN |
| ZNF204P | -1.1208 | -1.52893 | -3.40003 | 0.001435 | 0.020936 | -2.45624 | DOWN |
| CETN4P | -1.30354 | -4.88989 | -3.52888 | 0.000985 | 0.015836 | -2.45832 | DOWN |
| RP11-422P24.9 | -1.00045 | -4.83297 | -3.51424 | 0.001029 | 0.016343 | -2.45963 | DOWN |
| RP11-2E11.9 | -1.1834 | -3.98817 | -3.38133 | 0.001515 | 0.021779 | -2.45976 | DOWN |
| LINC00861 | -1.25914 | -1.62412 | -3.39336 | 0.001463 | 0.021233 | -2.46169 | DOWN |
| RP11-265E18.1 | -1.08355 | -4.88609 | -3.52572 | 0.000994 | 0.01593 | -2.47013 | DOWN |
| bP-21264C1.2 | -1.42399 | -2.99862 | -3.36539 | 0.001586 | 0.022476 | -2.47769 | DOWN |
| DEPDC1B | -1.46837 | -3.16195 | -3.36803 | 0.001574 | 0.022376 | -2.48278 | DOWN |
| RP11-396C23.2 | -1.24655 | -4.04461 | -3.37739 | 0.001532 | 0.021947 | -2.48477 | DOWN |
| C9orf163 | -1.39574 | -2.51468 | -3.35421 | 0.001638 | 0.023071 | -2.49865 | DOWN |
| RP11-203H19.2 | -1.5123 | -3.00521 | -3.36126 | 0.001605 | 0.022695 | -2.4993 | DOWN |
| UBASH3A | -1.29515 | -2.54588 | -3.35028 | 0.001657 | 0.023266 | -2.49942 | DOWN |
| RP11-417L19.5 | -1.33557 | -1.7041 | -3.37452 | 0.001545 | 0.022074 | -2.50344 | DOWN |
| RP11-803B1.8 | -1.27378 | -3.96998 | -3.36381 | 0.001593 | 0.022556 | -2.5087 | DOWN |
| AC009299.2 | -1.21841 | -4.95668 | -3.52784 | 0.000988 | 0.015867 | -2.5098 | DOWN |
| FAXC | -1.49484 | -5.02625 | -3.54447 | 0.000941 | 0.015315 | -2.5099 | DOWN |
| RN7SKP203 | -1.03448 | -5.13559 | -3.58806 | 0.000827 | 0.013893 | -2.51273 | DOWN |
| RP3-323P24.3 | -1.39352 | -3.2227 | -3.3551 | 0.001634 | 0.023023 | -2.51324 | DOWN |
| TMCC1-AS1 | -1.12528 | -1.77749 | -3.366 | 0.001583 | 0.022463 | -2.51712 | DOWN |
| C15orf65 | -1.26204 | -2.16655 | -3.35167 | 0.00165 | 0.0232 | -2.51839 | DOWN |
| HOXB-AS3 | -1.22131 | -3.70394 | -3.34832 | 0.001666 | 0.023357 | -2.52857 | DOWN |
| LINC00487 | -1.24902 | -4.1473 | -3.37016 | 0.001564 | 0.022277 | -2.53001 | DOWN |
| RP11-348J12.5 | -1.41462 | -4.79794 | -3.46628 | 0.001184 | 0.018164 | -2.53424 | DOWN |
| RPS4XP8 | -1.13648 | -4.73867 | -3.45887 | 0.00121 | 0.018445 | -2.53664 | DOWN |
| STX17-AS1 | -1.01749 | -1.97459 | -3.34951 | 0.00166 | 0.023305 | -2.53821 | DOWN |
| FBXL16 | -1.68393 | -3.09417 | -3.35067 | 0.001655 | 0.02325 | -2.54103 | DOWN |
| RPL5P4 | -1.6643 | -4.75511 | -3.46544 | 0.001187 | 0.018195 | -2.54452 | DOWN |
| RP11-480I12.5 | -1.10279 | -0.43624 | -3.42542 | 0.001333 | 0.019899 | -2.55445 | DOWN |
| RP11-354K4.2 | -1.21201 | -4.66359 | -3.42332 | 0.001341 | 0.019967 | -2.56061 | DOWN |
| RP11-730K11.1 | -1.31652 | -3.80792 | -3.33703 | 0.001721 | 0.023925 | -2.56894 | DOWN |
| RP11-304L19.13 | -1.43132 | -3.06755 | -3.33199 | 0.001746 | 0.024216 | -2.57163 | DOWN |
| CTD-3162L10.3 | -1.39036 | -3.04491 | -3.32995 | 0.001756 | 0.024329 | -2.57447 | DOWN |
| NCAN | -1.4398 | -3.07218 | -3.32558 | 0.001778 | 0.024595 | -2.59407 | DOWN |
| CTD-2506P8.6 | -1.45129 | -2.82747 | -3.31956 | 0.001809 | 0.024927 | -2.59463 | DOWN |
| CTB-158D10.3 | -1.0551 | -1.46259 | -3.35244 | 0.001646 | 0.023161 | -2.59593 | DOWN |
| RP11-80H18.4 | -1.20883 | -5.22789 | -3.56939 | 0.000874 | 0.014491 | -2.60243 | DOWN |
| AC087884.1 | -1.12898 | -4.44909 | -3.37521 | 0.001542 | 0.022041 | -2.60374 | DOWN |
| NTN3 | -1.11515 | -1.54986 | -3.34231 | 0.001695 | 0.023663 | -2.6115 | DOWN |
| RP11-403B2.7 | -1.24611 | -4.92839 | -3.48248 | 0.001129 | 0.017532 | -2.61735 | DOWN |
| RP11-78O7.2 | -1.51932 | -2.81274 | -3.31265 | 0.001845 | 0.025312 | -2.61859 | DOWN |
| GRIK1 | -1.5339 | -4.80024 | -3.42453 | 0.001337 | 0.019933 | -2.61934 | DOWN |
| RP11-159F24.2 | -1.49069 | -2.83359 | -3.30934 | 0.001863 | 0.02548 | -2.62554 | DOWN |
| AP000254.8 | -1.25239 | -2.68656 | -3.30172 | 0.001904 | 0.025871 | -2.62898 | DOWN |
| AP003068.18 | -1.24206 | -2.33588 | -3.30589 | 0.001881 | 0.025643 | -2.63023 | DOWN |
| RP11-715J22.4 | -1.15407 | -4.49166 | -3.36875 | 0.001571 | 0.022341 | -2.63232 | DOWN |
| VTI1BP1 | -1.00276 | -4.83755 | -3.44979 | 0.001242 | 0.018798 | -2.64495 | DOWN |
| ST8SIA6-AS1 | -1.20063 | -4.76626 | -3.42875 | 0.00132 | 0.019763 | -2.6515 | DOWN |
| SFRP4 | -1.27487 | -3.85026 | -3.30469 | 0.001888 | 0.025712 | -2.65793 | DOWN |
| AC142472.6 | -1.15344 | -2.05939 | -3.30279 | 0.001898 | 0.025823 | -2.65909 | DOWN |
| EFCAB1 | -1.29528 | -1.90113 | -3.30747 | 0.001873 | 0.025551 | -2.66345 | DOWN |
| AL161668.5 | -1.11413 | 0.389418 | -3.43523 | 0.001296 | 0.019495 | -2.66736 | DOWN |
| IMPG2 | -1.44611 | -3.30591 | -3.29577 | 0.001936 | 0.026223 | -2.67502 | DOWN |
| RP11-686D22.4 | -1.34114 | -3.92731 | -3.30254 | 0.001899 | 0.025834 | -2.67792 | DOWN |
| RP11-109D9.4 | -1.25465 | -4.1554 | -3.31084 | 0.001855 | 0.025401 | -2.6812 | DOWN |
| KC6 | -1.55123 | -3.13768 | -3.29597 | 0.001935 | 0.02622 | -2.68226 | DOWN |
| CTD-2134A5.3 | -1.40276 | -3.26749 | -3.28655 | 0.001988 | 0.026741 | -2.69869 | DOWN |
| RP11-84A14.5 | -1.37346 | -3.35983 | -3.28567 | 0.001993 | 0.026789 | -2.69915 | DOWN |
| ZNF727 | -1.2935 | -4.17599 | -3.30688 | 0.001876 | 0.025582 | -2.70862 | DOWN |
| FAM227A | -1.30985 | -3.69405 | -3.28172 | 0.002016 | 0.027036 | -2.71148 | DOWN |
| RP11-33O4.2 | -1.49026 | -3.09527 | -3.28292 | 0.002009 | 0.026962 | -2.713 | DOWN |
| ZNF790-AS1 | -1.29036 | -3.22771 | -3.27999 | 0.002026 | 0.027138 | -2.71336 | DOWN |
| RP5-894A10.2 | -1.47942 | -3.33056 | -3.26953 | 0.002087 | 0.027741 | -2.74663 | DOWN |
| RPL37P23 | -1.16752 | -4.05005 | -3.27413 | 0.00206 | 0.0275 | -2.75784 | DOWN |
| RP11-119F19.4 | -1.28755 | -3.61994 | -3.25964 | 0.002146 | 0.028298 | -2.76717 | DOWN |
| SUMO4 | -1.28095 | -3.56285 | -3.25884 | 0.002151 | 0.028348 | -2.76802 | DOWN |
| OR10J5 | -1.45736 | -4.8713 | -3.41314 | 0.001382 | 0.020355 | -2.77181 | DOWN |
| TRDC | -1.482 | -3.27781 | -3.26029 | 0.002142 | 0.028266 | -2.77275 | DOWN |
| TAB3P1 | -1.18479 | -5.0374 | -3.45171 | 0.001235 | 0.018722 | -2.7767 | DOWN |
| CTA-212A2.1 | -1.22069 | -4.07683 | -3.26945 | 0.002087 | 0.027741 | -2.78139 | DOWN |
| RP11-474I16.8 | -1.21368 | -4.13225 | -3.27566 | 0.002051 | 0.027424 | -2.78143 | DOWN |
| RPL10P3 | -1.05529 | -4.74772 | -3.3656 | 0.001585 | 0.022475 | -2.78249 | DOWN |
| TMIGD2 | -1.39008 | -2.55905 | -3.2472 | 0.002223 | 0.029077 | -2.78462 | DOWN |
| HSPE1P3 | -1.24225 | -4.61066 | -3.33119 | 0.00175 | 0.024254 | -2.78833 | DOWN |
| AC104076.3 | -1.2856 | -5.07706 | -3.45487 | 0.001224 | 0.018589 | -2.7888 | DOWN |
| C20orf166-AS1 | -1.4417 | -3.89475 | -3.26496 | 0.002114 | 0.027976 | -2.78954 | DOWN |
| RPS3AP54 | -1.27328 | -3.49762 | -3.24869 | 0.002214 | 0.028987 | -2.79463 | DOWN |
| RP11-624L12.1 | -1.07 | -4.28769 | -3.27828 | 0.002035 | 0.027252 | -2.79684 | DOWN |
| GPR150 | -1.44096 | -3.49638 | -3.249 | 0.002212 | 0.028975 | -2.79977 | DOWN |
| CTC-241N9.1 | -1.05112 | -1.72978 | -3.26172 | 0.002133 | 0.028164 | -2.8054 | DOWN |
| LA16c-380H5.6 | -1.38115 | -4.63422 | -3.32711 | 0.001771 | 0.024505 | -2.80854 | DOWN |
| RP11-113K21.6 | -1.11033 | -4.26355 | -3.27456 | 0.002057 | 0.027485 | -2.80937 | DOWN |
| RP1-265C24.8 | -1.01381 | -4.71321 | -3.34714 | 0.001672 | 0.02342 | -2.8135 | DOWN |
| FPGT-TNNI3K | -1.38646 | -2.01656 | -3.24712 | 0.002224 | 0.029077 | -2.81364 | DOWN |
| RP11-795F19.5 | -1.42251 | -3.18976 | -3.24483 | 0.002238 | 0.029196 | -2.81385 | DOWN |
| RP11-883G14.3 | -1.25408 | -3.84306 | -3.24474 | 0.002239 | 0.029196 | -2.81625 | DOWN |
| LINC00940 | -1.0925 | -4.60347 | -3.31577 | 0.001829 | 0.025155 | -2.82163 | DOWN |
| RPL3P1 | -1.28167 | -4.80614 | -3.37699 | 0.001534 | 0.021956 | -2.82343 | DOWN |
| RP1-102E24.10 | -1.35231 | -3.29348 | -3.23908 | 0.002275 | 0.029582 | -2.8236 | DOWN |
| RP11-170N16.3 | -1.29031 | -2.33885 | -3.23216 | 0.00232 | 0.030044 | -2.82963 | DOWN |
| RP5-867C24.1 | -1.43303 | -4.79217 | -3.36971 | 0.001567 | 0.022295 | -2.83001 | DOWN |
| DOCK3 | -1.18026 | -4.15222 | -3.25536 | 0.002172 | 0.028566 | -2.83362 | DOWN |
| ADH5P3 | -1.02151 | -4.86468 | -3.38949 | 0.00148 | 0.021409 | -2.83419 | DOWN |
| RP4-798A10.2 | -1.29303 | -2.65649 | -3.22381 | 0.002375 | 0.03055 | -2.83965 | DOWN |
| COL18A1-AS2 | -1.40607 | -3.74237 | -3.23527 | 0.002299 | 0.029834 | -2.84297 | DOWN |
| AC023115.2 | -1.63658 | -3.21928 | -3.23629 | 0.002293 | 0.029769 | -2.84408 | DOWN |
| PRRT1 | -1.32568 | -3.38831 | -3.23069 | 0.002329 | 0.030123 | -2.84431 | DOWN |
| STRCP1 | -1.39083 | -3.15975 | -3.23175 | 0.002322 | 0.030053 | -2.84791 | DOWN |
| RP3-405J10.3 | -1.20021 | -3.72105 | -3.22829 | 0.002345 | 0.030287 | -2.85187 | DOWN |
| CTB-131B5.5 | -1.15669 | -1.19387 | -3.27174 | 0.002074 | 0.027636 | -2.85309 | DOWN |
| DPEP3 | -1.45488 | -3.36594 | -3.22602 | 0.00236 | 0.030435 | -2.86172 | DOWN |
| RP11-495P10.1 | -1.45333 | -3.58571 | -3.22591 | 0.002361 | 0.030438 | -2.86299 | DOWN |
| RP1-168L15.6 | -1.31234 | -3.97664 | -3.23449 | 0.002305 | 0.029884 | -2.86481 | DOWN |
| C1orf204 | -1.30682 | -3.47872 | -3.22209 | 0.002387 | 0.030645 | -2.8661 | DOWN |
| RPL27AP5 | -1.34744 | -4.79495 | -3.35874 | 0.001617 | 0.022838 | -2.8673 | DOWN |
| AC025627.7 | -1.32757 | -3.33878 | -3.22186 | 0.002388 | 0.030659 | -2.86817 | DOWN |
| RP11-132A1.3 | -1.7245 | -4.75309 | -3.34849 | 0.001665 | 0.023352 | -2.86936 | DOWN |
| AC079779.4 | -1.3807 | -4.88177 | -3.37803 | 0.001529 | 0.021917 | -2.87005 | DOWN |
| HNRNPLP2 | -1.03486 | -2.66645 | -3.20632 | 0.002495 | 0.031668 | -2.87329 | DOWN |
| RP11-301L8.2 | -1.30879 | -3.53947 | -3.21913 | 0.002407 | 0.030834 | -2.87529 | DOWN |
| SOWAHD | -1.38075 | -2.71536 | -3.21182 | 0.002457 | 0.031278 | -2.87814 | DOWN |
| RP11-10N23.4 | -1.29066 | -4.31016 | -3.25602 | 0.002168 | 0.028526 | -2.88266 | DOWN |
| CTD-2192J16.20 | -1.40547 | -2.77931 | -3.20983 | 0.002471 | 0.031424 | -2.8836 | DOWN |
| ZNF90 | -1.22049 | -2.87967 | -3.20181 | 0.002527 | 0.031954 | -2.89589 | DOWN |
| RP11-824M15.3 | -1.1092 | -4.75326 | -3.33265 | 0.001743 | 0.024176 | -2.91117 | DOWN |
| TSSK3 | -1.21413 | -1.52068 | -3.23178 | 0.002322 | 0.030053 | -2.91319 | DOWN |
| ZNF114 | -1.31703 | -4.03378 | -3.21749 | 0.002418 | 0.030903 | -2.92066 | DOWN |
| RP11-545N8.3 | -1.39447 | -3.33821 | -3.20137 | 0.00253 | 0.031986 | -2.92445 | DOWN |
| C1QTNF1-AS1 | -1.24646 | -3.56854 | -3.1985 | 0.002551 | 0.032183 | -2.92769 | DOWN |
| AP006216.5 | -1.10806 | -1.75878 | -3.21292 | 0.002449 | 0.031221 | -2.93221 | DOWN |
| RP11-186N15.3 | -1.40321 | -2.94157 | -3.19361 | 0.002586 | 0.032467 | -2.93306 | DOWN |
| CKMT2 | -1.43197 | -3.18257 | -3.19968 | 0.002542 | 0.032105 | -2.93363 | DOWN |
| CTD-2545M3.8 | -1.30534 | -3.90823 | -3.20483 | 0.002506 | 0.031753 | -2.93445 | DOWN |
| RP11-54O7.1 | -1.3379 | -3.43936 | -3.19697 | 0.002562 | 0.03228 | -2.93456 | DOWN |
| DNAH12 | -1.25369 | -3.02222 | -3.18159 | 0.002675 | 0.033317 | -2.95785 | DOWN |
| RP11-332H14.1 | -1.31174 | -3.36661 | -3.18769 | 0.002629 | 0.03289 | -2.95833 | DOWN |
| SYCE2 | -1.31786 | -3.20886 | -3.18732 | 0.002632 | 0.032911 | -2.96271 | DOWN |
| RP11-74J13.9 | -1.18126 | -4.09118 | -3.20184 | 0.002527 | 0.031954 | -2.9641 | DOWN |
| RP11-10A14.9 | -1.34134 | -3.4035 | -3.1846 | 0.002652 | 0.033111 | -2.96726 | DOWN |
| DOCK9-AS2 | -1.26433 | -2.79511 | -3.17443 | 0.002729 | 0.033833 | -2.96923 | DOWN |
| RP11-82O19.2 | -1.22193 | -4.99626 | -3.37167 | 0.001558 | 0.022208 | -2.97635 | DOWN |
| RP11-554A11.4 | -1.58576 | -3.31921 | -3.18397 | 0.002657 | 0.033145 | -2.97811 | DOWN |
| BEND5 | -1.12667 | -3.82062 | -3.18183 | 0.002673 | 0.033307 | -2.97837 | DOWN |
| AC097468.4 | -1.27033 | -4.43425 | -3.23228 | 0.002319 | 0.030041 | -2.9786 | DOWN |
| RP4-568B10.1 | -1.18219 | -4.4545 | -3.23028 | 0.002332 | 0.030144 | -2.98371 | DOWN |
| CTC-523E23.1 | -1.18049 | -4.14178 | -3.19837 | 0.002551 | 0.032188 | -2.99055 | DOWN |
| FANCB | -1.31593 | -4.05295 | -3.19794 | 0.002555 | 0.032216 | -2.9913 | DOWN |
| MMP23B | -1.19463 | -3.86494 | -3.17533 | 0.002722 | 0.033789 | -3.00296 | DOWN |
| RNF144A-AS1 | -1.3247 | -3.82818 | -3.17441 | 0.002729 | 0.033833 | -3.00566 | DOWN |
| RP11-49I11.2 | -1.38323 | -3.36775 | -3.16679 | 0.002788 | 0.034387 | -3.01565 | DOWN |
| ZNF860 | -1.21671 | -4.00012 | -3.17484 | 0.002726 | 0.033814 | -3.02016 | DOWN |
| RP11-894J14.2 | -1.28639 | -3.68667 | -3.16503 | 0.002801 | 0.03452 | -3.02058 | DOWN |
| CTC-527H23.3 | -1.15377 | -4.32904 | -3.19959 | 0.002543 | 0.032105 | -3.02607 | DOWN |
| HMSD | -1.18781 | -3.65371 | -3.16043 | 0.002838 | 0.034842 | -3.02881 | DOWN |
| RP11-645C24.5 | -1.44583 | -3.15735 | -3.16251 | 0.002821 | 0.034713 | -3.03378 | DOWN |
| PRSS57 | -1.25772 | -4.70191 | -3.26757 | 0.002098 | 0.027832 | -3.04574 | DOWN |
| MAGOH2P | -1.17141 | -1.61101 | -3.17473 | 0.002726 | 0.033817 | -3.05243 | DOWN |
| CTB-96E2.7 | -1.45332 | -3.30947 | -3.15034 | 0.002919 | 0.035568 | -3.06217 | DOWN |
| RFPL1S | -1.41473 | -2.87154 | -3.14325 | 0.002977 | 0.036082 | -3.06552 | DOWN |
| SPATA9 | -1.37708 | -3.50626 | -3.14413 | 0.00297 | 0.036016 | -3.07562 | DOWN |
| UBTFL10 | -1.44602 | -3.636 | -3.1462 | 0.002953 | 0.035875 | -3.07577 | DOWN |
| KCNS2 | -1.25154 | -3.9605 | -3.15063 | 0.002916 | 0.035548 | -3.07993 | DOWN |
| RP11-15L13.4 | -1.37295 | -3.25582 | -3.13886 | 0.003014 | 0.036397 | -3.09032 | DOWN |
| RP11-84C10.1 | -1.06183 | -5.13255 | -3.37682 | 0.001535 | 0.021961 | -3.09645 | DOWN |
| RP5-902P8.12 | -1.20789 | -3.7289 | -3.13421 | 0.003053 | 0.036787 | -3.10058 | DOWN |
| FOXO3B | -1.35675 | -2.93985 | -3.12816 | 0.003105 | 0.037309 | -3.10123 | DOWN |
| TMEM169 | -1.03214 | -0.43161 | -3.22323 | 0.002379 | 0.030567 | -3.10413 | DOWN |
| IGLV2-18 | -1.34329 | -3.85573 | -3.13855 | 0.003016 | 0.036414 | -3.10484 | DOWN |
| RP11-484K9.4 | -1.19855 | -4.93931 | -3.30794 | 0.00187 | 0.025528 | -3.10563 | DOWN |
| RP11-649G15.2 | -1.04963 | -4.4723 | -3.18545 | 0.002646 | 0.03307 | -3.11448 | DOWN |
| VWA3A | -1.20379 | -3.62819 | -3.12725 | 0.003113 | 0.037383 | -3.11544 | DOWN |
| LAMTOR5P1 | -1.10317 | -4.89403 | -3.29201 | 0.001957 | 0.026441 | -3.1162 | DOWN |
| LINC01094 | -1.30061 | -3.39526 | -3.12696 | 0.003115 | 0.037405 | -3.1167 | DOWN |
| GAPDHP74 | -1.18998 | -4.00019 | -3.13173 | 0.003074 | 0.036997 | -3.1314 | DOWN |
| CTD-2256P15.5 | -1.20213 | -4.31057 | -3.15761 | 0.00286 | 0.035073 | -3.14348 | DOWN |
| CTD-2240J17.1 | -1.18344 | -4.03652 | -3.12645 | 0.00312 | 0.037443 | -3.1508 | DOWN |
| RSPH14 | -1.11759 | -4.61235 | -3.19512 | 0.002575 | 0.032378 | -3.15554 | DOWN |
| AC004012.1 | -1.35163 | -3.16302 | -3.11334 | 0.003235 | 0.038467 | -3.15731 | DOWN |
| MIR3685 | -1.32585 | -3.82112 | -3.11597 | 0.003212 | 0.038258 | -3.15908 | DOWN |
| CHRM3-AS2 | -1.36023 | -3.09802 | -3.10295 | 0.00333 | 0.039334 | -3.17773 | DOWN |
| PRR15L | -1.23943 | -1.32818 | -3.1416 | 0.002991 | 0.03621 | -3.17811 | DOWN |
| FBXO43 | -1.34854 | -3.84253 | -3.10909 | 0.003274 | 0.038836 | -3.17959 | DOWN |
| FOXP4-AS1 | -1.09903 | -4.39698 | -3.149 | 0.00293 | 0.035679 | -3.18225 | DOWN |
| RP13-735L24.1 | -1.19238 | -3.96169 | -3.109 | 0.003274 | 0.038838 | -3.18409 | DOWN |
| RP11-357H14.16 | -1.17897 | -4.56335 | -3.17254 | 0.002743 | 0.033982 | -3.18683 | DOWN |
| AC005562.1 | -1.33245 | -3.19671 | -3.10095 | 0.003348 | 0.039504 | -3.18925 | DOWN |
| RPS2P55 | -1.19994 | -3.58802 | -3.09799 | 0.003376 | 0.039776 | -3.19019 | DOWN |
| RP1-8B1.4 | -1.25677 | -3.69245 | -3.09637 | 0.003391 | 0.039879 | -3.1988 | DOWN |
| RP1-309F20.3 | -1.30535 | -3.34015 | -3.09405 | 0.003413 | 0.040071 | -3.20322 | DOWN |
| TSSC1-IT1 | -1.2541 | -3.41794 | -3.09296 | 0.003423 | 0.040176 | -3.20395 | DOWN |
| SLITRK6 | -1.44171 | -0.98864 | -3.14928 | 0.002927 | 0.035658 | -3.20588 | DOWN |
| RP11-187O7.1 | -1.08158 | -4.6841 | -3.1979 | 0.002555 | 0.032216 | -3.20707 | DOWN |
| RP11-403P17.3 | -1.2465 | -2.91363 | -3.07963 | 0.003551 | 0.041276 | -3.21886 | DOWN |
| RPL12L3 | -1.04893 | -4.93525 | -3.2644 | 0.002117 | 0.028014 | -3.21975 | DOWN |
| RP11-745A24.2 | -1.23148 | -4.66668 | -3.18916 | 0.002618 | 0.032797 | -3.22374 | DOWN |
| FDX1L | -1.10564 | -3.93386 | -3.0906 | 0.003445 | 0.040398 | -3.22504 | DOWN |
| CTD-2240J17.4 | -1.2739 | -4.4921 | -3.14772 | 0.00294 | 0.035791 | -3.22513 | DOWN |
| RP11-399J13.2 | -1.6891 | -2.60513 | -3.08577 | 0.003492 | 0.040789 | -3.22674 | DOWN |
| C9orf147 | -1.083 | -3.97824 | -3.09017 | 0.00345 | 0.040413 | -3.23035 | DOWN |
| SLC46A2 | -1.34393 | -3.50332 | -3.08358 | 0.003513 | 0.040958 | -3.23097 | DOWN |
| HMGB3P22 | -1.27463 | -3.60664 | -3.08273 | 0.003521 | 0.041045 | -3.23245 | DOWN |
| FOXS1 | -1.32536 | -3.86502 | -3.08909 | 0.00346 | 0.040526 | -3.23259 | DOWN |
| ARHGAP11A | -1.2879 | -3.58294 | -3.08005 | 0.003547 | 0.041252 | -3.23973 | DOWN |
| ADD3-AS1 | -1.2745 | -4.06393 | -3.09781 | 0.003378 | 0.039784 | -3.24122 | DOWN |
| AC098617.1 | -1.38117 | -3.26696 | -3.07667 | 0.003581 | 0.041539 | -3.25235 | DOWN |
| BRINP2 | -1.03661 | -1.66038 | -3.09532 | 0.003401 | 0.039967 | -3.25382 | DOWN |
| RP11-20J15.3 | -1.31533 | -3.28746 | -3.07094 | 0.003638 | 0.042016 | -3.2644 | DOWN |
| NAP1L1P1 | -1.15131 | -3.87384 | -3.07149 | 0.003632 | 0.041977 | -3.2705 | DOWN |
| AC114752.2 | -1.26959 | -4.65067 | -3.16817 | 0.002777 | 0.034304 | -3.27086 | DOWN |
| RP11-253M7.1 | -1.12224 | -4.2751 | -3.09796 | 0.003376 | 0.039776 | -3.27318 | DOWN |
| RP11-71E19.1 | -1.37687 | -3.47617 | -3.06755 | 0.003672 | 0.042351 | -3.27372 | DOWN |
| RPS4XP11 | -1.08916 | -4.76849 | -3.19644 | 0.002565 | 0.032293 | -3.27835 | DOWN |
| AP000347.4 | -1.24546 | -3.5892 | -3.06283 | 0.00372 | 0.042744 | -3.28284 | DOWN |
| RP11-2E17.2 | -1.2288 | -3.86279 | -3.06615 | 0.003686 | 0.042456 | -3.28745 | DOWN |
| CTC-338M12.5 | -1.17323 | -3.6992 | -3.05788 | 0.003771 | 0.043143 | -3.2971 | DOWN |
| AC011899.9 | -1.09294 | -0.40693 | -3.15142 | 0.00291 | 0.035505 | -3.29734 | DOWN |
| CDRT4 | -1.32462 | -3.23079 | -3.05747 | 0.003775 | 0.043183 | -3.30056 | DOWN |
| RP11-97O12.7 | -1.01812 | -4.80016 | -3.19568 | 0.002571 | 0.032341 | -3.30188 | DOWN |
| PROCA1 | -1.06484 | -1.78672 | -3.06926 | 0.003654 | 0.042186 | -3.30495 | DOWN |
| UQCRBP1 | -1.09243 | -4.63812 | -3.15407 | 0.002889 | 0.035317 | -3.3115 | DOWN |
| RP11-710C12.1 | -1.24938 | -3.5365 | -3.05145 | 0.003838 | 0.043749 | -3.31171 | DOWN |
| CH17-270A2.1 | -1.2637 | -4.74832 | -3.16551 | 0.002798 | 0.034488 | -3.3148 | DOWN |
| DPRXP4 | -1.1566 | -3.99738 | -3.05834 | 0.003766 | 0.043106 | -3.3158 | DOWN |
| LINC00605 | -1.10644 | -4.04961 | -3.0611 | 0.003737 | 0.042875 | -3.31588 | DOWN |
| ZNF99 | -1.07632 | -4.67345 | -3.15061 | 0.002917 | 0.035548 | -3.32062 | DOWN |
| RP11-214F16.8 | -1.26938 | -2.64471 | -3.0379 | 0.003983 | 0.044991 | -3.32446 | DOWN |
| FUT8-AS1 | -1.32078 | -3.52365 | -3.04622 | 0.003893 | 0.044234 | -3.32763 | DOWN |
| RP4-761J14.10 | -1.21381 | -4.05135 | -3.05936 | 0.003755 | 0.04301 | -3.32954 | DOWN |
| RP11-562A8.4 | -1.3168 | -3.43468 | -3.04409 | 0.003916 | 0.044432 | -3.33217 | DOWN |
| LRRN3 | -1.19116 | -1.51213 | -3.06798 | 0.003667 | 0.042327 | -3.34446 | DOWN |
| AL162759.1 | -1.28305 | -4.66749 | -3.14882 | 0.002931 | 0.035689 | -3.34684 | DOWN |
| MIR3671 | -1.31704 | -4.57511 | -3.11249 | 0.003243 | 0.038534 | -3.34824 | DOWN |
| FTOP1 | -1.09509 | -4.34945 | -3.07894 | 0.003558 | 0.041337 | -3.35743 | DOWN |
| CLRN1-AS1 | -1.33578 | -3.8023 | -3.03419 | 0.004024 | 0.045319 | -3.36954 | DOWN |
| RPL13AP3 | -1.15159 | -4.36409 | -3.066 | 0.003687 | 0.042456 | -3.37467 | DOWN |
| QRSL1P1 | -1.12122 | -4.36896 | -3.07505 | 0.003597 | 0.041674 | -3.37829 | DOWN |
| XKRX | -1.42019 | -4.61206 | -3.11299 | 0.003238 | 0.038496 | -3.38253 | DOWN |
| CTB-43E15.2 | -1.14944 | -1.54178 | -3.05127 | 0.00384 | 0.043762 | -3.38353 | DOWN |
| RP11-162G10.5 | -1.11934 | -2.96887 | -3.01143 | 0.004282 | 0.047457 | -3.38719 | DOWN |
| CTA-228A9.3 | -1.17093 | -2.17017 | -3.0211 | 0.00417 | 0.046549 | -3.38772 | DOWN |
| LRRC3-AS1 | -1.08338 | -2.19658 | -3.02008 | 0.004182 | 0.046634 | -3.38837 | DOWN |
| GPR142 | -1.09969 | -4.03646 | -3.03305 | 0.004036 | 0.045434 | -3.38869 | DOWN |
| NUTM2F | -1.03319 | -4.75575 | -3.14697 | 0.002946 | 0.035836 | -3.39309 | DOWN |
| RP11-981P6.1 | -1.1428 | -3.87192 | -3.02169 | 0.004164 | 0.0465 | -3.39838 | DOWN |
| CTB-179K24.3 | -1.17221 | -3.04902 | -3.00798 | 0.004322 | 0.047728 | -3.40265 | DOWN |
| RP11-539L10.2 | -1.29716 | -3.25854 | -3.01708 | 0.004216 | 0.046907 | -3.4027 | DOWN |
| RCVRN | -1.2205 | -4.35745 | -3.06093 | 0.003739 | 0.042875 | -3.40337 | DOWN |
| RP4-678D15.1 | -1.36681 | -3.80014 | -3.02133 | 0.004168 | 0.046537 | -3.40677 | DOWN |
| CNTNAP2 | -1.33451 | -3.34658 | -3.01509 | 0.004239 | 0.047128 | -3.407 | DOWN |
| RP11-526I2.5 | -1.18321 | -1.52439 | -3.04057 | 0.003954 | 0.044724 | -3.41311 | DOWN |
| LINC00441 | -1.17638 | -3.63459 | -3.00883 | 0.004312 | 0.047667 | -3.42092 | DOWN |
| IGKV3D-15 | -1.1143 | -4.93669 | -3.18824 | 0.002625 | 0.032853 | -3.42359 | DOWN |
| RP11-468H14.2 | -1.44574 | -3.45885 | -3.00761 | 0.004327 | 0.047754 | -3.42937 | DOWN |
| NALCN-AS1 | -1.10962 | -4.50707 | -3.06695 | 0.003678 | 0.042387 | -3.43394 | DOWN |
| CNN2P3 | -1.42741 | -3.67755 | -3.00751 | 0.004328 | 0.047757 | -3.43397 | DOWN |
| AC004022.7 | -1.03138 | -4.5621 | -3.076 | 0.003587 | 0.04159 | -3.43893 | DOWN |
| ST8SIA5 | -1.293 | -3.71387 | -3.00139 | 0.004401 | 0.04835 | -3.44472 | DOWN |
| RP11-702H23.6 | -1.05343 | -4.81409 | -3.13934 | 0.00301 | 0.036363 | -3.4459 | DOWN |
| NDST3 | -1.11718 | -0.46494 | -3.09051 | 0.003446 | 0.040399 | -3.44669 | DOWN |
| LINC01140 | -1.14042 | -2.42312 | -2.98934 | 0.004547 | 0.049543 | -3.45009 | DOWN |
| RP11-159N11.4 | -1.30797 | -2.54854 | -2.98978 | 0.004542 | 0.049529 | -3.453 | DOWN |
| RP1-69M21.2 | -1.10364 | -4.98113 | -3.19113 | 0.002604 | 0.03263 | -3.45624 | DOWN |
| OVOL2 | -1.10614 | -4.19281 | -3.01819 | 0.004204 | 0.046817 | -3.45753 | DOWN |
| CTD-2054N24.2 | -1.08168 | -4.75781 | -3.12557 | 0.003127 | 0.037511 | -3.46224 | DOWN |
| IL36G | -1.41679 | -3.07398 | -2.98945 | 0.004546 | 0.049543 | -3.47413 | DOWN |
| RP11-344P13.3 | -1.28193 | -3.45648 | -2.98721 | 0.004574 | 0.049718 | -3.47599 | DOWN |
| RP11-649E7.7 | -1.04253 | -4.43101 | -3.03768 | 0.003986 | 0.04501 | -3.4813 | DOWN |
| RP11-548P2.2 | -1.28981 | -4.70459 | -3.10447 | 0.003316 | 0.039207 | -3.48818 | DOWN |
| LINC00086 | -1.00444 | -1.37934 | -3.01941 | 0.00419 | 0.046701 | -3.48849 | DOWN |
| IGLV3-27 | -1.3352 | -3.93236 | -2.99229 | 0.004511 | 0.049253 | -3.48924 | DOWN |
| SNRPCP16 | -1.03289 | -4.80074 | -3.12862 | 0.003101 | 0.037272 | -3.48965 | DOWN |
| RP11-394G3.2 | -1.19108 | -4.17575 | -3.00795 | 0.004323 | 0.047728 | -3.49783 | DOWN |
| RP11-483I13.5 | -1.21351 | -4.65189 | -3.07962 | 0.003552 | 0.041276 | -3.49876 | DOWN |
| LINC01562 | -1.03393 | -4.45773 | -3.03204 | 0.004048 | 0.045526 | -3.51528 | DOWN |
| RP11-687F6.5 | -1.13395 | -4.23394 | -2.99408 | 0.004489 | 0.049081 | -3.53182 | DOWN |
| RP11-290H9.5 | -1.38268 | -4.95194 | -3.15172 | 0.002908 | 0.035491 | -3.53306 | DOWN |
| TCEB2P2 | -1.38357 | -4.62173 | -3.05692 | 0.003781 | 0.043227 | -3.53334 | DOWN |
| SYCP2L | -1.00123 | -4.59046 | -3.04274 | 0.003931 | 0.044572 | -3.54 | DOWN |
| LINC00950 | -1.39032 | -1.40417 | -2.99501 | 0.004478 | 0.049013 | -3.5456 | DOWN |
| RP11-552D4.1 | -1.32571 | -4.61701 | -3.04131 | 0.003946 | 0.044668 | -3.56664 | DOWN |
| RP11-863P13.2 | -1.01384 | -4.71322 | -3.051 | 0.003843 | 0.043786 | -3.58257 | DOWN |
| CASP16 | -1.13812 | 1.564736 | -3.16843 | 0.002775 | 0.034294 | -3.59623 | DOWN |
| RP3-337H4.10 | -1.28349 | -4.7361 | -3.05437 | 0.003807 | 0.04345 | -3.60205 | DOWN |
| ANKRD55 | -1.13051 | 0.878222 | -3.11433 | 0.003226 | 0.038385 | -3.61461 | DOWN |
| RP11-212P7.1 | -1.50976 | -4.62671 | -3.03325 | 0.004034 | 0.045419 | -3.62033 | DOWN |
| KB-1458E12.1 | -1.34518 | -4.73281 | -3.05908 | 0.003758 | 0.043026 | -3.62108 | DOWN |
| RNA5SP296 | -1.05183 | -5.00136 | -3.12499 | 0.003132 | 0.037541 | -3.64 | DOWN |
| RP11-666G4.1 | -1.60526 | -4.7205 | -3.04591 | 0.003897 | 0.044255 | -3.6482 | DOWN |
| MALRD1 | -1.19698 | -4.64187 | -3.00952 | 0.004304 | 0.047616 | -3.67162 | DOWN |
| RP11-431N15.2 | -1.13488 | -4.64243 | -2.99766 | 0.004446 | 0.048747 | -3.70672 | DOWN |
| CCDC74B | -1.13686 | -4.71285 | -2.99446 | 0.004485 | 0.049058 | -3.73835 | DOWN |
| RP11-379F4.9 | -1.00919 | -4.88894 | -3.02118 | 0.00417 | 0.046547 | -3.82245 | DOWN |
| FAM182A | -1.04845 | -4.93811 | -3.0322 | 0.004046 | 0.045514 | -3.83259 | DOWN |
| DNAJC19P9 | -1.27439 | -4.89659 | -3.00834 | 0.004318 | 0.047703 | -3.86051 | DOWN |
| RP11-568A7.3 | -1.18127 | -4.95601 | -3.02626 | 0.004112 | 0.046097 | -3.8647 | DOWN |
| MYCL | -1.2979 | 3.428664 | -3.13914 | 0.003011 | 0.036376 | -3.9863 | DOWN |
