# Supplementary material for: Integrated transcriptomic profiling of programmed cell death patterns unveils macrophage-hepatocyte crosstalk via THBS1-CD47 axis in hepatic ischemia-reperfusion injury
Source: Front Immunol. 2026 May 19;17:1769849. doi: 10.3389/fimmu.2026.1769849 (PMC13225957; doi:10.3389/fimmu.2026.1769849)
Supplement: Supplementary file 4 [file Table3.docx]

| **Table S3. Differentially expressed genes in GSE14951.** | | | | | | | |
| --- | --- | --- | --- | --- | --- | --- | --- |
|  | logFC | AveExpr | t | P.Value | adj.P.Val | B | change |
| IL1RN | 3.432819 | 10.49729 | 21.87869 | 1.28E-10 | 2.11E-06 | 13.85697 | UP |
| KLF10 | 2.963957 | 9.22959 | 20.87995 | 2.14E-10 | 2.11E-06 | 13.48469 | UP |
| TNFAIP3 | 3.219152 | 9.744878 | 18.36897 | 8.80E-10 | 5.67E-06 | 12.41229 | UP |
| C5AR1 | 2.009614 | 7.802082 | 17.9261 | 1.15E-09 | 5.67E-06 | 12.20014 | UP |
| DUSP16 | 1.4533 | 8.948151 | 17.40559 | 1.59E-09 | 6.26E-06 | 11.94091 | UP |
| SAMSN1 | 2.329513 | 7.647238 | 16.77637 | 2.38E-09 | 7.05E-06 | 11.61248 | UP |
| TSC22D2 | 3.250427 | 9.058156 | 16.69577 | 2.50E-09 | 7.05E-06 | 11.56917 | UP |
| MAFF | 2.628024 | 8.85912 | 15.83051 | 4.47E-09 | 1.10E-05 | 11.08539 | UP |
| TIPARP | 3.002048 | 9.297295 | 15.48038 | 5.70E-09 | 1.25E-05 | 10.8794 | UP |
| SLC20A1 | 3.297436 | 9.937879 | 15.19438 | 6.98E-09 | 1.35E-05 | 10.70652 | UP |
| DUSP5 | 2.859218 | 8.337746 | 15.08956 | 7.52E-09 | 1.35E-05 | 10.64209 | UP |
| RND1 | 2.378653 | 8.901926 | 14.59757 | 1.08E-08 | 1.77E-05 | 10.33176 | UP |
| PIGA | 1.652442 | 6.503899 | 14.06272 | 1.61E-08 | 2.28E-05 | 9.978954 | UP |
| CKS2 | 2.302877 | 7.196192 | 14.0542 | 1.62E-08 | 2.28E-05 | 9.9732 | UP |
| ARL5B | 2.359091 | 9.784486 | 13.86146 | 1.88E-08 | 2.34E-05 | 9.84183 | UP |
| JUN | 3.266456 | 9.964491 | 13.84843 | 1.90E-08 | 2.34E-05 | 9.832867 | UP |
| SPRY2 | 1.930906 | 8.139256 | 13.64619 | 2.22E-08 | 2.58E-05 | 9.692424 | UP |
| ATF3 | 3.24998 | 9.335697 | 13.44212 | 2.61E-08 | 2.86E-05 | 9.548121 | UP |
| JUND | 2.237562 | 11.69723 | 13.15911 | 3.28E-08 | 3.40E-05 | 9.343558 | UP |
| GPCPD1 | 1.616067 | 8.198005 | 12.861 | 4.19E-08 | 4.12E-05 | 9.122335 | UP |
| CCL4 | 3.034155 | 9.505858 | 12.63025 | 5.07E-08 | 4.39E-05 | 8.946925 | UP |
| PHLDA1 | 2.60056 | 9.580722 | 12.60749 | 5.17E-08 | 4.39E-05 | 8.929425 | UP |
| SERTAD1 | 1.534086 | 8.390305 | 12.59271 | 5.24E-08 | 4.39E-05 | 8.918036 | UP |
| TGIF1 | 2.319571 | 8.500643 | 12.56767 | 5.35E-08 | 4.39E-05 | 8.898711 | UP |
| FOSB | 3.787702 | 9.155579 | 12.50091 | 5.66E-08 | 4.46E-05 | 8.846966 | UP |
| AKAP12 | 2.449952 | 9.210814 | 12.27204 | 6.89E-08 | 5.09E-05 | 8.667137 | UP |
| JMJD6 | 1.330875 | 7.953454 | 12.25745 | 6.97E-08 | 5.09E-05 | 8.655546 | UP |
| FAM46A | 2.383528 | 9.796457 | 12.19353 | 7.37E-08 | 5.19E-05 | 8.604572 | UP |
| BMP2 | 1.327921 | 7.139674 | 12.10738 | 7.94E-08 | 5.33E-05 | 8.53538 | UP |
| MXD1 | 2.171273 | 7.702274 | 12.08333 | 8.11E-08 | 5.33E-05 | 8.515967 | UP |
| ADM | 2.25151 | 9.408549 | 11.8836 | 9.67E-08 | 6.15E-05 | 8.353041 | UP |
| NFKBIA | 2.023645 | 11.66215 | 11.77791 | 1.06E-07 | 6.55E-05 | 8.265587 | UP |
| CRY1 | 1.588158 | 6.76392 | 11.70857 | 1.13E-07 | 6.75E-05 | 8.207734 | UP |
| CREM | 1.494678 | 7.935348 | 11.65807 | 1.18E-07 | 6.86E-05 | 8.165362 | UP |
| NCOA7 | 1.684392 | 11.10561 | 11.58883 | 1.26E-07 | 7.10E-05 | 8.106939 | UP |
| ADRB2 | 2.472008 | 8.768045 | 11.45707 | 1.42E-07 | 7.78E-05 | 7.994694 | UP |
| TNFRSF1B | 1.295825 | 8.572171 | 11.41986 | 1.47E-07 | 7.83E-05 | 7.962749 | UP |
| PLEKHB2 | 1.050567 | 8.495149 | 11.06342 | 2.05E-07 | 0.000106 | 7.650848 | UP |
| KLHL15 | 2.307417 | 8.094697 | 11.0038 | 2.17E-07 | 0.00011 | 7.597624 | UP |
| SPIDR | 1.035992 | 8.565871 | 10.91689 | 2.35E-07 | 0.000115 | 7.519496 | UP |
| SOX7 | 2.091637 | 7.062542 | 10.90192 | 2.39E-07 | 0.000115 | 7.505977 | UP |
| CCL3 | 3.000324 | 8.519586 | 10.846 | 2.52E-07 | 0.000118 | 7.455289 | UP |
| TRIB1 | 3.298188 | 10.55598 | 10.52884 | 3.43E-07 | 0.000157 | 7.162541 | UP |
| THAP9-AS1 | 1.444138 | 6.560335 | 10.42743 | 3.79E-07 | 0.000167 | 7.067009 | UP |
| RUFY2 | 1.592329 | 6.26994 | 10.42133 | 3.81E-07 | 0.000167 | 7.06123 | UP |
| PPP1R15A | 2.127679 | 8.663057 | 10.35922 | 4.05E-07 | 0.000174 | 7.002221 | UP |
| LDLR | 2.952748 | 10.35363 | 10.30566 | 4.28E-07 | 0.000178 | 6.951044 | UP |
| KLF4 | 3.553855 | 9.518276 | 10.2717 | 4.42E-07 | 0.000178 | 6.918449 | UP |
| BTG3 | 1.303219 | 8.392779 | 10.27166 | 4.42E-07 | 0.000178 | 6.918412 | UP |
| BAG3 | 2.608327 | 10.21312 | 10.24777 | 4.53E-07 | 0.000178 | 6.895418 | UP |
| CXCL8 | 3.935917 | 8.699235 | 10.23424 | 4.59E-07 | 0.000178 | 6.88238 | UP |
| KLF5 | 1.198736 | 6.758394 | 10.13975 | 5.05E-07 | 0.000185 | 6.790786 | UP |
| SERPINE1 | 3.968724 | 10.32538 | 10.13866 | 5.06E-07 | 0.000185 | 6.789721 | UP |
| PER2 | 1.913493 | 8.389314 | 10.13732 | 5.07E-07 | 0.000185 | 6.788424 | UP |
| GATA6 | 1.571826 | 7.675185 | 10.04794 | 5.55E-07 | 0.000192 | 6.700971 | UP |
| DUSP6 | 2.963194 | 10.46472 | 10.02613 | 5.67E-07 | 0.000193 | 6.679519 | UP |
| RASD1 | 1.933223 | 11.70498 | 9.996959 | 5.85E-07 | 0.000194 | 6.650746 | UP |
| MCL1 | 1.322099 | 11.70466 | 9.984907 | 5.92E-07 | 0.000194 | 6.638835 | UP |
| ZBTB43 | 1.201326 | 8.685088 | 9.972951 | 5.99E-07 | 0.000194 | 6.627005 | UP |
| SPSB1 | 1.698007 | 8.999808 | 9.936163 | 6.22E-07 | 0.000195 | 6.590517 | UP |
| ICAM1 | 1.882793 | 9.888996 | 9.93099 | 6.26E-07 | 0.000195 | 6.585376 | UP |
| WEE1 | 3.730199 | 9.220181 | 9.919415 | 6.33E-07 | 0.000195 | 6.573862 | UP |
| MAP1LC3B | 1.39349 | 9.473261 | 9.88445 | 6.57E-07 | 0.000199 | 6.539001 | UP |
| EMP1 | 2.603058 | 8.712549 | 9.76418 | 7.44E-07 | 0.000222 | 6.418158 | UP |
| UPP1 | 1.373373 | 7.961086 | 9.747937 | 7.57E-07 | 0.000223 | 6.401727 | UP |
| FRMD4B | 2.725997 | 8.226225 | 9.711769 | 7.86E-07 | 0.000228 | 6.365043 | UP |
| KLF6 | 2.908898 | 10.49535 | 9.679865 | 8.13E-07 | 0.000232 | 6.332574 | UP |
| IER5 | 2.736918 | 8.054784 | 9.606971 | 8.78E-07 | 0.000247 | 6.257999 | UP |
| RIPK4 | 1.54746 | 9.635712 | 9.590873 | 8.93E-07 | 0.000248 | 6.241456 | UP |
| LATS2 | 1.123934 | 8.619196 | 9.567857 | 9.15E-07 | 0.00025 | 6.217758 | UP |
| ZNF281 | 1.210007 | 8.685891 | 9.478567 | 1.01E-06 | 0.000272 | 6.125302 | UP |
| ZC3H12A | 2.237674 | 8.034459 | 9.454706 | 1.03E-06 | 0.000274 | 6.100454 | UP |
| RGCC | 3.147503 | 7.356347 | 9.446891 | 1.04E-06 | 0.000274 | 6.092303 | UP |
| THBD | 1.316875 | 8.17408 | 9.379857 | 1.12E-06 | 0.000282 | 6.022122 | UP |
| B3GNT5 | 1.979372 | 7.348018 | 9.349788 | 1.16E-06 | 0.000282 | 5.990488 | UP |
| TNFRSF10D | 1.525438 | 7.445655 | 9.349236 | 1.16E-06 | 0.000282 | 5.989906 | UP |
| BHLHE40 | 1.872222 | 10.64182 | 9.346859 | 1.16E-06 | 0.000282 | 5.987401 | UP |
| GADD45A | 1.709669 | 10.08595 | 9.226914 | 1.32E-06 | 0.000315 | 5.860213 | UP |
| BCL2A1 | 2.603128 | 6.61375 | 9.223164 | 1.33E-06 | 0.000315 | 5.856212 | UP |
| REL | 2.039817 | 7.757533 | 9.211951 | 1.34E-06 | 0.000315 | 5.844238 | UP |
| PMAIP1 | 1.892431 | 6.290114 | 9.1547 | 1.43E-06 | 0.000332 | 5.782892 | UP |
| PNPLA8 | 1.320742 | 9.27938 | 9.125779 | 1.48E-06 | 0.000338 | 5.751767 | UP |
| GADD45B | 1.618297 | 10.53287 | 9.069945 | 1.57E-06 | 0.000352 | 5.691418 | UP |
| CYTIP | 1.515491 | 6.675086 | 9.037912 | 1.63E-06 | 0.00036 | 5.656642 | UP |
| MAFK | 1.916285 | 8.179591 | 9.013407 | 1.67E-06 | 0.000366 | 5.62996 | UP |
| DNAJB1 | 2.671042 | 10.54181 | 8.958144 | 1.78E-06 | 0.000385 | 5.569545 | UP |
| PFKFB3 | 2.897599 | 8.651035 | 8.934313 | 1.83E-06 | 0.000391 | 5.543388 | UP |
| ERN1 | 1.818962 | 8.575027 | 8.817234 | 2.09E-06 | 0.000437 | 5.413952 | UP |
| VCPKMT | 1.174983 | 6.293899 | 8.752435 | 2.24E-06 | 0.000466 | 5.341647 | UP |
| CCDC71L | 2.004677 | 9.158885 | 8.730298 | 2.30E-06 | 0.000473 | 5.316835 | UP |
| PPP1R15B | 1.625668 | 9.998544 | 8.601247 | 2.67E-06 | 0.000537 | 5.17107 | UP |
| G0S2 | 2.347007 | 11.41419 | 8.560665 | 2.80E-06 | 0.000557 | 5.124832 | UP |
| GPR183 | 1.392901 | 6.072204 | 8.538073 | 2.87E-06 | 0.000565 | 5.099008 | UP |
| APOLD1 | 2.446879 | 8.025684 | 8.530473 | 2.90E-06 | 0.000565 | 5.090308 | UP |
| ETS2 | 1.189551 | 10.59326 | 8.496674 | 3.01E-06 | 0.000582 | 5.051531 | UP |
| BAZ1A | 1.475665 | 7.884925 | 8.397305 | 3.39E-06 | 0.000648 | 4.936744 | UP |
| TNFRSF10B | 1.293665 | 8.87283 | 8.367174 | 3.51E-06 | 0.000665 | 4.901705 | UP |
| CSRNP1 | 1.788407 | 8.962367 | 8.34825 | 3.59E-06 | 0.000666 | 4.879643 | UP |
| RIPK2 | 1.050844 | 8.756736 | 8.345162 | 3.60E-06 | 0.000666 | 4.87604 | UP |
| HSPA1A | 2.903586 | 11.94399 | 8.338189 | 3.63E-06 | 0.000666 | 4.867897 | UP |
| EPHA2 | 1.837494 | 8.334845 | 8.331871 | 3.66E-06 | 0.000666 | 4.860514 | UP |
| CD55 | 1.193951 | 8.581375 | 8.326176 | 3.68E-06 | 0.000666 | 4.853855 | UP |
| NFIL3 | 2.24706 | 10.01236 | 8.310201 | 3.75E-06 | 0.000666 | 4.835156 | UP |
| RSRC2 | 1.061688 | 9.224208 | 8.303288 | 3.78E-06 | 0.000666 | 4.827054 | UP |
| LURAP1L | 1.740122 | 8.497036 | 8.283255 | 3.87E-06 | 0.000676 | 4.803544 | UP |
| HSPH1 | 3.114945 | 10.05915 | 8.240077 | 4.08E-06 | 0.000703 | 4.752705 | UP |
| GNA13 | 1.871769 | 9.545348 | 8.206639 | 4.24E-06 | 0.000721 | 4.71318 | UP |
| EAF1 | 1.202938 | 7.862337 | 8.164091 | 4.47E-06 | 0.000752 | 4.662687 | UP |
| SOCS3 | 2.05596 | 9.321583 | 8.150492 | 4.54E-06 | 0.000758 | 4.646503 | UP |
| VPS37B | 1.493914 | 6.984677 | 8.119579 | 4.71E-06 | 0.000767 | 4.609626 | UP |
| GJA1 | 2.273768 | 6.79081 | 8.10119 | 4.82E-06 | 0.000778 | 4.587634 | UP |
| IRAK2 | 1.247821 | 8.231713 | 8.05816 | 5.07E-06 | 0.000807 | 4.536009 | UP |
| TP53BP2 | 2.185719 | 8.001436 | 7.991691 | 5.50E-06 | 0.000861 | 4.455811 | UP |
| WTAP | 1.490944 | 9.379615 | 7.97803 | 5.59E-06 | 0.000864 | 4.439259 | UP |
| RGS2 | 2.391434 | 9.15761 | 7.970812 | 5.64E-06 | 0.000864 | 4.430505 | UP |
| RHOU | 1.260068 | 9.390139 | 7.96417 | 5.69E-06 | 0.000864 | 4.422443 | UP |
| HSPA6 | 2.592558 | 8.646668 | 7.934202 | 5.90E-06 | 0.000888 | 4.386 | UP |
| ZFP36 | 1.62552 | 11.33281 | 7.902762 | 6.13E-06 | 0.000911 | 4.347646 | UP |
| CHSY1 | 1.284232 | 7.837899 | 7.870524 | 6.38E-06 | 0.000939 | 4.308188 | UP |
| FEM1C | 1.901105 | 7.311546 | 7.812766 | 6.86E-06 | 0.001001 | 4.237163 | UP |
| PELO | 1.011882 | 8.730382 | 7.78067 | 7.13E-06 | 0.001034 | 4.197511 | UP |
| CDKN1A | 1.442543 | 10.74831 | 7.754214 | 7.37E-06 | 0.001047 | 4.164727 | UP |
| ELMSAN1 | 1.838801 | 8.001648 | 7.750662 | 7.41E-06 | 0.001047 | 4.160319 | UP |
| ARID5B | 1.861305 | 8.083763 | 7.745183 | 7.46E-06 | 0.001047 | 4.153516 | UP |
| SLC19A2 | 2.402079 | 8.564646 | 7.741322 | 7.49E-06 | 0.001047 | 4.14872 | UP |
| PELI1 | 1.995622 | 8.704478 | 7.730034 | 7.60E-06 | 0.001047 | 4.134685 | UP |
| FAM134B | 1.836355 | 8.651695 | 7.726894 | 7.63E-06 | 0.001047 | 4.130778 | UP |
| MIDN | 1.998541 | 8.592003 | 7.724298 | 7.65E-06 | 0.001047 | 4.127548 | UP |
| TMPRSS2 | 1.524018 | 9.477514 | 7.645042 | 8.45E-06 | 0.001133 | 4.028488 | UP |
| DLC1 | 1.305367 | 9.386159 | 7.602946 | 8.91E-06 | 0.001179 | 3.975542 | UP |
| ZFP36L2 | 1.098966 | 10.67745 | 7.581227 | 9.16E-06 | 0.001195 | 3.948133 | UP |
| FAM102A | 1.14558 | 8.600059 | 7.580214 | 9.17E-06 | 0.001195 | 3.946853 | UP |
| CDADC1 | 1.061148 | 6.522157 | 7.576133 | 9.22E-06 | 0.001195 | 3.941695 | UP |
| RHPN2 | 1.262991 | 7.957405 | 7.562914 | 9.38E-06 | 0.0012 | 3.924975 | UP |
| NEDD9 | 1.48989 | 7.824328 | 7.537249 | 9.69E-06 | 0.001224 | 3.892446 | UP |
| NEDD4L | 1.286982 | 7.908014 | 7.490155 | 1.03E-05 | 0.001283 | 3.832531 | UP |
| FOSL2 | 1.308585 | 8.280984 | 7.439799 | 1.10E-05 | 0.00136 | 3.76814 | UP |
| DDX21 | 1.133719 | 11.03407 | 7.430616 | 1.11E-05 | 0.001367 | 3.756361 | UP |
| COQ10B | 1.271773 | 8.737206 | 7.418951 | 1.13E-05 | 0.001377 | 3.741382 | UP |
| C8orf4 | 2.916548 | 8.356848 | 7.415288 | 1.13E-05 | 0.001377 | 3.736676 | UP |
| CCL20 | 3.979054 | 8.998481 | 7.394667 | 1.16E-05 | 0.001397 | 3.710142 | UP |
| IER2 | 1.986289 | 10.42066 | 7.3329 | 1.26E-05 | 0.001495 | 3.630327 | UP |
| IFRD1 | 1.64369 | 7.536492 | 7.319038 | 1.28E-05 | 0.001513 | 3.612343 | UP |
| SNAI2 | 2.084066 | 7.616473 | 7.304491 | 1.31E-05 | 0.001532 | 3.593443 | UP |
| FOXO1 | 1.313277 | 8.707103 | 7.283443 | 1.34E-05 | 0.001556 | 3.566048 | UP |
| FOS | 3.604034 | 9.460912 | 7.268323 | 1.37E-05 | 0.001578 | 3.54633 | UP |
| RCAN1 | 1.245624 | 10.65706 | 7.153025 | 1.59E-05 | 0.001812 | 3.394954 | UP |
| ANXA1 | 2.102646 | 8.498831 | 7.149533 | 1.60E-05 | 0.001812 | 3.39034 | UP |
| NR4A2 | 2.399632 | 7.471833 | 7.14374 | 1.61E-05 | 0.001814 | 3.382683 | UP |
| MAP3K8 | 1.500304 | 6.723883 | 7.105977 | 1.69E-05 | 0.001877 | 3.332661 | UP |
| ID2 | 1.323204 | 11.96351 | 7.105696 | 1.70E-05 | 0.001877 | 3.332288 | UP |
| DNAJB4 | 2.197173 | 7.406711 | 7.031691 | 1.87E-05 | 0.002043 | 3.233685 | UP |
| AREG | 1.064007 | 4.946085 | 6.992652 | 1.97E-05 | 0.002133 | 3.181364 | UP |
| TSC22D1 | 1.351498 | 11.06301 | 6.986603 | 1.99E-05 | 0.002139 | 3.173239 | UP |
| CCNL1 | 1.674526 | 9.552411 | 6.953892 | 2.08E-05 | 0.002213 | 3.129208 | UP |
| SLC2A3 | 1.413244 | 8.428756 | 6.953147 | 2.08E-05 | 0.002213 | 3.128203 | UP |
| CD97 | 1.09655 | 8.195995 | 6.909165 | 2.20E-05 | 0.002335 | 3.068763 | UP |
| JMJD1C | 1.140398 | 8.349775 | 6.887622 | 2.27E-05 | 0.002374 | 3.039549 | UP |
| ZFAND2A | 1.282979 | 9.300169 | 6.874107 | 2.31E-05 | 0.002392 | 3.021188 | UP |
| MYC | 2.412878 | 9.940353 | 6.859168 | 2.36E-05 | 0.002407 | 3.000863 | UP |
| PDE4B | 1.391885 | 7.87318 | 6.835423 | 2.43E-05 | 0.002459 | 2.968493 | UP |
| BAMBI | 1.137292 | 8.342121 | 6.832123 | 2.45E-05 | 0.002459 | 2.963989 | UP |
| SRGN | 1.061211 | 11.01147 | 6.819911 | 2.49E-05 | 0.002468 | 2.947303 | UP |
| PLK2 | 2.320639 | 8.031948 | 6.818403 | 2.49E-05 | 0.002468 | 2.945241 | UP |
| YOD1 | 1.144846 | 6.06221 | 6.808164 | 2.53E-05 | 0.00249 | 2.931234 | UP |
| IER3 | 1.789729 | 9.621883 | 6.754334 | 2.72E-05 | 0.002653 | 2.857349 | UP |
| HBEGF | 1.321165 | 7.844499 | 6.721349 | 2.84E-05 | 0.002762 | 2.811871 | UP |
| MYADM | 1.631565 | 9.443007 | 6.691354 | 2.96E-05 | 0.002864 | 2.770382 | UP |
| NAMPT | 1.179978 | 11.91347 | 6.669605 | 3.05E-05 | 0.002908 | 2.740219 | UP |
| RND3 | 2.031152 | 10.5397 | 6.663899 | 3.08E-05 | 0.002913 | 2.732295 | UP |
| TM4SF1 | 2.167194 | 10.75907 | 6.636318 | 3.20E-05 | 0.003001 | 2.693924 | UP |
| CXADR | 1.060143 | 9.978336 | 6.627277 | 3.24E-05 | 0.003025 | 2.681322 | UP |
| GDF15 | 1.825147 | 7.71935 | 6.591069 | 3.40E-05 | 0.003136 | 2.630736 | UP |
| ARRDC4 | 1.247951 | 7.649143 | 6.580337 | 3.46E-05 | 0.003165 | 2.615708 | UP |
| BIRC3 | 2.627334 | 9.671634 | 6.555831 | 3.58E-05 | 0.003248 | 2.581326 | UP |
| EGR2 | 1.762094 | 6.831135 | 6.531601 | 3.70E-05 | 0.003329 | 2.547247 | UP |
| KLF9 | 1.464213 | 9.635059 | 6.522842 | 3.74E-05 | 0.003354 | 2.534907 | UP |
| IL1R2 | 1.942388 | 8.293531 | 6.513787 | 3.79E-05 | 0.003382 | 2.522138 | UP |
| RAB8B | 1.056454 | 8.250876 | 6.504245 | 3.84E-05 | 0.003396 | 2.50867 | UP |
| EGR3 | 1.514883 | 6.011325 | 6.462199 | 4.08E-05 | 0.003538 | 2.449166 | UP |
| NRIP1 | 1.297847 | 9.540209 | 6.461434 | 4.08E-05 | 0.003538 | 2.448081 | UP |
| GRAMD3 | 1.144293 | 7.236096 | 6.424948 | 4.30E-05 | 0.003663 | 2.396235 | UP |
| SYBU | 1.4712 | 8.710477 | 6.423884 | 4.30E-05 | 0.003663 | 2.39472 | UP |
| ODC1 | 1.578492 | 10.36367 | 6.403545 | 4.43E-05 | 0.003745 | 2.365732 | UP |
| ETV3 | 1.084421 | 7.976329 | 6.389109 | 4.52E-05 | 0.003806 | 2.34512 | UP |
| SOCS2 | 1.80101 | 8.190543 | 6.337944 | 4.86E-05 | 0.004041 | 2.271823 | UP |
| ZBTB11 | 1.033927 | 6.726329 | 6.325983 | 4.94E-05 | 0.004052 | 2.254633 | UP |
| MTHFD2 | 1.323991 | 6.926538 | 6.323557 | 4.96E-05 | 0.004052 | 2.251144 | UP |
| ZNF267 | 1.123196 | 6.855009 | 6.318038 | 5.00E-05 | 0.004052 | 2.243204 | UP |
| SLC25A33 | 1.622902 | 9.196015 | 6.316025 | 5.01E-05 | 0.004052 | 2.240306 | UP |
| ZBTB21 | 1.572036 | 8.693265 | 6.315606 | 5.02E-05 | 0.004052 | 2.239703 | UP |
| PLIN2 | 1.501981 | 10.99437 | 6.307163 | 5.08E-05 | 0.004059 | 2.227544 | UP |
| SPAG1 | 1.112337 | 5.225093 | 6.306049 | 5.09E-05 | 0.004059 | 2.225939 | UP |
| RALGDS | 1.465696 | 8.71542 | 6.305786 | 5.09E-05 | 0.004059 | 2.22556 | UP |
| RNF138 | 1.395597 | 8.41281 | 6.300611 | 5.13E-05 | 0.004073 | 2.218101 | UP |
| METRNL | 1.411418 | 7.956097 | 6.285911 | 5.23E-05 | 0.004126 | 2.196892 | UP |
| SNRK | 1.198431 | 9.036496 | 6.25203 | 5.49E-05 | 0.004297 | 2.147888 | UP |
| U2SURP | 1.21226 | 7.963731 | 6.178953 | 6.10E-05 | 0.004662 | 2.041621 | UP |
| FLRT3 | 1.155121 | 8.018128 | 6.141944 | 6.44E-05 | 0.004772 | 1.987504 | UP |
| ZSWIM6 | 1.387501 | 8.008787 | 6.141895 | 6.44E-05 | 0.004772 | 1.987432 | UP |
| NFATC2 | 1.012187 | 7.763943 | 6.134933 | 6.50E-05 | 0.004772 | 1.977229 | UP |
| PNP | 1.531478 | 8.976784 | 6.132314 | 6.53E-05 | 0.004772 | 1.973389 | UP |
| NUPL1 | 1.063239 | 7.631706 | 6.127248 | 6.58E-05 | 0.004772 | 1.96596 | UP |
| RNF149 | 1.115909 | 9.008104 | 6.126254 | 6.59E-05 | 0.004772 | 1.964501 | UP |
| RNF19A | 1.643797 | 8.551013 | 6.101229 | 6.83E-05 | 0.00493 | 1.927736 | UP |
| CHD1 | 1.393412 | 8.353921 | 6.098821 | 6.85E-05 | 0.00493 | 1.924193 | UP |
| ACKR3 | 2.461798 | 7.105248 | 6.069502 | 7.15E-05 | 0.005053 | 1.880992 | UP |
| PLOD2 | 1.256392 | 10.058 | 6.018542 | 7.71E-05 | 0.005331 | 1.805603 | UP |
| MESDC1 | 1.114398 | 8.881248 | 6.018394 | 7.71E-05 | 0.005331 | 1.805384 | UP |
| JUNB | 1.28354 | 10.09339 | 6.009569 | 7.81E-05 | 0.005381 | 1.792289 | UP |
| OTUD1 | 1.259723 | 8.547603 | 5.994664 | 7.98E-05 | 0.005462 | 1.770147 | UP |
| THBS1 | 1.937571 | 8.056992 | 5.956437 | 8.44E-05 | 0.005718 | 1.713208 | UP |
| SERPINB9 | 1.620784 | 7.736889 | 5.925334 | 8.84E-05 | 0.005886 | 1.66672 | UP |
| FAM133B | 1.099335 | 7.739405 | 5.899679 | 9.18E-05 | 0.006073 | 1.628269 | UP |
| HCAR3 | 1.376126 | 6.641222 | 5.853841 | 9.83E-05 | 0.006457 | 1.559325 | UP |
| KLHL21 | 1.057575 | 8.220456 | 5.832348 | 0.000101 | 0.006602 | 1.526889 | UP |
| CLK1 | 1.643659 | 8.213854 | 5.812301 | 0.000105 | 0.006757 | 1.496576 | UP |
| EIF2AK3 | 1.192425 | 8.359485 | 5.790805 | 0.000108 | 0.006888 | 1.464006 | UP |
| SOWAHC | 1.845098 | 9.538722 | 5.76219 | 0.000113 | 0.007117 | 1.420542 | UP |
| PIM3 | 1.004902 | 9.93017 | 5.760377 | 0.000113 | 0.007117 | 1.417783 | UP |
| HIVEP1 | 1.634703 | 7.560676 | 5.737962 | 0.000117 | 0.007314 | 1.383647 | UP |
| ARL4A | 1.073531 | 7.960482 | 5.734632 | 0.000117 | 0.007314 | 1.378568 | UP |
| CDC37L1 | 1.635047 | 9.037518 | 5.731704 | 0.000118 | 0.007314 | 1.374102 | UP |
| CSF2RB | 1.150539 | 6.647413 | 5.729529 | 0.000118 | 0.007315 | 1.370784 | UP |
| ADAMTS1 | 1.835188 | 9.326556 | 5.719586 | 0.00012 | 0.007388 | 1.355604 | UP |
| ZXDB | 1.276742 | 7.660446 | 5.71782 | 0.000121 | 0.007388 | 1.352907 | UP |
| GPBP1 | 1.040909 | 9.035325 | 5.696338 | 0.000124 | 0.007457 | 1.320057 | UP |
| CYR61 | 2.476356 | 8.943239 | 5.688779 | 0.000126 | 0.007497 | 1.308482 | UP |
| RHOB | 1.314935 | 12.40074 | 5.679042 | 0.000128 | 0.007585 | 1.29356 | UP |
| F2RL1 | 1.741133 | 8.242088 | 5.65536 | 0.000132 | 0.007748 | 1.257205 | UP |
| DNAJB6 | 1.216223 | 8.38229 | 5.655133 | 0.000132 | 0.007748 | 1.256857 | UP |
| GCA | 1.391087 | 7.330004 | 5.651263 | 0.000133 | 0.007771 | 1.250908 | UP |
| ZNF217 | 1.386807 | 7.363877 | 5.636444 | 0.000136 | 0.007901 | 1.228109 | UP |
| C11orf96 | 2.14757 | 11.25042 | 5.612064 | 0.000141 | 0.00808 | 1.190528 | UP |
| SOX17 | 1.118788 | 6.461201 | 5.588144 | 0.000147 | 0.00827 | 1.153573 | UP |
| ANKRD37 | 2.48502 | 8.456972 | 5.585017 | 0.000147 | 0.008277 | 1.148735 | UP |
| ZFAND5 | 1.580672 | 11.35749 | 5.562607 | 0.000153 | 0.008492 | 1.114026 | UP |
| FILIP1L | 1.265613 | 7.817145 | 5.512078 | 0.000165 | 0.008974 | 1.035491 | UP |
| TNFAIP8 | 1.702114 | 7.575234 | 5.506782 | 0.000166 | 0.008997 | 1.027239 | UP |
| TDG | 1.432286 | 7.480016 | 5.499925 | 0.000168 | 0.009068 | 1.016547 | UP |
| KLF2 | 1.852265 | 8.734565 | 5.43665 | 0.000185 | 0.009731 | 0.917561 | UP |
| PPARGC1A | 2.282836 | 7.985145 | 5.425274 | 0.000188 | 0.009833 | 0.899702 | UP |
| PNRC1 | 1.001972 | 11.45798 | 5.400613 | 0.000196 | 0.010101 | 0.860923 | UP |
| ELL2 | 2.113897 | 9.719349 | 5.368695 | 0.000206 | 0.010397 | 0.8106 | UP |
| SDE2 | 1.54622 | 7.415072 | 5.339741 | 0.000215 | 0.010783 | 0.764823 | UP |
| CDH19 | 1.115015 | 7.063008 | 5.338805 | 0.000216 | 0.010783 | 0.76334 | UP |
| TJP2 | 1.309789 | 9.736912 | 5.320627 | 0.000222 | 0.011065 | 0.734534 | UP |
| IRF1 | 1.285943 | 8.747871 | 5.309982 | 0.000226 | 0.011194 | 0.717644 | UP |
| EXOC8 | 1.337773 | 6.558842 | 5.293667 | 0.000231 | 0.011427 | 0.691725 | UP |
| EHD4 | 1.076086 | 8.877839 | 5.289803 | 0.000233 | 0.011467 | 0.68558 | UP |
| FGR | 1.151276 | 7.075826 | 5.2821 | 0.000236 | 0.011578 | 0.673326 | UP |
| PTGS2 | 2.267982 | 6.231258 | 5.257587 | 0.000245 | 0.011884 | 0.634269 | UP |
| ZBTB1 | 1.022623 | 7.675019 | 5.24697 | 0.000249 | 0.012025 | 0.617326 | UP |
| GRHL1 | 1.083243 | 7.658604 | 5.191305 | 0.000272 | 0.012979 | 0.528225 | UP |
| IVNS1ABP | 1.113217 | 9.066785 | 5.189327 | 0.000273 | 0.012979 | 0.525052 | UP |
| VCAN | 1.795955 | 7.943033 | 5.170841 | 0.000281 | 0.013333 | 0.495358 | UP |
| RCL1 | 1.753186 | 9.816266 | 5.155688 | 0.000288 | 0.013592 | 0.470982 | UP |
| PHLDA2 | 1.748982 | 6.982108 | 5.151291 | 0.00029 | 0.013654 | 0.463903 | UP |
| RGS1 | 2.004414 | 7.192871 | 5.141348 | 0.000294 | 0.013805 | 0.447884 | UP |
| CNKSR3 | 1.111668 | 8.596195 | 5.122881 | 0.000303 | 0.014049 | 0.418094 | UP |
| HSPB1 | 1.331064 | 10.82216 | 5.099945 | 0.000314 | 0.014366 | 0.381028 | UP |
| SGMS2 | 1.581251 | 8.416361 | 5.071765 | 0.000329 | 0.014825 | 0.335386 | UP |
| KDM3A | 1.075148 | 7.990038 | 5.048309 | 0.000341 | 0.015167 | 0.297308 | UP |
| SMIM3 | 1.178696 | 6.90701 | 5.047588 | 0.000342 | 0.015167 | 0.296137 | UP |
| FOXP1 | 1.312861 | 9.44787 | 5.021888 | 0.000356 | 0.015699 | 0.254324 | UP |
| SKIL | 1.339056 | 7.839078 | 5.020325 | 0.000357 | 0.015703 | 0.251779 | UP |
| CHIC2 | 1.330345 | 7.688106 | 5.008519 | 0.000364 | 0.015862 | 0.232537 | UP |
| USP12 | 1.43796 | 8.268222 | 4.969589 | 0.000387 | 0.016506 | 0.168953 | UP |
| PPIL4 | 1.051464 | 6.285705 | 4.951822 | 0.000399 | 0.016872 | 0.139862 | UP |
| SDS | 2.243802 | 11.48061 | 4.951314 | 0.000399 | 0.016872 | 0.13903 | UP |
| ATP1B3 | 1.158421 | 9.676441 | 4.944682 | 0.000403 | 0.016981 | 0.128161 | UP |
| IRS2 | 1.584373 | 10.45401 | 4.94232 | 0.000405 | 0.017009 | 0.124288 | UP |
| CRISPLD2 | 1.295012 | 8.453235 | 4.903892 | 0.000431 | 0.017687 | 0.06117 | UP |
| P2RX7 | 1.170385 | 6.600691 | 4.885173 | 0.000444 | 0.018045 | 0.030351 | UP |
| KPNA2 | 1.004427 | 8.556496 | 4.874295 | 0.000452 | 0.018254 | 0.012419 | UP |
| NR0B2 | 1.335003 | 9.912606 | 4.862709 | 0.000461 | 0.018445 | -0.0067 | UP |
| STK17A | 1.314876 | 8.398186 | 4.819486 | 0.000494 | 0.019327 | -0.07818 | UP |
| GADD45G | 1.13139 | 8.551325 | 4.819476 | 0.000494 | 0.019327 | -0.07819 | UP |
| RORA | 1.722676 | 8.494726 | 4.816009 | 0.000497 | 0.019374 | -0.08394 | UP |
| N4BP2L1 | 1.357453 | 9.480599 | 4.806692 | 0.000505 | 0.019541 | -0.09938 | UP |
| ZCCHC2 | 1.002082 | 8.540236 | 4.779583 | 0.000528 | 0.020035 | -0.14439 | UP |
| TCERG1 | 1.248984 | 7.109472 | 4.774602 | 0.000532 | 0.020048 | -0.15267 | UP |
| MAP3K14 | 1.024996 | 7.157528 | 4.739327 | 0.000564 | 0.020814 | -0.2114 | UP |
| TP53INP1 | 1.19421 | 10.41126 | 4.73909 | 0.000564 | 0.020814 | -0.2118 | UP |
| CXCL1 | 1.506322 | 6.249021 | 4.735832 | 0.000567 | 0.020845 | -0.21723 | UP |
| TRIM35 | 1.097739 | 7.467694 | 4.716482 | 0.000585 | 0.021203 | -0.24953 | UP |
| UGCG | 1.215193 | 9.549346 | 4.705893 | 0.000596 | 0.02138 | -0.26722 | UP |
| PCK1 | 1.746378 | 12.67516 | 4.695 | 0.000606 | 0.021611 | -0.28544 | UP |
| SOCS6 | 1.477361 | 7.419036 | 4.67622 | 0.000626 | 0.022211 | -0.31689 | UP |
| GAREM | 1.440196 | 8.161025 | 4.662435 | 0.00064 | 0.022515 | -0.34 | UP |
| PLEKHF2 | 1.081869 | 7.607773 | 4.655804 | 0.000647 | 0.022592 | -0.35113 | UP |
| CXCR4 | 1.246168 | 7.838144 | 4.646262 | 0.000657 | 0.022687 | -0.36715 | UP |
| FOXO3 | 1.084707 | 9.732124 | 4.62107 | 0.000685 | 0.023348 | -0.4095 | UP |
| CXCL3 | 1.440065 | 6.369534 | 4.598962 | 0.000711 | 0.023832 | -0.44673 | UP |
| ANGPTL4 | 1.545149 | 9.737849 | 4.593012 | 0.000718 | 0.024028 | -0.45677 | UP |
| ZBTB2 | 1.318885 | 6.415024 | 4.587746 | 0.000724 | 0.024158 | -0.46565 | UP |
| MASTL | 1.008851 | 5.660918 | 4.561093 | 0.000757 | 0.024539 | -0.51066 | UP |
| CHORDC1 | 1.633292 | 8.329116 | 4.560581 | 0.000758 | 0.024539 | -0.51152 | UP |
| BIRC2 | 1.041043 | 9.331829 | 4.558925 | 0.00076 | 0.024539 | -0.51432 | UP |
| BACH1 | 1.063301 | 7.984593 | 4.558403 | 0.000761 | 0.024539 | -0.5152 | UP |
| MED17 | 1.071952 | 7.137571 | 4.540448 | 0.000784 | 0.025113 | -0.54558 | UP |
| SLC25A32 | 1.299227 | 7.636904 | 4.50425 | 0.000833 | 0.026022 | -0.60695 | UP |
| PANX1 | 1.164464 | 8.522156 | 4.470247 | 0.000882 | 0.026988 | -0.66474 | UP |
| GBP1 | 1.563087 | 9.992472 | 4.453406 | 0.000907 | 0.027549 | -0.69341 | UP |
| ZNF165 | 1.571217 | 5.336027 | 4.442786 | 0.000924 | 0.027875 | -0.71151 | UP |
| SELL | 1.487541 | 7.199343 | 4.436562 | 0.000933 | 0.028009 | -0.72212 | UP |
| GNL2 | 1.069248 | 7.49209 | 4.434063 | 0.000937 | 0.028009 | -0.72639 | UP |
| SMURF2 | 1.032086 | 5.991663 | 4.406026 | 0.000983 | 0.02882 | -0.77427 | UP |
| NCEH1 | 1.358281 | 5.442815 | 4.397076 | 0.000998 | 0.029086 | -0.78957 | UP |
| SH3BP4 | 1.036574 | 9.043493 | 4.389256 | 0.001011 | 0.029257 | -0.80295 | UP |
| RP2 | 1.162154 | 5.854156 | 4.344654 | 0.00109 | 0.030874 | -0.87939 | UP |
| NBPF8 | 1.071668 | 6.859837 | 4.326566 | 0.001124 | 0.031488 | -0.91046 | UP |
| RNF168 | 1.054699 | 7.258507 | 4.321307 | 0.001134 | 0.031669 | -0.91949 | UP |
| MIR22HG | 1.12771 | 8.579594 | 4.268606 | 0.001241 | 0.033097 | -1.01025 | UP |
| NOP58 | 1.023225 | 9.289009 | 4.264134 | 0.001251 | 0.033261 | -1.01796 | UP |
| G6PC | 1.952883 | 9.936817 | 4.248921 | 0.001284 | 0.033907 | -1.04423 | UP |
| SPATA13 | 1.034014 | 8.21085 | 4.223098 | 0.001342 | 0.034742 | -1.08886 | UP |
| RP11-307N16.6 | 1.034014 | 8.21085 | 4.223098 | 0.001342 | 0.034742 | -1.08886 | UP |
| C2CD4A | 1.437062 | 6.150536 | 4.199849 | 0.001396 | 0.035639 | -1.12911 | UP |
| TFPI2 | 1.196207 | 8.461659 | 4.174016 | 0.001459 | 0.036416 | -1.1739 | UP |
| SIK1 | 1.408269 | 10.30554 | 4.151213 | 0.001518 | 0.037144 | -1.21349 | UP |
| ZNF331 | 1.140675 | 8.034983 | 4.150615 | 0.001519 | 0.037144 | -1.21453 | UP |
| FABP5 | 1.099756 | 8.085716 | 4.148336 | 0.001525 | 0.037158 | -1.21849 | UP |
| STRN3 | 1.177897 | 7.09979 | 4.140358 | 0.001546 | 0.03745 | -1.23236 | UP |
| ALAS1 | 1.394903 | 10.6714 | 4.111187 | 0.001626 | 0.038142 | -1.28311 | UP |
| ADAMTS9 | 1.139998 | 7.524828 | 4.070522 | 0.001745 | 0.039436 | -1.35401 | UP |
| GEM | 1.357732 | 7.67008 | 4.06027 | 0.001776 | 0.039817 | -1.37191 | UP |
| CPEB4 | 1.379248 | 8.581678 | 4.013992 | 0.001924 | 0.041873 | -1.45283 | UP |
| NUP153 | 1.107696 | 8.456727 | 4.011576 | 0.001932 | 0.041944 | -1.45706 | UP |
| TAF13 | 1.057932 | 8.433978 | 3.967199 | 0.002088 | 0.044481 | -1.53484 | UP |
| HES1 | 1.062282 | 8.30927 | 3.962644 | 0.002104 | 0.044689 | -1.54284 | UP |
| SDC4 | 1.036988 | 11.56012 | 3.957774 | 0.002122 | 0.044925 | -1.55139 | UP |
| AGPAT9 | 2.162659 | 7.876185 | 3.941439 | 0.002184 | 0.045684 | -1.58008 | UP |
| KLF11 | 1.055163 | 9.106647 | 3.934584 | 0.00221 | 0.045941 | -1.59212 | UP |
| MAVS | -1.10816 | 8.393057 | -10.1114 | 5.20E-07 | 0.000186 | 6.763161 | DOWN |
| PTPRF | -1.15988 | 10.79956 | -10.0802 | 5.37E-07 | 0.000189 | 6.732619 | DOWN |
| KIAA0100 | -1.29117 | 7.996651 | -8.12958 | 4.66E-06 | 0.000765 | 4.621571 | DOWN |
| HNF4A | -1.25403 | 10.00798 | -7.72745 | 7.62E-06 | 0.001047 | 4.131469 | DOWN |
| RTP3 | -1.09673 | 9.351249 | -7.71521 | 7.74E-06 | 0.001052 | 4.116232 | DOWN |
| MLEC | -1.58198 | 9.955143 | -7.29587 | 1.32E-05 | 0.00154 | 3.582228 | DOWN |
| PARP14 | -1.05993 | 8.766421 | -6.04531 | 7.41E-05 | 0.005179 | 1.845244 | DOWN |
| NDST1 | -1.03755 | 8.90647 | -5.97034 | 8.27E-05 | 0.005639 | 1.733945 | DOWN |
| INSR | -1.29342 | 9.962795 | -5.41333 | 0.000192 | 0.009981 | 0.880937 | DOWN |
| HIPK2 | -1.11847 | 10.03025 | -5.13701 | 0.000296 | 0.013835 | 0.440886 | DOWN |
