# Supplementary material for: Integrated transcriptomic profiling of programmed cell death patterns unveils macrophage-hepatocyte crosstalk via THBS1-CD47 axis in hepatic ischemia-reperfusion injury
Source: Front Immunol. 2026 May 19;17:1769849. doi: 10.3389/fimmu.2026.1769849 (PMC13225957; doi:10.3389/fimmu.2026.1769849)
Supplement: Supplementary file 5 [file Table4.docx]

| **Table S4. Differentially expressed genes in GSE12720.** | | | | | | | |
| --- | --- | --- | --- | --- | --- | --- | --- |
|  | logFC | AveExpr | t | P.Value | adj.P.Val | B | change |
| ATF3 | 3.600807 | 8.351031 | 14.10769 | 9.59E-18 | 1.89E-13 | 29.48359 | UP |
| CCL2 | 3.032267 | 7.909005 | 13.82139 | 1.98E-17 | 1.95E-13 | 28.82134 | UP |
| TNFAIP3 | 1.832396 | 8.102691 | 12.96985 | 1.82E-16 | 1.20E-12 | 26.78559 | UP |
| JUN | 2.63902 | 9.375262 | 11.48741 | 1.08E-14 | 5.30E-11 | 22.99765 | UP |
| SERTAD1 | 1.560864 | 7.644355 | 11.16807 | 2.69E-14 | 9.18E-11 | 22.14011 | UP |
| ZC3H12A | 1.915096 | 6.445582 | 11.15302 | 2.80E-14 | 9.18E-11 | 22.09934 | UP |
| PLK2 | 2.32129 | 7.150035 | 11.10099 | 3.26E-14 | 9.18E-11 | 21.95808 | UP |
| SOCS3 | 2.451579 | 7.880086 | 10.90775 | 5.72E-14 | 1.41E-10 | 21.43007 | UP |
| ADAMTS1 | 2.437027 | 7.610849 | 10.79405 | 7.98E-14 | 1.75E-10 | 21.11688 | UP |
| CCDC71L | 2.114719 | 8.26698 | 10.61807 | 1.34E-13 | 2.55E-10 | 20.6284 | UP |
| CCL4 | 2.36658 | 7.94122 | 10.59774 | 1.42E-13 | 2.55E-10 | 20.5717 | UP |
| MYC | 2.378862 | 9.233143 | 10.33723 | 3.09E-13 | 5.08E-10 | 19.83963 | UP |
| THBS1 | 2.113759 | 7.78713 | 10.17604 | 5.02E-13 | 7.60E-10 | 19.38179 | UP |
| VMP1 | 1.342879 | 9.91641 | 10.0728 | 6.85E-13 | 9.65E-10 | 19.08662 | UP |
| MCL1 | 1.19416 | 10.87997 | 9.740553 | 1.89E-12 | 2.33E-09 | 18.12646 | UP |
| CDKN1A | 1.804162 | 8.678472 | 9.740377 | 1.89E-12 | 2.33E-09 | 18.12595 | UP |
| MAFF | 1.710863 | 7.288471 | 9.686252 | 2.23E-12 | 2.58E-09 | 17.96808 | UP |
| RIPK4 | 1.361324 | 8.400786 | 9.584924 | 3.05E-12 | 3.34E-09 | 17.67144 | UP |
| FOSB | 2.80507 | 7.248092 | 9.409918 | 5.24E-12 | 5.44E-09 | 17.15581 | UP |
| IL1RN | 2.461854 | 9.20147 | 8.992581 | 1.94E-11 | 1.91E-08 | 15.90972 | UP |
| RALGDS | 1.943387 | 7.450082 | 8.88275 | 2.75E-11 | 2.58E-08 | 15.57805 | UP |
| ICAM1 | 1.606935 | 7.91631 | 8.821056 | 3.34E-11 | 2.93E-08 | 15.39108 | UP |
| C5AR1 | 1.138587 | 6.230553 | 8.813281 | 3.42E-11 | 2.93E-08 | 15.36748 | UP |
| G0S2 | 2.002163 | 10.50731 | 8.796524 | 3.61E-11 | 2.97E-08 | 15.3166 | UP |
| PLAUR | 1.146935 | 6.182306 | 8.753991 | 4.14E-11 | 3.17E-08 | 15.1873 | UP |
| ARL5B | 1.897631 | 8.893418 | 8.750277 | 4.19E-11 | 3.17E-08 | 15.176 | UP |
| KLF6 | 2.031764 | 10.26002 | 8.65012 | 5.76E-11 | 4.03E-08 | 14.87059 | UP |
| TGIF1 | 1.479782 | 7.511261 | 8.640974 | 5.93E-11 | 4.03E-08 | 14.84265 | UP |
| TRIB1 | 2.027238 | 9.759677 | 8.594309 | 6.89E-11 | 4.53E-08 | 14.69989 | UP |
| C11orf96 | 2.917052 | 9.53227 | 8.499 | 9.35E-11 | 5.95E-08 | 14.40753 | UP |
| FILIP1L | 1.484515 | 6.699428 | 8.480872 | 9.92E-11 | 6.11E-08 | 14.3518 | UP |
| GADD45B | 1.65526 | 10.10935 | 8.449654 | 1.10E-10 | 6.55E-08 | 14.25575 | UP |
| TIPARP | 1.592202 | 8.330484 | 8.422345 | 1.20E-10 | 6.94E-08 | 14.17162 | UP |
| LDLR | 2.016384 | 9.315576 | 8.406348 | 1.26E-10 | 7.01E-08 | 14.12231 | UP |
| BHLHE40 | 1.752894 | 9.75335 | 8.401455 | 1.28E-10 | 7.01E-08 | 14.10722 | UP |
| CYR61 | 2.158674 | 8.215135 | 8.386036 | 1.35E-10 | 7.17E-08 | 14.05965 | UP |
| DUSP5 | 2.22327 | 6.957268 | 8.300074 | 1.78E-10 | 9.21E-08 | 13.79396 | UP |
| KLF4 | 2.362047 | 7.649817 | 8.277473 | 1.91E-10 | 9.66E-08 | 13.72397 | UP |
| PPP1R15A | 1.439912 | 6.939931 | 8.252242 | 2.07E-10 | 1.02E-07 | 13.64577 | UP |
| NCOA7 | 1.984082 | 9.775801 | 8.180725 | 2.62E-10 | 1.23E-07 | 13.42374 | UP |
| IL1B | 1.373524 | 6.330795 | 8.152285 | 2.87E-10 | 1.31E-07 | 13.33529 | UP |
| DNAJB1 | 1.78224 | 8.432759 | 8.112529 | 3.26E-10 | 1.46E-07 | 13.2115 | UP |
| SDC4 | 1.13913 | 10.05297 | 8.069085 | 3.76E-10 | 1.65E-07 | 13.07605 | UP |
| BAG3 | 1.788353 | 8.181693 | 8.023128 | 4.37E-10 | 1.87E-07 | 12.93255 | UP |
| RND1 | 1.906873 | 8.145943 | 7.929248 | 5.93E-10 | 2.49E-07 | 12.63876 | UP |
| FOSL2 | 1.374654 | 7.34611 | 7.886912 | 6.82E-10 | 2.80E-07 | 12.50599 | UP |
| BTG2 | 1.522295 | 7.602375 | 7.810956 | 8.74E-10 | 3.38E-07 | 12.26735 | UP |
| SLC2A3 | 1.168293 | 6.426936 | 7.772578 | 9.92E-10 | 3.76E-07 | 12.14658 | UP |
| IRF1 | 1.319679 | 7.007952 | 7.715884 | 1.19E-09 | 4.44E-07 | 11.96791 | UP |
| IER2 | 1.305915 | 9.088451 | 7.63057 | 1.58E-09 | 5.67E-07 | 11.69852 | UP |
| ZFP36 | 1.283708 | 10.27306 | 7.582161 | 1.86E-09 | 6.48E-07 | 11.54539 | UP |
| MXD1 | 1.391123 | 6.653935 | 7.578942 | 1.88E-09 | 6.48E-07 | 11.5352 | UP |
| CEBPD | 1.038011 | 10.68741 | 7.568795 | 1.94E-09 | 6.59E-07 | 11.50307 | UP |
| ELMSAN1 | 1.357649 | 7.132394 | 7.528302 | 2.22E-09 | 7.28E-07 | 11.37479 | UP |
| CCL20 | 2.540842 | 6.634386 | 7.521053 | 2.27E-09 | 7.34E-07 | 11.35181 | UP |
| JUND | 1.422212 | 9.73143 | 7.448779 | 2.88E-09 | 9.02E-07 | 11.12248 | UP |
| RGS2 | 1.678964 | 8.562759 | 7.432527 | 3.04E-09 | 9.37E-07 | 11.07085 | UP |
| CSRNP1 | 1.814733 | 7.946808 | 7.426392 | 3.11E-09 | 9.41E-07 | 11.05136 | UP |
| TM4SF1 | 1.463464 | 9.044392 | 7.389991 | 3.50E-09 | 1.05E-06 | 10.93564 | UP |
| SLC20A1 | 1.743064 | 8.808459 | 7.36484 | 3.81E-09 | 1.12E-06 | 10.85563 | UP |
| RASD1 | 2.416942 | 9.228497 | 7.361463 | 3.85E-09 | 1.12E-06 | 10.84488 | UP |
| SPSB1 | 1.641777 | 6.959354 | 7.310775 | 4.55E-09 | 1.30E-06 | 10.68348 | UP |
| ETS2 | 1.211284 | 9.579006 | 7.28912 | 4.89E-09 | 1.38E-06 | 10.61448 | UP |
| TSC22D2 | 2.024832 | 7.914105 | 7.273104 | 5.16E-09 | 1.43E-06 | 10.56342 | UP |
| IER3 | 1.904805 | 8.597347 | 7.145398 | 7.89E-09 | 2.10E-06 | 10.15571 | UP |
| NAMPT | 1.220528 | 10.66786 | 7.099902 | 9.18E-09 | 2.41E-06 | 10.01022 | UP |
| IER5 | 1.690971 | 6.968991 | 7.032445 | 1.15E-08 | 2.98E-06 | 9.794282 | UP |
| ELF3 | 1.236526 | 7.720518 | 6.988613 | 1.33E-08 | 3.40E-06 | 9.653845 | UP |
| CKS2 | 2.075616 | 7.640075 | 6.974548 | 1.39E-08 | 3.52E-06 | 9.60876 | UP |
| HBEGF | 1.32963 | 6.407867 | 6.884342 | 1.88E-08 | 4.70E-06 | 9.319382 | UP |
| JUNB | 1.647984 | 7.909671 | 6.873934 | 1.95E-08 | 4.79E-06 | 9.285969 | UP |
| ARID5B | 1.15681 | 7.080091 | 6.871361 | 1.97E-08 | 4.79E-06 | 9.277708 | UP |
| ADM | 1.657085 | 8.440119 | 6.833053 | 2.24E-08 | 5.37E-06 | 9.154687 | UP |
| EPHA2 | 1.469572 | 6.784017 | 6.725055 | 3.21E-08 | 7.62E-06 | 8.807567 | UP |
| WEE1 | 1.66713 | 7.636138 | 6.673146 | 3.82E-08 | 8.96E-06 | 8.640586 | UP |
| KLF2 | 1.035664 | 7.450071 | 6.653123 | 4.08E-08 | 9.36E-06 | 8.576154 | UP |
| FAM46A | 1.471089 | 9.287241 | 6.653112 | 4.08E-08 | 9.36E-06 | 8.576119 | UP |
| SERPINE1 | 2.29035 | 8.485265 | 6.584117 | 5.14E-08 | 1.16E-05 | 8.354021 | UP |
| FPR1 | 1.194489 | 7.90009 | 6.57484 | 5.31E-08 | 1.18E-05 | 8.324151 | UP |
| PIM3 | 1.01148 | 8.372869 | 6.495802 | 6.92E-08 | 1.48E-05 | 8.069585 | UP |
| ATP1B3 | 1.022536 | 7.95388 | 6.467857 | 7.59E-08 | 1.59E-05 | 7.979557 | UP |
| IL15RA | 1.010043 | 7.484648 | 6.46565 | 7.65E-08 | 1.59E-05 | 7.972447 | UP |
| NFIL3 | 1.882023 | 9.78508 | 6.464647 | 7.68E-08 | 1.59E-05 | 7.969214 | UP |
| PELI1 | 1.529336 | 7.208121 | 6.396048 | 9.66E-08 | 1.93E-05 | 7.748172 | UP |
| CCNL1 | 1.125942 | 9.344945 | 6.310327 | 1.29E-07 | 2.49E-05 | 7.471919 | UP |
| SPRY2 | 1.251466 | 7.203052 | 6.276235 | 1.44E-07 | 2.74E-05 | 7.362051 | UP |
| MAFK | 1.437199 | 7.421729 | 6.260795 | 1.52E-07 | 2.85E-05 | 7.31229 | UP |
| BIRC3 | 1.240523 | 5.652539 | 6.245628 | 1.60E-07 | 2.97E-05 | 7.263416 | UP |
| HSPA1A | 1.604524 | 10.58345 | 6.209108 | 1.81E-07 | 3.30E-05 | 7.14573 | UP |
| TNFRSF12A | 1.369485 | 7.81999 | 6.177473 | 2.01E-07 | 3.60E-05 | 7.043801 | UP |
| VCAN | 1.503331 | 6.459633 | 6.143223 | 2.26E-07 | 4.00E-05 | 6.933458 | UP |
| FRMD4B | 1.435969 | 6.38736 | 6.122173 | 2.42E-07 | 4.26E-05 | 6.86565 | UP |
| IL1RL1 | 1.53246 | 7.758944 | 6.098394 | 2.62E-07 | 4.57E-05 | 6.789062 | UP |
| CXCL8 | 1.901626 | 6.671449 | 6.08865 | 2.71E-07 | 4.68E-05 | 6.757681 | UP |
| ODC1 | 1.779148 | 10.52805 | 6.071238 | 2.87E-07 | 4.88E-05 | 6.701609 | UP |
| SGK1 | 1.426389 | 10.35982 | 6.049762 | 3.09E-07 | 5.20E-05 | 6.63246 | UP |
| MAP1LC3B | 1.024584 | 8.58543 | 6.035229 | 3.24E-07 | 5.36E-05 | 6.585675 | UP |
| SOCS1 | 1.026726 | 6.30737 | 6.031435 | 3.28E-07 | 5.39E-05 | 6.573463 | UP |
| CCL3 | 1.788483 | 7.151373 | 6.027063 | 3.33E-07 | 5.42E-05 | 6.559388 | UP |
| NR4A3 | 1.349149 | 6.561475 | 5.988381 | 3.79E-07 | 6.02E-05 | 6.434892 | UP |
| AKAP12 | 1.060877 | 8.178255 | 5.936223 | 4.51E-07 | 6.95E-05 | 6.2671 | UP |
| PHLDA2 | 1.405821 | 6.2036 | 5.918833 | 4.78E-07 | 7.25E-05 | 6.211179 | UP |
| EMP1 | 1.08546 | 6.398877 | 5.887994 | 5.30E-07 | 7.98E-05 | 6.112033 | UP |
| BCL3 | 1.147053 | 7.643807 | 5.797993 | 7.17E-07 | 0.000105 | 5.822927 | UP |
| NFKBIZ | 1.52461 | 9.27446 | 5.781041 | 7.58E-07 | 0.00011 | 5.768513 | UP |
| KLHL15 | 1.249937 | 7.440224 | 5.682283 | 1.05E-06 | 0.000147 | 5.451843 | UP |
| EGR2 | 1.698807 | 5.868263 | 5.590292 | 1.43E-06 | 0.000187 | 5.157425 | UP |
| CDC37L1 | 1.249021 | 9.160302 | 5.540256 | 1.69E-06 | 0.000218 | 4.997541 | UP |
| CREM | 1.147561 | 7.89954 | 5.495889 | 1.96E-06 | 0.000248 | 4.855939 | UP |
| RGCC | 2.17056 | 6.173157 | 5.494354 | 1.97E-06 | 0.000248 | 4.851042 | UP |
| TNFRSF10D | 1.050262 | 7.335702 | 5.478982 | 2.08E-06 | 0.000257 | 4.80202 | UP |
| PTGS2 | 1.473386 | 4.736977 | 5.467143 | 2.16E-06 | 0.000266 | 4.76428 | UP |
| HSPH1 | 1.384935 | 8.883281 | 5.447395 | 2.31E-06 | 0.000281 | 4.701355 | UP |
| VNN3 | 1.228937 | 7.11039 | 5.401783 | 2.68E-06 | 0.000317 | 4.556154 | UP |
| DDX21 | 1.197334 | 9.722143 | 5.355568 | 3.13E-06 | 0.00036 | 4.409238 | UP |
| SIK1 | 1.416067 | 9.013848 | 5.354476 | 3.14E-06 | 0.00036 | 4.405769 | UP |
| PFKFB3 | 1.189904 | 7.971554 | 5.33328 | 3.37E-06 | 0.00038 | 4.338464 | UP |
| SGMS2 | 1.342631 | 7.351824 | 5.194138 | 5.34E-06 | 0.000572 | 3.897864 | UP |
| ZBTB21 | 1.720362 | 7.476651 | 5.16603 | 5.85E-06 | 0.00062 | 3.809137 | UP |
| ZNF331 | 1.154553 | 6.736529 | 5.079063 | 7.79E-06 | 0.000817 | 3.535271 | UP |
| PHLDA1 | 1.450085 | 8.724061 | 5.032199 | 9.09E-06 | 0.000919 | 3.388122 | UP |
| DUSP6 | 1.080241 | 9.347818 | 5.024824 | 9.31E-06 | 0.000936 | 3.364994 | UP |
| ANXA1 | 1.008859 | 6.882791 | 5.00579 | 9.91E-06 | 0.000982 | 3.305344 | UP |
| APOLD1 | 1.317977 | 6.593993 | 4.998469 | 1.02E-05 | 0.001001 | 3.282413 | UP |
| CD83 | 1.071944 | 7.005736 | 4.964551 | 1.13E-05 | 0.001096 | 3.176289 | UP |
| FHL2 | 1.25157 | 7.045602 | 4.953586 | 1.18E-05 | 0.001131 | 3.142018 | UP |
| NOP58 | 1.092544 | 9.422335 | 4.927748 | 1.28E-05 | 0.001205 | 3.06134 | UP |
| B4GALT5 | 1.033636 | 7.768254 | 4.926711 | 1.28E-05 | 0.001205 | 3.058104 | UP |
| ADRB2 | 1.06371 | 7.395935 | 4.85718 | 1.61E-05 | 0.001456 | 2.841553 | UP |
| CH25H | 1.672109 | 5.381989 | 4.831573 | 1.75E-05 | 0.001554 | 2.762007 | UP |
| RPRD1B | 1.528371 | 8.751736 | 4.713867 | 2.56E-05 | 0.002089 | 2.397897 | UP |
| GRAMD4 | 1.610733 | 7.628631 | 4.679406 | 2.87E-05 | 0.002242 | 2.291796 | UP |
| HSPA6 | 1.22823 | 6.575801 | 4.579081 | 3.96E-05 | 0.003013 | 1.984282 | UP |
| GEM | 1.100375 | 5.578707 | 4.556182 | 4.26E-05 | 0.003169 | 1.914393 | UP |
| BCL2A1 | 1.158654 | 5.125679 | 4.532401 | 4.60E-05 | 0.003356 | 1.841934 | UP |
| KLF10 | 1.114133 | 9.120189 | 4.495565 | 5.17E-05 | 0.003654 | 1.729948 | UP |
| CHORDC1 | 1.175399 | 6.810497 | 4.418972 | 6.60E-05 | 0.004441 | 1.498111 | UP |
| SERPINB9 | 1.249672 | 7.213148 | 4.41369 | 6.71E-05 | 0.004483 | 1.482173 | UP |
| STK17A | 1.216654 | 6.09358 | 4.387463 | 7.30E-05 | 0.004746 | 1.403147 | UP |
| RBBP8 | 1.122167 | 7.419944 | 4.365009 | 7.83E-05 | 0.004991 | 1.335626 | UP |
| SERPINB1 | 1.155206 | 8.197206 | 4.364316 | 7.85E-05 | 0.004991 | 1.333544 | UP |
| S100A8 | 1.433607 | 10.80164 | 4.341626 | 8.44E-05 | 0.005328 | 1.265446 | UP |
| FOS | 2.079517 | 8.45784 | 4.30558 | 9.45E-05 | 0.005876 | 1.157536 | UP |
| NR4A2 | 1.297465 | 6.089799 | 4.24515 | 0.000114 | 0.006763 | 0.977404 | UP |
| S100A9 | 1.190744 | 9.124947 | 4.18075 | 0.00014 | 0.007931 | 0.786545 | UP |
| GRHL1 | 1.228604 | 7.287339 | 4.180285 | 0.00014 | 0.007931 | 0.785169 | UP |
| CHAC1 | 1.184085 | 6.469028 | 4.170583 | 0.000144 | 0.008059 | 0.75652 | UP |
| TGM2 | 1.001175 | 9.180089 | 4.166714 | 0.000146 | 0.008097 | 0.745102 | UP |
| MID1IP1 | 1.079011 | 7.470153 | 3.925557 | 0.000308 | 0.014177 | 0.042417 | UP |
| EGR1 | 1.273952 | 10.32003 | 3.874856 | 0.000359 | 0.01604 | -0.10293 | UP |
| S100A12 | 1.565472 | 6.497005 | 3.848946 | 0.000388 | 0.016832 | -0.17686 | UP |
| FOXQ1 | 1.458349 | 6.0978 | 3.843289 | 0.000395 | 0.017032 | -0.19297 | UP |
| FLRT3 | 1.066406 | 8.000199 | 3.650625 | 0.000704 | 0.024947 | -0.73471 | UP |
| C10orf10 | 1.101487 | 8.399806 | 3.584528 | 0.000856 | 0.028609 | -0.91729 | UP |
| ADCY1 | -1.28584 | 6.465172 | -8.64288 | 5.90E-11 | 4.03E-08 | 14.84849 | DOWN |
| PRKAB2 | -1.36988 | 7.764807 | -6.03842 | 3.20E-07 | 5.35E-05 | 6.595947 | DOWN |
| RTP3 | -1.44172 | 7.694364 | -5.07241 | 7.97E-06 | 0.000822 | 3.514347 | DOWN |
| FRK | -1.00462 | 6.913336 | -4.70584 | 2.63E-05 | 0.002119 | 2.373177 | DOWN |
| FAM13A | -1.25617 | 9.298894 | -3.8362 | 0.000404 | 0.017215 | -0.21314 | DOWN |
| DGAT2 | -1.02003 | 8.174998 | -3.75658 | 0.000513 | 0.020181 | -0.43849 | DOWN |
| RP11-372E1.4 | -1.16152 | 7.927132 | -3.75201 | 0.00052 | 0.020418 | -0.45134 | DOWN |
| SLC22A7 | -1.18298 | 9.176214 | -3.63916 | 0.000728 | 0.025444 | -0.76651 | DOWN |
| GPAM | -1.06552 | 8.51194 | -3.51462 | 0.00105 | 0.032841 | -1.10848 | DOWN |
| ZKSCAN1 | -1.02175 | 8.597739 | -3.47186 | 0.001189 | 0.035843 | -1.2244 | DOWN |
| TNFSF10 | -1.13973 | 8.309916 | -3.37735 | 0.001562 | 0.042587 | -1.47786 | DOWN |
