# Supplementary material for: Integrated transcriptomic profiling of programmed cell death patterns unveils macrophage-hepatocyte crosstalk via THBS1-CD47 axis in hepatic ischemia-reperfusion injury
Source: Front Immunol. 2026 May 19;17:1769849. doi: 10.3389/fimmu.2026.1769849 (PMC13225957; doi:10.3389/fimmu.2026.1769849)
Supplement: Supplementary file 6 [file Table5.docx]

| **Table S5. Differentially expressed programmed cell death-related genes (DE-PCDRGs) in the three datasets.** | | | | | | | |
| --- | --- | --- | --- | --- | --- | --- | --- |
| DE-PCDRGs | |  |  |  |  |  |  |
| ATF3 |  |  |  |  |  |  |  |
| BAG3 |  |  |  |  |  |  |  |
| BCL2A1 |  |  |  |  |  |  |  |
| CDKN1A |  |  |  |  |  |  |  |
| EPHA2 |  |  |  |  |  |  |  |
| G0S2 |  |  |  |  |  |  |  |
| ICAM1 |  |  |  |  |  |  |  |
| JUN |  |  |  |  |  |  |  |
| MCL1 |  |  |  |  |  |  |  |
| NR4A2 |  |  |  |  |  |  |  |
| PPP1R15A | |  |  |  |  |  |  |
| SERPINE1 | |  |  |  |  |  |  |
| THBS1 |  |  |  |  |  |  |  |
| TNFAIP3 |  |  |  |  |  |  |  |
| IRF1 |  |  |  |  |  |  |  |
| MAP1LC3B | |  |  |  |  |  |  |
| PTGS2 |  |  |  |  |  |  |  |
| ADRB2 |  |  |  |  |  |  |  |
| PLK2 |  |  |  |  |  |  |  |
| ZC3H12A |  |  |  |  |  |  |  |
| BIRC3 |  |  |  |  |  |  |  |
| CXCL8 |  |  |  |  |  |  |  |
| PHLDA2 |  |  |  |  |  |  |  |
| MYC |  |  |  |  |  |  |  |
| PFKFB3 |  |  |  |  |  |  |  |
