# Supplementary material for: Integrated transcriptomic profiling of programmed cell death patterns unveils macrophage-hepatocyte crosstalk via THBS1-CD47 axis in hepatic ischemia-reperfusion injury
Source: Front Immunol. 2026 May 19;17:1769849. doi: 10.3389/fimmu.2026.1769849 (PMC13225957; doi:10.3389/fimmu.2026.1769849)
Supplement: Supplementary file 7 [file Table6.docx]

| **Table S6. Average AUC values obtained based on the 101 algorithm combinations for the three datasets.** | | | |
| --- | --- | --- | --- |
| Algorithm combinations | Average AUC values | | |
|  | GSE151648 | GSE14951 | GSE12720 |
| SVM | 1 | 1 | 0.721088435 |
| glmBoost+SVM | 0.971644612 | 1 | 0.950113379 |
| Ridge | 1 | 1 | 0.721088435 |
| Lasso+SVM | 0.956521739 | 1 | 0.986394558 |
| glmBoost+Ridge | 0.971644612 | 1 | 0.950113379 |
| Enet[alpha=0.1] | 1 | 1 | 0.721088435 |
| glmBoost+Enet[alpha=0.1] | 0.971644612 | 1 | 0.950113379 |
| Enet[alpha=0.2] | 1 | 0.84 | 0.697278912 |
| Enet[alpha=0.3] | 1 | 0.92 | 0.723356009 |
| glmBoost+Enet[alpha=0.3] | 0.971644612 | 1 | 0.950113379 |
| glmBoost+Enet[alpha=0.2] | 0.971644612 | 1 | 0.950113379 |
| Enet[alpha=0.4] | 1 | 0.96 | 0.706349206 |
| glmBoost+Enet[alpha=0.4] | 0.971644612 | 1 | 0.950113379 |
| Lasso+glmBoost | 0.956521739 | 1 | 0.98185941 |
| Enet[alpha=0.5] | 0.956521739 | 1 | 0.880952381 |
| glmBoost | 0.971644612 | 1 | 0.950113379 |
| glmBoost+Enet[alpha=0.5] | 0.971644612 | 1 | 0.950113379 |
| Enet[alpha=0.6] | 1 | 1 | 0.880952381 |
| glmBoost+Enet[alpha=0.6] | 0.971644612 | 1 | 0.950113379 |
| glmBoost+Enet[alpha=0.7] | 0.971644612 | 1 | 0.950113379 |
| glmBoost+Enet[alpha=0.8] | 0.956521739 | 1 | 0.98185941 |
| Enet[alpha=0.8] | 0.967863894 | 1 | 0.931972789 |
| Enet[alpha=0.9] | 0.956521739 | 1 | 0.986394558 |
| Lasso | 0.960302457 | 1 | 0.993197279 |
| Enet[alpha=0.7] | 1 | 1 | 0.904761905 |
| glmBoost+Enet[alpha=0.9] | 0.956521739 | 1 | 0.98185941 |
| glmBoost+Lasso | 0.960302457 | 1 | 0.995464853 |
| Lasso+plsRglm | 0.956521739 | 1 | 0.986394558 |
| glmBoost+plsRglm | 0.971644612 | 1 | 0.950113379 |
| glmBoost+Stepglm[forward] | 0.971644612 | 1 | 0.950113379 |
| Lasso+Stepglm[forward] | 0.956521739 | 1 | 0.986394558 |
| RF+SVM | 1 | 1 | 0.80952381 |
| Stepglm[forward] | 1 | 1 | 0.721088435 |
| plsRglm | 1 | 1 | 0.719954649 |
| RF+Ridge | 1 | 1 | 0.80952381 |
| RF+Enet[alpha=0.1] | 1 | 1 | 0.80952381 |
| RF+plsRglm | 1 | 1 | 0.80952381 |
| RF+Stepglm[forward] | 1 | 1 | 0.80952381 |
| RF+Enet[alpha=0.2] | 1 | 1 | 0.80952381 |
| RF+Enet[alpha=0.3] | 1 | 1 | 0.80952381 |
| RF+Enet[alpha=0.6] | 0.994328922 | 1 | 0.880952381 |
| RF+Lasso | 0.969754253 | 1 | 0.968253968 |
| RF+Enet[alpha=0.7] | 0.975425331 | 1 | 0.959183673 |
| RF+Enet[alpha=0.5] | 1 | 1 | 0.80952381 |
| RF+glmBoost | 0.975425331 | 1 | 0.968253968 |
| RF+Enet[alpha=0.9] | 0.969754253 | 1 | 0.968253968 |
| RF+Enet[alpha=0.4] | 1 | 1 | 0.80952381 |
| RF+Enet[alpha=0.8] | 0.969754253 | 1 | 0.968253968 |
| RF+Stepglm[both] | 1 | 1 | 0.880952381 |
| RF+Stepglm[backward] | 1 | 1 | 0.880952381 |
| Stepglm[both]+Ridge | 1 | 0.9 | 0.714285714 |
| Stepglm[backward]+Ridge | 1 | 0.9 | 0.714285714 |
| Stepglm[both]+plsRglm | 1 | 0.9 | 0.714285714 |
| Stepglm[backward]+plsRglm | 1 | 0.9 | 0.714285714 |
| Stepglm[both]+Enet[alpha=0.9] | 0.960302457 | 1 | 0.970521542 |
| Stepglm[backward]+Enet[alpha=0.9] | 0.960302457 | 1 | 0.970521542 |
| Stepglm[both]+Enet[alpha=0.1] | 1 | 0.9 | 0.714285714 |
| Stepglm[backward]+Enet[alpha=0.1] | 1 | 0.9 | 0.714285714 |
| Stepglm[both]+Enet[alpha=0.8] | 0.960302457 | 1 | 0.970521542 |
| Stepglm[backward]+Enet[alpha=0.8] | 0.960302457 | 1 | 0.970521542 |
| Stepglm[both]+Enet[alpha=0.2] | 1 | 0.9 | 0.714285714 |
| Stepglm[backward]+Enet[alpha=0.2] | 1 | 0.9 | 0.714285714 |
| Stepglm[both]+Enet[alpha=0.6] | 1 | 0.9 | 0.714285714 |
| Stepglm[backward]+Enet[alpha=0.6] | 1 | 0.9 | 0.714285714 |
